# The Dynamic Proteome of Oligodendrocyte Lineage Differentiation Features Planar Cell Polarity and Macroautophagy Pathways

**DOI:** 10.1093/gigascience/giaa116

**Published:** 2020-10-31

**Authors:** Paria Pooyan, Razieh Karamzadeh, Mehdi Mirzaei, Anna Meyfour, Ardeshir Amirkhan, Yunqi Wu, Vivek Gupta, Hossein Baharvand, Mohammad Javan, Ghasem Hosseini Salekdeh

**Affiliations:** Department of Molecular Systems Biology, Cell Science Research Center, Royan Institute for Stem Cell Biology and Technology, Banihashem St., ACECR, Tehran 16635-148, Iran; Department of Stem Cells and Developmental Biology, Cell Science Research Center, Royan Institute for Stem Cell Biology and Technology, Banihashem St., ACECR, Tehran 16635-148, Iran; Department of Brain and Cognitive Science, Cell Science Research Center, Royan Institute for Stem Cell Biology and Technology, Banihashem St., ACECR, Tehran 16635-148, Iran; Department of Molecular Systems Biology, Cell Science Research Center, Royan Institute for Stem Cell Biology and Technology, Banihashem St., ACECR, Tehran 16635-148, Iran; Department of Stem Cells and Developmental Biology, Cell Science Research Center, Royan Institute for Stem Cell Biology and Technology, Banihashem St., ACECR, Tehran 16635-148, Iran; Department of Brain and Cognitive Science, Cell Science Research Center, Royan Institute for Stem Cell Biology and Technology, Banihashem St., ACECR, Tehran 16635-148, Iran; Department of Molecular Sciences, Macquarie University, North Ryde, Sydney, NSW 2109, Australia; Australian Proteome Analysis Facility, Macquarie University, North Ryde, NSW 2109, Australia; Basic and Molecular Epidemiology of Gastrointestinal Disorders Research Center, Research Institute for Gastroenterology and Liver Diseases, Shahid Beheshti University of Medical Sciences, Daneshjoo Blv., Velenjak, Tehran 19839-63113, Iran; Australian Proteome Analysis Facility, Macquarie University, North Ryde, NSW 2109, Australia; Australian Proteome Analysis Facility, Macquarie University, North Ryde, NSW 2109, Australia; Department of Clinical Medicine, Macquarie University, North Ryde, Sydney, NSW 2109, Australia; Department of Stem Cells and Developmental Biology, Cell Science Research Center, Royan Institute for Stem Cell Biology and Technology, Banihashem St., ACECR, Tehran 16635-148, Iran; Department of Brain and Cognitive Science, Cell Science Research Center, Royan Institute for Stem Cell Biology and Technology, Banihashem St., ACECR, Tehran 16635-148, Iran; Department of Developmental Biology, University of Science and Culture, Ashrafi Esfahani, Tehran 1461968151, Iran; Department of Brain and Cognitive Science, Cell Science Research Center, Royan Institute for Stem Cell Biology and Technology, Banihashem St., ACECR, Tehran 16635-148, Iran; Department of Physiology, Faculty of Medical Sciences, Tarbiat Modares University, Jalal AleAhmad, Tehran 14115-111, Iran; Department of Molecular Systems Biology, Cell Science Research Center, Royan Institute for Stem Cell Biology and Technology, Banihashem St., ACECR, Tehran 16635-148, Iran; Department of Molecular Sciences, Macquarie University, North Ryde, Sydney, NSW 2109, Australia

**Keywords:** human embryonic stem cell, neural stem cell, progenitor cell, oligodendrocyte, Wnt signalling, autophagy, quantitative proteomics, multiplexed tandem mass tag

## Abstract

**Background:**

Generation of oligodendrocytes is a sophisticated multistep process, the mechanistic underpinnings of which are not fully understood and demand further investigation. To systematically profile proteome dynamics during human embryonic stem cell differentiation into oligodendrocytes, we applied in-depth quantitative proteomics at different developmental stages and monitored changes in protein abundance using a multiplexed tandem mass tag-based proteomics approach.

**Findings:**

Our proteome data provided a comprehensive protein expression profile that highlighted specific expression clusters based on the protein abundances over the course of human oligodendrocyte lineage differentiation. We identified the eminence of the planar cell polarity signalling and autophagy (particularly macroautophagy) in the progression of oligodendrocyte lineage differentiation—the cooperation of which is assisted by 106 and 77 proteins, respectively, that showed significant expression changes in this differentiation process. Furthermore, differentially expressed protein analysis of the proteome profile of oligodendrocyte lineage cells revealed 378 proteins that were specifically upregulated only in 1 differentiation stage. In addition, comparative pairwise analysis of differentiation stages demonstrated that abundances of 352 proteins differentially changed between consecutive differentiation time points.

**Conclusions:**

Our study provides a comprehensive systematic proteomics profile of oligodendrocyte lineage cells that can serve as a resource for identifying novel biomarkers from these cells and for indicating numerous proteins that may contribute to regulating the development of myelinating oligodendrocytes and other cells of oligodendrocyte lineage. We showed the importance of planar cell polarity signalling in oligodendrocyte lineage differentiation and revealed the autophagy-related proteins that participate in oligodendrocyte lineage differentiation.

## Data Description

### Background

Oligodendrocytes (OLs; for abbreviations, please refer to Supplementary Table S1) are the myelinating cells of the central nervous system (CNS) that insulate axons with their multispiral membrane-forming myelin. Therefore, OLs allow swift saltatory conduction of action potentials in the CNS [1]. The functional significance of OLs is manifested through myelin loss, in addition to its damage or dysfunction-related neurological disorders such as multiple sclerosis, optic neuritis, spinal cord injury, and Pelizaeus-Merzbacher disease [2]. Irrespective of its background, myelin loss and nervous system failure in remyelination lead to conduction hindrance along the axonal fibers, followed by the interruption of nerve impulses, degenerative axonal loss, and the accumulation of functional disabilities [3]. Oligodendrocyte progenitor cells (OPCs) are the main source of new OLs that can carry on the remyelination process, while neural stem cells (NSCs) and neural progenitor cells (NPCs) may also contribute in new OL generation. In a nutshell, remyelination demands the activation, recruitment, and OL-differentiation of OPCs, and possibly their predecessors [2].

A deeper understanding of the biology of myelinating OL generation alongside their progenitors can equip us with invaluable tools to achieve proper remyelination and prevent further clinical complications of the diseases related to myelin destruction. To accomplish this goal, it is necessary to understand the ways to (i) improve NSC, NPC, and OPC migration into the required site; (ii) enhance the aforementioned cells' survival especially during this process; and (iii) boost their differentiation into myelinating OLs in the demyelination niche. To fulfill these prospects, we conducted an in-depth quantitative proteomic analysis that spanned the entire course of OL lineage cell generation in an attempt to survey the order, timing, and magnitude of proteome changes during human embryonic stem cell (hESC) differentiation into OL lineage cells. This versatile differentiation model system provides tremendous insight into human OL development, in addition to the information needed for targeted/specific cell-based medical therapies and overall disease modelling [4, 5].

Therefore, we studied the global proteome signature of developing OL by conducting a stepwise differentiation process to differentiate the hESC RH6 line into an OL lineage. This process provided us with cell samples from each of the distinct stages of OL differentiation: hESCs, NSCs, NPCs, pre-OPCs, OPCs, and OLs [6, 7]. We attempted to use the advantage of the sensitive and precise tandem mass tag (TMT)-based quantitative proteomics to spot every stage's specific proteins in OL lineage differentiation and to identify the key proteins of each step achievement. Our study provides an inclusive profile of the proteins involved in Wnt signalling throughout OL lineage differentiation. Our findings put planar cell polarity (PCP) noncanonical Wnt signalling in the spotlight for further analysis of this controversial signalling of OL differentiation. In addition, the proteome of OL lineage differentiation presents an all-embracing autophagy-associated protein profile and accentuates macroautophagy as a valuable contributing factor in OL lineage differentiation.

### Global characterization of protein expression during OL generation

To provide a systematic proteomic profiling map of the representative cells in human OL development, the hESC line Royan H6 (RH6) [8] was differentiated into OL lineage through a well-defined stepwise protocol [6, 7, 9] (Supplementary Fig. S1A). Briefly, hESC (d0, Supplementary Fig. S1B) differentiation was initiated by neural induction through dual SMAD inhibition; within 8 days, we observed the presence of SOX1^+^ NSCs (d8, Supplementary Fig. S1C). Further treatment of NSCs by caudalizing and ventralizing morphogens gave rise to OLIG2^+^ NPCs on day 12 (d12, Supplementary Fig. S1D), which then committed to an OL lineage by day 20 (d20, NKX2.2^+^ pre-OPCs, Supplementary Fig. S1E). Next, maturation of pre-oligodendrocyte progenitor cells (pre-OPCs) was promoted via a chemically defined, growth factor–rich medium, and PDGFRA^+^ OPCs were generated on day 80 (d80, Supplementary Fig. S1F). Finally, PDGFRA^+^ OPCs were terminally differentiated into MBP^+^ OLs (d120, Supplementary Fig. S1G).

**Figure 1:**
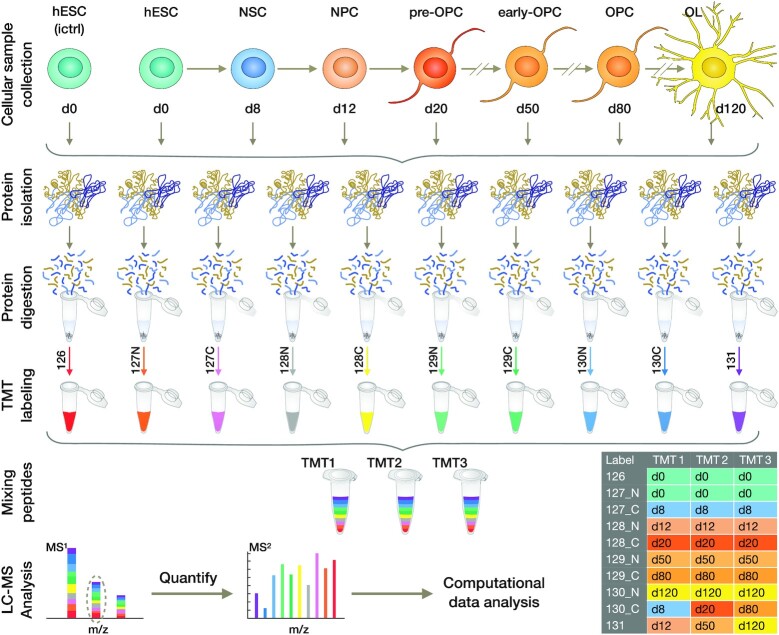
TMT labelling workflow for comprehensive proteome study of hESC differentiation into the OL lineage. For in-depth quantitative proteomic profiling of the human OL lineage, the hESC line (Royan H6) first went through a stepwise differentiation process that resulted in the generation of NSCs, NPCs, pre-OPCs, early-OPCs, OPCs, and OLs (in 3 replicates). Cellular samples were collected at baseline and 6 subsequent consecutive time points (d0, d8, d12, d20, d50, d80, and d120). Their protein contents were extracted and denatured, reduced and alkylated, and then subjected to digestion with lysine-C besides trypsin. Peptides were next quantified and reacted with isobaric TMT reagents across individual batches per sample. After labelling, the samples were combined equally and ionized onto a mass spectrometer. Three TMT experiments were conducted to accommodate all the replicates. In the MS^1^ spectrum, the peptides were detected as a single and identical precursor ion peak. Following fragmentation, in the MS^2^ spectrum, the tags from each differentiation time point produced a unique signature reporter ion. The intensities of these reporter ions were used for the relative quantification of peptides. The identification of peptides was achieved through matching the resulting ion peaks to those indexed fragments in UniProt. The table shows the study design of each TMT experiment. Note that, after Pearson correlation coefficient analysis, the samples analysed in this study have been refined (see TMT data analysis). hESC: human embryonic stem cell; ictrl: inner control; LC: liquid chromatography; MS: mass spectrum; NPC: neural progenitor cell; NSC: neural stem cell; OL: oligodendrocyte; OPC: oligodendrocyte progenitor cell; TMT: tandem mass tag.

Following recapitulation of OL lineage development, the cells were harvested at 6 distinct time points (in 3 biological replicates per time point), which corresponded to the hESC (d0), NSC (d8), NPC (d12), pre-OPC (d20), OPC (d80), and OL (d120) differentiation stages (Fig. 1) [6, 7]. Then to accommodate all the biological replicates of each time point, 3 TMT mass spectrometry experiments were carried out (Fig. 1).

**Figure 2:**
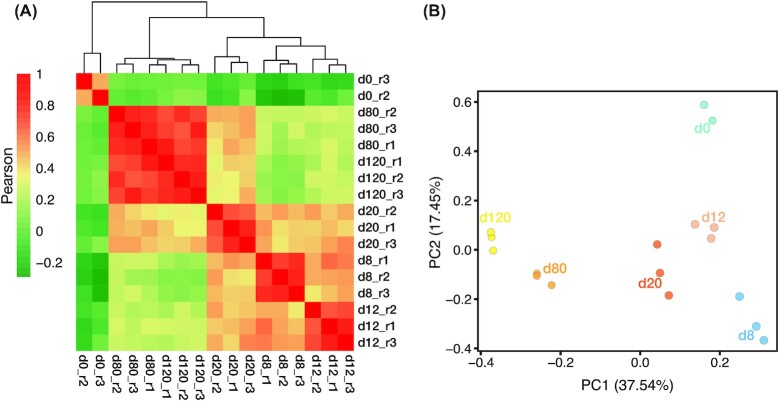
Temporal profiling of protein expression through hESC differentiation into OL lineage. (A) Pearson correlation analysis along with the hierarchical clustering of the 3,855 quantified proteins reveals the biological replicates’ cohesion and dynamics of the proteome during OL lineage differentiation. Red colour denotes stronger correlations. (B) Principal component analysis (PCA) reveals a temporal trend in protein expression patterns. The same colour represents different replicates of the same differentiation time point. PC1 and PC2 axes demonstrate 37.54% and 17.45% of variations.

For in-depth quantitative proteomic analysis, the harvested cells were homogenized and protein extracts of each sample were treated with lysine-C/trypsin sequential digestion. Subsequently, the peptides were quantified and subjected to TMT labelling (Fig. 1), then fractionated and analysed by means of high-resolution nanoflow liquid chromatography positive ion electrospray ionization tandem mass spectrometry (nanoLC/ESI-MS/MS) on a Q Exactive Orbitrap mass spectrometer (Thermo Scientific). Therefore, upon fragmentation in tandem mass spectroscopy (MS/MS) mode, sequence assignment of the MS/MS spectra was achieved using the indexed human UniProt database [10], and next relative protein expression changes were quantified from the fragmentation of the tags, which gave rise to mass reporter ions. The mass spectrometry proteomics data can be retrieved via the ProteomeXchange Consortium [11] through the PRIDE partner repository (accession code: PXD017649). In total, at a false discovery rate (FDR) of 1%, a total of 66,083 peptides and 59,404 unique peptides from 5,753 unique proteins were identified; among them, 3,527 unique proteins were identified within all 3 biological replicates (Supplementary Fig. S2A), and 3,519 unique proteins were quantified across all time points (Supplementary Table S2). Furthermore, 1,056 identified proteins were found to be in common only between 2 replicas (Supplementary Fig. S2A) of which 1,045 proteins were quantified across all time points. Applying a supervised approach [12] to these 1,045 proteins and to proteins that were identified in all time points of the 3 TMT experiments but were not quantified in all of them resulted in the imputation of quantitative measurements of 336 proteins. Importantly, according to the analysis of variance (ANOVA) ∼81% (3,132) of the proteins showed significant changes through the OL lineage differentiation of hESCs (adjusted *P* ≤ 0.05; Supplementary Table S2). Pearson correlation coefficient coupled with hierarchical clustering (using the relative expression for all of the 3,855 quantified proteins) implied a high degree of consistency among sample replicates (Fig. 2A, Supplementary Table S3). The heat map presentation of the protein distribution profiles demonstrates 5 distinct groups associated with the differentiation steps. It also represents d8 (NSC stage), d12 (NPC stage), and d20 (pre-OPC stage) in 1 supergroup, and d20, d80 (OPC stage), and d120 (OL stage) in another supergroup. Therefore, in agreement with the sequential stages of the differentiation process, d20 demonstrated a transition state between the initial and final steps (Fig. 2A). The standard principal component analysis (PCA) was performed to project the proteome profile of each differentiation time point into a 2D space. PCA clustered all 3 replicates of each time point together (Fig. 2B and Supplementary Table S3). To evaluate the functional diversity of the detected proteins, we classified the total proteins into 26 classes using the PANTHER (PANTHER13.1) classification system of 29 indexed parent protein class terms (Supplementary Fig. S2B) [13]. Our data covered a significant number of enriched proteins that included 1,180 enzymes and enzyme modulators, 698 nucleic acid binding and transcription factors (TFs), 425 intra/extracellular trafficking and signalling proteins, 203 cytoskeletal and extracellular matrix (ECM) proteins, and 57 structural and adhesive proteins, indicating the essential role of catalytic activity, gene expression, biosynthesis/trafficking processes, and cellular structure, in addition to their surroundings, in OL differentiation (Supplementary Fig. S2B).

### OL lineage differentiation of the hESCs is led by the cooperation of 3 protein clusters

**Figure 3:**
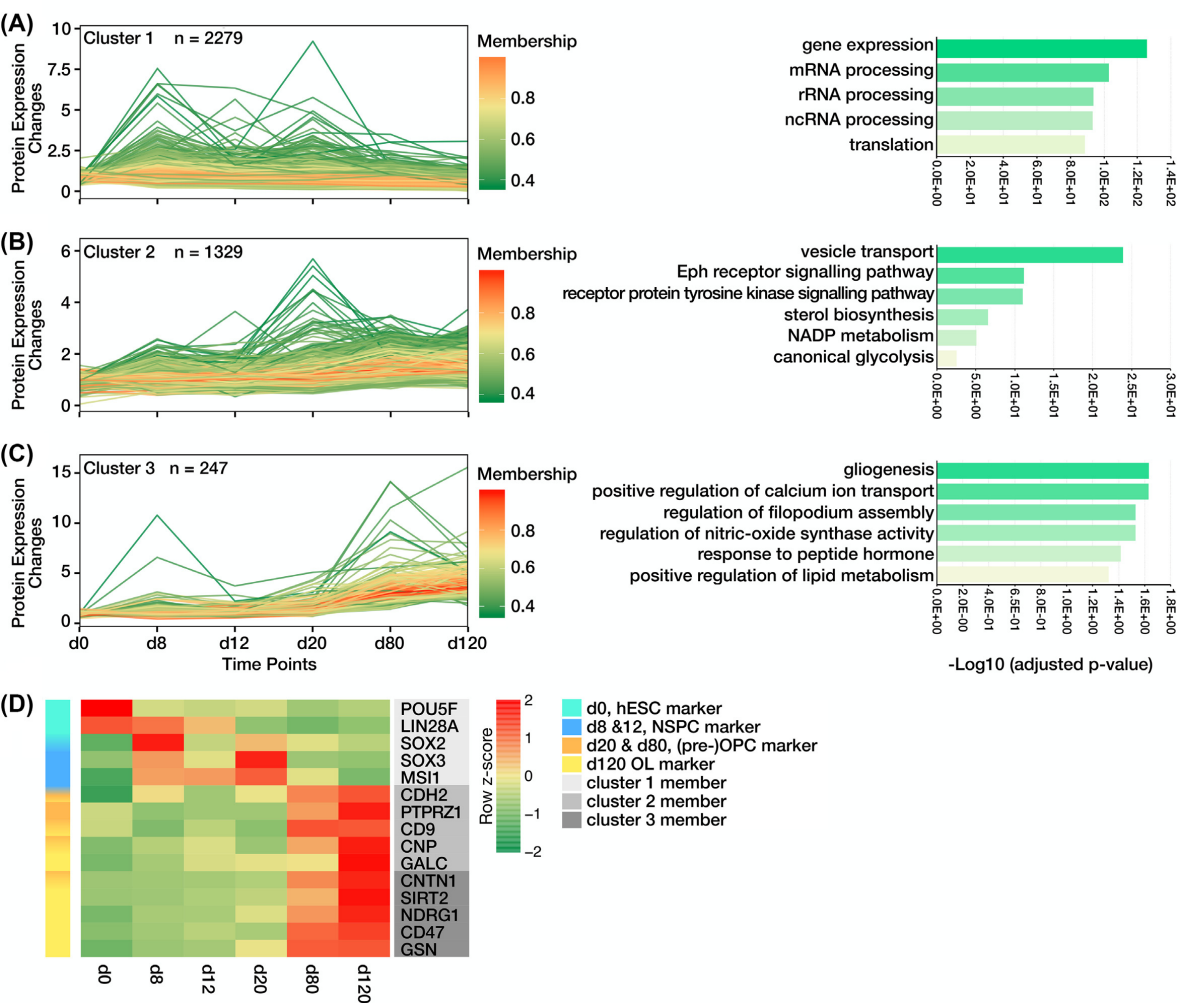
Proteome dynamic landscape of hESC differentiation into OL lineage and expression pattern of marker proteins. Unsupervised clustering of the quantified proteins using the fuzzy c-means algorithm distinguishes 3 protein expression profiles (A–C, *left*) mainly on the basis of protein abundance trends at the initial and final time points. The identified clusters are visualized separately with line charts plotting protein expression level against differentiation time points. Colour-coded membership represents how well a single protein expression pattern fits with the general profile of the cluster. GO enrichment analysis of each cluster was performed with respect to the BPs by Enrichr. Some of the overrepresented BPs of each cluster are shown with bar charts (A–C, *right*). (D) Heat map illustrates the standardized relative protein expression changes of several stage-specific proteins along with differentiation. Markers of early differentiation stages (d0, d8, and d12) are members of cluster 1, which shows a slight decreasing trend. Cluster 2, with a slightly increasing pattern, accommodates CDH2, a marker of NSCs (d8), which is also involved in the regulation of OPC (d80) proliferation and OL (d120) myelination. The heat map also reveals that (pre-)OPC and OL markers are generally assigned to the 2 increasing clusters (clusters 2 and 3). BP: biological processes; Eph: ephrin; GO: Gene Ontology; hESC: human embryonic stem cell; mRNA: messenger RNA; n: number of protein counts in a cluster; NADP: nicotinamide adenine dinucleotide phosphate; ncRNA: non-coding RNA; NSPC: neural stem and progenitor cell; OL: oligodendrocyte; OPC: oligodendrocyte progenitor cell; rRNA: ribosomal RNA.

To get a deep understanding of major functional players during OL lineage differentiation, we explored a dynamic view of the proteome expression during OL differentiation using unsupervised fuzzy c-means clustering on all quantified proteins. As a result, a total of 3,855 proteins (Supplementary Table S3) were segregated into 3 clusters by their expression trends during differentiation. Functional enrichment analysis of the clusters was performed against the Gene Ontology (GO) Biological Process (BP) gene set collection (2018) to ascertain functional groups associated with this differentiation progress (Fig. 3 and Supplementary Table S4).

Cluster 1, consisting of the majority of proteins (2,279), demonstrated a slight decreasing expression profile (d0–d120). Based on the functional enrichment analysis, this cluster mostly contained proteins that contribute to gene expression and translation (Fig. 3A and Supplementary Table S4). The 2 other clusters reflected increasing trends in accordance with the progression and specification of the differentiation process (Fig. 3B and C, and Supplementary Table S4). Both clusters shared some common developmental terms with regard to cellular structure, migration, division, and secretion, which are also in agreement with the OL development; however, Cluster 2, with a slightly increasing pattern, seemed to be more involved in early developmental processes by enrichment of the GO terms predominantly related to the regulation of neural stem and progenitor cells (NSPCs), pre-OPCs, and OPC maintenance and differentiation. This was reflected by GO terms, including “vesicle transport” [14], “ephrin (Eph) receptor signalling pathway” [15], “receptor protein tyrosine kinase signalling pathway” [16], “sterol biosynthesis” [17], “nicotinamide adenine dinucleotide phosphate (NADP) metabolic process” [18], and “canonical glycolysis” [18] (Fig. 3B). On the other hand, Cluster 3, which indicated an upward trend, mostly on d20 (pre-OPC stage) to d120 (OL stage), revealed enrichment of proteins mainly involved in OPCs' differentiation into OLs, OL maturation, and myelin formation, such as “gliogenesis,” “positive regulation of calcium ion transport” [19–22], “regulation of filopodium assembly” [23], “regulation of nitric-oxide synthase activity” [24], “response to peptide hormone” [25], and “positive regulation of lipid metabolism” [26] (Fig. 3C). These results indicated that the functional enrichment of the derived proteome architecture was associated with the corresponding differentiation states.

To confirm the authenticity of our clusters, we checked the expression patterns of several marker proteins related to the OL differentiation stages. The depicted heat map showed that the time-dependent changes of these markers were consistent with their fitted clusters and aligned with the progression of the differentiation process (Fig. 3D). Notably, the hESC (d0) markers and regulators of stem cell proliferation, including POU5F (OCT4) and LIN28A, along with NSC (d8) markers SOX2, SOX3, and MSI, were grouped in Cluster 1, which had a gently decreasing pattern [27–29]. Nevertheless, CDH2, an NSC (d8) marker that is also known to be highly expressed in myelinating OLs [30, 31], like OPC (d80) and OL (d120) markers (PTPRZ, CD9, CNP, and GALC), was classified in Cluster 2 (with a slightly increasing trend). Likewise, OPC (d80) and OL (d120) specific proteins, i.e., CNTN1, SIRT2, NDRG1, ACTR1, and GSN, were located in Cluster 3 (with a sharp upward trend) [29, 32–35]. In general, these results revealed the expression distribution of enriched proteins with stage-specific biological functions within our 3 clusters, which may assist us to identify key proteome signatures associated with OL lineage differentiation. Therefore, this dynamic proteome outlook could support additional discovery of potentially competent proteins for *in vivo* OL differentiation of various neural precursors in patients with myelination defects. It also may provide us with stage-specific profiles that correlate with predominant biological functions associated with this differentiation process.

### Canonical and non-canonical Wnt signalling protein profiling during the generation of the OL lineage cells

Looking through enriched BPs of the clusters, we found the Wnt signalling pathway to be prominently affected (Supplementary Table S4). This pathway has been shown to be involved not only in OL development but also in other developmental processes [36]. We observed enrichment of the GOs related to the regulation of the Wnt signalling pathway, and non-canonical Wnt signalling pathways, particularly thet PCP pathway in both Clusters 1 and 2 (Fig. 3A and B, *left*, and Supplementary Table S4), which seems to have a complementary function in this context. Wnt signalling pathways, including Wnt/β-catenin (canonical) pathway, Wnt/Ca2^+^ (non-canonical) pathway, and PCP (non-canonical) pathway are fundamental mechanisms associated with various levels of vertebrate developmental procedures [37, 38].

**Figure 4:**
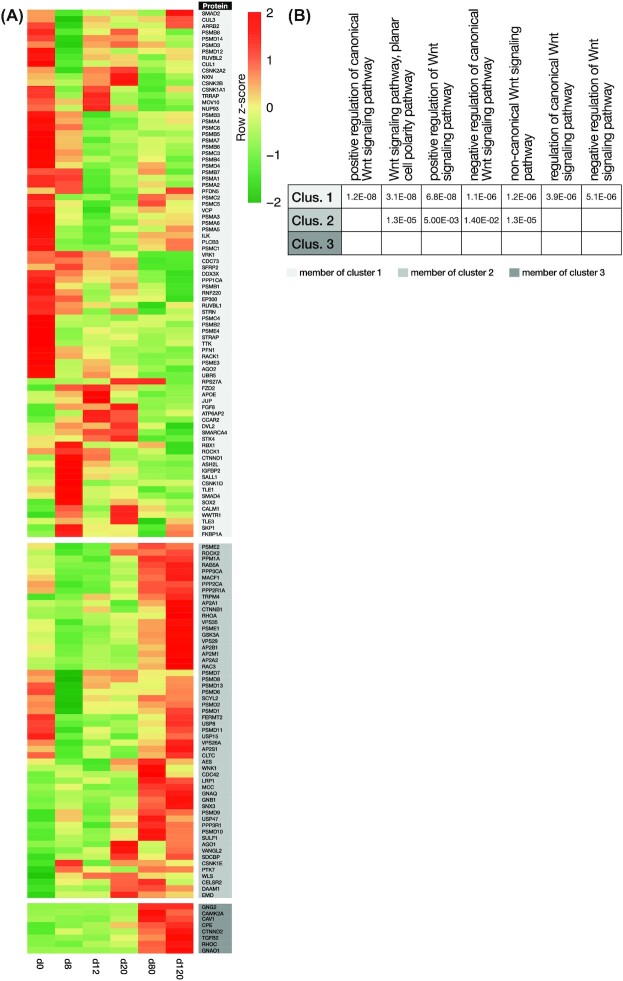
The dynamics of Wnt signalling–associated proteins during OL lineage differentiation. (A) Heat map shows the standardized relative expression changes of Wnt signalling–associated proteins along the OL lineage differentiation process. The colour of the protein name bar demonstrates the cluster status of proteins. Light gray, medium gray, and gray indicate whether the protein is a fit for Cluster 1, 2, or 3, respectively. Most expression changes occurred during the early and late stages. (B) The table shows the active Wnt signalling–related biological processes (BP) in OL lineage differentiation. The enrichment analysis of the proteins of each cluster indicates their roles with the Wnt signalling–related BPs. Their involvement scores are based on −log_10_ of the adjusted *P*-value. The blank cells show there either was no participation or the −log_10_ of the adjusted *P*-value was >0.05.

Systematic analysis of our data by applying Database for Annotation, Visualization and Integrated Discovery (DAVID 6.8) and UniProt (besides Enrichr, used in cluster functional enrichment analysis) revealed that 147 Wnt signalling–related proteins were enriched in the OL lineage differentiation process [39–42]. We found that 83 proteins from Cluster 1, 56 from Cluster 2, and 8 from Cluster 3 orchestrated Wnt signalling pathways during OL lineage differentiation (Fig. 4A). According to the heat map illustration of the relative abundances of Wnt signalling–related proteins, we noticed that the Wnt signalling pathways seemed to be highly active in this differentiation process distinctly at the hESCs (d0) and late (OPC and OL) stages (Fig. 4A). In support of our observations in cluster enrichment analysis, we noted that these proteins are mainly involved in GO related to the regulation of both canonical and non-canonical Wnt signalling pathways, especially the PCP pathway (Fig. 4B). To scrutinize the contribution of Wnt signalling components in detail, we also applied Gene Set Enrichment Analysis (GSEA) using whole protein expression profiles. Remarkably, Wnt signalling pathway was enriched especially at the 3 last time points of differentiation (i.e., d20, d80, and d120) compared to the other days (FDR *q*-value 0.029), indicating the importance of this pathway in OL differentiation (Supplementary Fig. S3). In general, this feature supported the results of previous studies that mentioned the crucial implication of canonical Wnt signalling in regulating stemness and development of ESCs; specification, proliferation, and differentiation of OPCs; and maturation and myelination of OLs [43–45]. Even though some observations made the effect of canonical Wnt signalling on these 3 cell types baffling, based on the ultimate outcome of all former studies, this cascade effect is amenable to the developmental stage, microenvironment, and intensity of the signalling [43, 46–50].

The enriched PCP pathway that regulates cell polarity is implicated in cellular morphogenesis, migration, intercalation, and function [51, 52]. This non-canonical Wnt signalling pathway has been shown to maintain the NSCs of the subventricular zone (located in the periventricular region) in the quiescence state. Chavali et al. demonstrated that a shift in PCP to canonical Wnt activity and keeping their balance induces NSC activation and lineage progression toward the generation of the progenitors that eventually go on to participate in the repair process [53]. It was also well demonstrated by Jarjour et al. that the PCP pathway is involved in the myelination initiation, and the structural organization of the axonal myelin sheath [54, 55]. Along with these reports, our finding shows that the high abundance of PCP pathway–related proteins in the middle stages of OL lineage differentiation (Fig. 4A and Supplementary Table S5) may convey the necessity of polarization in OPC specification, migration, and differentiation, which would need additional functional investigation because no study could be found that explored the PCP pathway in pre-OPC and OPC generation and their differentiation into OL. In addition, significant enrichment of PCP pathway–related proteins in hESCs possibly implies their role in modulating stem cell self-renewal [56].

Briefly, these findings represent non-canonical Wnt signalling pathways, especially the balance between PCP and Wnt/β-catenin pathway, as an enticing field of study in OL development. The results can potentially improve our knowledge about the implications of all of the Wnt signalling pathways in hESC maintenance and their further differentiation into the particular OL lineage; specification, migration, and differentiation of OPCs; and OL maturation and myelination, with the ultimate goal of recruiting pre-OPCs and OPCs to the demyelinated regions and achieving their OL differentiation, which may result in restoring myelin regeneration in diseases associated with myelin deficiencies.

### Macroautophagy-associated protein profile of the generation of OL lineage cells

Another set of remarkable BPs in our cluster enrichment analysis was related to autophagy (Supplementary Fig. S4A and Supplementary Table S6), an important lysosomal degradation and recycling pathway in mammalian cell development and differentiation [57]. Autophagy is a substantial issue in developmental processes. Because of the lack of a comprehensive study on the role of autophagy in OL lineage development or differentiation, we sought to peruse the proteome signature of the autophagy pathway through OL lineage differentiation of hESCs.

Autophagy is a highly conserved lysosomal-mediated cellular pathway responsible for catabolism plus recycling of damaged or outlived intracellular cargoes (macromolecules and organelles) to maintain cellular homeostasis and assist cellular structural remodelling during normal development and differentiation. The most common form of autophagy in eukaryotic cells is macroautophagy, which is mainly referred to as autophagy. In this major cellular degradation pathway, double-membrane vesicles (autophagosomes) engulf the cytoplasmic cargoes and digest them through the autophagosome-lysosome system [58, 59] (Supplementary Fig. S4B).

**Figure 5:**
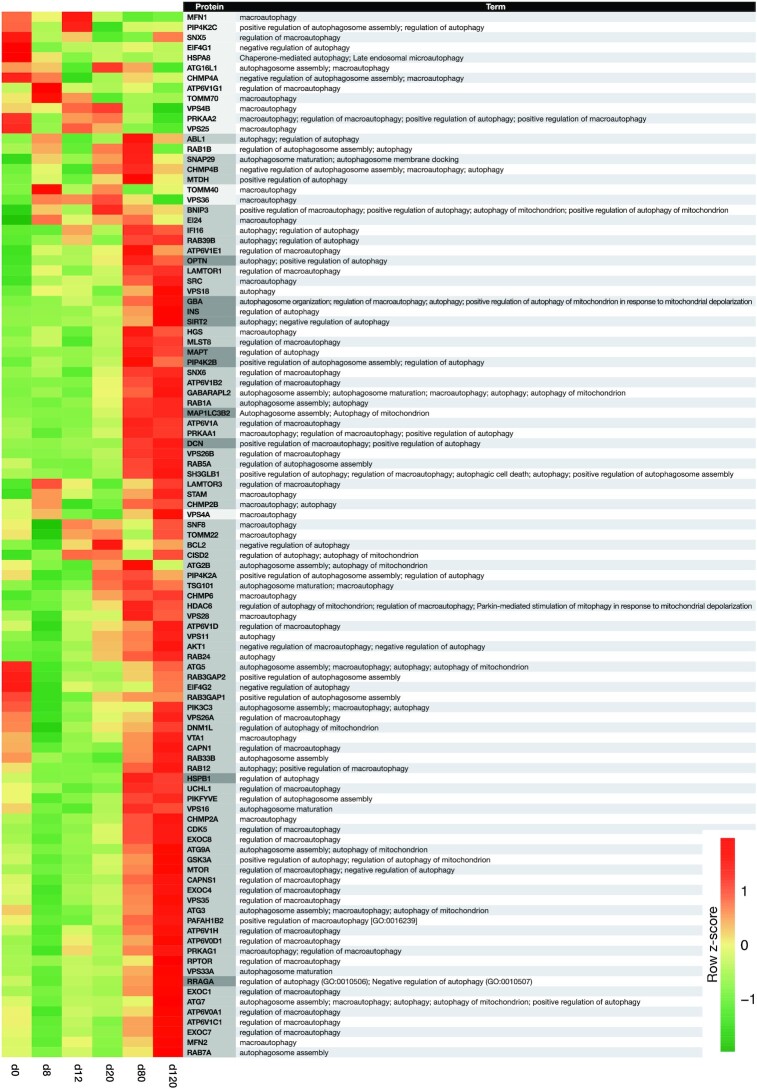
Expression profile of autophagy-associated proteins through oligodendrocyte (OL) lineage differentiation. Heat map shows the standardized relative expression changes of autophagy-associated proteins along the OL lineage differentiation process. The colour of the protein name bar demonstrates the cluster status of proteins. Light gray, medium gray, and gray indicate whether the protein is a fit for Cluster 1, 2, or 3. The autophagy-related biological processes (BPs) in which each protein is implicated are shown in front of it.

Further functional analysis of our proteome data using DAVID and UniProt [39, 40, 60] showed that the proteins involved in autophagy, particularly macroautophagy, were enriched through the OL lineage differentiation process. Our analysis highlighted the BPs engaged in autophagy, macroautophagy, autophagosome formation, and their regulation (Fig. 5). It featured the expressions of key upstream triggers of this pathway, adenosine monophosphate (AMP)-activated protein kinases PRKAA1, PRKAA2, and PRKAG1 [61], in addition to the proteins involved in the early stages of autophagosome formation, MAP1LC3B2 (a member of LC3s) and PI3KC3 [61]. Our data also showed enrichment of the autophagy-related (ATG) proteins (ATG16L1, ATG5, ATG7, ATG3, ATG2B, and ATG9A) that, in cooperation with LC3s (GABARAPL2 and PI3KC3), control major steps of autophagy, including autophagosome expansion, maturation, and lysosomal fusion, as well as cargo recruitment, degradation, and the recycling system [62] (Fig. 5 and Supplementary Fig. S4). A relative expression heat map of the 103 proteins involved in autophagy (found in our data) showed that 77 proteins were members of Cluster 2 and 10 proteins were members of Cluster 3. This may show the major influence of autophagy and macroautophagy in both specification and function of NPCs, pre-OPC, and OPCs, in addition to OL maturation and myelination (Fig. 5 and Supplementary Table S6). Our results corroborated previous findings that showed the crucial role of macroautophagy in OPC/OL differentiation, survival, maturation, and proper myelin development. In addition, these findings also brought up a possible vital role of this pathway in the early OL developmental stages [63, 64]. Furthermore, our data provided a novel proteome profile of autophagy-associated proteins through the OL lineage differentiation process. It revealed proteins related to each cell type (hESCs, NSCs, NPCs, pre-OPCs, OPCs, and OLs) and their expression trends in their generation process. Therefore, this may provide researchers with a tremendous repository for a better understanding of the molecular mechanism of autophagy in the OL lineage, and designing more effective therapeutic strategies toward demyelination disorders.

Owing to its cytoprotective role, autophagy is increasingly believed to promote neuronal and OL survival. However, in a disease like multiple sclerosis (the most well-known example of demyelinating diseases), therapeutic intervention of autophagy becomes complicated because multiple sclerosis is an inflammatory-mediated demyelinating disease, wherein cells of the immune system destroy the myelin sheaths of the nerve axons in the CNS and this is followed by neurodegeneration of both myelinating cells (OPCs and OLs) and neurons [65]. While studies show that pharmacological inducers of autophagy, such as rapamycin, can improve myelination of OLs and Schwann cells (the myelinating glia of the peripheral nervous system), the elevated levels of autophagy in immune cells of patients with multiple sclerosis make this type of medication hazardous [63, 66–69]. Nevertheless, our proteome data can be considered as a valuable source for finding an appropriate way to target a drug delivery system for these types of medications [70], particularly with the aim of directly inducing autophagy in OPCs and OLs. Therefore, the induced autophagy would promote these cells' function in clearing cellular and myelin debris, protein aggresomes, and their development toward achieving remyelination [64, 70, 71]. Currently, the pharmaceutical compounds that are used to induce autophagy may target signalling pathways other than autophagy [72]. One potential strategy to minimize adverse effects is to identify more specific autophagy regulators and mechanisms in every cell type to help to achieve targeted autophagy modulations [72, 73].

### Identification of potential biomarkers in each step of hESC differentiation into OLs

**Figure 6:**
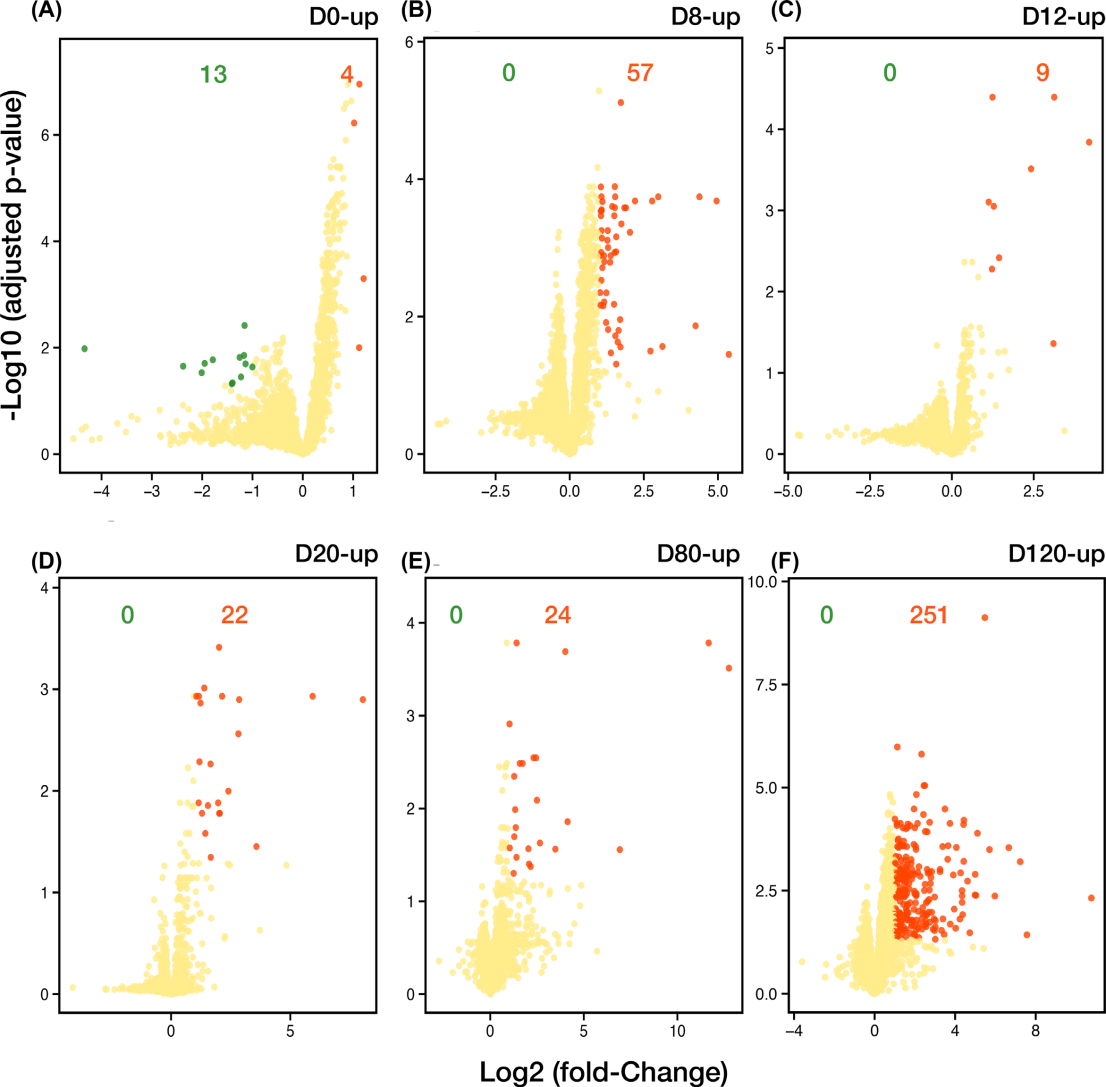
Stage-specific proteins of the oligodendrocyte (OL) lineage differentiation. (A–F) Volcano plots represent the comparison of protein expression at each time point with all other differentiation time points (or stages). The colour code separates differentially expressed proteins (DEPs) and similarly expressed proteins. The red dots indicate proteins that were highly upregulated at that specific stage (specified at the top right corner, above the plot) compared to all other stages while the green dots display the proteins with significantly low abundances at that stage, and the yellow dots indicate the proteins with consistent expression level, log_2_ (fold-change) >1, and −log_10_ (adjusted *P*-value) <0.05. The coloured numbers indicate how many dots of that colour appear in the plot.

In this study, we used TMT-based quantitative proteomics to discover important proteins involved in OL lineage differentiation. Furthermore, we exploited this approach to identify novel potential biomarkers that improve the selection, tracking, and monitoring of each specific cell type of OL lineage. Currently, the majority of the markers in the OL lineage are not specific to 1 cell type or 1 differentiation stage. Hence, the expression pattern of a panel of markers during the differentiation process is usually used, especially in *in vitro* studies [29]. For instance, LIN28A, a known marker of ESCs (Fig. 3D), is also a marker of stemness, which is highly expressed in NSCs [74–76]. This is also true for MSI1, a marker of NSPCs, which is also a well-known marker for adult stem cells and progenitors in hair follicles, mammary glands, and intestine [77–80]. MSI1 is also considered to be a prognostic biomarker in various human malignant neoplasms [81]. Thus, we investigated the identification and abundance of proteins that differentially expressed only in 1 of the differentiation stages. We observed significant (>2-fold) expression changes in the numbers of differentially expressed proteins (DEPs) in the hESC (d0, n = 4), NSC (d8, n = 57), NPC (d12, n = 9), pre-OPC (d20, n = 22), OPC (d80, n = 24), and OL (d120, n = 251) differentiation stages (Fig. 6, Supplementary Fig. S5 and Supplementary Table S7).

According to our DEP analysis, 4 proteins (HMOX1, MT1E, MT2A, and ASNS) showed their specificity at the hESC (d0) state (Fig. 6A and Supplementary Fig. S5), all of which are cytoprotective factors [82–84]. Among them, the specific role of HMOX1 (heme oxygenase-1) in the maintenance of self-renewal and pluripotency of ESCs and induced pluripotent stem cells (iPSCs) is well studied [85–87]. Even though the roles of MT1E and MT2A in ESCs have not yet been specifically studied, emerging evidence shows the remarkable expression of these metallothioneins in ESCs and iPSCs [88–90]. ASNS (asparagine synthetase), another deferentially upregulated protein in hESCs, is an ATP-dependent enzyme that synthesizes asparagine (Asn) and glutamate (Glu) at the expense of aspartate (Asp) and glutamine (Gln) [91]. Glu can be used in Gln synthesis by glutamine synthetase (GLUL), an enzyme that has been shown to be involved in cell proliferation, and demonstrated a considerable expression in all steps of OL lineage differentiation (data not shown) [92]. On the basis of previous studies, Gln and Asn act as principal survival and self-renewal factors in ESCs and cancer cells, respectively [91, 93, 94]. Ryu et al. have reported that Gln is an important factor in regulating maintenance of mouse ESCs through transcription regulation via the Akt, PKCe, and mTOR signalling pathways [95]; while Krall et al. have revealed that Asn is a powerful regulator of cell amino acid homeostasis; therefore it controls mTOR complex 1 (mTORC1) activation and cellular anabolic metabolism and proliferation [91]. Consequently, these data suggest that the 4 DEPs (HMOX1, MT1E, MT2A, and ASNS) can be efficient biomarkers for ESCs.

As mentioned above, we found 57 proteins that were specifically related to NSCs (Fig. 6B and Supplementary Fig. S5). Most of these proteins, like SOX2, CBX2, CBX5, HMGB2, RHF6, RBMS1, and SALL1 (all of which have already been reported as NSC-specific genes by Xiao et al. [96]), are involved in BPs related to chromatin organization and gene expression, reflecting the needed trigger for differentiation onset. Epigenetic modification of chromatin, in response to differentiation cues, controls gene expression in different cellular transitions, such as the differentiation of hESCs into NSCs [97]. The identified NSC state-specific proteins, like LUZP1 [98, 99], SOX2, PPT1 [100], SMOC1 [101], MAZ [102, 103], CRABP1 [104], GKAP1 [105], and CSRP2 [106], are also involved in BPs associated with cell division, proliferation, and differentiation of the nervous system. These findings demonstrate that NSC state-specific proteins (Fig. 6B and Supplementary Fig. S5) can be used as biomarkers of active NSCs.

As illustrated in Fig. 6C, there are only 9 proteins with specifically high abundances at the NPC stage (Fig. 6C and Supplementary Fig. S5). These proteins are mainly associated with cellular junction, adhesion, mitosis, and proliferation as well as cytoskeleton proteins [107]; however, apart from FREM2 [108, 109], the roles of the other proteins in NPC maintenance and function are not well documented [110]. According to previous studies, which highlighted the importance of cell-cell connections in NSPCs' biological behaviour [111–113], our data may bring the 3 differentially expressed desmosomal proteins, DSP, JUP, and PKP2, into the spotlight for further investigation of the impact of the desmosome junction on providing more desirable niches for NPC maintenance, proliferation, and differentiation. These findings may lead to the introduction of new potential biomarker proteins for the NPC state.

The 2 most highly differentially expressed proteins (mhDEPs, with log_2_ fold-change ≥ 5) on d20, MDK and RBP1, truly reflect the role of this leading step, the pre-OPC stage, in OL generation. Both mhDEPs instigate pre-OPC specification toward OL differentiation [114–117]. Among the 22 DEPs at the pre-OPC stage (Fig. 6D and Supplementary Fig. S5), there are 10 members of the histone family (H1F0, H1FX, H2AFX, H2AFZ, HIST1H1B, HIST1H1C, HIST1H1D, HIST1H2BM, HIST1H3A, and HIST2H2AB) that are involved in chromatin organization and show the dynamics of chromatin interaction through this differentiation state. This finding may indicate the significance of histone mark repatterning and remodelling of the chromatin architecture at the NPC stage (Supplementary Fig. S5). The correlation of this differential repatterning with the high abundance of known pre-OPC proteins such as MDK, RBP1 [114–117], SOX3 [118], and TNFRSF10B [119] points to the potential of these d20 DEPS to be used as biomarkers for the pre-OPC state.

Our findings also represent 24 DEPs at the OPC stage (Fig. 6E and Supplementary Fig. S5), 4 (FN1, TGFBI, TNC, and COL3A1) of which have been previously reported as DEPs of OPC by Chaerkady et al. [120]. FN1 (Fibronectin) is a glycoprotein of the ECM that stimulates OPC proliferation [121] and also has the capacity to impair OL differentiation and myelin sheath formation [122–124]. In OPCs, FN1 with PDGFA accompaniment leads to the actin-pERK1 and 2 co-localization and formation of filopodia, thus enhancing the migration of these cells [125]. The impact of the ECM protein TGFBI is not completely clear; however, it is known that TGFBI can regulate cellular adhesion and migration [126]. TNC, another ECM-glycoprotein, is produced by OPCs and preserves their proliferation. Downregulation of TNC is followed by OL maturation [127–129]. There is no information about the role of COL3A1 in the maintenance or function of OPCs; however, Gao et al. have reported that COL3A1 is a valid biomarker for diagnostic or therapeutic strategies for glioblastoma [130]. In fact, this protein may play a role in the maintenance of OPCs' stemness. Among the other noteworthy proteins at the OPC stage, there are 3 mhDEPs—GAP43, ATP2B2, and PCSK1N. GAP43 is a membrane protein in OPCs, and its expression decreases during OL differentiation [131]. ATP2B2, a magnesium-dependent enzyme that catalyzes the hydrolysis of ATP coupled with calcium transport, is a known constituent of the CNS myelin proteome [35] but its function in OPCs remains to be investigated. PCSK1N, a neuropeptide [132], is a proliferation-related molecule [133]. There is no evidence that directly focuses on the role of PCSK1N in OPC function or maintenance; however, it has been reported that, in the mouse insulinoma 6 (MIN6) cell line, Pax6 can directly downregulate *Pcsk1n*expression [134]. In addition, it is shown that, in the embryonic chick neural tube and spinal cord, Pax6 promotes OPC migration [135], the process that is usually followed by OL generation. Therefore, it can be hypothesized that PCSK1N may support the proliferation of OPCs. All in all, the company of the DEPs that are associated with cell proliferation and migration can indicate their utility as potential biomarkers at the OPC stage (d80).

Finally, we identified 251 DEPs on d120 (Fig. 6F and Supplementary Fig. S5), among which the transcripts of 40 proteins in the OL state have been previously reported: 4 transcripts by Hu et al. [136], 18 transcripts by Najm et al. [129], and 25 transcripts by Lager et al. [137]. Enrichment analysis of the 251 specifically upregulated proteins in the OL state by Enrichr [41, 42] showed that these proteins participate in OL developmental-related BPs such as ECM and cytoskeleton organization (e.g., BCAN, CD44, CDK5, FMNL2, HTRA1, ITGB4, PFN2, and TGFB2), nervous system development and OL differentiation (e.g., BCAN, CDK5, CNP, DPYSL2, FYN, MAPK1, and LSAMP), post-translational regulation of gene expression (e.g., CRYAB, INS, LUM, PACS2, RRAGA, SEPT3, and TGFB2), L-glutamate transport (e.g., ARL6IP5, PRAF2, and SLC1A3), and fatty acid metabolic processes (e.g., ABCD3, ACOX1, ACADS, BDH2, CRAT, CRYL1, and DECR1). According to neXtProt (v2.24.0), amongst these 251 DEPs, there are proteins associated with myelination such as TSPAN3, NDRG1, CA2, EPB41L3, CLU, GALC, ANXA2, GPC1, and AKT1 [107]. In addition, amongst the 40 DEPs that our study shares with the aforementioned works [129, 136, 137], there are 6 proteins with well-known functions in promoting OPC development into mature OLs: CNTN1 [33], SIRT2 [32, 138], TPPP [139], TGFB2 [140], CNP [141], and CDK5 [142]. These findings show the tremendous potential use of the identified DEPs as OL differentiation inducer, and potential biomarkers for diagnostic and prognostic purposes.

Therefore, our study, the first extensive step-by-step proteomic profiling of hESC differentiation into OL lineage, reveals several novel proteins that are potentially a part of this differentiation process and OL generation. These results propose a total of 378 potential biomarkers for every cell type of this lineage.

### Coordinated proteome dynamics of the stage transition–specific proteins throughout OL lineage differentiation

**Figure 7:**
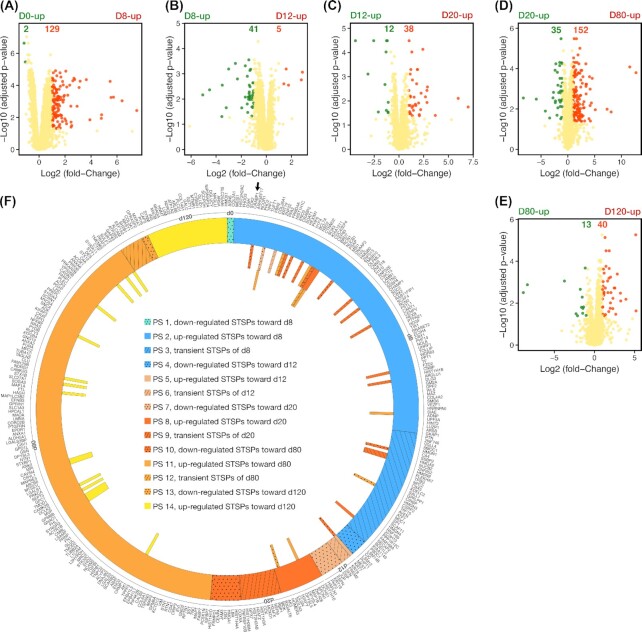
Dynamic proteome remodelling across the stepwise differentiation of hESCs into the oligodendrocyte (OL) lineage cells. (A–E) Volcano plot representations of the log-ratio of protein expression values in 2 different consecutive differentiation stages. The colour code separates differentially expressed proteins (stage transition–specific proteins [STSPs]) and similarly expressed proteins. The red dots demonstrate proteins that are highly upregulated at the achieved stage, while the green dots display the proteins with high abundances at its predecessor stage. The selection criteria of log_2_ fold-change >1 and −log_10_ (adjusted p-value) <0.05 indicate the differentially expressed stage transition proteins. (F) Generally, the STSPs can be classified in 14 protein sets (PS), illustrated as a colour-coded circular track, based on their differential expressions. The circular line between protein names and colour-coded track reveals the stages of the differentiation. As shown by the colour-coded bars attached to the circular track, 42 of the STSPs are specific to >1 stage transition event; e.g., CRABP1 (black arrow) abundance increases during d0 (human embryonic stem cell [hESC] stage) conversion into d8 (neural stem cell [NSC] stage), and its expression differentially downregulates through d20 (pre-oligodendrocyte progenitor cell [pre-OPC] stage) to d80 (OPC stage) transition.

Due to the time-series nature of our study, which enables a deeper insight into the development of every cell type in the OL lineage, subsequently, we sequentially compared pairwise stages towards their achievements by identifying stage transition–specific proteins (STSPs) (Fig. 7 ). Comparative pairwise analysis of differentiation stages showed distinct sets of STSPs (PS 1–14) that meticulously escort sequential steps of this differentiation process (Fig. 7F). At first glance, our results distinctly highlight the weighty role of transition from hESC (d0) to NSC (d8) and that of pre-OPC (d20) to OPC (d80) by upregulation of 129 and 152 proteins, respectively (Fig. 7A and D). A considerable transition is also displayed from NSC (d8) to NPC (d12), NPC (d12) to pre-OPC (d20), and OPC (d80) to OL (d120) by downregulation of 41 and upregulation of 38 and 40 proteins, respectively (Fig. 7B, C, and E).

Rolling down across the Waddington landscape toward NSC (d8) generation, hESCs (d0) go through the significant downregulation of HMOX1 and ASNS, 2 potential hESC (d0) biomarkers (discussed in the section Identification of potential biomarkers in every step of hESC differentiation into OLs), as well as upregulation of 129 proteins that may be divided into 2 protein sets (PSs; PS 2 and 3) (Fig. 7F). PS 2 (includes 93 upregulated proteins on d8) and PS 3, respectively, comprise 26 and 29 potential NSC biomarkers. As previously mentioned, on d8, we found 57 NSC stage–specific proteins, 55 of which also show their stage transition specification. The upregulated STSPs of the NSC (d8) stage also share 54 DEPoblast-derived NSC reported by Xiao et als. [96]. A number of them are known to be associated with NSC development and function such as SMOC1 [101], SOX2, CRABP1 [104], CSRP2 [106], CRABP2 [143], CTNND2 [144], SOX3, MSI1 [145], HMGB2 [146], and LAMB1 [147]. Moreover, among the 129 upregulated STSPs at the NSC (d8) stage, 36 STSPs (PS 3) show a transient trend as their abundances decrease while approaching the NPC (d12) state. In parallel to the 36 transient STSPs, there are 5 downregulated STSPs (PS 4), comprising PBDC1, LSM14B, CIC, LSM12, and PHPT1, in addition to 5 upregulated STSPs (PS 5 and 6) toward reaching the NPC (d12) state (Fig. 7F). The high abundance of STSPs at the NPC (d12) stage includes 2 well-known NPC proteins, MEST (NPC marker) [148, 149] and FERM2 (morphogenetic rearrangement protein of the ECM, which has a crucial role in neural tube closure) [108], in addition to 3 intermediate filament family members (KRT19, KRT7, and KRT8). The expression levels of these STSPs, which have also been represented here as the NPC stage–specific DEPs (Supplementary Fig. S5), show a differential reduction shortly after NPC achievement, with the exception of MEST, which first displayed a gentle decrease reaching the pre-OPC (d20) stage, followed by a dramatic reduction during the pre-OPC (d20) to OPC (d80) conversion. These findings, in addition to those reported by Najm et al. [129] , who monitored the overexpression of MEST in pluripotent mouse epiblast stem cell (EpiSC)-derived OPCs, indicate the significance of MEST in cell fate switching from pluripotent NSCs to pre-OPCs, as more specific OL progenitors.

Taking the principal step towards more committed cells of the OL lineage, 12 highly expressed proteins (STSPs at PS 6 and 7) in the NPC (d12) stage underwent differential downregulation while the expression levels of 38 STSPs (PS 8 and 9) increased, which promoted entrance of these cells into the pre-OPC (d20) stage (Fig. 7F). At this point, downregulation of NPC (d12) related STSPs such as FREM2 [108], DSP [150], TPP1 [100], KRT7 [151], KRT8 [152, 153], and KRT18 [154] was accompanied by upregulation of epigenetic factors and transcription regulators (e.g., MDK, HIST1H1C, HIST1H1E, H1FX, H2AFX, HMGA2, HIST1H3A, H1F0, SOX3, H2AFZ, HIST1H1B, H2AFY, HIST2H2AB, TAGLN3, and HIST1H2BM [107]) and some well-known pre-OPC STSPs (e.g., MDK, RBP1 [114–117], DCHS1 [155], SOX3 [118], MAP1B [156], DPYSL4, DPYSL5 [157, 158], TF [159], and TNFRSF10B [119]) (Fig. 7F). After succeeding the requisite regulation of the chromatin remodelling and gene expression, the abundance of the mediator STSPs, e.g., MDK, HMGA2, HIST1H1C, SOX3, H2AFX, H1FX, HIST1H3A, H2AFZ, HIST1H1B, HIST2H2AB, H1F0, and HIST1H2BM (the transition STSPs, settled in PS 9), along with MEIS1, HIST1H1D, CRABP1, HMGA1, and HIST1H4A (members of PS 10) [107], decreased distinctively (Fig. 7F), while pre-OPCs (d20) proceeded towards the specified OPCs (d80). Moreover, the fate specification of the cells into the OPC (d80) stage was assisted by the upregulation of 152 STSPs (PS 11 and 12) (Fig. 6F), which are involved in BPs such as cellular proliferation and its regulation (e.g., IGFBP7, FABP7, TNC, GNG2, CRIP2, CD47, and PRKCA), cellular differentiation and development (e.g., GAP43, MAPT, VIM, GPC1, GSN, SLC1A3, and SIRT2), and cellular migration and regulation (e.g., TSPAN3, L1CAM, FN1, NTN1, LMNA, RUFY3, and SULF1) [107]. Amid these upregulated proteins (PS 11) there was a subset of STSPs (TPPP3, FHL1, GNAO1, GPM6B, DCLK2, TMOD1, CRYL1, CAVIN4, FTL, SLC27A1, CAMK2G, TSPO, PLEC, DES, TMEM65, and SIRT2) that underwent an additional significant upregulation during OPC (d80) conversion into OL (d120) stage (Fig. 7F). These STSPs, which were shared between PS 11 and PS 14, participate in cytoskeleton organization (e.g., TPPP3, GPM6B, TMOD1, DCLK2, DES, and PLEC [107]), a pivotal BP in OPC generation, differentiation, and migration in conjunction with OL generation and myelination. These STSPs are also involved in other OPC and OL BPs that are necessary, such as regulation of cell proliferation and cell cycle (FHL1, SIRT2, and TSPO), cell differentiation (FHL1, CAMK2G, and CAVIN4), nervous system development (CAMK2G, GPM6B, DCLK2, SIRT2, and GNAO1), and in BPs that are implicated in the production of proteins (TPPP3, GPM6B, DCLK2, SLC27A1, SIRT2, TSPO, and GNAO1) and lipids (CRYL1, GPM6B, DCLK2, SLC27A1, SIRT2, and TSPO), which are 2 main components of myelin [107]. We found that OPC (d80) differentiation was accompanied by selective downregulation of the 13 STSPs, 9 of which showed transient differential expressions at this stage (Fig. 7F). Among the transient STSPs, GAP43 and ATP2B2 were the top 2 most highly differentially expressed STSPs at the OPC (d80) stage, with log_2_ fold-change of 12.73 and 11.57, respectively. GAP43 is an OPC protein, and its expression has been shown to decrease and reach a plateau during OPCs' differentiation into OLs [160, 161]. Although GAP43 is a calmodulin (CaM)-binding protein, like ATP2B2, GAP43 phosphorylation reduces its affinity for CaM. Hence, the interaction between GAP43 and CaM controls the availability of CaM [162], which then regulates the calcium pump activity of ATP2B2, and thus GAP43 takes part in the regulation of OPC maintenance and differentiation [163–165]. PS 14 is another subset of STSPs that promotes this prolonged differentiation process into its final OL target. PS 14 has 40 members, 16 of which are shared with PS 11 (as mentioned above) (Fig. 7F). All 40 STSP members of PS 14 are part of the aforementioned OL stage–specific proteins. For instance, the merits of INS [166, 167], RRAGA [168], GALC, GPM6B [169], PLEC [170], and SIRT2 [32, 138] for the OL (d120) stage have been already discussed.

Consequently, these results demonstrate the outstanding potential of STSPs to improve the status of OL lineage differentiation at each of the different stages and shed light on future mechanism-based developments of strategies for the treatment of demyelinating disorders.

## Methods

### hESC differentiation into OL lineage cells

Adherent confluent RH6 cells (passages 45, 48, and 50, to accommodate the 3 biological replicates) were induced into SOX1^+^ NSCs by dual inhibition of SMAD signalling [171, 172]. Under the treatment of 10 µM SB431542 (SB, inhibitor of TGF-β/activin/nodal signalling), 250 nM LDN193189 (LDN, inhibitor of bone morphogenetic protein signalling), and 100 nM all trans-retinoic acid (RA, caudalizing patterning agent) for 8 days, nearly all the differentiated cells were SOX1^+^ NSCs (Supplementary Fig. S1A and C). Next, to mimic the embryonic ventral spinal cord environment (pMN domain) and achieve pre-OPC, 100 nM RA and 1 µM smoothened agonist of sonic hedgehog (SAG, ventralizing patterning agent) were applied for 22 days. By day 12 of differentiation, NSCs gave rise to OLIG2^+^ NPCs (Supplementary Fig. S1D), which were then detached for sphere aggregation; this enriched the OLIG2^+^ population. By day 20 of differentiation, the OLIG2^+^ progenitors committed to the OL lineage by co-expressing NKX2.2 (pre-OPCs; Supplementary Fig. S1E). On day 20, supportive reagents for pre-OPCs' expansion and maturation toward OPCs, and further OPC expansion and maturation toward OL-producing OPCs, i.e., 10 ng/mL platelet-derived growth factor AA (PDGF-AA), 5 ng/mL hepatocyte growth factor, 10 ng/mL insulin-like growth factor 1 (IGF1), 10 ng/mL neurotrophin 3 (NTF3), 60 ng/mL 3,3,5-triiodo-l-thyronine (T3 [thyroid hormone]), 25 µg/mL insulin, 100ng/mL biotin,  and 1 µM cyclic adenosine monophosphate (cAMP), were added to the culture medium for 60 days (Supplementary Fig. S1A). On day 30, spherical aggregates were plated onto poly-L-ornithine/laminin-coated (pO/L) dishes and on day 80 PDGF^+^ OPCs were generated (Supplementary Fig. S1F). To eliminate the neurons and astrocytes that migrated out of the spherical aggregates and to achieve a homogenous population of OPCs, we replated the cells twice onto pO/L dishes, on days 65 and 75 of differentiation. On day 80, the growth factors were withdrawn from the culture medium and OPCs were differentiated into MBP^+^ OLs in the presence of 10 mM 4-(2-hydroxyethyl)-1-piperazineethanesulfonic acid (HEPES) buffer and 20 µg/mL ascorbic acid (supportive for OL differentiation), in addition to T3, insulin, biotin, and cAMP (Supplementary Figs S1A and G and S6 and Supplementary Table S9) [6, 7, 173].

### Immunostaining

Cells were washed 3 times in phosphate-buffered saline (PBS^−^) (Life Technologies, Cat. No. 10010023) for 3 min and then fixed with 4% (w/v) paraformaldehyde for 15 min at room temperature (RT). Following fixation, the cells were washed 3 times in 0.1% PBS^−^ Tween (PBS^−^ that contained 0.1% Tween 20) for 3 min and stored at 4°C. At the time of staining, cellular membranes were permeabilized by 0.5% PBS^−^ Triton (PBS^−^ that contained 0.5% Triton X-100) for 1 hour at RT. Then, the cells were incubated in blocking solution that consisted of 0.1% PBS^−^ Triton, 0.2% donkey serum, and 0.2% bovine serum albumin (BSA) for 1 hour at 37°C. After washing 3 times with washing solution (0.1% PBS^−^ Tween), the primary antibodies were applied overnight at 4°C (for antibody information, refer to Supplementary Table S10). The next day, these cells were washed 3 times in a washing solution for 10 min and stained with a secondary antibody for 45 min at 37°C (for antibody information, refer to Supplementary Table S10). Thereafter, the cells were washed 3 times with washing solution for 10 min, counterstained with DAPI at RT, and washed in washing solution. Images were captured using an Olympus IX71 inverted fluorescent microscope equipped with an Olympus DP72 Digital Color Microscope Camera.

### Protein isolation

Total protein extraction was performed using TRIzol^TM^ reagent. Cells were washed with PBS, detached mechanically, pelleted by centrifugation, and snap frozen in liquid nitrogen before they were stored at −80°C. For each differentiation stage, we pulled 3 plates of cultured cells. At the time of isolation, the cell pellets were lysed and homogenized with TRIzol^TM^ reagent (Invitrogen, USA, Catalog No. 15596018) according to the manufacturer's instructions. Then, chloroform (Merck Millipore, USA, Catalog No. 102444) was used for phase separation. The samples were centrifuged for 15 min at 12,000*g* at 4°C and the clear upper aqueous phase was used for RNA isolation, the white interphase was discarded, and protein extraction proceeded by the red organic phase. Cold (−20°C) 100% ethanol (Merck Millipore, USA, Catalog No. 107017) was applied to dissociate DNA from proteins trapped within the lower red phenol-chloroform phase. Thereafter, the dissolved proteins were precipitated by an overnight incubation in acetone (Merck Millipore, USA, Catalog No. 100014) at −20°C. The following day, the total proteins of each sample were precipitated via centrifugation and the resultant pellets were washed with washing solution, first one that contained 1 mL 0.3 M guanidine hydrochloride (Sigma-Aldrich, USA, Catalog No. G3272) in 95% ethanol and 2.5% glycerol (Sigma-Aldrich, USA, Catalog No. G5516), and then a washing solution composed of 100% cold ethanol that contained 2.5% glycerol. The resultant pure protein pellets were air-dried and solubilized in lysis buffer that contained 7 M urea (Sigma-Aldrich, USA, Catalog No. U5378), 2 M thiourea (Sigma-Aldrich, USA, Catalog No. T8656), 4% CHAPS detergent (Sigma-Aldrich, USA, Catalog No. C3023), 50 mM dithiothreitol (Sigma-Aldrich, USA, Catalog No. D0632), and protease and phosphatase inhibitor cocktails (both from Sigma-Aldrich, USA, Catalog No. P2714 & P5726). In the end, the protein samples were stored at −70°C.

### Protein preparation

The isolated protein samples were thawed at 4°C. The concentrations of the total proteins were determined by a spectrophotometer using a modified Bradford dye-binding method [174] and BSA as the standard. A total of 300 μg of each protein sample was reduced with 5 mM dithiothreitol (Sigma-Aldrich, Cat. No. D0632) for 30 min at 56°C. Thereafter, an alkylation agent, iodoacetamide (Sigma-Aldrich, Cat. No. I6125), was mixed with the reduced proteins at a concentration of 14 mM and samples were left at RT for 30 min in the dark. The process was followed by another reduction procedure. This time, after mixing 5 mM dithiothreitol with alkylated protein samples, the mixtures were kept at RT for 30 min. Subsequently, for protein shipment, protein samples were stored at −70°C for 24 h. After 24 h, the protein samples were lyophilized for 48 h at ∼−50°C. Next, the lyophilized proteins were shipped in a pack filled with silica gel beads to prevent any potential dampness.

To remove the interfering detergents and contaminants, the proteins were precipitated using the methanol-chloroform protocol [175]. After sequential addition of ice-cold methanol, chloroform, and water with vortexing intervals, the samples were centrifuged for 2 min at 1,000*g* at 4°C. The protein aggregate at the interface layer was washed with ice-cold methanol and acetone. Next, the protein pellet was air-dried and then resuspended in 200 μL of 8 M urea in 50 mM Tris (pH 8.8). Eventually, the protein concentration was determined by a Bicinchoninic Acid (BCA) Assay Kit (Pierce, Rockford, IL, USA) using BSA as the standard.

In the end, dual digestion was conducted on 150 μg of each protein sample, first with Lys-C (Wako, Japan) at a 1:100 enzyme: protein ratio overnight at RT, followed by trypsin (Promega, Madison, WI, USA) digestion at a 1:100 enzyme: protein ratio for ≥4 h at 37°C. The reactions were stopped using trifluoroacetic acid to a final concentration of 1% (pH 2–3). Peptide yields were desalted by SDB-RPS (3M-Empore) Stage Tips and the eluted peptides were dried by vacuum centrifuge, then the dried peptides were reconstituted in 200 μL of 200 mM HEPES (pH 8). Next, the peptide concentration was measured using the Micro BCA™ Protein Assay Kit (Thermo Scientific, Rockford, IL, USA) and 70 μg from each sample was aliquoted for labelling in a 10-plex TMT reaction [176].

### TMT labelling

To accommodate 4 biological replicates from 7 different sampling points (0, 8, 12, 20, 50, 80, and 120 days) we performed 3 interdependent TMT experiments. Each TMT experiment contained the same technical replicate (d0) as a common reference and ≥1 biological replicate per sampling point as illustrated in Fig. 1. The remaining 2 empty channels in each TMT experiment were assigned to the fourth biological replicate per sampling points. We have made the experimental design clear by providing a table containing the exact details of the labels and sample identifications (Fig. 1). However, in our preliminary PCA analysis, we noticed inconsistency with our fourth replicate (unknown reason); therefore, we decided to present the most correlated triplicates for the further analysis. Furthermore, day 50 (early-OPC stage) was not further considered in the analysis owing to its remarkable similarity to day 80 (OPC stage) in our preliminary Pearson correlation coefficient analysis (explained in “TMT data analysis” section).

The TMT labelling was carried out as previously described [176–178]; we added 41 μL of anhydrous acetonitrile to each 0.8-mg label vial, followed by occasional vortexing for 5 min and brief centrifugation. Ten TMT labels (Thermo, San Jose, CA, USA) were added to the 10 individual protein samples in each experiment. Labelling was performed for 1 h at RT with occasional vortexing. To quench any remaining TMT reagent and reverse the tyrosine labelling, 8 μL of 5% hydroxylamine (Sigma-Aldrich) was added to each tube, followed by vortexing and incubation at RT for 15 min. For each of the respective 10-plex experiments, all 10 labelled samples were combined in a clean 2 mL Eppendorf tube and then dried via speed vacuum centrifugation. The dried peptide mixture was reconstituted in 1% formic acid (FA, pH ∼2–3) and desalted on a 130-mg C18 Sep-Pak (Waters, Milford, MA, USA) as previously described [179], and eventually dried down again using a speed vacuum centrifuge.

### Fractionation

To reduce the complexity of the mixture, offline strong cation exchange fractionation was carried out for each of the TMT experiments. The labelled peptides were resuspended in strong cation exchange buffer A (5 mM KH_2_PO_4_ and 25% v/v acetonitrile [ACN], pH 2.72) and were injected onto a PolySULFOETHYL A™ column (200 mm × 2.1 mm, 5 μm, 200 Å; PolyLC, Columbia, MD, USA), which was also equilibrated with buffer A. The adsorbed peptides were eluted with a linear gradient of 10–45% buffer B (5 mM KH_2_PO_4_, pH 2.72, 350 mM KCl, 25% ACN) for 70 min, which was then rapidly increased to 100% buffer B for 10 min at a flow rate of 300 μL/min. The collected samples were then desalted using SDB-RPS Stage Tips, dried by vacuum centrifuge, and reconstituted in 40 μL of 0.1% FA in preparation for nanoLC-ESI-MS/MS as reported by Mirzaei et al. [176].

### NanoLC-ESI-MS/MS of TMT-labelled peptides

Fractionated peptide samples were analysed on a Q Exactive Orbitrap mass spectrometer (Thermo Scientific, San Jose, CA, USA) coupled to an EASY-nLC1000 nanoflow HPLC system (Thermo Scientific, San Jose, CA, USA) as described previously [179]. The peptides were separated on an in-house–packed reverse-phase column (75 μm inner diameter × 100 mm, C18 HALO column, 2.7 μm bead size, 160 Å pore size, Advanced Materials Technology). For sample elution, fractionated labelled peptides were run for >170 min on a linear gradient of 1–30% solvent B (99.9% ACN/0.1% FA). The Q Exactive mass spectrometer was operated in the data-dependent acquisition (DDA) mode to automatically switch between full Orbitrap MS and ion trap MS/MS acquisition. Survey full-scan MS spectra (from *m*/*z* 350–1850) were received at a precursor isolation width of 0.7*m*/*z*, resolution of 70,000 at *m*/*z* 400, and an automatic gain control target value of 1 × 10^6^ ions. For identification of the TMT-labelled peptides, the 10 most abundant ions were selected for higher energy collisional dissociation (HCD) fragmentation. HCD normalized collision energy was set to 35% and fragmentation ions were detected in the Orbitrap at a resolution of 70,000. Dynamic exclusion of target ions (selected for MS/MS) was set to 90 s and for accurate mass measurement, the lock mass option was enabled using the polydimethylcyclosiloxane ion (*m*/*z* 445.12003) as the internal calibrant [180].

### Data processing and protein identification

MS raw data were generated by Xcalibur software (Thermo Scientific) and processed with Proteome Discoverer V1.3 (Thermo Scientific, San Jose, CA, USA). Peptide identification was performed using a local MASCOT server V2.3 (Matrix Science, London, UK). The MS/MS spectra were searched against the reviewed UniProt *Homo sapiens* protein database (20,352 sequences—August 2018) [10]. The following parameters and adjustments were used: MS^1^ tolerance was set to ±10 ppm precursor. A limit of 0.1 Da was applied for MS/MS fragment ion tolerance and trypsin was set as cleavage specificity that allowed only 1 missed cleavage. Carbamidomethylation of cysteine was set as a fixed modification and TMT modification of peptide N-termini plus lysine residues, oxidation of methionine, and deamidation of Asn and Gln residues were set as variable modifications. For deconvolution of the high-resolution MS^2^ spectra, only peptides with a score >15 and below the Mascot significance threshold filter of *P* = 0.05 were included in the search results. Single peptide identifications required a score equal to or above the Mascot identity threshold to be incorporated in the search results. Protein grouping was enabled such that when a set of peptides in 1 protein were equal to or completely contained within the set of peptides of another protein, the 2 proteins were confined in 1 protein group. Proteins with ≥2 unique peptides were regarded as confident identifications. Hence, search results were further filtered to retain proteins that had *q*-values (FDR) of <1% and only master proteins assigned via the protein-grouping algorithm were retained.

The mass spectrometry proteomics raw data files, database search results, and TMT ratios can be retrieved via the ProteomeXchange Consortium [11] through the PRIDE partner repository with the dataset identifier PXD017649. In each TMT experiment, relative quantitation of proteins was achieved by pairwise comparison of TMT reporter ion signal to noise (S/N) ratios as the ratio S/N of the labels for each of the differentiation time points vs the labels of the internal control (labelled with TMT 126 reagent) of the corresponding run (Supplementary Table S2).

### TMT data analysis

Relative quantitation of all protein abundances, in every differentiation time point, with respect to the reference (labelled with TMT 126 reagent), was extracted from the 3 TMT experiments and aggregated into a single report (Supplementary Table S2). Overall data quality was first checked by the analysis of variance (ANOVA) adjusted *P* ≤ 0.05 (Supplementary Table S2). Further data analyses and visualization were generally performed with homemade programs developed in the R statistical computing environment [181]. A proportional Venn diagram comparing the depth of protein coverage (identified proteins) in 3 TMT mass spectrometry experiments (Supplementary Fig. S2A) was created by the “venn.diagram" function from the VennDiagram package [182]. For each time point, the pairwise correlation of the replicates was computed by the Pearson correlation coefficient method through the “cor" function; then in order to incorporate the highest-correlated replicates into our study, we decided to present the most correlated triplicates for further analysis (Supplementary Table S2). The proteins that were only quantified in some time points of 1 TMT experiment, the proteins that were quantified in only 1 TMT experiment, and the proteins that were quantified in 2 TMT experiments but not in all time points were excluded from the study. Then the missForest function from R package missForest [12, 183] was applied for the imputation of those identified proteins that were quantified in all time points of 2 TMT mass spectrometry experiments while their relative expressions were not quantified in some or all time points of the other TMT experiment. As a result of this supervised approach, the missing expression of 336 proteins was imputed and the “Quantified Proteins” table in Supplementary Table S3 was created. To illustrate the diversity of the quantified proteins (Supplementary Table S3), protein classification in Supplementary Figure S2B was performed using the PANTHER classification system ([184]; Supplementary Table S11) [13]. To visualize the correlation of the analysed samples, in R, the “cor" function with the “pearson" method argument was applied (on “Quantified Proteins” table in Supplementary Table S3) for Pearson correlation coefficient analysis, and the “pheatmap" function from the pheatmap package [185] was used to illustrate the results in Fig. 2A. The clustering_distance_rows argument in the pheatmap function was set as “correlation" (at this point we noticed the remarkable similarity between samples of day 50 and day 80; thus because day 80 contained more mature OPCs, we decided to exclude the data of day 50 from the present study). With the aim of finding the best summary of our dataset (Supplementary Table S3), we performed PCA by first scaling the expression values in R, using the “scale" function and then applying the “prcomp" function. In this regard, we used the “rotation" matrix as a PCA loading matrix and visualized the linear combination of PC1 and PC2 by means of the ggplot function from the R package ggplot2 (Fig. 2B and Supplementary Table S3). The Biological DataBase network (bioDBnet) was used for conversion of accession IDs into HGNC Symbols (protein names) [186, 187]. The averages of the relative protein expression of all quantified proteins, in 3 replicates, were calculated by the “mean" function and the following analyses were performed on the resulting dataset (Supplementary Table S3) [181]. To cluster the data and investigate co-regulated proteins associated with similar BPs, a c-means unsupervised fuzzy clustering algorithm was performed using the cmeans function of the package e1071. A maximum of 100 iterations were considered while the degree of fuzzification was set to 2. The c-membership values denote the similarity of the data points to each of the cluster centers [188] (Fig. 3A–C and Supplementary Table S5). Then, the protein members of each cluster were subjected to Gene Ontology (GO) analysis using Enrichr [189] and an adjusted *P*-value cut-off of <0.05 was used to filter the results [41, 42] (Fig. 3A–C and Supplementary Table S6). For the visualization, some of the overrepresented BPs of each cluster were selected and presented with bar plots showing the −log_10_ of the adjusted *P*-values of significantly enriched BPs (Fig. 3A–C). Heat map illustrations of the relative protein expression changes of the marker proteins in Fig. 3D were created by the pheatmap function from the pheatmap package, while the “scale" and “clustering_distance_rows" arguments were set at “row" and “correlation," respectively [185]. Noticing the participation of some OL lineage proteins in Wnt signalling and autophagy pathways (Supplementary Table S6), next we investigated single-protein indexed GOs by the UniProt (based on reviewed indexed data) database [40,190] and confirmed the result by the neXtProt [107, 191] and DAVID gene annotation tool v6.8 [39, 192] databases. Then, we filtered the proteins involved in these 2 pathways and put them in 2 data tables (Supplementary Table S5 [contains Wnt signalling–associated proteins] and Fig. 5 [autophagy-associated proteins]). The expression profile of these 2 protein sets (Supplementary Tables S5 and S6) was illustrated by heat maps, using the pheatmap function. For visualization of the Wnt signalling–associated proteins’ expression, we set the “scale" arguments at “row," then we cut the heat map into 3 pieces each of which reveals the contribution of 1 cluster (Fig. 4A). And for the autophagy-associated proteins in Fig. 5, in the pheatmap function, we set the “scale" and “clustering_distance_rows" arguments at “row" and “correlation," respectively. GO enrichment analysis of the Wnt signalling–associated proteins were performed by Enrichr and an adjusted *P*-value cut-off of <0.05 was used to filter the results (Fig. 4B) [41, 42]. We have also performed GSEA using GSEA 4.0.3 [193] with weighted enrichment statistics. The number of permutations was set to 1,000. We used gene_set as the permutation type and Signal2Noise metric for ranking genes. The minimum and maximum sizes of the sets were adjusted to 15 and 500, respectively. Wnt signalling components for GSEA analysis were obtained from the Molecular Signatures Database (MSigDB) [194] (Supplementary Fig. S3). To find specific proteins of each differentiation stage that were differentially expressed in only 1 time point in comparison with all other time points (Supplementary Table S3), we conducted a differential expression analysis by applying the R/Bioconductor package limma version 3.34.9 [195]. Moreover, owing to the time-series nature of our study, we analysed the differential expression of each protein (Supplementary Table S3) between 2 sequential time points by applying the R/Bioconductor package limma [195], using the lmFit, makeContrasts, contrasts.fit, ebayes, and toptable functions from limma in both differential expression analysis procedures [195]. Proteins having absolute log_2_ fold-change > 1 and Benjamini-Hochberg adjusted *P* < 0.05 were considered to be significant DEPs (Supplementary Table S7 and S8). The DEP analyses were illustrated by volcano plots (Fig. 6A–F and 7A–E). The stage-specific proteins were also demonstrated in a heat map (Supplementary Fig. S5) using the pheatmap function with the “scale" argument set at “row," and “cluster_cols" and “cluster_rows" arguments set at “FALSE." Finally, for the illustration of the STSPs in Fig. 7F, we used a built-in doughnut chart type in Excel; however, the circular visualization of the protein members of each slice was performed by setting the circos.par, circos.initialize, circos.track, and circos.trackText functions of the package circlize version 0.4.8 [196].

## Availability of Supporting Data and Materials

The mass spectrometry proteomics raw data have been deposited in the ProteomeXchange Consortium through the PRIDE partner repository [11] with the dataset identifier PXD017649. Other data further supporting this work are openly available in the *GigaScience* respository, GigaDB [197].

## Additional Files

**Fig. S1:** OL lineage cell generation. (A) Schematic representative of OL differentiation protocol (for materials information see Methods and Supplementary Table S9). First NANOG^+^ hESCs (B) were induced to NSCs by dual SMAD inhibition. Then, neural progenitor patterning and OPC commitment were achieved by the application of 2 morphogens, RA and SAG. Subsequently, PDGF medium was used to promote OPC formation. From day 80 onward, Glial medium was utilized for OL derivation. SOX1^+^ NSCs were detected on day 8 (C) and OLIG2^+^ NPCs appeared on day 12 (D) and participated in aggregate formation after being detached. Consequently, NKX2.2^+^ pre-OPCs (E), day 20, differentiated into PDGFRA^+^ OPCs on day 80 (F); their further differentiation resulted in MBP^+^ OLs (G) on day 120. The attained OLs demonstrate a typical OL morphology that consisted of a round, central soma with multiple branching processes that expanded symmetrically outward and gave the OL a spider-in-a-web–like appearance [198]. hESC: human embryonic stem cell; NPC: neural progenitor cell; NSC: neural stem cell; OL: oligodendrocyte; OPC: oligodendrocyte progenitor cell; RH6: Royan H6 cell line; scale bars: 50 µm.

**Fig. S2:** Overall validation of the collected proteome data. (A) Proportional Venn diagram compares the depth of protein coverage in 3 replicates. A total of 3,527 proteins were identified in the 3 TMT mixtures that were analysed, while 1,056 proteins were only identified in 2 replicates. (B) The doughnut chart represents the diversity of the quantified proteins based on PANTHER protein class annotation.

**Fig. S3:** Enrichment plot of Wnt signalling components (from GSEA data set) between the last 3 time points (d20, d80, and d120) compared to the others using MSigDB set for Wnt signalling components (FDR *q*-value 0.029).

**Fig. S4:** The contribution of macroautophagy in generation of the OL lineage. (A) Cluster enrichment analysis (see Supplementary Table S6) featured the prominent participation of macroautophagy and autophagy pathways in OL lineage differentiation of hESCs. “in C2 and C3” reveal the clusters in which these GO terms were enriched. (B) Schematic illustration of the process and main regulatory machinery of macroautophagy. AMP-activated kinase (AMPK) signalling is depicted as the activator of the macroautophagy process (initiation) that targets the ULK1 (Unc-51-like kinase 1) initiation complex. The initiation complex then triggers membrane nucleation and phagophore formation. Hence, the cup-shaped double membrane phagophore begins to engulf the autophagic cargo and expands into the double-membrane vesicle (autophagosome) that sequesters the cargoes completely (phagophore expansion). Subsequently, the autophagosome fuses with acidic lysosomes (fusion with the lysosome) and forms autolysosomes, where the cargo will be degraded (degradation). The coloured ovals encompass the proteins quantified in our study of OL lineage differentiation. This figure is adapted from Hansen et al. [60].

**Fig. S5:** Illustration of the abundances of differentially expressed proteins (DEPs) at each stage of oligodendrocyte (OL) lineage differentiation. Heat map shows the standardized relative protein expression changes of DEPs at each step of OL lineage differentiation. The 7 expression profile clusters (left colour-coded bar) describe stage-specific patterns of the dynamics of 378 DEPs. The clusters are indicated by different colours, each of which demonstrates 1 specific differentiation step.

**Fig. S6:** Supporting figure for Supplementary Fig. S2. (A) Cells at the NSC stage show an almost uniform expression of CDH2. (B, C) Immunofluorescent staining of the control NSC line, RSCB0181, by CDH2 and SOX1 antibodies. (D–L) Nearly 100% of the generated cells at OPC stage (d80) expressed PDGFRA, and 97% of them were also SOX10^+^. (M) Immunofluorescent staining of the OLIG2^+^ NPCs at the d12 stage with an antibody from a different provider shows the same result. (N, O) A total of 90% of the cells at pre-OPC stage express NKX2.2. We counted 5 different fields of these 2 figures as well as Supplementary Fig. S1E. (P–W) A total of 22% of the cells at the OL stage were mature oligodendrocyte. (X–Z) Phase contrast photos of the cells at the OL stage.

**Table S1:** The neXtProt entry and the full name of the proteins mentioned in the text of this article.

**Table S2:** Protein identification, TMT reporter ion ratios, protein quantitation, and study design. The tables numbered 171102, 171108, and 171105 represent the data acquired by the first, second, and third TMT experiments. The “Aggregated Data" table includes all of the identified proteins of the 3 replicates in 1 table. The “Study Design" table shows the TMT experimental design and the “Replicates Correlation" table illustrates the Pearson correlation coefficients of the replicates. The analysis of variance (ANOVA, in the Aggregated Data table) represents 3,132 proteins (∼81% of all of the identified proteins) that showed significant changes (adjusted *P* ≤ 0.05) through the OL lineage differentiation of hESCs. On the basis of the Pearson correlation coefficient analysis, “d0_r1" was left out of the study. In addition, we decided to present the most correlated triplicates for further analysis.

**Table S3:** The first table (Quantified Proteins) shows the relative expression of all of the identified and quantified proteins in every biological replicate of each time point during OL lineage differentiation. The second table (Variable Loadings Matrix) includes the details of the PCA of the proteome profile of each differentiation stage in Fig. 2B. The third table (Contribution of PCs) reveals the contribution of computed PCs, among which we chose PC1 and PC2 for the illustration of the PCA analysis in Fig. 2B. The fourth table (Averages of the Replicates) contains the total of 3,855 quantified proteins along with the average of their relative expression with respect to each differentiation stage. The following analyses were conducted on the “Averages of the Replicates" table.

**Table S4:** Cluster 1, 2, and 3 tables present each cluster's members, their relative expression, and their membership score. The highlighted BPs table involves the highlighted biological processes of each protein cluster.

**Table S5:** Wnt signalling–related GOs of the demonstrated Wnt signalling–associated proteins in Fig. 4A. These proteins' relative expression changes and the resultant GOs of their enrichment analysis are presented in Fig. 4A_GO, Fig. 4A_expression, and Fig. 4B sheets, respectively.

**Table S6:** The relative expression of the autophagy-associated proteins, which have been illustrated by heatmap in Fig. 5.

**Table S7:** The first 6 sheets (named Fig. 6A–F) provide the tabular illustration of the stage-specific proteins of the oligodendrocyte lineage differentiation. The table in “Fig. S5" sheet demonstrates the relative expression changes of the stage-specific proteins. Each table provides supporting data for the figure with the same name. For the identification of the stage-specific proteins, the expression of each protein at a specific time point was compared with its expression in all other time points using the R/Bioconductor package limma.

**Table S8:** The first 5 sheets (named Fig. 7A–E) provide the tabular illustration of the stage transition–specific proteins (STSPs) in the oligodendrocyte lineage differentiation. Each sheet provides supporting data for the figure with the same name. In this analysis, the expression of each protein at a specific time point was compared with its expression at the next time point using the R/Bioconductor package limma. The table presented in the sixth sheet (Fig. 7F) involves the relative expression changes of all STSPs, the proteins shown in Fig. 7F.

**Table S9:** Detailed compositions of the culture media used in the experiments.

**Table S10:** List of antibodies used in the experiments.

**Table S11:** Oligodendrocyte lineage proteome data covered a significant number of enriched proteins, which included 1,180 enzymes and enzyme modulators, 698 nucleic acid binding and transcription factors (TFs), 425 intra/extracellular trafficking and signalling proteins, 203 cytoskeletal and extracellular matrix (ECM) proteins, and 57 structural and adhesive proteins.

## Abbreviations

Asn: asparagine; Asp: aspartate; BCA: bicinchoninic acid; bioDBnet: biological database network; BP: biological process; BSA: bovine serum albumin; CaM: calmodulin; cAMP: cyclic adenosine monophosphate; CNS: central nervous system; d0: day 0 of differentiation; d12: day 12 of differentiation; d120: day 120 of differentiation; d20: day 20 of differentiation; d8: day 8 of differentiation; d80: day 80 of differentiation; DAVID: Database for Annotation, Visualization and Integrated Discovery; DDA: data-dependent acquisition; DEP: differentially expressed protein; ECM: extracellular matrix; Eph: ephrin; EpiSC: epiblast stem cell; FA: formic acid; FDR: false discovery rate; Gln: glutamine; Glu: glutamate; GO: gene ontology; GSEA: gene set enrichment analysis; HCD: higher energy collisional dissociation; hESC: human embryonic stem cell; ictrl: inner control; IGF1: insulin-like growth factor 1; iPSC: induced pluripotent stem cell; LDN: LDN193189; mhDEP: most highly differentially expressed protein; MIN6: mouse insulinoma 6; MS: mass spectroscopy; MSigDB: Molecular Signatures Database; mTOR signalling pathway: mammalian target of rapamycin signalling pathway; mTORC1: mTOR complex 1; NADP: nicotinamide adenine dinucleotide phosphate; nanoLC/ESI-MS/MS: high-resolution nanoflow liquid chromatography positive ion electrospray ionization tandem mass spectrometry; NCE: normalized collision energy; NPC: neural progenitor cell; NSC: neural stem cell; NSPCs: neural stem and progenitor cells; NTF3: neurotrophin 3; OL: oligodendrocyte; OPC: oligodendrocyte progenitor cell; PANTHER: protein analysis through evolutionary relationships; PCA: principal component analysis; PCC: Pearson correlation coefficient; PCP: non-canonical planar cell polarity; PDGF-AA: platelet-derived growth factor AA; PKCe signalling pathway: protein kinase C epsilon signalling pathway; pO/L: poly-L-ornithine/laminin; pre-OPC: pre-oligodendrocyte progenitor cell; PRIDE: Proteomics Identifications Database; PS: protein set; RA: all trans-retinoic acid; RH6: Royan human embryonic stem cell line 6; SAG: smoothened agonist of sonic hedgehog; SB: SB431542; STSP: stage transition–specific protein; T3: 3,3,5-triiodo-l-thyronine; TF: transcription factor; TGF-β signalling pathway: transforming growth factor β signalling pathway; TMT: tandem mass tag; UniProt: Universal Protein Resource.

## Competing Interests

The authors declare that they have no competing interests.

## Authors' contributions

G.H.S., P.P., and M.J. conceived the project, designed the study, and interpreted results with the efforts of R.K., M.M., and H.B.; P.P. performed the experiments, except for the TMT experiment, which was designed, performed, and, in part, analysed by M.M., Y.W., A.A., and V.G.; A.M. contributed in RH6 preparation; P.P. and R.K. performed computational analyses and prepared figures and tables; P.P. wrote the manuscript with input from the co-authors; and G.H.S., M.M., and H.B. oversaw all aspects of the study. All authors proofread the manuscript and approved the final version.

## Supplementary Material

giaa116_GIGA-D-20-00058_Original_Submission

giaa116_GIGA-D-20-00058_Revision_1

giaa116_GIGA-D-20-00058_Revision_2

giaa116_GIGA-D-20-00058_Revision_3

giaa116_Response_to_Reviewer_Comments_Original_Submission

giaa116_Response_to_Reviewer_Comments_Revision_1

giaa116_Response_to_Reviewer_Comments_Revision_2

giaa116_Reviewer_1_Report_Original_Submission Amit Yadav -- 4/17/2020 Reviewed

giaa116_Reviewer_1_Report_Revision_1 Amit Yadav -- 8/9/2020 Reviewed

giaa116_Reviewer_2_Report_Original_Submission Tim Ahfeldt -- 4/29/2020 Reviewed

giaa116_Supplemental_Figures_and_Tables
