# Supplementary material for: The Dynamic Proteome of Oligodendrocyte Lineage Differentiation Features Planar Cell Polarity and Macroautophagy Pathways
Source: Gigascience. 2020 Oct 31;9(11):giaa116. doi: 10.1093/gigascience/giaa116 (PMC7601170; doi:10.1093/gigascience/giaa116)
Supplement: giaa116_GIGA-D-20-00058_Original_Submission [file giaa116_giga-d-20-00058_original_submission.pdf]

GigaScience

The Dynamic Proteome of Oligodendrocyte Lineage Differentiation Features Planar Cell Polarity and Macroautophagy Pathways

--Manuscript Draft--

**Manuscript Number:**	GIGA-D-20-00058
**Full Title:**	The Dynamic Proteome of Oligodendrocyte Lineage Differentiation Features Planar Cell Polarity and Macroautophagy Pathways
**Article Type:**	Data Note
**Funding Information:**	Royan Institute (243154) — Ms Paria Pooyan
**Abstract:**	Background<br><br>Generation of oligodendrocytes (OLs) is a sophisticated multistep process, mechanistic underpinnings of which are not fully understood and demand further investigation. To systematically profile proteome dynamics during human embryonic stem cell (hESC) differentiation into OLs, we applied in-depth quantitative proteomics at different developmental stages and monitored changes in protein abundance using a multiplexed tandem mass tag (TMT) based proteomics approach.<br><br>Findings<br><br>Our proteome data provided a comprehensive protein expression profile that highlighted specific expression clusters based on the protein abundances over the course of human OL lineage differentiation. We identified the eminence of the planar cell polarity (PCP) signaling and autophagy (particularly macroautophagy) in the progression of OL lineage differentiation; the cooperation of which is assisted by 106 and 77 proteins, respectively, that showed significant expression changes in this differentiation process. Further, differentially expressed protein (DEP) analysis of the proteome profile of OL lineage cells revealed 378 proteins that were specifically up-regulated only in one differentiation stage. In addition, comparative pairwise analysis of differentiation stages demonstrated that abundances of 352 proteins differentially changed between consecutive differentiation time points.<br><br>Conclusion<br><br>Our study provides a comprehensive systematic proteomics profile of OL lineage cells that can serve as a rich resource not only for identifying novel biomarkers from these cells, but also for indicating numerous proteins that may contribute to the regulation of the development of myelinating OLs and other cells of OL lineage. We showed the importance of PCP signaling in OL lineage differentiation, and revealed the autophagy-related proteins that participate in OL lineage Differentiation.
**Corresponding Author:**	Ghasem Hosseini Salekdeh<br>Macquarie University Faculty of Science and Engineering<br>Sydney, NSW AUSTRALIA
**Corresponding Author Secondary Information:**	
**Corresponding Author's Institution:**	Macquarie University Faculty of Science and Engineering
**Corresponding Author's Secondary Institution:**	
**First Author:**	Paria Pooyan
**First Author Secondary Information:**	
**Order of Authors:**	Paria Pooyan<br>Razieh Karamzadeh

Powered by Editorial Manager® and ProduXion Manager® from Aries Systems Corporation

	Mehdi Mirzaei
	Anna Meyfour
	Ardeshir Amirkhan
	Yunqi Wu
	Vivek Gupta
	Hossein Baharvand
	Mohammad Javan
	Ghasem Hosseini Salekdeh
**Order of Authors Secondary Information:**	
**Additional Information:**	
**Question**	**Response**
Are you submitting this manuscript to a special series or article collection?	No
**Experimental design and statistics**<br><br>Full details of the experimental design and statistical methods used should be given in the Methods section, as detailed in our Minimum Standards Reporting Checklist. Information essential to interpreting the data presented should be made available in the figure legends.<br><br>Have you included all the information requested in your manuscript?	Yes
**Resources**<br><br>A description of all resources used, including antibodies, cell lines, animals and software tools, with enough information to allow them to be uniquely identified, should be included in the Methods section. Authors are strongly encouraged to cite Research Resource Identifiers (RRIDs) for antibodies, model organisms and tools, where possible.<br><br>Have you included the information requested as detailed in our Minimum Standards Reporting Checklist?	Yes
**Availability of data and materials**	Yes

All datasets and code on which the conclusions of the paper rely must be either included in your submission or deposited in publicly available repositories (where available and ethically appropriate), referencing such data using a unique identifier in the references and in the “Availability of Data and Materials” section of your manuscript.<br><br>Have you have met the above requirement as detailed in our Minimum Standards Reporting Checklist?	

Powered by Editorial Manager® and ProduXion Manager® from Aries Systems Corporation

Manuscript Click here to access/download;Manuscript;Pooyan et al.docx

The Dynamic Proteome of Oligodendrocyte Lineage Differentiation Features Planar Cell Polarity and Macroautophagy Pathways

Paria Pooyan[1,2,3], Razieh Karamzadeh[1,2], Mehdi Mirzaei[4,5], Anna Meyfour[6], Ardeshir Amirkhan[5], Yunqi Wu[5], Vivek Gupta[7], Hossein Baharvand[2,3,8*], Mohammad Javan[3,9*], Ghasem Hosseini Salekdeh[1,4*]

[1] Department of Molecular Systems Biology, Cell Science Research Center, Royan Institute for Stem Cell Biology and Technology, ACECR, Tehran, Iran

[2] Department of Stem Cells and Developmental Biology, Cell Science Research Center, Royan Institute for Stem Cell Biology and Technology, ACECR, Tehran, Iran

[3] Department of Brain and Cognitive Science, Cell Science Research Center, Royan Institute for Stem Cell Biology and Technology, ACECR, Tehran, Iran

[4] Department of Molecular Sciences, Macquarie University, Sydney, New South Wales 2109, Australia.

[5] Australian Proteome Analysis Facility, Macquarie University, North Ryde, NSW, Australia

[6] Basic and Molecular Epidemiology of Gastrointestinal Disorders Research Center, Research Institute for Gastroenterology and Liver Diseases, Shahid Beheshti University of Medical Sciences, Tehran, Iran

[7] Department of Clinical Medicine, Macquarie University, Sydney, NSW, Australia

[8]Department of Developmental Biology, University of Science and Culture, Tehran, Iran

[9]Department of Physiology, Faculty of Medical Sciences, Tarbiat Modares University, Tehran, Iran

*Corresponding authors

Ghasem Hosseini Salekdeh, Ph.D., Mohammad Javan, Ph.D., Hossein Bahrvand, Ph.D.

Royan Institute, Banihashem Sq., Banihashem St., Resalat highway, Tehran, Iran. Postal Code: 1665659911, P.O. Box: 16635-148, Tel: +98 21 22306485, Fax: +98 21 23562507. Emails: Hosseini.salekdeh@mq.edu.au, mjavan@royaninstitute.org, baharvand@royaninstitute.org

Abstract

Background: Generation of oligodendrocytes is a sophisticated multistep process, mechanistic underpinnings of which are not fully understood and demand further investigation. To systematically profile proteome dynamics during human embryonic stem cell differentiation into oligodendrocytes, we applied in-depth quantitative proteomics at different developmental stages and monitored changes in protein abundance using a multiplexed tandem mass tag-based proteomics approach. **Findings:** Our proteome data provided a comprehensive protein expression profile that highlighted specific expression clusters based on the protein abundances over the course of human oligodendrocyte lineage differentiation. We identified the eminence of the planar cell polarity signaling and autophagy (particularly macroautophagy) in the progression of oligodendrocyte lineage differentiation; the cooperation of which is assisted by 106 and 77 proteins, respectively, that showed significant expression changes in this differentiation process. Further, differentially expressed protein analysis of the proteome profile of oligodendrocyte lineage cells revealed 378 proteins that were specifically up-regulated only in one differentiation stage. In addition, comparative pairwise analysis of differentiation stages demonstrated that abundances of 352 proteins differentially changed between consecutive differentiation time points. **Conclusion:** Our study provides a comprehensive systematic proteomics profile of oligodendrocyte lineage cells that can serve as a rich resource for identifying novel biomarkers from these cells, and for indicating numerous proteins that may contribute to the regulation of the development of myelinating oligodendrocytes and other cells of oligodendrocyte lineage. We showed the importance of planar cell polarity signaling in oligodendrocyte lineage differentiation, and revealed the autophagy-related proteins that participate in oligodendrocyte lineage Differentiation.

Keywords: human embryonic stem cell; neural stem cell; progenitor cell; oligodendrocyte; Wnt signaling; autophagy; quantitative proteomics; multiplexed tandem mass tag

Background

Oligodendrocytes (OLs; for abbreviations, please refer to Table S1) are the myelinating cells of the central nervous system (CNS) that insulate axons with their multispiral membrane-forming myelin. Therefore, OLs allow swift saltatory conduction of action potentials in the CNS [1]. The functional significance of OLs is manifested through myelin loss, in addition to its damage or dysfunction-related neurological disorders such as multiple sclerosis (MS), optic neuritis (ON), spinal cord injury (SCI), and Pelizaeus–Merzbacher disease (PMD) [2]. Irrespective of its background, myelin loss and nervous system failure in remyelination lead to conduction hindrance along the axonal fibers, followed by the destruction of nerve impulses, degenerative axonal loss, and the accumulation of functional disabilities [3]. Oligodendrocyte progenitor cells (OPCs) are the main source of new OLs that can carry on the remyelination process, while neural stem cells (NSCs) and neural progenitor cells (NPCs) may also contribute in new OL generation. In a nutshell, remyelination demands the activation, recruitment, and OL-differentiation of OPCs, and possibly their predecessors [2].

A deeper understanding of the biology of myelinating OL generation alongside their progenitors can equip us with invaluable tools to achieve proper remyelination and preventing further clinical complications of the diseases related to myelin destruction. To accomplish this goal, it is necessary to understand the ways to 1) improve NSCs, NPCs, and OPCs migration into the required site; 2) enhance the mentioned cells survival especially during this process; and 3) boost their differentiation into myelinating OLs in demyelination niche. To fulfill these prospects, we conducted an in-depth quantitative proteomic analysis that spanned the entire course of OL lineage cells generation in an attempt to survey the order, timing, and magnitude of proteome changes during human embryonic stem cell (hESC) differentiation into OL lineage cells. This versatile differentiation model system provides tremendous insight into human OL development, in addition to the information needed for targeted/specific cell-based medical therapies and overall disease modeling [4,5].

Therefore, we studied the global proteome signature of developing OL by conducting a stepwise differentiation process to differentiate the hESC RH6 line into an OL lineage. This process provided us with cell samples from each of the distinct stages of OL differentiation: hESCs, NSCs, NPCs, pre-OPCs, OPCs, and OLs [6,7]. We attempted to spot both the key proteins of each step achievement and the biomarkers of every stage in OL lineage differentiation. Our study provides an inclusive profile of the proteins involved in Wnt signaling throughout OL lineage differentiation. Our findings put planar cell polarity (PCP) noncanonical Wnt signaling in the spotlight for further analysis of this controversial signaling of OL differentiation. In addition, the proteome of OL lineage differentiation presents an all-embracing autophagy associated protein profile and accentuates macroautophagy as a valuable contributing factor in OL lineage differentiation.

Data Description

Global characterization of protein expression during oligodendrocyte (OL) generation

In order to provide a systematic proteomic profiling map of the representative cells in human OL development, the hESC line Royan H6 (RH6) [8] was differentiated into OL lineage through a well-defined stepwise protocol [6,7,9] (Fig. S1A). Briefly, hESC (d0, Fig. S1B) differentiation was initiated by neural induction through dual SMAD inhibition; within eight days, we observed the presence of SOX1$^+$ NSCs (d8, Fig. S1C). Further treatment of NSCs by caudalizing and ventralizing morphogens, gave rise to OLIG2$^+$ NPCs on day 12 (d12, Fig. S1D), which then committed to an OL lineage by day 20 (d20, NKX2.2$^+$ pre-OPCs, Fig. S1E). Next, maturation of pre-oligodendrocyte progenitor cells (pre-OPCs) was promoted via a chemically defined, growth factor-rich medium, and PDGFRA$^+$ OPCs were generated on day 80 (d80, Fig. S1F). Finally, PDGFRA$^+$ OPCs were terminally differentiated into MBP$^+$ OLs (d120, Fig. S1G).

Following recapitulation of OL lineage development, the cells were harvested at six distinct time points (in three biological replicates per time point), which corresponded to

the hESC (d0), NSC (d8), NPC (d12), pre-OPC (d20), OPC (d80), and OL (d120) differentiation stages (Fig. 1) [6,7]. Then to accommodate all the biological replicates of each time point, three tandem mass tag (TMT) mass spectrometry experiments were carried out (Fig. 1).

For in-depth quantitative proteomic analysis, the harvested cells were homogenized and protein extracts of each sample were treated with lysine-C/trypsin sequential digestion. Subsequently, the peptides were quantified and subjected to TMT labelling (Fig. 1), then fractionated and analysed by a high-resolution nanoflow liquid chromatography positive ion electrospray ionization tandem mass spectrometry (nanoflow LC/ESI-MS/MS) on a Q Exactive Orbitrap mass spectrometer (Thermo Scientific). Therefore, upon fragmentation in MS/MS mode, sequence assignment of the MS/MS spectra achieved using indexed human UniProt database [10], and next quantification of relative protein expression changes accomplishment from the fragmentation of the tags, which gave rise to mass reporter ions. The mass spectrometry proteomics data can be retrieved via the ProteomeXchange Consortium [11] through the PRIDE partner repository (accession code: PXD017649). In total, at false discovery rate (FDR) of 1%, a total of 66,083 peptides and 59,404 unique peptides from 5,753 unique proteins were identified; among them, 3,527 unique proteins were identified within all three biological replicates (Fig. S2A), and 3,524 of unique proteins were quantified across all time points. Furthermore, 1,056 proteins were found to be in common only between two replicas (Fig. S2A) of which quantitative measurements of 328 proteins could be imputed using a supervised approach [12]. Importantly, according to the analysis of variance (ANOVA) about 81% (3132 proteins) of the proteins showed significant changes through the OL lineage differentiation of hESCs. Pearson correlation coefficient coupled with hierarchical clustering (using the relative expression for all of the 3,855 quantified proteins) implied a high degree of consistency among sample replicates (Fig. 2A, Table S2). The heat map presentation of the protein distribution profiles demonstrates five distinct groups associated with the differentiation steps. It also represents d8 (NSC stage), d12 (NPC stage), and d20 (pre-OPC stage) in one supergroup, and d20, d80 (OPC stage), and d120 (OL stage) in another supergroup. Therefore, in agreement with the sequential stages of

the differentiation process, d20 demonstrated a transition state between the initial and final steps (Fig. 2A). The standard Principal component analysis (PCA) was performed to project the proteome profile of each differentiation time point into a two-dimensional space. PCA clustered all three replicates of each time point together (Fig. 2B). In order to evaluate the functional diversity of the detected proteins, we classified the total proteins into 26 classes using the PANTHER (PANTHER13.1) classification system — out of 29 indexed parent protein class terms (Fig. S2B) [13]. Our data covered a significant number of enriched proteins that included 1,180 enzymes and enzyme modulators, 698 nucleic acid binding and transcription factors (TFs), 425 intra/extracellular trafficking and signaling proteins, 203 cytoskeletal and extracellular matrix (ECM) proteins, and 57 structural and adhesive proteins, indicating the essential role of catalytic activity, gene expression, biosynthesis/trafficking processes and cellular structure in addition to their surroundings, OL differentiation (Fig. S2B).

Results and Discussion

Oligodendrocyte (OL) lineage differentiation of the human embryonic stem cells (hESCs) is led by the co-operation of three protein clusters

To get a deep understanding of major functional players during the OL lineage differentiation, we explored the dynamic view of the proteome expression during OL differentiation using unsupervised fuzzy c-means clustering on all quantified proteins. As a result, a total of 3,855 proteins were segregated into three clusters by their expression trends during differentiation. The clusters' functional enrichment analysis was performed against the Gene Ontology (GO) Biological Process (BP) gene set collection (2018) to ascertain functional groups associated with this differentiation progress (Fig. 3).

Cluster 1, consisted of the majority of proteins (2,223), demonstrated a slight decreasing expression profile (d0 to d120). Based on the functional enrichment analysis, this cluster mostly contained proteins that contribute to gene expression and translation (Fig. 3A). The two other clusters reflected increasing trends in accordance with the progression and specification of the differentiation process (Fig. 3B, C). Both clusters shared some

common developmental terms with regard to cellular structure, migration, division, and secretion, that are also in agreement with the OL development; however, cluster 2 with a slightly increasing pattern, seemed to be more involved in early developmental processes by enrichment of the GO terms predominantly related to the regulation of neural stem and progenitor cells (NSPCs), pre-OPCs and OPCs maintenance and differentiation. This was reflected by GO terms, including “vesicle transport” [14], “ephrin (Eph) receptor signaling pathway” [15], “receptor protein tyrosine kinase signaling pathway” [16], “sterol biosynthesis” [17], “nicotinamide adenine dinucleotide phosphate (NADP) metabolic process” [18] and “canonical glycolysis” [18] (Fig. 3B). On the other hand, cluster 3, which indicated an upward trend, mostly on d20 (pre-OPC stage) to d120 (OL stage), revealed enrichment of proteins mainly involved in OPCs differentiation into OLs, OL maturation and myelin formation, such as “gliogenesis”, “positive regulation of calcium ion transport” [19–22], “regulation of filopodium assembly” [23], “regulation of nitric-oxide synthase activity” [24], “response to peptide hormone” [25] and “positive regulation of lipid metabolism” [26] (Fig. 3C). These results indicated that the functional enrichment of the derived proteome architecture was associated with the corresponding differentiation states.

To confirm the authenticity of our clusters, we checked the expression patterns of several marker proteins related to the OL differentiation stages. The depicted heat map showed that the time-dependent changes of these markers was consistent with their fitted clusters and aligned with the progression of the differentiation process (Fig. 3D). Notably, the hESC (d0) markers and regulators of stem cell proliferation, including POU5F (OCT4) and LIN28A, along with NSC (d8) markers SOX2, SOX3, and MSI were grouped in cluster 1, which had a gently decreasing pattern [27–29]. Nevertheless, CDH2, an NSC (d8) marker that is also known to be highly expressed in myelinating OLs [30,31], like OPC (d80) and OL (d120) markers (PTPRZ, CD9, CNP, and GALC), was classified in cluster 2 (with a slightly increasing trend). Likewise, OPC (d80) and OL (d120) specific proteins, i.e. CNTN1, SIRT2, NDRG1, ACTR1, and GSN were located in cluster 3 (with a sharp upward trend) [29,32–35]. In general, these results revealed the expression distribution of enriched proteins with stage-specific biological functions within our three clusters,

which may assist us to identify key proteome signatures associated with OL lineage differentiation. Therefore, this dynamic proteome outlook could support additional discovery of potentially competent proteins for *in vivo* OL differentiation of various neural precursors in patients with myelination defects. It also may provide us with stage-specific profiles that correlate with predominant biological functions associated with this differentiation process.

Canonical and non-canonical Wnt signaling protein profiling during the generation of the oligodendrocyte (OL) lineage cells

Looking through enriched BPs of the clusters, we found the Wnt signaling pathway to be prominently affected. This pathway has been shown to be involved not only in OL development but also in other developmental processes [36]. We observed enrichment of the GOs related to the regulation of the Wnt signaling pathway, and non-canonical Wnt signaling pathways, particularly planar cell polarity (PCP) pathway in both clusters 1 and 2 (Fig. 3A-, B-left charts), which seems to have a complementary function in this context. Wnt signaling pathways, including Wnt/β-catenin (canonical) pathway, Wnt/$Ca2^{+}$ (non-canonical) pathway, and PCP (non-canonical) pathway are fundamental mechanisms associated with various levels of vertebrate developmental procedures [37,38].

Systematic analysis of our data by applying DAVID (DAVID 6.8) and UniProt (besides Enrichr, used in clusters functional enrichment analysis) revealed that 150 Wnt signaling related proteins enriched in the OL lineage differentiation process [39–42]. We found that 86 proteins from cluster 1, 56 from cluster 2, and 8 from cluster 3 orchestrated Wnt signaling pathways during OL lineage differentiation (Fig. 4A). According to the heat map illustration of the relative abundances of Wnt signaling related proteins, we noticed that the Wnt signaling pathways seemed to be highly active in this differentiation process distinctly at the hESCs (d0) and late (OPC and OL) stages (Fig. 4A). In support of our observations in cluster enrichment analysis, we noted that these proteins are mainly involved in GO related to the regulation of both canonical and non-canonical Wnt signaling pathways, especially the PCP pathway (Fig. 4B). To scrutinize the contribution of Wnt signaling components in detail, we also applied Gene Set Enrichment Analysis (GSEA) using whole protein expression profiles.

Remarkably, Wnt signaling pathway was enriched especially at three last time points of differentiation (i.e. d20, d80 and d120) compared to the other days (FDR q-value 0.029) indicting the importance of this pathway in OL differentiation (Fig S3). In general, this feature supported the results of previous studies that mentioned the crucial implication of canonical Wnt signaling in regulating stemness and development of ESCs; specification, proliferation, and differentiation of OPCs; and maturation and myelination of OLs [43–45]. Even though some observations made canonical Wnt signaling impact on these three cell types baffling, based on the ultimate outcome of all former studies, this cascade's effect is amenable to the developmental stage, microenvironment, and intensity of the signaling [43,46–50].

The enriched PCP pathway that regulates cell polarity is implicated in cellular morphogenesis, migration, intercalation, and function [51,52]. The high abundance of PCP pathway-related proteins may convey the necessity of polarization in OPC specification, migration, and differentiation, as well as OL maturation and myelination. Each would need additional functional investigation as there cannot be found any study that explored the PCP pathway in cells of the OL lineage. In addition, significant enrichment of PCP pathway-related proteins in hESCs possibly implies their role in modulating stem cell self-renewal [53].

Briefly, these findings represent non-canonical Wnt signaling pathways, especially the PCP pathway, as an enticing field of study in the OL development. The results can potentially improve our knowledge about the implications of all of the Wnt signaling pathways in hESCs maintenance and its further differentiation into the particularly OL lineage; specification, migration and differentiation of OPCs; and OL maturation and myelination, with the ultimate goal of restoring myelin regeneration in diseases associated with myelin deficiencies.

Macroautophagy-associated protein profile of the generation of oligodendrocyte (OL) lineage cells

Another set of remarkable BPs in our cluster enrichment analysis was related to autophagy (Fig. S4A), an important lysosomal degradation and recycling pathway in mammalian cell development and differentiation [54]. Autophagy is a substantial issue in developmental processes. Because of the lack of a comprehensive study on the role of autophagy in OL lineage development or differentiation, we sought to peruse the proteome signature of the autophagy pathway through OL lineage differentiation of hESCs.

Autophagy is a highly conserved lysosomal-mediated cellular pathway responsible for catabolism plus recycling of damaged or outlived intracellular cargoes (macromolecules and organelles) to maintain cellular homeostasis and assist cellular structural remodeling during normal development and differentiation. The most common form of autophagy in eukaryotic cells is macroautophagy, which is mainly referred to as autophagy. In this major cellular degradation pathway, double-membrane vesicles (autophagosomes) engulf the cytoplasmic cargos and digest them through the autophagosome-lysosome system [55,56] (Fig. S4B).

Further functional analysis of our proteome data using DAVID and UniProt [39,40,57] showed that the proteins involved in autophagy, particularly macroautophagy, were enriched through the OL lineage differentiation process. Our analysis highlighted the BPs engaged in autophagy, macroautophagy, autophagosome formation, and their regulation (Fig. 5). It featured the expressions of key upstream triggers of this pathway, AMP-activated protein kinases PRKAA1, PRKAA2, and PRKAG1 [58] in addition to the proteins involved in the early stages of autophagosome formation, MAP1LC3B2 (a member of LC3s) and PI3KC3 [58]. Our data also showed enrichment of the autophagy-related (ATG) proteins (ATG16L1, ATG5, ATG7, ATG3, ATG2B, and ATG9A) that, in cooperation with LC3s (GABARAPL2 and PI3KC3), control major steps of autophagy, including autophagosome expansion, maturation and lysosomal fusion, as well as cargo recruitment, degradation, and the recycling system [59] (Fig. 5 and Fig. S4). Relative expression heat map of the 107 proteins involved in autophagy (found in our data) showed that 80 proteins were members of cluster 2. This may show the major influence of autophagy and macroautophagy in both specification and function of NPCs, pre-OPC,

and OPCs, in addition to OL maturation and myelination (Fig. 5). Our results corroborated previous findings that showed the crucial role of macroautophagy in OPC/OL differentiation, survival, maturation, and proper myelin development. In addition, these findings also brought up a possible vital role of this pathway in the early OL developmental stages [60,61]. Furthermore, our data provided a novel proteome profile of autophagy-associated proteins through the OL lineage differentiation process. It revealed proteins related to each cell type (hESCs, NSCs, NPCs, pre-OPCs, OPCs and OLs) and their expression trends in their generation process. Therefore, this may provide researchers with a tremendous repository for designing more effective therapeutic strategies toward demyelination disorders.

Due to its cytoprotective role, autophagy is increasingly believed to promote neuronal and OL survival. However, in a disease like MS (the most well-known example of demyelinating diseases), therapeutic intervention of autophagy gets greatly complicated since MS is an inflammatory-mediated demyelinating disease, wherein cells of the immune system destroy the myelin sheaths of the nerve axons in the CNS and this is followed by neurodegeneration of both myelinating cells (OPCs and OLs) and neurons [62]. While studies show that pharmacological inducers of autophagy, like rapamycin, can improve myelination of OLs and Schwann cells (SCs; the myelinating glia of the peripheral nervous system), the elevated levels of autophagy in immune cells of MS patients makes this type of medications hazardous [60,63–66]. Nevertheless, our proteome data can be considered as a valuable source for finding an appropriate way of the targeted drug delivery system for these types of medications [67].

Identification of potential biomarkers in each step of human embryonic stem cell (hESC) differentiation into oligodendrocytes (OLs)

Next, we investigated the identification and abundance of proteins that differentially expressed only in one of the differentiation stages. We observed significant (>2-fold) changes in the numbers of differentially expressed proteins (DEPs) in the hESC (d0, n=4), NSC (d8, n=57), NPC (d12, n=9), pre-OPC (d20, n=22), OPC (d80, n=24), and OL (d120, n=251) differentiation stages (Fig. 6 and Fig. S5).

According to Our DEP analysis, four proteins (HMOX1, MT1E, MT2A and ASNS) showed their specificity at hESC (d0) state, all of which are cytoprotective factors [68–70]. Among them, the specific role of HMOX1 (Heme oxygenase-1) in the maintenance of self-renewal and pluripotency of ESCs and induced pluripotent stem cells (iPSCs) is well-studied [71–73]. Even though the roles of MT1E and MT2A in ESCs are still not specifically studied, emerging evidence shows the remarkable expression of these metallothioneins in ESCs and iPSCs [74–76]. ASNS (asparagine synthetase), another deferentially up-regulated protein in hESCs, is an ATP-dependent enzyme that synthesizes asparagine (Asn) and glutamate (Glu) at the expense of aspartate (Asp) and glutamine (Gln) [77]. Glu can be used in Gln synthesis by glutamine synthetase (GLUL), an enzyme which has been shown to be involved in cell proliferation, and demonstrated a considerable expression in all steps of OL lineage differentiation (data not shown) [78]. On the basis of previous studies, Gln and Asn act as principal survival and self-renewal factors in ESCs and cancer cells, respectively [77,79,80]. Ryu *et al.* have reported that Gln is an important factor in regulating maintenance of mouse ESCs through transcription regulation via the Akt, PKCe, and mTOR signaling pathways [81]; while Krall *et al.* have revealed that Asn is a powerful regulator of cell amino acid homeostasis, therefore it controls mTOR complex 1 (mTORC1) activation, cellular anabolic metabolism and proliferation [77]. Consequently, this data suggests that the four DEPs (HMOX1, MT1E, MT2A and ASNS) can be efficient biomarkers for ESCs.

As mentioned above, we found 57 proteins that were specifically related to NSCs (Fig. 6 and Fig. S5). Most of these proteins, like SOX2, CBX2, CBX5, HMGB2, RHF6, RBMS1 and SALL1 (all of which has been already reported as NSC specific gene by Xiao *et. al.*) [82] are involved in BPs related to chromatin organization and gene expression, reflecting the needed trigger for the differentiation onset. Epigenetic modification of chromatin, in response to differentiation cues, controls gene expression in different cellular transitions, such as the differentiation of hESC into NSC [83]. The identified NSC state-specific proteins, like LUZP1 [84,85], SOX2, PPT1 [86], SMOC1 [87], MAZ [88,89], CRABP1 [90], GKAP1 [91] and CSRP2 [92], are also involved in BPs associated with cell division, proliferation and differentiation of specially nervous system. These findings demonstrate

that NSC state-specific proteins (Fig. 6 and Fig. S5) can be used as biomarkers of active NSCs.

As illustrated in Fig. 6, there are only 9 proteins with specifically high abundances at the NPC stage (Fig. 6 and Fig. S5). These proteins are mainly associated with cellular junction, adhesion, mitosis, proliferation as well as cytoskeleton proteins [93], however, apart from FREM2 [94,95], the roles of the other proteins in NPC maintenance and function are not well documented [96]. According to previous studies, that highlighted the importance of cell-cell connections in NSPCs biological behavior [97–99], our data may bring the three differentially expressed desmosomal proteins, DSP, JUP and PKP2, into the spotlight for further investigation of the impact of the desmosome junction on providing more desirable niches for NPCs maintenance, proliferation and differentiation. These findings may lead to the introduction of new potential biomarker proteins for NPC state.

The two most highly DEPs (mhDEPs with Log2 (fold-Change) ≥ 5) on d20, MDK and RBP1, truly reflect the role of this leading step, the pre-OPC stage, in OL generation. Both mhDEPs instigate pre-OPC specification toward OL differentiation [100–103]. Among the 22 DEPs at the pre-OPC stage (Fig. 6 and Fig. S5), there are 10 members of histone family (H1F0, H1FX, H2AFX, H2AFZ, HIST1H1B, HIST1H1C, HIST1H1D, HIST1H2BM, HIST1H3A and HIST2H2AB) involved in chromatin organization and show the dynamics of chromatin interaction through this differentiation state. This finding may indicate the significance of histone mark repatterning and remodeling of the chromatin architecture at the NPC stage (Fig. S5). The correlation of this differential re-patterning with the high abundance of known pre-OPC proteins such as MDK, RBP1 [100–103], SOX3 [104], and TNFRSF10B [105] points to the potential of these d20 DEPS to be used as biomarkers for the pre-OPC state.

Our findings also represent 24 DEPs at OPC state (Fig. 6, and Fig. S5), four (FN1, TGFBI, TNC and COL3A1) of which have been previously reported as DEPs of OPC by Chaerkady *et al.* [106]. FN1 (Fibronectin) is a glycoprotein of the extracellular matrix (ECM) that stimulates OPC proliferation [107], also has the capacity to impair oligodendrocyte differentiation and myelin sheath formation [108–110]. In OPC, FN1 with

PDGFA accompaniment leads to the actin-pERK1 and 2 co-localization and formation of filopodia, thus enhances the migration of these cells [111]. The impact of the ECM protein, TGFBI is not completely clear; however, it is known that TGFBI can regulate cellular adhesion and migration [112]. TNC, another ECM-glycoprotein, is produced by OPCs and preserves their proliferation. Down-regulation of TNC is followed by OL maturation [113–115]. There is no information about the role of COL3A1 in maintenance or function of OPCs; however, Gao *et al.* have reported that COL3A1 is a valid biomarker for diagnostic or therapeutic strategies for glioblastoma [116]. In fact, this protein may put its impact on the maintenance of OPC's stemness. Among the other worth noting proteins at OPC state, there are three mhDEPs - GAP43, ATP2B2 and PCSK1N. GAP43 is a membrane protein in OPCs, and its expression decreases during OL differentiation [117]. ATP2B2, a magnesium-dependent enzyme that catalyzes the hydrolysis of ATP coupled with calcium transport, is a known constituent of the CNS myelin proteome [35] but its function in OPCs remains to be investigated. PCSK1N, a neuropeptide [118], is a proliferation-related molecule [119]. There is no evidence that directly focuses on the role of PCSK1N in OPCs function or maintenance; however, it has been reported that, in mouse insulinoma 6 (MIN6) cell line, Pax6 can directly down-regulate *Pcsk1n* expression [120]. In addition, it is shown that, in the embryonic chick neural tube and spinal cord, Pax6 promotes OPC migration [121], the process which is usually followed by Oligodendrocyte generation. Therefore, it can be hypothesized that PCSK1N may support the proliferation of OPCs. All in all, the company of the DEPs that are associated with cell proliferation and migration can indicate their utility as potential biomarkers at the OPC stage (d80).

Finally, we identified 251 DEPs on d120 (Fig. 6 and Fig. S5), among which the transcripts of 40 proteins in the OL state have been previously reported: 4 transcripts by Hu *et al.* [122], 18 transcripts by Najm *et al.* [115], and 25 transcripts by Lager *et al.* [123]. Enrichment analysis of the 251 specifically up-regulated proteins in the OL state by Enrichr [41,42], showed that these proteins participate in OL developmental-related BPs such as ECM and cytoskeleton organization (*e.g.*, BCAN, CD44, CDK5, FMNL2, HTRA1, ITGB4, PFN2 and TGFB2), nervous system development and OL differentiation (*e.g.*, BCAN, CDK5, CNP, DPYSL2, FYN, MAPK1 and LSAMP), post-translational regulation

of gene expression (*e.g.,* CRYAB, INS, LUM, PACS2, RRAGA, SEPT3 and TGFB2), L-glutamate transport (*e.g.,* ARL6IP5, PRAF2 and SLC1A3) and fatty acid metabolic process (*e.g.,* ABCD3, ACOX1, ACADS, BDH2, CRAT, CRYL1 and DECR1). According to neXtProt (v2.24.0), amongst these 251 DEPs, there are proteins associated with myelination such as TSPAN3, NDRG1, CA2, EPB41L3, CLU, GALC, ANXA2, GPC1 and AKT1 [93]. In addition, amongst the 40 DEPs that our study shares with the above mentioned works [115,122,123], there are six proteins with well-known functions in promoting OPC development into mature OL: CNTN1 [33], SIRT2 [32,124], TPPP [125], TGFB2 [126], CNP [127] and CDK5 [128]. These findings show the tremendous potential use of the identified DEPs as OL differentiation inducer, and potential biomarkers for diagnostic and prognostic purposes.

Therefore, our study, the first extensive step by step proteomic profiling of hESC differentiation into OL lineage, reveals several novel proteins that are potentially a part of this differentiation process and OL generation. These results propose a total of 378 potential biomarkers for every cell type of this lineage.

Coordinated proteome dynamics of the stage transition specific proteins (STSPs) throughout oligodendrocyte (OL) lineage differentiation

Due to the time-series nature of our study, which enables a deeper insight into the development of every cell type in the OL lineage, subsequently, we sequentially compared pairwise stages towards their achievements by identifying stage transition specific proteins (STSPs) (Fig. 7). Comparative pairwise analysis of differentiation stages showed distinct sets of STSPs (PS 1-14) that meticulously escort sequential steps of this differentiation process (Fig. 7F). At first glance, our results distinctly highlight the weighty role of transition between hESC(d0) – NSC (d8), and pre-OPC (d20) – OPC (d80) by up-regulation of 129 and 152 proteins respectively (Fig. 7A, D). It also displays a considerable transition from NSC (d8) to NPC (d12), NPC (d12) to pre-OPC (d20), and OPC (d80) to OL (d120) by down-regulation of 41 and up-regulation of 38 and 40 proteins respectively (Fig. 7B, C, E).

Rolling down across the Waddington landscape toward NSCs (d8) generation, hESCs (d0) go through the significant down-regulation of HMOX1 and ASNS, two potential hESC (d0) biomarkers (discussed in **Identification of Potential Biomarkers in every step of hESC differentiation into OL**), as well as up-regulation of 129 proteins which may be divided into 2 protein sets (PSs; PS 2 and 3) (Fig. 7F). PS 2 (includes 93 up-regulated proteins on d8) and PS 3, respectively comprise 26 and 29 potential NSC biomarkers. As previously mentioned, on d8, we found 57 NSC stage-specific proteins, 55 of which also show their stage transition specification. The up-regulated STSPs of the NSC (d8) stage also share 54 differentially expressed proteins with those DEPs found in fibroblast-derived NSC reported by Xiao *et. al.* [82]. A number of them are known to be associated with NSC development and function such as SMOC1 [87], SOX2, CRABP1 [90], CSRP2 [92], CRABP2 [129], CTNND2 [130], SOX3, MSI1 [131], HMGB2 [132] and LAMB1 [133]. Moreover, among the 129 up-regulated STSPs at NSC (d8) stage, 36 STSPs (PS 3) show a transient trend as their abundances decrease while approaching the NPC (d12) state. In parallel to the 36 transient STSPs, there are 5 down-regulated STSPs (PS 4) composed of PBDC1, LSM14B, CIC, LSM12, and PHPT1 in addition to 5 up-regulated STSPs (PS 5 and 6) toward reaching the NPC (d12) state (Fig. 7F). The high abundance of STSPs at the NPC (d12) stage include two well-known NPC proteins, MEST (NPC marker) [132], [133] and FERM2 (morphogenetic rearrangement protein of the ECM that has a crucial role in neural tube closure) [94], in addition to three intermediate filament family members (KRT19, KRT7, and KRT8). The expression levels of these STSPs, which have been presented as potential biomarkers of the NPC stage (Fig. S5), show a differential reduction shortly after NPC achievement, with the exception of MEST, which first displayed a gentle decrease reaching the pre-OPC (d20) stage, followed by a dramatic reduction during the pre-OPC (d20) to OPC (d80) conversion. These findings, in addition to those reported by Najm *et. al.*, who monitored the overexpression of MEST in pluripotent mouse epiblast stem cell (EpiSC)-derived OPCs, indicated the significance of MEST in cell fate switching from pluripotent NSCs to pre-OPCs, as more specific OL progenitors.

Taking the principal step towards more committed cells of the OL lineage, 12 highly expressed proteins (STSPs at PS 6 and 7) in the NPC (d12) stage underwent differential down-regulation while the expression levels of 38 STSPs (PS 8 and 9) increased, which promoted entrance of these cells into the pre-OPC (d20) stage (Fig. 7F). At this point, down-regulation of NPC (d12) related STSPs such as FREM2 [94], DSP [134], TPP1 [86], KRT7 [135], KRT8 [136,137], and KRT18 [138] were accompanied by up-regulation of epigenetic factors and transcription regulators (*e.g.*, MDK, HIST1H1C, HIST1H1E, H1FX, H2AFX, HMGA2, HIST1H3A, H1F0, SOX3, H2AFZ, HIST1H1B, H2AFY, HIST2H2AB, TAGLN3 and HIST1H2BM [93]) and some well-known pre-OPC STSPs (*e.g.*, MDK, RBP1 [100–103], DCHS1 [139], SOX3 [104], MAP1B [140], DPYSL4, DPYSL5 [141,142], TF [143], and TNFRSF10B [105]) (Fig. 7F). After succeeding the requisite regulation of the chromatin remodeling and gene expression, the abundance of the mediator STSPs, *e.g.*, MDK, HMGA2, HIST1H1C, SOX3, H2AFX, H1FX, HIST1H3A, H2AFZ, HIST1H1B, HIST2H2AB, H1F0, and HIST1H2BM (the transition STSPs, settled in PS 9), along with MEIS1, HIST1H1D, CRABP1, HMGA1 and HIST1H4A (members of PS 10) [93]) decreased distinctively (Fig. 7F), while pre-OPCs (d20) proceeded towards the specified OPCs (d80). Moreover, the fate specification of the cells into OPC (d80) stage was assisted with the up-regulation of 152 STSPs (PS 11 and 12) (Fig.6F), which are involved in BPs such as cellular proliferation and its regulation (*e.g.*, IGFBP7, FABP7, TNC, GNG2, CRIP2, CD47, and PRKCA), cellular differentiation and development (*e.g.*, GAP43, MAPT, VIM, GPC1, GSN, SLC1A3, and SIRT2) and cellular migration and regulation (*e.g.*, TSPAN3, L1CAM, FN1, NTN1, LMNA, RUFY3 and SULF1) [93]. Amid these up-regulated proteins (PS 11) there was a subset of STSPs (TPPP3, FHL1, GNAO1, GPM6B, DCLK2, TMOD1, CRYL1, CAVIN4, FTL, SLC27A1, CAMK2G, TSPO, PLEC, DES, TMEM65 and SIRT2), that underwent an additional significant up-regulation during OPC (d80) conversion into OL (d120) stage (Fig. 7F). These STSPs which were shared between PS 11 and PS 14, participate in cytoskeleton organization (*e.g.*, TPPP3, GPM6B, TMOD1, DCLK2, DES and PLEC [93]) a pivotal BP in OPC generation, differentiation, and migration in conjunction with OL generation and myelination. These STSPs are also involved in other OPC and OL BPs that are necessary, such as regulation of cell proliferation and cell cycle (FHL1, SIRT2, and TSPO), cell differentiation (FHL1,

CAMK2G, and CAVIN4), nervous system development (CAMK2G, GPM6B, DCLK2, SIRT2, and GNAO1), and in BPs that are implicated in the production of proteins (TPPP3, GPM6B, DCLK2, SLC27A1, SIRT2, TSPO, and GNAO1) and lipids (CRYL1, GPM6B, DCLK2, SLC27A1, SIRT2, and TSPO), which are two main components of myelin [93]. We found that OPC (d80) differentiation was accompanied by selective down-regulation of the 13 STSPs, 9 of which showed transient differential expressions at this stage (Fig. 7F). Among the transient STSPs, GAP43 and ATP2B2 were the top two most highly differentially expressed STSPs at OPC (d80) stage, with log2 (fold-Change) equal to 12.73 and 11.57, respectively. GAP43 is a OPC protein, and its expression has been shown to decrease and reach a plateau, during OPCs differentiation into OLs [144,145]. Although GAP43 is a calmodulin (CaM)-binding protein, like ATP2B2, GAP43 phosphorylation reduces its affinity for CaM. Hence, the interaction between GAP43 and CaM controls the availability of CaM [146], which then regulates the calcium pump activity of ATP2B2, and thus GAP43 takes part in the regulation of OPC maintenance and differentiation [147–149]. PS 14 is another subset of STSPs that promotes this prolonged differentiation process into its final OL target. PS 14 has 40 members, 16 of which are shared with PS 11 (as mentioned above) (Fig. 7F). All 40 STSP members of PS 14 are part of the previously mentioned OL stage-specific proteins. For instance, the merits of INS [150,151], RRAGA [152], GALC, GPM6B [153], PLEC [154], and SIRT2 [32,124] for the OL (d120) stage have been already discussed.

Consequently, these results demonstrate the outstanding potential of STSPs to improve the status of OL lineage differentiation at each of the differential stages and shed light on future mechanism based developments of demyelinating disorder associated strategies.

Methods

Human embryonic stem cell (hESC) differentiation into oligodendrocyte (OL) lineage cells. Adherent RH6 cells were induced into $SOX1^+$ NSCs by dual inhibition of SMAD signaling [155]. Under the treatment of 10 μM SB431542 (SB, inhibitor of TGF-β/activin/nodal signaling), 250 nM LDN193189 (LDN, inhibitor of bone morphogenetic protein signaling) and 100 nM all trans-retinoic acid (RA, caudalizing patterning agent) for

8 days, nearly all the differentiated cells were $SOX1^+$ NSCs (Figure S1A, C). Next, in order to mimic the embryonic ventral spinal cord environment (pMN domain) and achieve pre-OPC, 100 nM RA and 1 µM smoothened agonist of sonic hedgehog (SAG, ventralizing patterning agent) were applied for 22 days. By day 12 of differentiation, NSCs gave rise to $OLIG2^+$ NPCs (Figure S1D), which were then detached for sphere aggregation; this enriched the $OLIG2^+$ population. By day 20 of differentiation, the $OLIG2^+$ progenitors committed to the OL lineage by co-expressing NKX2.2 (pre-OPCs; Figure S1E). On day 20, supportive reagents for pre-OPCs expansion and maturation toward OPCs, and further OPCs expansion and maturation toward OL producing OPCs, *i.e.* 10 ng/ml platelet-derived growth factor AA (PDGF-AA), 5 ng/ml hepatocyte growth factor (HGF), 10 ng/ml insulin-like growth factor 1 (IGF1), 10 ng/ml neurotrophin 3 (NTF3), 60 ng/ml 3,3,5-Triiodo-l-thyronine (T3, thyroid hormone) , 25 µg/ml insulin, 100ng/ml biotin, and 1µM cyclic adenosine monophosphate (cAMP) were added to the culture medium for 60 days (Figure S1A). On day 30, spherical aggregates were plated onto poly-L-ornithine/laminin-coated dishes and on day 80 $PDGF^+$ OPCs were generated (Figure S1F). In order to eliminate the neurons and astrocytes that migrated out of the spherical aggregates and to achieve a homogenous population of OPCs, we re-plated the cells twice onto poly-L-ornithine/laminin-coated dishes, on day 65 and on day 75 of differentiation. On day 80, the growth-factors were withdrawn from the culture medium and OPCs were differentiated into MBP^+ OLs in the presence of 10 mM HEPES buffer and 20 µg/ml ascorbic acid (AA, supportive for OL differentiation), in addition to T3, insulin, biotin, and cAMP (Figure S1A, G, Table S3) [6,7].

Immunostaining. Cells were washed 3X in PBS^- (Life Technologies, cat. no. 10010023) for 3 min and then fixed with 4% (w/v) paraformaldehyde (PFA) for 15 min at room temperature (RT). Following fixation, the cells were washed 3X in 0.1% PBS^- Tween (PBS^- that contained 0.1% Tween 20) for 3 min and stored at 4°C. At the time of staining, cellular membranes were permeabilized by 0.5% PBS^- Triton (PBS^- that contained 0.5% Triton X-100) for one hour at RT. Then, the cells were incubated in blocking solution that consisted of 0.1% PBS^- Triton, 0.2% donkey serum, and 0.2% bovine serum albumin (BSA) for one hour at 37°C. After washing 3X with washing solution (0.1% PBS^- Tween),

the primary antibodies were applied overnight at 4°C (for antibodies information, please refer to Table S4). The next day, these cells were washed 3X in a washing solution for 10 min and stained with a secondary antibody for 45 min at 37°C (for antibodies information, please refer to Table S4). Thereafter, the cells were washed 3X with washing solution for 10 min, counterstained with DAPI at RT, and washed in washing solution. Images were captured using an Olympus IX71 inverted fluorescent microscope equipped with an Olympus DP72 Digital Color Microscope Camera.

Protein isolation. Total protein extraction was performed using TRIzol^{TM} reagent. Cells were washed with PBS, detached mechanically, pelleted by centrifugation, and snap frozen in liquid nitrogen before they were stored at -80°C. For each differentiation stage, we pulled three plates of cultured cells. At the time of isolation, the cell pellets were lysed and homogenized with TRIzol^{TM} reagent according to the manufacturer's instructions. Then, chloroform was used for phase separation. The samples were centrifuged for 15 min at 12000 ×g at 4°C and the clear upper aqueous phase was used for RNA isolation, the white interphase was discarded, and protein extraction proceeded by the red organic phase. Cold (-20°C) 100% ethanol was applied to dissociate DNA from proteins trapped within the lower red phenol-chloroform phase**.** Thereafter, the dissolved proteins were precipitated by an overnight incubation in acetone at -20°C. The following day, the total proteins of each sample were precipitated via centrifugation and the resultant pellets were washed with washing solution one that contained 1 ml 0.3 M guanidine hydrochloride in 95% ethanol and 2.5% glycerol, and washing solution two comprised of 100% cold ethanol that contained 2.5% glycerol. The resultant pure protein pellets were air-dried and solubilized in lysis buffer that contained 7 M urea, 2 M thiourea, 4% CHAPS detergent, 50 mM dithiothreitol, and protease and phosphatase inhibitor cocktails). In the end, the protein samples were stored at -70℃ (for provider information, please refer to Table S5).

Protein preparation. The isolated protein samples were thawed at 4℃. The concentrations of the total proteins were determined by a spectrophotometer using a modified Bradford dye-binding method [156] and BSA as the standard. A total of 300 μg of each protein sample was reduced with 5 mM dithiothreitol (Sigma-Aldrich, cat. no. D0632) for 30 min at 56℃. Thereafter, an alkylation agent, iodoacetamide (Sigma-Aldrich,

cat. no. I6125), was mixed with the reduced proteins at a concentration of 14 mM and samples were left at RT for 30 min in the dark. The process was followed by another reduction procedure. This time, after mixing 5 mM dithiothreitol with alkylated protein samples, the mixtures were kept at RT for 30 min. Subsequently, for protein shipment, protein samples were stored at -70℃ for 24 h. After 24 h, the protein samples were lyophilized for 48 h at about -50℃. Next, the lyophilized proteins were shipped in a pack filled with silica gel beads to prevent any probable dampness.

In order to remove the interfering detergents and contaminants, the proteins were precipitated using the methanol-chloroform protocol [157]. After sequential addition of ice-cold methanol, chloroform, and water with vortexing intervals, the samples were centrifuged for 2 min at 1000 ×g at 4°C. The protein aggregate at the interface layer was washed with ice-cold methanol and acetone. Next, the protein pellet was air-dried and then resuspended in 200 µl of 8 M urea in 50 mM Tris (pH 8.8). Eventually, the protein concentration was determined by a Bicinchoninic Acid (BCA) Assay Kit (Pierce, Rockford, LI, USA) using BSA as the standard.

In the end, dual digestion was conducted on 150 µg of each protein sample, first with Lys-C (Wako, Japan) at a 1:100 enzyme:protein ratio overnight at RT, followed by trypsin (Promega, Madison, WI, USA) digestion at a 1:100 enzyme:protein ratio for at least 4 h at 37°C. The reactions were stopped using trifluoroacetic acid (TFA) to a final concentration of 1% (pH 2 to 3). Peptide yields were desalted by SDB-RPS (3M-Empore) Stage Tips and the eluted peptides were dried by vacuum centrifuge, then the dried peptides were reconstituted in 200 µl of 200 mM HEPES (pH 8). Next, the peptide concentration was measured using the Micro BCA™ Protein Assay Kit (Thermo Scientific, Rockford, IL, USA) and 70 µg from each sample was aliquoted for labeling in a 10-plex TMT reaction [158].

Tandem mass tag (TMT) labeling. To accommodate all the 3 biological replicates of 6 differentiation stages, 3 TMT experiments were carried out; each contained one technical inner control (D0) and a single biological replicate of each stage. The remaining channels in each experiment were filled with other OL lineage peptide samples not utilized in this

study (in Figure 1 you can see the samples applied in this study). The TMT labeling was carried out as previously described [158–160]; we added 41 µl of anhydrous acetonitrile to each 0.8 mg label vial, followed by occasional vortexing for 5 min and brief centrifugation. Ten TMT labels (Thermo, San Jose, CA, USA) were added to the 10 individual protein samples in each experiment. Labeling was performed for 1 h at RT with occasional vortexing. To quench any remaining TMT reagent and reverse the tyrosine labeling, 8 µl of 5% hydroxylamine (Sigma-Aldrich) was added to each tube, followed by vortexing and incubation at RT for 15 min. For each of the respective 10-plex experiments, all 10 labeled samples were combined in a clean 2 ml Eppendorf tube and then dried via speed vacuum centrifugation. The dried peptide mixture was reconstituted in 1% formic acid (FA, pH approximately 2–3) and desalted on a 130 mg C18 Sep-Pak (Waters, Milford, MA, USA) as previously described [161], and eventually dried down again using speed vacuum centrifuge.

Fractionation. To reduce the complexity of the mixture, offline strong cation-exchange (SCX) fractionation was carried out for each of the TMT experiments. The labeled peptides were resuspended in strong cation exchange buffer A (5 mM KH_2PO_4 and 25% v/v acetonitrile [ACN], pH 2.72), and were injected onto a PolySULFOETHYL A™ column (200 mm × 2.1 mm, 5 µm, 200 Å; PolyLC, Columbia, MD, USA), which was also equilibrated with buffer A. The adsorbed peptides were eluted with a linear gradient of 10–45% buffer B (5 mM KH_2PO_4, pH 2.72, 350 mM KCl, 25% ACN) for 70 min, which was then rapidly increased to 100% buffer B for 10 min at a flow rate of 300 µl/min. The collected samples were then desalted using SDB-RPS Stage Tips, dried by vacuum centrifuge, and reconstituted in 40 µl of 0.1% FA in preparation for nanoflow Liquid Chromatography Electrospray Ionization Tandem Mass Spectrometry (nanoflow LC-ESI-MS/MS) as reported by Mirzaei *et al.* [158].

Nano LC-ESI-MS/MS of tandem mass tag (TMT)-labelled peptides. Fractionated peptide samples were analysed on a Q Exactive Orbitrap mass spectrometer (Thermo Scientific, San Jose, CA, USA) coupled to an EASY-nLC1000 nanoflow HPLC system (Thermo Scientific, San Jose, CA, USA) as described previously [161]. The peptides were separated on an in-house packed reverse-phase column (75 µm inner diameter × 100

mm, C18 HALO column, 2.7 μm bead size, 160 Å pore size, Advanced Materials Technology). For sample elution, fractionated labeled peptides were run for over 170 min on a linear gradient of 1–30% solvent B (99.9% ACN/0.1% FA). The Q Exactive mass spectrometer was operated in the data-dependent acquisition (DDA) mode to automatically switch between full Orbitrap MS and ion trap MS/MS acquisition. Survey full-scan MS spectra (from m/z 350–1850) were received at a precursor isolation width of 0.7m/z, resolution of 70000 at m/z 400 and an Automatic Gain Control (AGC) target value of 1×10^6 ions. For identification of the TMT labeled peptides, the 10 most abundant ions were selected for higher energy collisional dissociation (HCD) fragmentation. HCD normalized collision energy (NCE) was set to 35% and fragmentation ions were detected in the Orbitrap at a resolution of 70000. Dynamic exclusion of target ions (selected for MS/MS) was set to 90 s and for accurate mass measurement, the lock mass option was enabled using the polydimethylcyclosiloxane ion (*m*/*z* 445.12003) as the internal calibrant [162].

Data processing and protein identification. MS raw data were generated by Xcalibur software (Thermo Scientific) and processed with Proteome Discoverer V1.3 (Thermo Scientific, San Jose, CA, USA). Peptide identification was performed using a local MASCOT server V2.3 (Matrix Science, London, UK). The MS/MS spectra were searched against the reviewed UniProt *Homo sapiens* protein database (20,352 sequences-August 2018)[10]. The following parameters and adjustments were used: MS^1 tolerance was set to ±10 ppm precursor. A limit of 0.1 Da was applied for MS/MS fragment ion tolerance and trypsin was set as cleavage specificity that allowed only one missed cleavage. Carbamidomethylation of cysteine was set as a fixed modification and TMT modification of peptide N-termini plus lysine residues, oxidation of methionine and deamidation of Asn and Gln residues were set as variable modifications. For deconvolution of the high-resolution MS^2 spectra, only peptides with a score >15 and below the Mascot significance threshold filter of $P = 0.05$ were included in the search results. Single peptide identifications required a score equal to or above the Mascot identity threshold to be incorporated in the search results. Protein grouping was enabled such that when a set of peptides in one protein were equal to, or completely contained within the set of peptides

of another protein, the two proteins were confined in one protein group. Proteins with at least two unique peptides were regarded as confident identifications. Hence, search results were further filtered to retain proteins that had q values (FDR) of <1% and only master proteins assigned via the protein grouping algorithm were retained. Relative quantitation of proteins was achieved by pairwise comparison of TMT reporter ion signal to noise (S/N) ratios as the ratio S/N of the labels for each of the differentiation time points versus the labels of the internal standard (reference) of that run.

TMT data analysis. All protein ratios with respect to the reference (label-126) were extracted and a limit of 1.5 was set as the cutoff for differential expression. No limit was set for the number of counts per peptide; however, 100 × log2(ratio)/variability was set as the protein z-score. All downstream data analysis including Pearson correlation coefficients (PCCs), Principal component analysis (PCA), c-means unsupervised fuzzy clustering and visualization of the results were implemented using custom scripts in R statistical computing environment [163]. PCA was performed using the mean-subtracted expression values. Protein classification in Figure S2C was performed using the PANTHER classification system (http://pantherdb.org/) [13]. Biological DataBase network (bioDBnet) was used for conversion of accession IDs into HGNC Symbols (protein names) (https://biodbnet-abcc.ncifcrf.gov/db/db2db.php) [164]. Normalization and differentially expression analysis were performed using the R/Bioconductor package limma version 3.34.9 [165]. Proteins having absolute log2 fold-change higher than 1 and Benjamini-Hochberg adjusted p-value less than 0.05 were considered as significant differentially expressed proteins (DEPs) in all comparisons. Gene Set Enrichment Analysis (GSEA) was performed using GSEA 4.0.3 [166] with weighted enrichment statistics. The number of permutations was set to 1000. We used gene_set as the permutation type and Signal2Noise metric for ranking genes. The minimum and maximum sizes of the sets were adjusted to 15 and 500, respectively. Wnt signaling components for GSEA analysis were obtained from Molecular Signatures Database (MSigDB) [167]. Gene ontology (GO) and pathway analysis were performed using Enrichr (https://amp.pharm.mssm.edu/Enrichr/) [41,42]. Single protein indexed GOs were achieved by UniProt (https://www.uniprot.org/, based on reviewed indexed data) [40],

neXtProt (https://www.nextprot.org/) [93], and David gene annotation tool v6.8 (https://david.ncifcrf.gov/) [39] databases.

Competing interests

The authors declare that they have no conflict of interests.

Funding

This work was supported by a grant from Royan Institute. Aspects of this research, including the efforts of M. Mirzaei, have been enabled by access to the Australian Proteome Analysis Facility supported under the Australian Government's National Collaborative Research Infrastructure Strategy (NCRIS).

Author contributions

G. H. S., P. P., and M. J. conceived the project, designed the study and interpreted results with the efforts of R. K. and M. M. and H. B.

P. P. performed the experiments, except for the TMT experiment which was performed by M. M., Y. W., and A. A., and V. G.

A. M. contributed in sample preparation.

P. P. and R. K. performed computational analyses and prepared figures.

P. P. wrote the manuscript with input from the co-authors. G. H. S. and M. M., and H. B. oversaw all aspects of the study. All authors proofread the paper and approved the final version.

Supplementary data

Supplemental Information includes five figures and five tables and accompanies this article online at *GIGSCI*.

Acknowledgements

We would like to thank the RSCB team for sharing the RH6 (hESC) line. We thank H. Ansari and A. Meyfour for their assistance in RH6 expansion and express our appreciation to R. Moghimi, F. S. Samani, F. Yekani, S. Nemati, S. Mirshahvaladi, P. Parsamatin, M. Alikhani, A. Samadian, M. Azimi, M. Dorraj, and N. Pirhadi for laboratory management and support. We wish to acknowledge S. M. Hosseini for providing antibodies, E. Shahbazi for his help in immunostaining of the OLIG2$^+$ and MBP$^+$ cells, F. Moeinvaziri for her help in immunostaining of the NKX2.2$^+$ cells, M. Saberian for unsupervised data imputation, and A. Sharifi-Zarchi for technical supports. We also would like to thank S. Simorgh, A. M. Khanghahi, and F. Shekari for their discussions. The authors would like to thank Iran's National Elites Foundation (INEF) for its support.

References

1. Baumann N, Pham-Dinh D. Biology of oligodendrocyte and myelin in the mammalian central nervous system. Physiol Rev. 2001;81:871–927.

2. Franklin RJM, Goldman SA. Glia disease and repair—Remyelination. Cold Spring Harb Perspect Biol. 2015;7:1–28.

3. Abu-Rub M, Miller RH. Emerging cellular and molecular strategies for enhancing central nervous system (CNS) remyelination. Brain Sci. 2018;8.

4. Zhu Z, Huangfu D. Human pluripotent stem cells: An emerging model in developmental biology. Dev [Internet]. 2013;140:705–17. Available from: http://dev.biologists.org/cgi/doi/10.1242/dev.086165

5. Chen X, He Y, Lu F. Autophagy in stem cell biology: A perspective on stem cell self-renewal and differentiation. Stem Cells Int. Hindawi; 2018;2018.

6. Douvaras P, Wang J, Zimmer M, Hanchuk S, O'Bara MA, Sadiq S, et al. Efficient generation of myelinating oligodendrocytes from primary progressive multiple sclerosis patients by induced pluripotent stem cells. Stem Cell Reports [Internet]. The Authors; 2014;3:250–9. Available from: http://dx.doi.org/10.1016/j.stemcr.2014.06.012

7. Douvaras P, Fossati V. Generation and isolation of oligodendrocyte progenitor cells from human pluripotent stem cells. Nat Protoc [Internet]. 2015;10:1143–54. Available from: http://dx.doi.org/10.1038/nprot.2015.075

8. Baharvand H, Ashtiani SK, Taee A, Massumi M, Valojerdi MR, Yazdi PE, et al. Generation of new human embryonic stem cell lines with diploid and triploid karyotypes. Dev Growth Differ. 2006;48:117–28.

9. Douvaras P, Rusielewicz T, Kim KH, Haines JD, Casaccia P, Fossati V. Epigenetic modulation of human induced pluripotent stem cell differentiation to oligodendrocytes. Int J Mol Sci. 2016;17.

10. UniProt Consortium T. Erratum: UniProt: the universal protein knowledgebase (Nucleic acids research (2017) 45 D1 (D158-D169)). Nucleic Acids Res. Oxford University Press; 2018;46:2699.

11. Deutsch EW, Csordas A, Sun Z, Jarnuczak A, Perez-Riverol Y, Ternent T, et al. The ProteomeXchange consortium in 2017: Supporting the cultural change in proteomics public data deposition. Nucleic Acids Res. 2017;45:D1100–6.

12. Wei R, Wang J, Su M, Jia E, Chen S, Chen T, et al. Missing Value Imputation Approach for Mass Spectrometry-based Metabolomics Data. Sci Rep [Internet]. Springer US; 2018;8:1–10. Available from: http://dx.doi.org/10.1038/s41598-017-19120-0

13. Mi H, Muruganujan A, Huang X, Ebert D, Mills C, Guo X, et al. Protocol Update for large-scale genome and gene function analysis with the PANTHER classification system (v.14.0). Nat Protoc [Internet]. Springer US; 2019;14:703–21. Available from: http://dx.doi.org/10.1038/s41596-019-0128-8

14. Vogel A, Upadhya R, Shetty AK. Neural stem cell derived extracellular vesicles: Attributes and prospects for treating neurodegenerative disorders. EBioMedicine [Internet]. Elsevier B.V.; 2018;38:273–82. Available from: https://doi.org/10.1016/j.ebiom.2018.11.026

15. Horgusluoglu E, Nudelman K, Nho K, Saykin AJ. Adult neurogenesis and neurodegenerative diseases: A systems biology perspective. Am. J. Med. Genet. Part B

Neuropsychiatr. Genet. 2017. p. 93–112.

16. Annenkov A. Receptor tyrosine kinase (RTK) signalling in the control of neural stem and progenitor cell (NSPC) development. Mol Neurobiol. 2014;49:440–71.

17. Knobloch M. The Role of Lipid Metabolism for Neural Stem Cell Regulation. Brain Plast. 2017;3:61–71.

18. Kim DY, Rhee I, Paik J. Metabolic circuits in neural stem cells. Cell Mol Life Sci. 2014;71:4221–41.

19. Soliven B. Calcium Signalling in Cells of Oligodendroglial Lineage. 2001;679:672–9.

20. Haak LL, Grimaldi M, Russell JT. Mitochondria in myelinating cells : calcium signaling in oligodendrocyte precursor cells. 2000;28:297–306.

21. Butt AM. Neurotransmitter-Mediated Calcium Signalling in Oligodendrocyte Physiology and Pathology. 2006;675:666–75.

22. Li T, Wang L, Ma T, Wang S, Niu J, Li H, et al. Dynamic Calcium Release From Endoplasmic Reticulum Mediated by Ryanodine Receptor 3 Is Crucial for Oligodendroglial Differentiation. 2018;11:1–11.

23. Bacon C, Lakics V, Machesky L, Rumsby M. N-WASP Regulates Extension of Filopodia and Processes by Oligodendrocyte Progenitors , Oligodendrocytes , and Schwann Cells — Implications for Axon Ensheathment at Myelination. 2007;858:844–58.

24. Boullerne AI, Benjamins JA. Nitric oxide synthase expression and nitric oxide toxicity in oligodendrocytes. Antioxidants Redox Signal. 2006. p. 967–80.

25. Tager HS, Steiner DF. Peptide hormones. Annu Rev Biochem. 1974;43:509–38.

26. Chrast R, Saher G, Nave K-A, Verheijen MHG. Lipid metabolism in myelinating glial cells: lessons from human inherited disorders and mouse models. J Lipid Res. 2011;52:419–34.

27. Yu J, Vodyanik MA, Smuga-Otto K, Antosiewicz-Bourget J, Frane JL, Tian S, et al. Induced pluripotent stem cell lines derived from human somatic cells. Science (80-). 2007;318:1917–20.

28. Cimadamore F, Amador-Arjona A, Chen C, Huang CT, Terskikh A V. SOX2-LIN28/let-7 pathway regulates proliferation and neurogenesis in neural precursors. Proc Natl Acad Sci U S A. 2013;110:E3017–26.

29. Goldman SA, Kuypers NJ. How to make an oligodendrocyte. Development. 2015;142:3983–95.

30. Almeida RG. The Rules of Attraction in Central Nervous System Myelination. Front Cell Neurosci. 2018;12:1–9.

31. Schnädelbach O, Blaschuk OW, Symonds M, Gour BJ, Doherty P, Fawcett JW. N-Cadherin influences migration of oligodendrocytes on astrocyte monolayers. Mol Cell Neurosci. 2000;15:288–302.

32. Ji S, Doucette JR, Nazarali AJ. Sirt2 is a novel in vivo downstream target of Nkx2.2 and enhances oligodendroglial cell differentiation. J Mol Cell Biol. 2011;3:351–9.

33. Lamprianou S, Chatzopoulou E, Thomas J-L, Bouyain S, Harroch S. A complex between contactin-1 and the protein tyrosine phosphatase PTPRZ controls the development of oligodendrocyte precursor cells. Proc Natl Acad Sci. 2011;108:17498–503.

34. Shao Z, Lee X, Huang G, Sheng G, Henderson CE, Louvard D, et al. LINGO-1 Regulates Oligodendrocyte Differentiation through the Cytoplasmic Gelsolin Signaling Pathway. J Neurosci. 2017;37:3127–37.

35. Jahn O, Tenzer S, Werner HB. Myelin proteomics: Molecular anatomy of an insulating sheath. Mol Neurobiol. 2009;40:55–72.

36. Barresi MJF, Gilbert SF. Developmental Biology, Twelfth Edition [Internet]. Twelfth Ed. New York: Oxford University Press; 2019. Available from: http://www.isbnsearch.org/isbn/9781605358246

37. Veeman MT, Axelrod JD, Moon RT. A Second Canon: Review Functions and Mechanismsof. 2003;5:1–11.

38. Marinou K, Christodoulides C, Antoniades C, Koutsilieris M. Wnt signaling in cardiovascular physiology. Trends Endocrinol Metab [Internet]. Elsevier Ltd; 2012;23:628–36. Available from: http://dx.doi.org/10.1016/j.tem.2012.06.001

39. Huang DW, Sherman BT, Lempicki RA. Systematic and integrative analysis of large gene lists using DAVID bioinformatics resources. Nat Protoc. 2009;4:44–57.

40. Bateman A. UniProt: A worldwide hub of protein knowledge. Nucleic Acids Res. Oxford University Press; 2019;47:D506–15.

41. Kuleshov M V., Jones MR, Rouillard AD, Fernandez NF, Duan Q, Wang Z, et al. Enrichr: a comprehensive gene set enrichment analysis web server 2016 update. Nucleic Acids Res. 2016;44:W90–7.

42. Chen EY, Tan CM, Kou Y, Duan Q, Wang Z, Meirelles GV, et al. Enrichr: interactive and collaborative HTML5 gene list enrichment analysis tool. BMC Bioinformatics. 2013;14:128–42.

43. Self-renewal SC, Merrill BJ. Wnt Pathway Regulation of Embryonic. 2016;1–18.

44. Fancy SPJ, Baranzini SE, Zhao C, Yuk DI, Irvine KA, Kaing S, et al. Dysregulation of the Wnt pathway inhibits timely myelination and remyelination in the mammalian CNS. Genes Dev. 2009;23:1571–85.

45. Dai Z-M, Sun S, Wang C, Huang H, Hu X, Zhang Z, et al. Stage-Specific Regulation of Oligodendrocyte Development by Wnt/ -Catenin Signaling. J Neurosci. 2014;34:8467–73.

46. Sato N, Meijer L, Skaltsounis L, Greengard P, Brivanlou AH. Maintenance of pluripotency in human and mouse embryonic stem cells through activation of Wnt signaling by a pharmacological GSK-3-specific inhibitor. Nat Med. 2004;10:55–63.

47. Singla DK, Schneider DJ, LeWinter MM, Sobel BE. Wnt3a But Not Wnt11 Supports Self-Renewal of Embryonic Stem Cells. Biochem Biophys Res Commun. 2006;345:789–95.

48. Sokol SY. Maintaining embryonic stem cell pluripotency with Wnt signaling. Development. 2011;138:4341–50.

49. Guo F, Lang J, Sohn J, Hammond E, Chang M, Pleasure D. Canonical Wnt signaling in the oligodendroglial lineage-puzzles remain. Glia. 2015;63:1671–93.

50. Soomro SH, Jie J, Fu H. Oligodendrocytes Development and Wnt Signaling

Pathway. Int J Hum Anat. 2018;1:17–35.

51. Albors AR, Tazaki A, Rost F, Nowoshilow S, Chara O, Tanaka EM. Planar cell polarity-mediated induction of neural stem cell expansion during axolotl spinal cord regeneration. Elife. 2015;4:1–29.

52. Butler MT, Wallingford JB. Planar cell polarity in development and disease. Nat Rev Mol Cell Biol [Internet]. Nature Publishing Group; 2017;18:375–88. Available from: http://dx.doi.org/10.1038/nrm.2017.11

53. Hayes MN, McCarthy K, Jin A, Oliveira ML, Iyer S, Garcia SP, et al. Vangl2/RhoA Signaling Pathway Regulates Stem Cell Self-Renewal Programs and Growth in Rhabdomyosarcoma. Cell Stem Cell [Internet]. Elsevier Inc.; 2018;22:414-427.e6. Available from: https://doi.org/10.1016/j.stem.2018.02.002

54. Mizushima N, Levine B. Autophagy in mammalian development and differentiation. Nat Cell Biol [Internet]. Nature Publishing Group; 2010;12:823–30. Available from: http://dx.doi.org/10.1038/ncb0910-823

55. Yue Z, Jin S, Yang C, Levine AJ, Heintz N. Beclin 1, an autophagy gene essential for early embryonic development, is a haploinsufficient tumor suppressor. Proc Natl Acad Sci U S A. 2003;100:15077–82.

56. Levine B, Kroemer G. Autophagy in the Pathogenesis of Disease. Cell. 2008;132:27–42.

57. Huang DW, Sherman BT, Lempicki RA. Bioinformatics enrichment tools: Paths toward the comprehensive functional analysis of large gene lists. Nucleic Acids Res. 2009;37:1–13.

58. Hansen M, Rubinsztein DC, Walker DW. Autophagy as a promoter of longevity: insights from model organisms. Nat Rev Mol Cell Biol [Internet]. Springer US; 2018;19:579–93. Available from: http://dx.doi.org/10.1038/s41580-018-0033-y

59. Behrends C, Sowa ME, Gygi SP, Harper JW. Network organization of the human autophagy system. Nature [Internet]. Nature Publishing Group; 2010;466:68–76. Available from: http://dx.doi.org/10.1038/nature09204

60. Smith CM, Mayer JA, Duncan ID. Autophagy Promotes Oligodendrocyte Survival

and Function following Dysmyelination in a Long-Lived Myelin Mutant. J Neurosci. 2013;33:8088–100.

61. Bankston AN, Forston MD, Howard RM, Andres KR, Smith AE, Ohri SS, et al. Autophagy is essential for oligodendrocyte differentiation, survival, and proper myelination. Glia. 2019;67:1745–59.

62. Vaughn CB, Jakimovski D, Kavak KS, Ramanathan M, Benedict RHB, Zivadinov R, et al. Epidemiology and treatment of multiple sclerosis in elderly populations. Nat Rev Neurol [Internet]. Springer US; 2019;15:329–42. Available from: http://dx.doi.org/10.1038/s41582-019-0183-3

63. Alirezaei M, Fox HS, Flynn CT, Moore CS, Hebb ALO, Frausto RF, et al. Elevated ATG5 expression in autoimmune demyelination and multiple sclerosis. Autophagy. Autophagy. 2009;5:152–8.

64. Igci M, Baysan M, Yigiter R, Ulasli M, Geyik S, Bayraktar R, et al. Gene expression profiles of autophagy-related genes in multiple sclerosis. Gene. 2016;588:38–46.

65. Rangaraju S, Verrier JD, Madorsky I, Nicks J, Dunn WA, Notterpek L. Rapamycin activates autophagy and improves myelination in explant cultures from neuropathic mice. J Neurosci. 2010;30:11388–97.

66. Meikle L, Pollizzi K, Egnor A, Kramvis I, Lane H, Sahin M, et al. Response of a neuronal model of tuberous sclerosis to mammalian target of rapamycin (mTOR) inhibitors: Effects on mTORC1 and Akt signaling lead to improved survival and function. J Neurosci. 2008;28:5422–32.

67. Liang P, Le W. Role of autophagy in the pathogenesis of multiple sclerosis. Neurosci Bull. 2015;31:435–44.

68. Slamecka J, McClellan S, Wilk A, Laurini J, Manci E, Hoerstrup SP, et al. Induced pluripotent stem cells derived from human amnion in chemically defined conditions. Cell Cycle. 2018. p. 330–47.

69. Loboda A, Damulewicz M, Pyza E, Jozkowicz A, Dulak J. Role of Nrf2/HO-1 system in development, oxidative stress response and diseases: an evolutionarily conserved mechanism. Cell Mol Life Sci. Springer International Publishing; 2016;73:3221–47.

70. Lomelino CL, Andring JT, McKenna R, Kilberg MS. Asparagine synthetase: Function, structure, and role in disease. J Biol Chem. 2017;292:19952–8.

71. Lin CY, Peng CY, Huang TT, Wu ML, Lai YL, Peng DH, et al. Exacerbation of oxidative stress-induced cell death and differentiation in induced pluripotent stem cells lacking heme oxygenase-1. Stem Cells Dev. 2012. p. 1675–87.

72. Stepniewski J, Kachamakova-Trojanowska N, Ogrocki D, Szopa M, Matlok M, Beilharz M, et al. Induced pluripotent stem cells as a model for diabetes investigation. Sci Rep. 2015;5:1–14.

73. Stepniewski J, Pacholczak T, Skrzypczyk A, Ciesla M, Szade A, Szade K, et al. Heme oxygenase-1 affects generation and spontaneous cardiac differentiation of induced pluripotent stem cells. IUBMB Life. 2018. p. 129–42.

74. Phanstiel DH, Brumbaugh J, Wenger CD, Tian S, Probasco MD, Bailey DJ, et al. Proteomic and phosphoproteomic comparison of human ES and iPS cells. Nat Methods. 2011;8:821–7.

75. Kempf H, Olmer R, Kropp C, Rückert M, Jara-Avaca M, Robles-Diaz D, et al. Controlling expansion and cardiomyogenic differentiation of human pluripotent stem cells in scalable suspension culture. Stem Cell Reports. 2014;3:1132–46.

76. Choi J, Lee S, Mallard W, Clement K, Tagliazucchi GM, Lim H, et al. A comparison of genetically matched cell lines reveals the equivalence of human iPSCs and ESCs. Nat Biotechnol [Internet]. Nature Publishing Group; 2015;33:1173–81. Available from: http://dx.doi.org/10.1038/nbt.3388

77. Krall AS, Xu S, Graeber TG, Braas D, Christofk HR. Asparagine promotes cancer cell proliferation through use as an amino acid exchange factor. Nat Commun [Internet]. Nature Publishing Group; 2016;7:1–13. Available from: http://dx.doi.org/10.1038/ncomms11457

78. Vermeulen T, Görg B, Vogl T, Wolf M, Varga G, Toutain A, et al. Glutamine synthetase is essential for proliferation of fetal skin fibroblasts. Arch Biochem Biophys. 2008;478:96–102.

79. Carey BW, Finley LWS, Cross JR, Allis CD, Thompson CB. Intracellular α-

ketoglutarate maintains the pluripotency of embryonic stem cells. Nature [Internet]. Nature Publishing Group; 2015;518:413–6. Available from: http://dx.doi.org/10.1038/nature13981

80. Shparberg R, Glover H, Morris MB. Modelling mammalian commitment to the neural lineage using embryos and embryonic stem cells. Front Physiol. 2019;10.

81. Ryu JM, Lee SH, Seong JK, Han HJ. Glutamine contributes to maintenance of mouse embryonic stem cell self-renewal through PKC-dependent downregulation of HDAC1 and DNMT1/3a. Cell Cycle. 2015;14:3292–305.

82. Xiao D, Liu X, Zhang M, Zou M, Deng Q, Sun D, et al. Direct reprogramming of fibroblasts into neural stem cells by single non-neural progenitor transcription factor Ptf1a. Nat Commun [Internet]. Springer US; 2018;9. Available from: http://dx.doi.org/10.1038/s41467-018-05209-1

83. Atlasi Y, Stunnenberg HG. The interplay of epigenetic marks during stem cell differentiation and development. Nat Rev Genet [Internet]. Nature Publishing Group; 2017;18:643–58. Available from: http://dx.doi.org/10.1038/nrg.2017.57

84. Chan SF, Huang X, McKercher SR, Zaidi R, Okamoto SI, Nakanishi N, et al. Transcriptional profiling of MEF2-regulated genes in human neural progenitor cells derived from embryonic stem cells. Genomics Data [Internet]. The Authors; 2015;3:24–7. Available from: http://dx.doi.org/10.1016/j.gdata.2014.10.022

85. Lee MWY, Chang AC, Sun DS, Hsu CY, Chang NCA. Restricted expression of LUZP in neural lineage cells: A study in embryonic stem cells. J Biomed Sci. 2001;8:504–11.

86. Sima N, Li R, Huang W, Xu M, Beers J, Zou J, et al. Neural stem cells for disease modeling and evaluation of therapeutics for infantile (CLN1/PPT1) and late infantile (CLN2/TPP1) neuronal ceroid lipofuscinoses. Orphanet J Rare Dis [Internet]. Orphanet Journal of Rare Diseases; 2018;13:54. Available from: https://ojrd.biomedcentral.com/articles/10.1186/s13023-018-0798-2

87. Liu B, Li Y, Stackpole EE, Novak A, Gao Y, Zhao Y, et al. Regulatory discrimination of mRNAs by FMRP controls mouse adult neural stem cell differentiation. Proc Natl

Acad Sci U S A. 2018;115:E11397–405.

88. Wang J, Cheng H, Li X, Lu W, Wang K, Wen T. Regulation of neural stem cell differentiation by transcription factors HNF4-1 and MAZ-1. Mol Neurobiol. 2013;47:228–40.

89. Liu B, Ma A, Zhang F, Wang Y, Li Z, Li Q, et al. MAZ mediates the cross-talk between CT-1 and NOTCH1 signaling during gliogenesis. Sci Rep [Internet]. Nature Publishing Group; 2016;6:1–12. Available from: http://dx.doi.org/10.1038/srep21534

90. Najm FJ, Lager AM, Zaremba A, Wyatt K, Caprariello A V., Factor DC, et al. Transcription factor-mediated reprogramming of fibroblasts to expandable, myelinogenic oligodendrocyte progenitor cells. Nat Biotechnol [Internet]. Nature Publishing Group; 2013;31:426–33. Available from: http://dx.doi.org/10.1038/nbt.2561

91. Thakurela S, Tiwari N, Schick S, Garding A, Ivanek R, Berninger B, et al. Mapping gene regulatory circuitry of Pax6 during neurogenesis. Cell Discov [Internet]. Nature Publishing Group; 2016;2. Available from: http://dx.doi.org/10.1038/celldisc.2015.45

92. Andersen J. Study of Ascl1 function in the neurogenic lineage of the adult mouse hippocampus. Dr thesis, UCL (University Coll London) [Internet]. 2015;1–212. Available from: http://discovery.ucl.ac.uk/1469969/

93. Gaudet P, Michel PA, Zahn-Zabal M, Britan A, Cusin I, Domagalski M, et al. The neXtProt knowledgebase on human proteins: 2017 update. Nucleic Acids Res. 2017;45:D177–82.

94. Timmer JR, Mak TW, Manova K, Anderson K V., Niswander L. Tissue morphogenesis and vascular stability require the Frem2 protein, product of the mouse myelencephalic blebs gene. Proc Natl Acad Sci U S A. 2005;102:11746–50.

95. Beaumont M, Akloul L, Carré W, Quélin C, Journel H, Pasquier L, et al. Targeted panel sequencing establishes the implication of planar cell polarity pathway and involves new candidate genes in neural tube defect disorders. Hum Genet [Internet]. Springer Berlin Heidelberg; 2019;138:363–74. Available from: http://dx.doi.org/10.1007/s00439-019-01993-y

96. cortecon.neuralsci.org/index.php?cort_mode=genedisplay&geneid=3728 [Internet].

[cited 2019 Aug 30]. Available from: cortecon.neuralsci.org/index.php?cort_mode=genedisplay&geneid=3728

97. Angst BD, Marcozzi C, Magee AI. The cadherin superfamily: Diversity in form and function. J Cell Sci. 2001;114:629–41.

98. Yang L, Jiang Z, Zhou L, Zhao K, Ma X, Cheng G. Hydrophilic cell-derived extracellular matrix as a niche to promote adhesion and differentiation of neural progenitor cells. RSC Adv. Royal Society of Chemistry; 2017;7:45587–94.

99. Jiao Q, Li X, An J, Zhang Z, Chen X, Tan J, et al. Cell-cell connection enhances proliferation and neuronal differentiation of rat embryonic neural stem/progenitor cells. Front Cell Neurosci. 2017;11:1–11.

100. Kim S young, Kelland EE, Kim J hong, Lund BT, Chang X, Wang K, et al. The influence of retinoic acid on the human oligodendrocyte precursor cells by RNA-sequencing. Biochem Biophys Reports [Internet]. Elsevier B.V.; 2017;9:166–72. Available from: http://dx.doi.org/10.1016/j.bbrep.2016.12.004

101. Zhang YW, Denham J, Thies RS. Oligodendrocyte progenitor cells derived from human embryonic stem cells express neurotrophic factors. Stem Cells Dev. 2006;15:943–52.

102. Chou AP, Chowdhury R, Li S, Chen W, Kim AJ, Piccioni DE, et al. Identification of retinol binding protein 1 promoter hypermethylation in isocitrate dehydrogenase 1 and 2 mutant gliomas. J Natl Cancer Inst. 2012;104:1458–69.

103. Singec I, Crain AM, Hou J, Tobe BTD, Talantova M, Winquist AA, et al. Quantitative Analysis of Human Pluripotency and Neural Specification by In-Depth (Phospho)Proteomic Profiling. Stem Cell Reports [Internet]. The Authors; 2016;7:527–42. Available from: http://dx.doi.org/10.1016/j.stemcr.2016.07.019

104. Hoffmann SA, Hos D, Küspert M, Lang RA, Lovell-Badge R, Wegner M, et al. Stem cell factor Sox2 and its close relative Sox3 have differentiation functions in oligodendrocytes. Dev. 2014;141:39–50.

105. Xiao ML, Liu JQ, Chen C. Effect of tumor necrosis factor-related apoptosis-inducing ligand on developing human oligodendrocytes in culture. Mol Biol.

2014;48:845–51.

106. Chaerkady R, Letzen B, Renuse S, Sahasrabuddhe NA, Kumar P, All AH, et al. Quantitative temporal proteomic analysis of human embryonic stem cell differentiation into oligodendrocyte progenitor cells. Proteomics. 2011;11:4007–20.

107. Stoffels JMJ, Hoekstra D, Franklin RJM, Baron W, Zhao C. The EIIIA domain from astrocyte-derived fibronectin mediates proliferation of oligodendrocyte progenitor cells following CNS demyelination. Glia. 2015;63:242–56.

108. Domingues HS, Portugal CC, Socodato R, Relvas JB. Oligodendrocyte, astrocyte, and microglia crosstalk in myelin development, damage, and repair. Front Cell Dev Biol. 2016;4:1–16.

109. Šišková Z, Yong VW, Nomden A, van Strien M, Hoekstra D, Baron W. Fibronectin attenuates process outgrowth in oligodendrocytes by mislocalizing MMP-9 activity. Mol Cell Neurosci. 2009;42:234–42.

110. Stoffels JMJ, De Jonge JC, Stancic M, Nomden A, Van Strien ME, Ma D, et al. Fibronectin aggregation in multiple sclerosis lesions impairs remyelination. Brain. 2013;136:116–31.

111. Tripathi A, Parikh ZS, Vora P, Frost EE, Pillai PP. pERK1/2 Peripheral Recruitment and Filopodia Protrusion Augment Oligodendrocyte Progenitor Cell Migration: Combined Effects of PDGF-A and Fibronectin. Cell Mol Neurobiol. Springer US; 2017;37:183–94.

112. Logan TT, Villapol S, Symes AJ. TGF-β Superfamily Gene Expression and Induction of the Runx1 Transcription Factor in Adult Neurogenic Regions after Brain Injury. PLoS One. 2013;8:1–14.

113. Czopka T, Von Holst A, Ffrench-Constant C, Faissner A. Regulatory mechanisms that mediate tenascin C-dependent inhibition of oligodendrocyte precursor differentiation. J Neurosci. 2010;30:12310–22.

114. Faissner A, Reinhard J. The extracellular matrix compartment of neural stem and glial progenitor cells. Glia. 2015;63:1330–49.

115. Najm FJ, Zaremba A, Caprariello A V., Nayak S, Freundt EC, Scacheri PC, et al.

Rapid and robust generation of functional oligodendrocyte progenitor cells from epiblast stem cells. Nat Methods. 2011;8:957–62.

116. Gao YF, Mao XY, Zhu T, Mao CX, Liu ZX, Wang Z Bin, et al. COL3A1 and SNAP91: Novel glioblastoma markers with diagnostic and prognostic value. Oncotarget. 2016;7:70494–503.

117. Curtis R, Hardy R, Reynolds R, Spruce BA, Wilkin GP. Down-regulation of GAP-43 During Oligodendrocyte Development and Lack of Expression by Astrocytes In Vivo: Implications for Macroglial Differentiation. Eur J Neurosci. 1991;3:876–86.

118. Hu ML, Zhu HM, Zhang QL, Liu JJ, Ding Y, Zhong JM, et al. Exploring the mechanisms of electroacupuncture-induced analgesia through RNA sequencing of the periaqueductal gray. Int. J. Mol. Sci. 2018.

119. Tsukushi S, Nishida Y, Sugiura H, Nakashima H, Ishiguro N. Soft tissue sarcomas of the chest wall. J Thorac Oncol [Internet]. International Association for the Study of Lung Cancer; 2009;4:834–7. Available from: http://dx.doi.org/10.1097/JTO.0b013e3181a97da3

120. Liu T, Zhao Y, Tang N, Feng R, Yang X, Lu N, et al. Pax6 Directly Down-Regulates Pcsk1n Expression Thereby Regulating PC1/3 Dependent Proinsulin Processing. PLoS One. 2012;7:1–11.

121. Marques S, Zeisel A, Codeluppi S, Van Bruggen D, Falcão AM, Xiao L, et al. Oligodendrocyte heterogeneity in the mouse juvenile and adult central nervous system. Science (80-.). 2016. p. 1326–9.

122. Hu JG, Wang YX, Zhou JS, Chen CJ, Wang FC, Li XW, et al. Differential gene expression in oligodendrocyte progenitor cells, oligodendrocytes and type II astrocytes. Tohoku J Exp Med. 2011;223:161–76.

123. Lager AM, Corradin OG, Cregg JM, Elitt MS, Shick HE, Clayton BLL, et al. Rapid functional genetics of the oligodendrocyte lineage using pluripotent stem cells. Nat Commun [Internet]. Springer US; 2018;9:1–8. Available from: http://dx.doi.org/10.1038/s41467-018-06102-7

124. Thangaraj MP, Furber KL, Gan JK, Ji S, Sobchishin L, Doucette JR, et al. RNA-

binding protein quaking stabilizes Sirt2 mRNA during oligodendroglial differentiation. J Biol Chem. 2017;292:5166–82.

125. Lehotzky A, Lau P, Tokési N, Muja N, Hudson LD, Ovádi J. Tubulin polymerization-promoting protein (TPPP/p25) is critical for oligodendrocyte differentiation. Glia. 2010;58:157–68.

126. McKinnon RD, Piras G, Ida JA, Dubois-Dalcq M. A role for TGF-β in oligodendrocyte differentiation. J Cell Biol. 1993;121:1397–408.

127. Gravel M, Peterson J, Yong VW, Kottis V, Trapp B, Braun PE. Overexpression of 2’,3’-cyclic nucleotide 3’-phosphodiesterase in transgenic mice alters oligodendrocyte development and produces aberrant myelination. Mol Cell Neurosci. 1996;7:453–66.

128. Miyamoto Y, Yamauchi J, Chan JR, Okada A, Tomooka Y, Hisanaga SI, et al. Cdk5 regulates differentiation of oligodendrocyte precursor cells through the direct phosphorylation of paxillin. J Cell Sci. 2007;120:4355–66.

129. Yu S, Levi L, Siegel R, Noy N. Retinoic acid induces neurogenesis by activating both retinoic acid receptors (RARs) and peroxisome proliferator-activated receptor β/δ (PPARβ/δ). J Biol Chem. 2012;287:42195–205.

130. Maussion G, Diallo AB, Gigek CO, Chen ES, Crapper L, Théroux JF, et al. Investigation of genes important in neurodevelopment disorders in adult human brain. Hum Genet. Springer Berlin Heidelberg; 2015;134:1037–53.

131. Lin JC, Tsai JT, Chao TY, Ma HI, Liu WH. Musashi-1 Enhances Glioblastoma Migration by Promoting ICAM1 Translation. Neoplasia (United States) [Internet]. The Authors; 2019;21:459–68. Available from: https://doi.org/10.1016/j.neo.2019.02.006

132. Kimura A, Matsuda T, Sakai A, Murao N, Nakashima K. HMGB2 expression is associated with transition from a quiescent to an activated state of adult neural stem cells. Dev Dyn. 2018;247:229–38.

133. Sun YM, Cooper M, Finch S, Lin HH, Chen ZF, Williams BP, et al. Rest-mediated regulation of extracellular matrix is crucial for neural development. PLoS One. 2008;3.

134. Pham JT, Gallicano GI. Specification of neural cell fate and regulation of neural stem cell proliferation by microRNAs. Am J Stem Cells. 2012;1:182–95.

135. Herberth B, Minkó K, Csillag A, Jaffredo T, Madarász E. SCL, GATA-2 and Lmo2 expression in neurogenesis. Int J Dev Neurosci. 2005;23:449–63.

136. de Souza Martins SC, Agbulut O, Diguet N, Larcher JC, Paulsen BS, Rehen SK, et al. Dynamic expression of synemin isoforms in mouse embryonic stem cells and neural derivatives. BMC Cell Biol. 2011;12.

137. Iwatsuki H, Suda M. Transient expression of keratin during neuronal development in the adult rabbit spinal ganglion. Anat Sci Int. 2010;85:46–55.

138. Kania G, Corbeil D, Fuchs J, Tarasov K V., Blyszczuk P, Huttner WB, et al. Somatic Stem Cell Marker Prominin-1/CD133 Is Expressed in Embryonic Stem Cell-Derived Progenitors. Stem Cells. 2005;23:791–804.

139. Lin J, Luo J, Redies C. Cadherin-19 expression is restricted to myelin-forming cells in the chicken embryo. Neuroscience [Internet]. Elsevier Inc.; 2010;165:168–78. Available from: http://dx.doi.org/10.1016/j.neuroscience.2009.10.032

140. Zhao L, Ku L, Chen Y, Xia M, LoPresti P, Feng Y. QKI Binds MAP1B mRNA and Enhances MAP1B Expression during Oligodendrocyte Development. Wickens MP, editor. Mol Biol Cell [Internet]. 2006;17:4179–86. Available from: http://www.ncbi.nlm.nih.gov/pubmed/1635361

141. Ricard D, Stankoff B, Bagnard D, Aguera M, Rogemond V, Antoine JC, et al. Differential expression of collapsin response mediator proteins (CRMP/Ulip) in subsets of oligodendrocytes in the postnatal rodent brain. Mol Cell Neurosci. 2000;16:324–37.

142. Ricard D, Rogemond V, Charrier E, Aguera M, Bagnard D, Belin MF, et al. Isolation and expression pattern of human Unc-33-like phosphoprotein 6/collapsin response mediator protein 5 (Ulip6/CRMP5): Coexistence with Ulip2/CRMP2 in Sema3A-sensitive oligodendrocytes. J Neurosci. 2001;21:7203–14.

143. Silvestroff L, Franco PG, Pasquini JM. Neural and Oligodendrocyte Progenitor Cells: Transferrin Effects on Cell Proliferation. ASN Neuro [Internet]. 2013;5:AN20120075. Available from: http://journals.sagepub.com/doi/10.1042/AN20120075

144. Deloulme JC, Laeng P, Janet T, Sensenbrenner M, Baudier J. Expression of

neuromodulin (GAP-43) and its regulation by basic fibroblast growth factor during the differentiation of O-2A progenitor cells. J Neurosci Res. 1993;36:147–62.

145. Deloulme JC, Janet T, Au D, Storm DR, Sensenbrenner M, Baudier J. Neuromodulin (GAP43): A neuronal protein kinase C substrate is also present in 0-2A glial cell lineage. Characterization of neuromodulin in secondary cultures of oligodendrocytes and comparison with the neuronal antigen. J Cell Biol. 1990;111:1559–69.

146. Gnegy ME. Calmodulin in neurotransmitter and hormone action. Annu Rev Pharmacol Toxicol. 1993;33:45–70.

147. Boczek T, Kozaczuk A, Ferenc B, Kosiorek M, Pikula S, Zylinska L. Gene expression pattern in PC12 cells with reduced PMCA2 or PMCA3 isoform: Selective up-regulation of calmodulin and neuromodulin. Mol Cell Biochem. 2012;360:89–102.

148. Boczek T, Ferenc B, Lisek M, Zylinska L. Regulation of GAP43/calmodulin complex formation via calcineurin-dependent mechanism in differentiated PC12 cells with altered PMCA isoforms composition. Mol Cell Biochem. Springer US; 2015;407:251–62.

149. Nicot A, Ratnakar P V., Ron Y, Chen CC, Elkabes S. Regulation of gene expression in experimental autoimmune encephalomyelitis indicates early neuronal dysfunction. Brain. 2003;126:398–412.

150. Bassil F, Canron MH, Vital A, Bezard E, Li Y, Greig NH, et al. Insulin resistance and exendin-4 treatment for multiple system atrophy. Brain. 2017;140:1420–36.

151. Chirivella L, Kirstein M, Ferrón SR, Domingo-Muelas A, Durupt FC, Acosta-Umanzor C, et al. Cyclin-Dependent Kinase 4 Regulates Adult Neural Stem Cell Proliferation and Differentiation in Response to Insulin. Stem Cells [Internet]. 2017;35:2403–16. Available from: http://doi.wiley.com/10.1002/stem.2694

152. Meireles AM, Shen K, Zoupi L, Iyer H, Bouchard EL, Williams A, et al. The Lysosomal Transcription Factor TFEB Represses Myelination Downstream of the Rag-Ragulator Complex. Dev Cell [Internet]. Elsevier Inc.; 2018;47:319-330.e5. Available from: https://doi.org/10.1016/j.devcel.2018.10.003

153. Werner HB, Krämer-Albers EM, Strenzke N, Saher G, Tenzer S, Ohno-Iwashita Y, et al. A critical role for the cholesterol-associated proteolipids PLP and M6B in myelination of the central nervous system. Glia. 2013;61:567–86.

154. Walko G, Wögenstein KL, Winter L, Fischer I, Feltri ML, Wiche G. Stabilization of the dystroglycan complex in Cajal bands of myelinating Schwann cells through plectin-mediated anchorage to vimentin filaments. Glia. 2013;61:1274–87.

155. Chambers SM, Fasano CA, Papapetrou EP, Tomishima M, Sadelain M, Studer L. Highly efficient neural conversion of human ES and iPS cells by dual inhibition of SMAD signaling. Nat Biotechnol. 2009;27:275–80.

156. Kruger NJ. The Bradford Method for Protein Quantitation.

157. Wessel D, Flügge UI. A method for the quantitative recovery of protein in dilute solution in the presence of detergents and lipids. Anal Biochem. 1984;138:141–3.

158. Mirzaei M, Pascovici D, Wu JX, Chick J, Wu Y, Cooke B, et al. TMT One-Stop Shop: From Reliable Sample Preparation to Computational Analysis Platform. 2017. p. 45–66. Available from: http://link.springer.com/10.1007/978-1-4939-6740-7_5

159. Deng L, Pushpitha K, Joseph C, Gupta V, Rajput R, Chitranshi N, et al. Amyloid β induces early changes in the ribosomal machinery, cytoskeletal organization and oxidative phosphorylation in retinal photoreceptor cells. Front Mol Neurosci. 2019;12:1–14.

160. Mirzaei M, Pushpitha K, Deng L, Chitranshi N, Gupta V, Rajput R, et al. Upregulation of Proteolytic Pathways and Altered Protein Biosynthesis Underlie Retinal Pathology in a Mouse Model of Alzheimer’s Disease. Mol Neurobiol. Molecular Neurobiology; 2019;56:6017–34.

161. Emery SJ, Mirzaei M, Vuong D, Pascovici D, Chick JM, Lacey E, et al. Induction of virulence factors in giardia duodenalis independent of host attachment. Sci Rep. Nature Publishing Group; 2016;6:1–16.

162. McAlister GC, Nusinow DP, Jedrychowski MP, Wühr M, Huttlin EL, Erickson BK, et al. MultiNotch MS3 enables accurate, sensitive, and multiplexed detection of differential expression across cancer cell line proteomes. Anal Chem. 2014;86:7150–8.

163. R Core Team. R: A language and environment for statistical computing. Vienna, Austria: R Foundation for Statistical Computing; 2018.

164. Mudunuri U, Che A, Yi M, Stephens RM. bioDBnet: The biological database network. Bioinformatics. 2009;25:555–6.

165. Phipson B, Lee S, Majewski IJ, Alexander WS, Smyth G. Robust Hyperparameter Estimation Protects. 2016;10:946–63.

166. Subramanian A, Tamayo P, Mootha VK, Mukherjee S, Ebert BL, Gillette MA, et al. Gene set enrichment analysis: A knowledge-based approach for interpreting genome-wide expression profiles. Proc Natl Acad Sci U S A. 2005;102:15545–50.

167. Liberzon A, Subramanian A, Pinchback R, Thorvaldsdóttir H, Tamayo P, Mesirov JP. Molecular signatures database (MSigDB) 3.0. Bioinformatics. 2011;27:1739–40.

168. Nevin ZS, Factor DC, Karl RT, Douvaras P, Laukka J, Windrem MS, et al. Modeling the Mutational and Phenotypic Landscapes of Pelizaeus-Merzbacher Disease with Human iPSC-Derived Oligodendrocytes. Am J Hum Genet [Internet]. ElsevierCompany.; 2017;100:617–34. Available from: http://dx.doi.org/10.1016/j.ajhg.2017.03.005

Figure 1: TMT labeling workflow for comprehensive proteome study of hESCs differentiation into the OL lineage. For in-depth quantitative proteomic profiling of the human OL lineage, the hESC line (Royan H6) first went through a stepwise differentiation process that resulted in the generation of NSCs, NPCs, pre-OPCs, OPCs, and OLs (in three replicates). Cellular samples were collected at six consecutive time points (d0, d8, d12, d20, d80, and d120). Their protein contents were extracted and denatured, reduced and alkylated, and then subjected to digestion with lysine-C besides trypsin. Peptides were next quantified and reacted with isobaric TMT reagents across individual batches per sample (there were three other OL lineage peptide samples not utilized in this study). After labeling, the samples were combined equally and ionized onto a mass spectrometer. Three TMT experiments were conducted to accommodate all the replicates. In the MS^1 spectrum, the peptides were detected as a single and identical precursor ion peak. Following fragmentation, in the MS^2 spectrum, the tags from each differentiation time point produced a unique signature reporter ion. The intensities of these reporter ions were used for the relative quantification of peptides. The identification of peptides was achieved through matching the resulting ion peaks to those indexed fragments in UniProt. hESC: Human embryonic stem cell; NSC: Neural stem cell; NPC: Neural progenitor cell; OPC: Oligodendrocyte progenitor cell; OL: Oligodendrocyte; ictrl: Inner control; TMT: Tandem mass tag, LC: liquid chromatography; MS: Mass spectrum

Figure 2: Temporal Profiling of Protein Expression through hESC differentiation into OL lineage. (A) Pearson correlation analysis along with the hierarchical clustering of the 3,855 quantified proteins reveals the biological replicates' cohesion and dynamics of the proteome during OL lineage differentiation. Red to green color range indicates a relative increase to the decrease of the protein abundance, respectively. (B) Principal component analysis (PCA) reveals a temporal trend in protein expression patterns. The same colour represents different replicates of the same differentiation time point. PC1 and PC2 axes demonstrate 30.9% and 11.5% of variations.

Figure 3: Proteome dynamic landscape of hESC differentiation into OL lineage and expression pattern of marker proteins. Unsupervised clustering of the quantified

proteins using the fuzzy c-means algorithm distinguishes three protein expression profiles (A-, B- and C-left charts) mainly based on protein abundance trends at the initial and final time points. The identified clusters are visualized separately with line charts plotting protein expression level against differentiation time points. Color-coded membership represents how well a single protein expression pattern fits with the general profile of the cluster. GO enrichment analysis of each cluster was performed with respect to the BPs by Enrichr. Some of the overrepresented BPs of each cluster are shown with bar charts (A-, B- and C-right charts). (D) Heat map illustrates standardized average protein Log2 ratios of expression changes of several stage-specific proteins along with differentiation. Markers of early differentiation stages (d0, d8, and d12) are members of cluster 1, which shows a slight decreasing trend. Cluster 2, with a slightly increasing pattern, accommodates CDH2, a marker of NSCs (d8), which is also involved in the regulation of OPCs (d80) proliferation and OL (d120) myelination. The heat map also reveals that (pre-)OPC and OL markers are generally assigned to the two increasing clusters (clusters 2 and 3). n: Represents the protein counts in a cluster; Eph: Ephrin; NADP: Nicotinamide adenine dinucleotide phosphate; hESC: Human embryonic stem cell; NSPC: Neural Stem and Progenitor Cell; OPC: Oligodendrocyte progenitor cell; OL: Oligodendrocyte; GO: Gene Ontology; BP: Biological processes

Figure 4: The dynamics of Wnt signaling associated proteins during OL lineage differentiation. (A) Heat map shows the standardized average protein Log2 ratios of expression changes of Wnt signaling associated proteins along the OL lineage differentiation process. The colour of the protein names’ bar demonstrates the cluster statue of proteins. Light gray, medium gray, and gray indicate if the protein is fitted for clusters 1, 2 or 3. Most changes occurred during the early and late stages. (B) The table shows the active Wnt signaling related biological processes (BP) in OL lineage differentiation. The enrichment analysis of the proteins of each cluster indicates their roles with the Wnt signaling related BPs. Their involvement scores are based on -Log10 of the adjusted p-value. The blank cells show there either were no participation or the -Log10 of the adjusted p-value was more than 0.05.

Figure 5: Expression profile of autophagy associated proteins through oligodendrocyte (OL) lineage differentiation. Heat map shows the expression (standardized average protein Log2 ratios) changes of autophagy associated proteins along the OL lineage differentiation process. The colour of the protein names' bar demonstrates the cluster statue of proteins. Light gray, and medium gray indicate if the protein is a fit for cluster 1or 2. The autophagy related biological processes (BPs) that each protein is implicated in is shown in front of it.

Figure 6: Stage-specific proteins of the oligodendrocyte (OL) lineage differentiation. (A - F) Volcano plots represent the comparison of protein expression at each time point with all other differentiation time points (or stages). The colour code was used for separating differentially expressed proteins (DEPs) and similarly expressed proteins. The red dots demonstrate proteins that were highly up-regulated at that specific stage (mentioned at the top right corner, above the plot) compared to all other stages while the green dots display the proteins with significantly low abundances at that stage, and the light yellow dots indicate the proteins with consistent expression level, Log2 (fold-Change) >1 and -Log10 (adjusted p-value) <0.05.

Figure 7: Dynamic proteome remodeling across the stepwise differentiation of hESC into the oligodendrocyte (OL) lineage cells. (A - E) Volcano plot representations of the log-ratio of protein expression values in two different consecutive differentiation stages. The colour code was used for separating differentially expressed proteins (stage transition specific proteins [STSPs]) and similarly expressed proteins. The red dots demonstrate proteins that highly up-regulated at the achieved stage while the green dots display the proteins with high abundances at its predecessor stage. The selection criteria of Log2 (fold-Change) >1 and -Log10 (adjusted p-value) <0.05 were used to indicate the differentially expressed stage transition proteins. (F) Generally, the STSPs can be classified in 14 protein sets (PS), illustrated as a colour-coded circular track, based on their differential expressions. The circular line between protein names and colour-coded track reveals the stages of the differentiation. As shown by the colour-coded bars, attached to the circular track, 42 of the STSPs are specific to more than one stage transition events. *i.e.*, CRABP1 (the black arrow) abundance increases during

d0 (human embryonic stem cell [hESC] stage) conversion into d8 (neural stem cell [NSC] stage), and its expression differentially down-regulates through d20 (pre-oligodendrocyte progenitor cell [pre-OPC] stage) to d80 (OPC stage) transition. The colour key is placed inside the circle.

Supplementary Figure S1: OL lineage cell generation. (A) Schematic representative of OL differentiation protocol (for materials information please refer to Methods and Table S3). First NANOG$^+$ hESCs (B) were induced to NSCs by dual SMAD inhibition. Then, neural progenitor patterning and OPC commitment were achieved by the application of two morphogens, RA and SAG. Subsequently, PDGF medium was used to promote OPC formation. From day 80 onward, Glial medium was utilized for OL derivation. SOX1$^+$ NSCs were detected on day 8 (C) and OLIG2$^+$ NPCs appeared on day 12 (D) and participated in aggregate formation after being detached. Consequently, NKX2.2$^+$ pre-OPCs (E), day 20, differentiated into PDGFRA$^+$ OPCs on day 80 (F); their further differentiation resulted in MBP$^+$ OLs (G) on day 120. The attained OLs demonstrate a typical OL morphology that consisted of a round, central soma with multiple branching processes that expanded symmetrically outward and gave the OL a spider-in-a-web-like appearance [168]. hESC: Human embryonic stem cell; RH6: Royan H6 cell line; NSC: Neural stem cell; NPC: Neural progenitor cell; OPC: Oligodendrocyte progenitor cell; OL: Oligodendrocyte; Scale bars: 50 µm

Supplementary Figure S2: Overall validation of the collected proteome data. (A) Proportional Venn diagram compares the depth of protein coverage in three replicates. A total of 3,527 proteins were identified in the three TMT mixtures that were analysed; while 1,056 proteins were only identified in two replicates. (B) The doughnut chart represents the diversity of the quantified proteins based on PANTHER protein class annotation.

Supplementary Figure S3: Enrichment plot of Wnt signaling components (from GSEA data set) between the last three time points (d20, d80 and d120) compared to the others using MSigDB set for Wnt signaling components. (FDR q-value 0.029).

Supplementary Figure S4: The contribution of macroautophagy in the OL lineage generation. (A) Cluster enrichment analysis featured the prominent participation of macroautophagy and autophagy pathways in OL lineage differentiation of hESCs. “in C2 and C3” reveal the clusters that these GO terms were enriched in. (B) Schematic illustration of the process and main regulatory machinery of macroautophagy. AMP-

activated kinase (AMPK) signaling is depicted as the activator of the macroautophagy process (initiation) that targets the ULK1 (Unc-51-like kinase 1) initiation complex. The initiation complex then triggers membrane nucleation and phagophore formation. Hence, the cup-shaped double membrane phagophore begins to engulf the autophagic cargo and expands into the double-membrane vesicle (autophagosome) that sequesters the cargos completely (phagophore expansion). Subsequently, the autophagosome fuses with acidic lysosomes (fusion with the lysosome) and forms autolysosomes, where the cargo will be degraded (degradation). The colored ovals encompass the proteins quantified in our study of OL lineage differentiation. This figure is adapted from “Autophagy as a promoter of longevity: insights from model organisms,” by M. Hansen, D. C. Rubinsztein, and D. W. Walker, 2018, Nat. Rev. Mol. Cell Biol., vol. 19, no. 9, pp. 579–593.

Supplementary Figure S5: Illustration of the abundances of differentially expressed proteins at each stage of oligodendrocyte lineage differentiation. Heat map shows the standardized average protein Log2 ratios of expression changes of differentially expressed proteins (DEPs) at each step of oligodendrocyte (OL) lineage differentiation. The seven expression profile clusters (left colour-coded bar) describe stage-specific patterns of the dynamics of 378 DEPs. The clusters are indicated by different colors, each of which demonstrates one specific differentiation step.

Supplementary Table S3. Detailed compositions of the culture media used in the experiments.

Media	Components	Provider	Final Conc.
NI medium	DMEM/F12	Life Technologies	
	Knock-Out Serum⁎	Life Technologies	5%
	MEM Non-Essential Amino Acids (100X)	Life Technologies	1X
	GlutaMAX-I (100X)	Life Technologies	1X
	Penicillin-Streptomycin (100X)	Life Technologies	1X
	2-Mercaptoethanol (1000X)	Life Technologies	1X
	Insulin-Transferrin-Selenium (100X)	Life Technologies	1X
	N-2 Supplement (100X)	Life Technologies	1X
	SB431542	Stemgent	10 µM
	LDN193189	Stemgent	250 nM
	All Trans-Retinoic Acid	Sigma	100 nM
N2 medium	DMEM/F12	Life Technologies	
	MEM Non-Essential Amino Acids (100X)	Life Technologies	1X
	GlutaMAX-I (100X)	Life Technologies	1X
	Penicillin-Streptomycin (100X)	Life Technologies	1X
	2-Mercaptoethanol (1000X)	Life Technologies	1X
	Insulin-Transferrin-Selenium (100X)	Life Technologies	1X
	N-2 Supplement (100X)	Life Technologies	1X

	All Trans-Retinoic Acid	Sigma	100 nM
	Agonist of Sonic Hedgehog (SAG)	Stemgent	1 µM
N2B27 medium	DMEM/F12	Life Technologies	
	MEM Non-Essential Amino Acids (100X)	Life Technologies	1X
	GlutaMAX-I (100X)	Life Technologies	1X
	Penicillin-Streptomycin (100X)	Life Technologies	1X
	2-Mercaptoethanol (1000X)	Life Technologies	1X
	Insulin-Transferrin-Selenium (100X)	Life Technologies	1X
	N-2 Supplement (100X)	Life Technologies	1X
	B-27 Supplement without Vitamin A (50X)	Life Technologies	1X
	All Trans-Retinoic Acid	Sigma	100 nM
	SAG	Stemgent	1 µM
PDGF medium	DMEM/F12	Life Technologies	
	MEM Non-Essential Amino Acids (100X)	Life Technologies	1X
	GlutaMAX-I (100X)	Life Technologies	1X
	Penicillin-Streptomycin (100X)	Life Technologies	1X
	2-Mercaptoethanol (1000X)	Life Technologies	1X
	N-2 Supplement (100X)	Life Technologies	1X
	B-27 Supplement without Vitamin A (50X)	Life Technologies	1X
	Insulin	Sigma	25 µg/ml
	PDGF-AA	Sigma	10 ng/ml

	HGF	Sigma	5 ng/ml
	IGF-1	Sigma	10 ng/ml
	NT3	Royan Biotech	10 ng/ml
	T3	Sigma	60 ng/ml
	Biotin	Sigma	100ng/ml
	cAMP	Sigma	1µM
Glial medium	DMEM/F12	Life Technologies	
	MEM Non-Essential Amino Acids (100X)	Life Technologies	1X
	GlutaMAX-I (100X)	Life Technologies	1X
	Penicillin-Streptomycin (100X)	Life Technologies	1X
	2-Mercaptoethanol (1000X)	Life Technologies	1X
	N-2 Supplement (100X)	Life Technologies	1X
	B-27 Supplement without Vitamin A (50X)	Life Technologies	1X
	Insulin	Sigma	25 µg/ml
	HEPES	Sigma	10 mM
	T3	Sigma	60 ng/ml
	Biotin	Sigma	100ng/ml
	cAMP	Sigma	1µM
	AA	Sigma	20 µg/ml

⁕ From day four of differentiation, Knock-Out serum was gradually replaced by N-2 supplement (25%, 50%, 75%) as described previously [155].

Supplementary Table S4. List of antibodies used in the experiments.

Type	Antigen	Dilution	Host	Provider	Cat. No.
Primary antibodies	NANOG	1:100	Goat	R&D Systems	AF1997
	SOX1	1:200	Goat	R&D Systems	AF3369
	OLIG2	1:500	Rabbit	Sigma	AB9610
	NKX2.2	1:50	Mouse	Developmental Studies Hybridoma Bank	74.5A5
	PDGFRA	1:500	Rabbit	Abcam	ab61219
	MBP	1:100	Mouse	Sigma	SAB5300427
Secondary antibodies (Alexa Fluor 488 or 546)	Goat IgG	1:500	Donkey	Invitrogen Antibodies	A-11055
	Goat IgG	1:500	Donkey	Invitrogen Antibodies	A-11056
	Rabbit IgG	1:500	Donkey	Invitrogen Antibodies	A-10040
	Mouse IgG	1:500	Donkey	Invitrogen Antibodies	A-10036
	Mouse IgG	1:500	Donkey	Invitrogen Antibodies	A-21202

Supplementary Table S5. Details of the reagents used for protein isolation.

Reagent	Provider	Cat. No.
TRIzol^{TM} Reagent	Invitrogen	15596018
Chloroform	Merck Millipore	102444
Absolute ethanol	Merck Millipore	107017
Acetone	Merck Millipore	100014
Guanidine hydrochloride	Sigma-Aldrich	G3272
Glycerol	Sigma-Aldrich	G5516
Urea	Sigma-Aldrich	U5378
Thiourea	Sigma-Aldrich	T8656
CHAPS hydrate	Sigma-Aldrich	C3023
1,4-Dithiothreitol	Sigma-Aldrich	D0632
Protease Inhibitor Cocktail powder	Sigma-Aldrich	P2714
Phosphatase Inhibitor Cocktail 2	Sigma-Aldrich	P5726

Figure 1

Click here to access/download;Figure;Fig 1.pdf

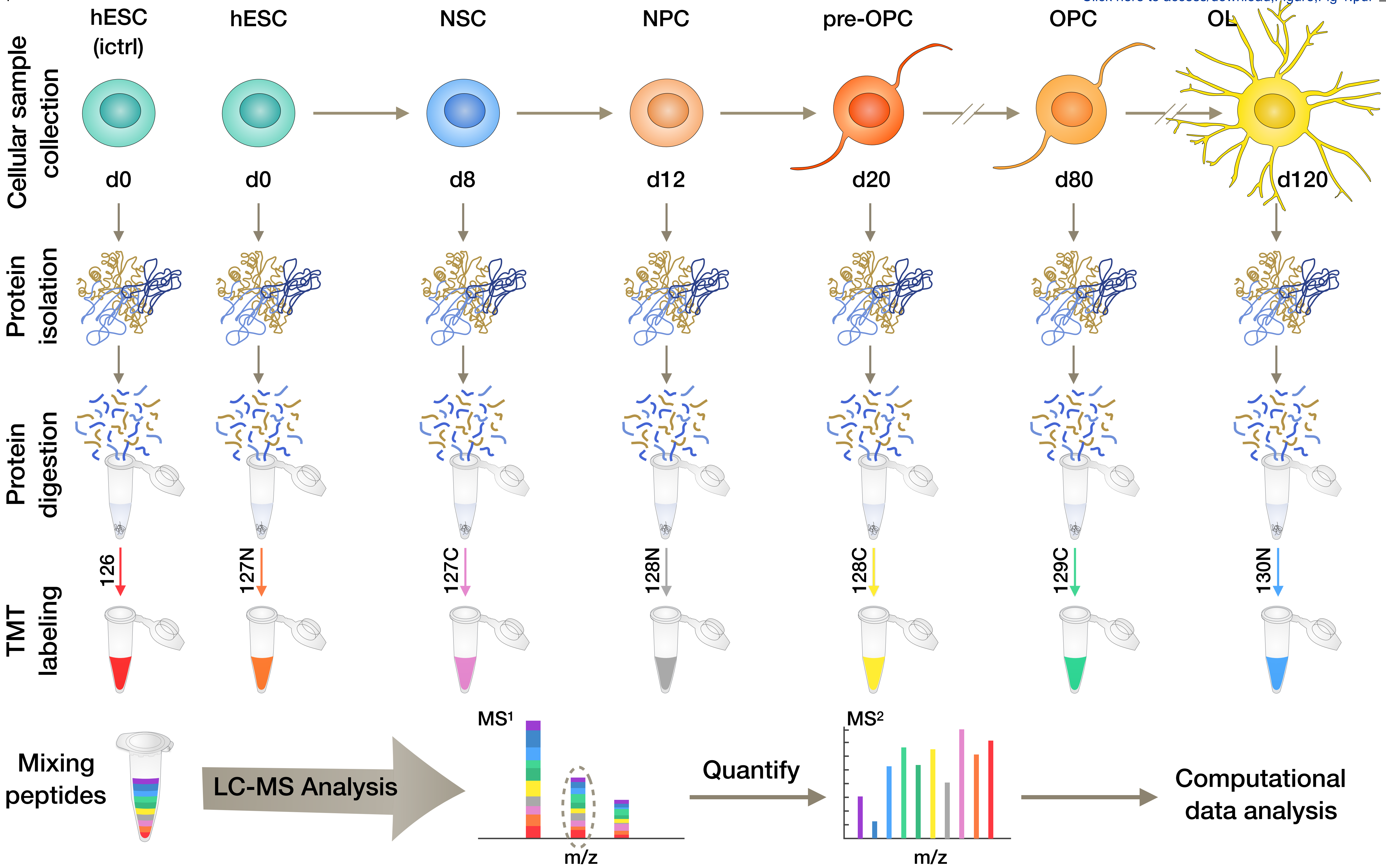

Figure 2

Click here to access/download;Figure;Fig 2.pdf

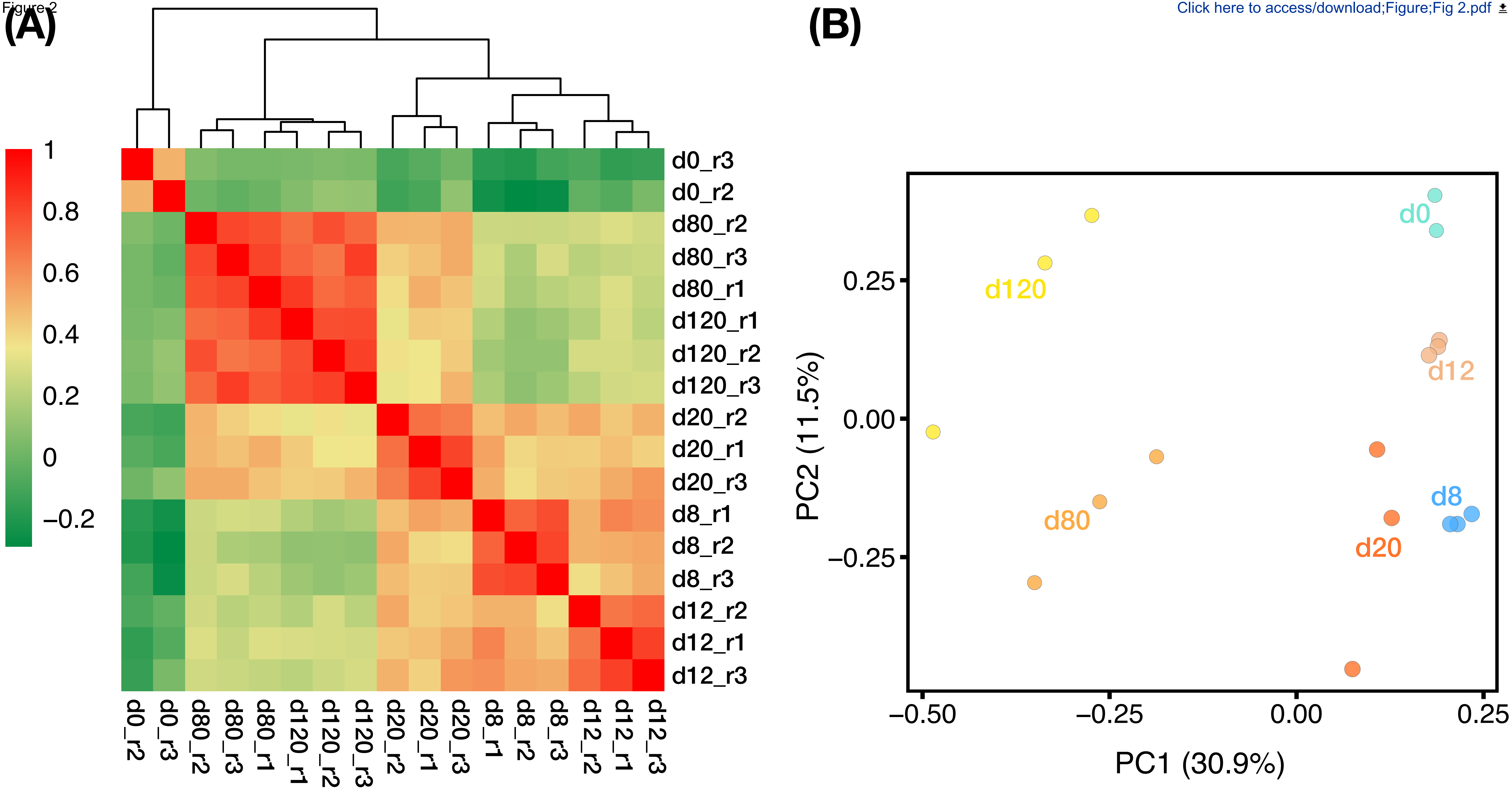

Figure 3

Click here to access/download;Figure;Fig 3.pdf

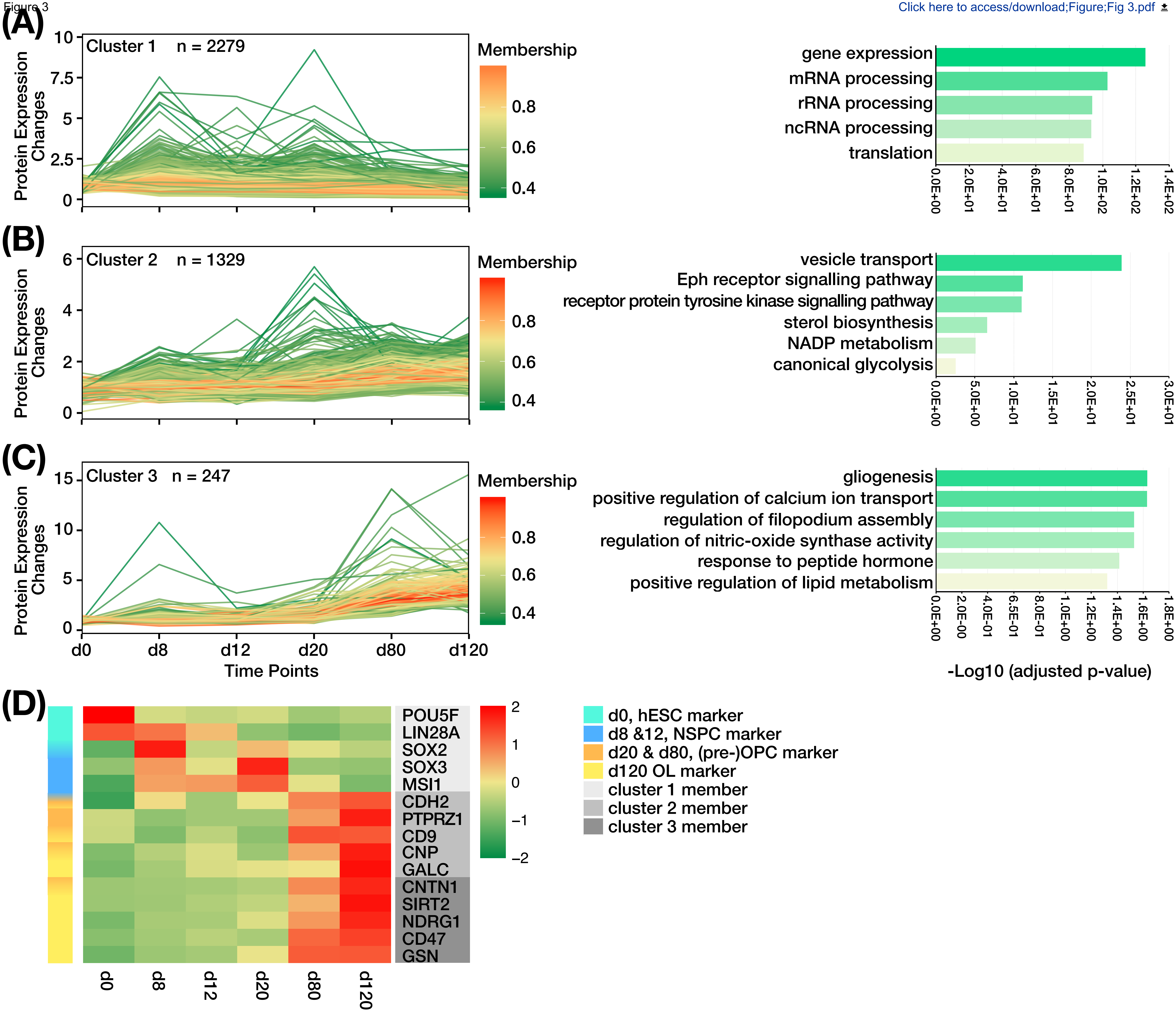

Figure 4

Click here to access/download;Figure;Fig 4.pdf

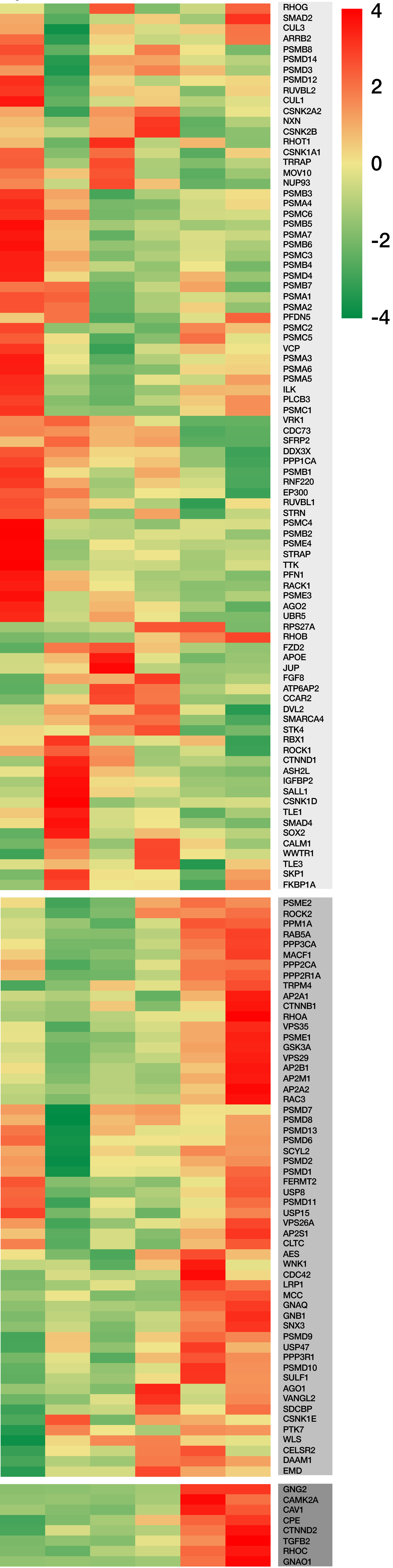

	positive regulation of canonical WNT signaling pathway	WNT signaling pathway, planar cell polarity pathway	positive regulation of WNT signaling pathway	negative regulation of canonical WNT signaling pathway	non-canonical WNT signaling pathway	regulation of canonical WNT signaling pathway	negative regulation of WNT signaling pathway
Clus. 1	1.2E-08	3.1E-08	6.8E-08	1.1E-06	1.2E-06	3.9E-06	5.1E-06
Clus. 2		1.3E-05	0.005	0.014	1.3E-05		
Clus. 3							

cluster 1 member cluster 2 member cluster 3 member

Click here to access/download;Figure;Fig 5.pdf

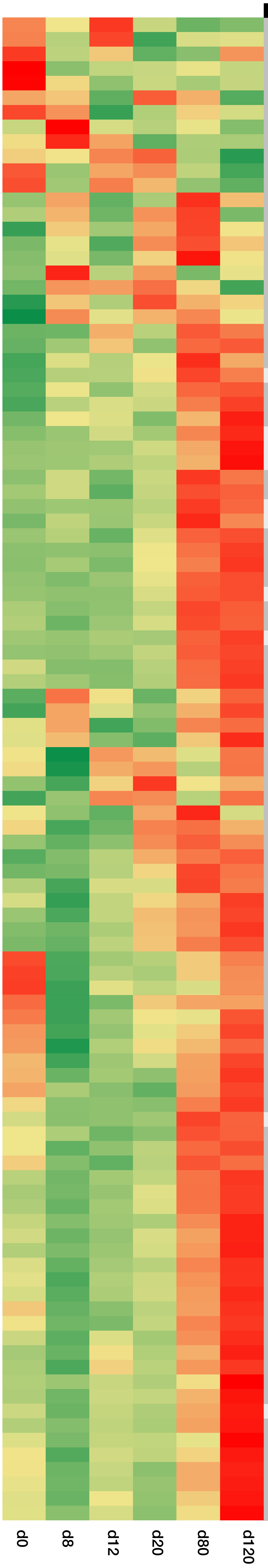

Protein	Term
MFN1	macroautophagy
PIP4K2C	positive regulation of autophagosome assembly; regulation of autophagy
SNX5	regulation of macroautophagy
EIF4G1	negative regulation of autophagy
HSPA8	Chaperone-mediated autophagy; Late endosomal microautophagy
ATG16L1	autophagosome assembly; macroautophagy
CHMP4A	negative regulation of autophagosome assembly; macroautophagy
ATP6V1G1	regulation of macroautophagy
TOMM70	macroautophagy
VPS4B	macroautophagy
PRKAA2	macroautophagy; regulation of macroautophagy; positive regulation of autophagy; positive regulation of macroautophagy
VPS25	macroautophagy
ABL1	autophagy; regulation of autophagy
RAB1B	regulation of autophagosome assembly; autophagy
SNAP29	autophagosome maturation; autophagosome membrane docking
CHMP4B	negative regulation of autophagosome assembly; macroautophagy; autophagy
MTDH	positive regulation of autophagy
TOMM40	macroautophagy
VPS36	macroautophagy
BNIP3	positive regulation of macroautophagy; positive regulation of autophagy; autophagy of mitochondrion; positive regulation of autophagy of mitochondrion
EI24	macroautophagy
IFI16	autophagy; regulation of autophagy
RAB39B	autophagy; regulation of autophagy
ATP6V1E1	regulation of macroautophagy
OPTN	autophagy; positive regulation of autophagy
LAMTOR1	regulation of macroautophagy
SRC	macroautophagy
VPS18	autophagy
GBA	autophagosome organization; regulation of macroautophagy; autophagy; positive regulation of autophagy of mitochondrion in response to mitochondrial depolarization
INS	regulation of autophagy
SIRT2	autophagy; negative regulation of autophagy
HGS	macroautophagy
MLST8	regulation of macroautophagy
MAPT	regulation of autophagy
PIP4K2B	positive regulation of autophagosome assembly; regulation of autophagy
SNX6	regulation of macroautophagy
ATP6V1B2	regulation of macroautophagy
GABARAPL2	autophagosome assembly; autophagosome maturation; macroautophagy; autophagy; autophagy of mitochondrion
RAB1A	autophagosome assembly; autophagy
MAP1LC3B2	Autophagosome assembly; Autophagy of mitochondrion
ATP6V1A	regulation of macroautophagy
PRKAA1	macroautophagy; regulation of macroautophagy; positive regulation of autophagy
DCN	positive regulation of macroautophagy; positive regulation of autophagy
VPS26B	regulation of macroautophagy
RAB5A	regulation of autophagosome assembly
SH3GLB1	positive regulation of autophagy; regulation of macroautophagy; autophagic cell death; autophagy; positive regulation of autophagosome assembly
LAMTOR3	regulation of macroautophagy
STAM	macroautophagy
CHMP2B	macroautophagy; autophagy
VPS4A	macroautophagy
SNF8	macroautophagy
TOMM22	macroautophagy
BCL2	negative regulation of autophagy
CISD2	regulation of autophagy; autophagy of mitochondrion
ATG2B	autophagosome assembly; autophagy of mitochondrion
PIP4K2A	positive regulation of autophagosome assembly; regulation of autophagy
TSG101	autophagosome maturation; macroautophagy
CHMP6	macroautophagy
HDAC6	regulation of autophagy of mitochondrion; regulation of macroautophagy; Parkin-mediated stimulation of mitophagy in response to mitochondrial depolarization
VPS28	macroautophagy
ATP6V1D	regulation of macroautophagy
VPS11	autophagy
AKT1	negative regulation of macroautophagy; negative regulation of autophagy
RAB24	autophagy
ATG5	autophagosome assembly; macroautophagy; autophagy; autophagy of mitochondrion
RAB3GAP2	positive regulation of autophagosome assembly
EIF4G2	negative regulation of autophagy
RAB3GAP1	positive regulation of autophagosome assembly
PIK3C3	autophagosome assembly; macroautophagy; autophagy
VPS26A	regulation of macroautophagy
DNM1L	regulation of autophagy of mitochondrion
VTA1	macroautophagy
CAPN1	regulation of macroautophagy
RAB33B	autophagosome assembly
RAB12	autophagy; positive regulation of macroautophagy
HSPB1	regulation of autophagy
UCHL1	regulation of macroautophagy
PIKFYVE	regulation of autophagosome assembly
VPS16	autophagosome maturation
CHMP2A	macroautophagy
CDK5	regulation of macroautophagy
EXOC8	regulation of macroautophagy
ATG9A	autophagosome assembly; autophagy of mitochondrion
GSK3A	positive regulation of autophagy; regulation of autophagy of mitochondrion
MTOR	regulation of macroautophagy; negative regulation of autophagy
CAPNS1	regulation of macroautophagy
EXOC4	regulation of macroautophagy
VPS35	regulation of macroautophagy
ATG3	autophagosome assembly; macroautophagy; autophagy of mitochondrion
PAFAH1B2	positive regulation of macroautophagy [GO:0016239]
ATP6V1H	regulation of macroautophagy
ATP6V0D1	regulation of macroautophagy
PRKAG1	macroautophagy; regulation of macroautophagy
RPTOR	regulation of macroautophagy
VPS33A	autophagosome maturation
RRAGA	regulation of autophagy (GO:0010506); Negative regulation of autophagy (GO:0010507)
EXOC1	regulation of macroautophagy
ATG7	autophagosome assembly; macroautophagy; autophagy; autophagy of mitochondrion; positive regulation of autophagy
ATP6V0A1	regulation of macroautophagy
ATP6V1C1	regulation of macroautophagy
EXOC7	regulation of macroautophagy
MFN2	macroautophagy
RAB7A	autophagosome assembly

Figure 6 Click here to access/download;Figure;Fig 6.pdf

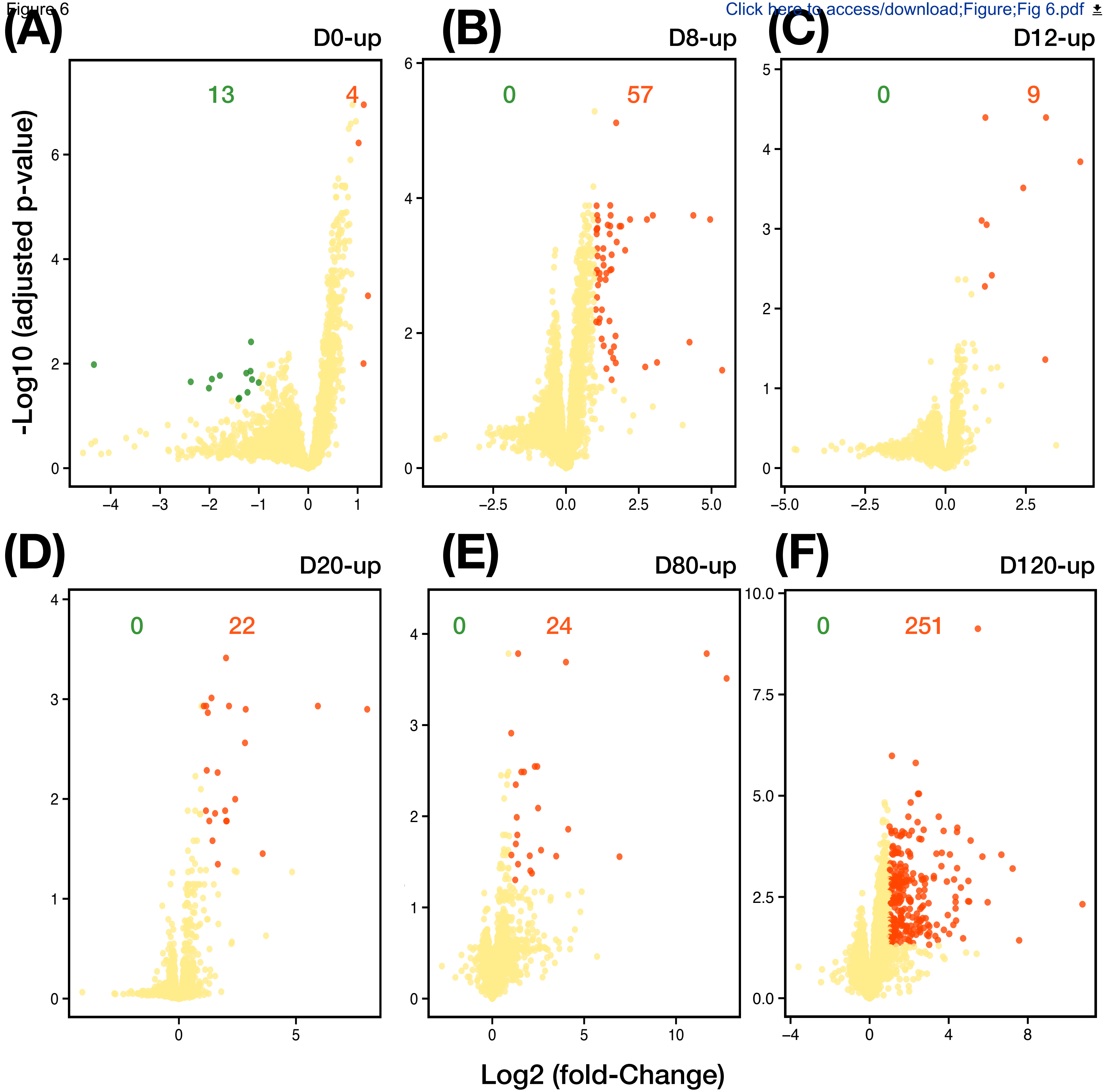

Figure 7

Click here to access/download;Figure;Fig 7.pdf

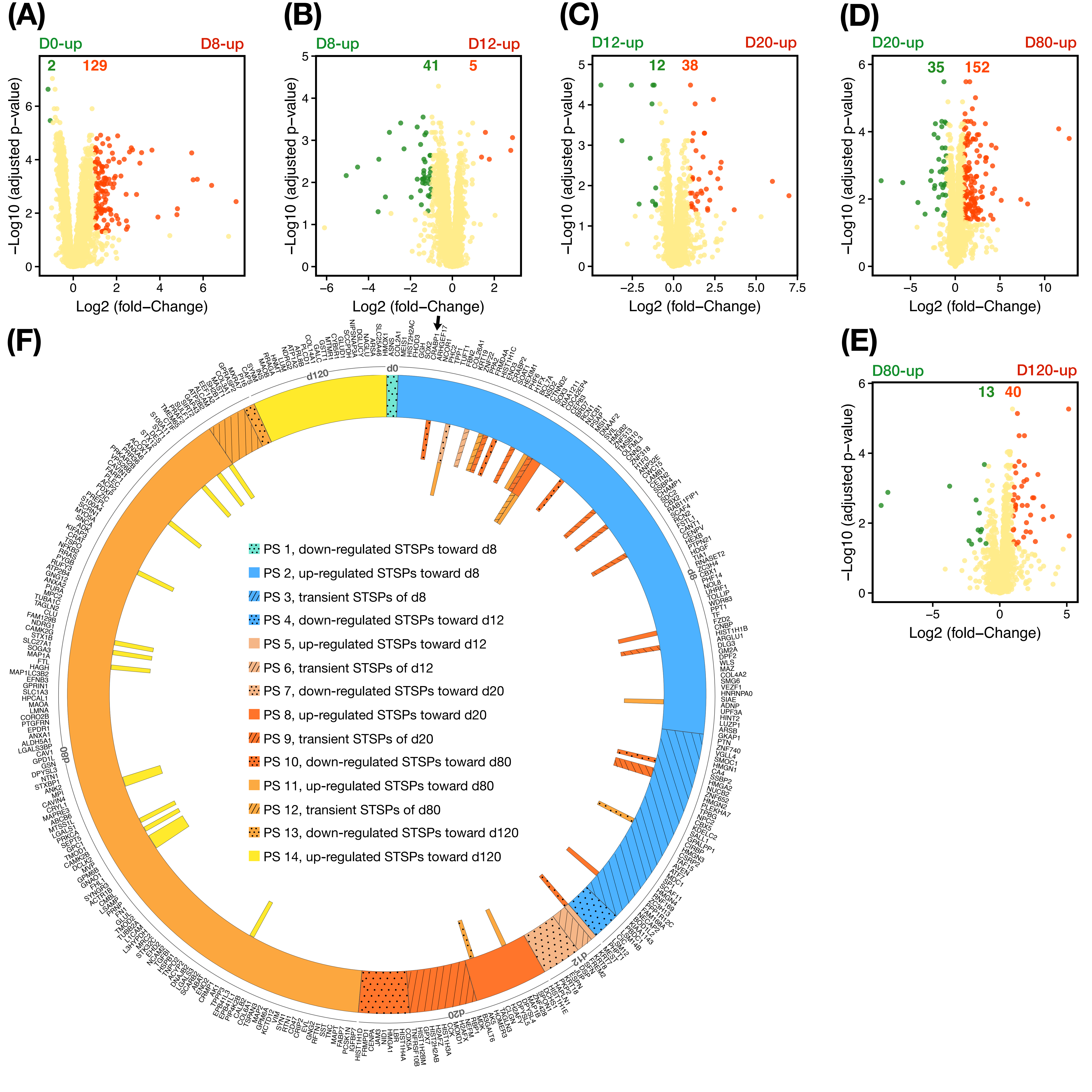

Supplementary Fig 1

Click here to access/download
Supplementary Material
Fig S1.pdf

Supplementary Figure 2

Click here to access/download

Supplementary Material

Fig S2.pdf

Supplementary Figure 3

Click here to access/download
Supplementary Material
Fig S3.pdf

Supplementary Figure 4

Click here to access/download
Supplementary Material
Fig S4.pdf

Supplementary Figure 5

Click here to access/download
Supplementary Material
Fig S5.pdf

Supplementary Table 1

Click here to access/download
Supplementary Material
Table S1.xlsx

Supplementary Table 2

Click here to access/download
Supplementary Material
Table S2.xlsx
