# Supplementary material for: The Dynamic Proteome of Oligodendrocyte Lineage Differentiation Features Planar Cell Polarity and Macroautophagy Pathways
Source: Gigascience. 2020 Oct 31;9(11):giaa116. doi: 10.1093/gigascience/giaa116 (PMC7601170; doi:10.1093/gigascience/giaa116)
Supplement: giaa116_GIGA-D-20-00058_Revision_3 [file giaa116_giga-d-20-00058_revision_3.pdf]

GigaScience

The Dynamic Proteome of Oligodendrocyte Lineage Differentiation Features Planar Cell Polarity and Macroautophagy Pathways

--Manuscript Draft--

Manuscript Number:	GIGA-D-20-00058R3
Full Title:	The Dynamic Proteome of Oligodendrocyte Lineage Differentiation Features Planar Cell Polarity and Macroautophagy Pathways
Article Type:	Data Note
Funding Information:	Royan Institute (243154) — Ms Paria Pooyan
Abstract:	Background<br><br>Generation of oligodendrocytes (OLs) is a sophisticated multistep process, mechanistic underpinnings of which are not fully understood and demand further investigation. To systematically profile proteome dynamics during human embryonic stem cell (hESC) differentiation into OLs, we applied in-depth quantitative proteomics at different developmental stages and monitored changes in protein abundance using a multiplexed tandem mass tag (TMT) based proteomics approach.<br><br>Findings<br><br>Our proteome data provided a comprehensive protein expression profile that highlighted specific expression clusters based on the protein abundances over the course of human OL lineage differentiation. We identified the eminence of the planar cell polarity (PCP) signaling and autophagy (particularly macroautophagy) in the progression of OL lineage differentiation; the cooperation of which is assisted by 106 and 77 proteins, respectively, that showed significant expression changes in this differentiation process. Further, differentially expressed protein (DEP) analysis of the proteome profile of OL lineage cells revealed 378 proteins that were specifically up-regulated only in one differentiation stage. In addition, comparative pairwise analysis of differentiation stages demonstrated that abundances of 352 proteins differentially changed between consecutive differentiation time points.<br><br>Conclusion<br><br>Our study provides a comprehensive systematic proteomics profile of OL lineage cells that can serve as a rich resource not only for identifying novel biomarkers from these cells, but also for indicating numerous proteins that may contribute to the regulation of the development of myelinating OLs and other cells of OL lineage. We showed the importance of PCP signaling in OL lineage differentiation, and revealed the autophagy-related proteins that participate in OL lineage Differentiation.
Corresponding Author:	Ghasem Hosseini Salekdeh<br>Macquarie University Faculty of Science and Engineering<br>Sydney, NSW AUSTRALIA
Corresponding Author Secondary Information:	
Corresponding Author's Institution:	Macquarie University Faculty of Science and Engineering
Corresponding Author's Secondary Institution:	
First Author:	Paria Pooyan
First Author Secondary Information:	
Order of Authors:	Paria Pooyan<br>Razieh Karamzadeh

Powered by Editorial Manager® and ProduXion Manager® from Aries Systems Corporation

	Mehdi Mirzaei
	Anna Meyfour
	Ardeshir Amirkhan
	Yunqi Wu
	Vivek Gupta
	Hossein Baharvand
	Mohammad Javan
	Ghasem Hosseini Salekdeh
**Order of Authors Secondary Information:**	
**Response to Reviewers:**	Response to the editorial comments<br>1. AU: Please add the following sentence here:<br>"Other data further supporting this work are openly available in the GigaScience respository, GigaDB [192]."<br>RE: done!<br>2. AU: Please add new Reference #192 and cite the DOI exactly as follows:<br>Pooyan P; Karamzadeh R; Mirzaei M; Meyfour A; Amirkhan A; Wu Y; Gupta V; Baharvand H; Javan M; Salekdeh GH: Supporting data for "The Dynamic 192. Proteome of Oligodendrocyte Lineage Differentiation Features Planar Cell Polarity and Macroautophagy Pathways" GigaScience Database. 2020. http://dx.doi.org/10.5524/100798<br>RE: The new reference and its DOI was added as instructed<br>3. Added edits<br>We also edited the following parts:<br>On page 22, line 625: the misplaced parenthesis was removed.<br>On page 31, line 893: the legend text for Fig. S6C was clarified by adding the letter C.<br>On page 31, line 895: the PDGF protein name is now written in its standard format "PDGFRA"
**Additional Information:**	
**Question**	**Response**
Are you submitting this manuscript to a special series or article collection?	No
**Experimental design and statistics**<br><br>Full details of the experimental design and statistical methods used should be given in the Methods section, as detailed in our Minimum Standards Reporting Checklist. Information essential to interpreting the data presented should be made available in the figure legends.<br><br>Have you included all the information requested in your manuscript?	Yes
**Resources**<br><br>A description of all resources used,	Yes

including antibodies, cell lines, animals and software tools, with enough information to allow them to be uniquely identified, should be included in the Methods section. Authors are strongly encouraged to cite Research Resource Identifiers (RRIDs) for antibodies, model organisms and tools, where possible.<br><br>Have you included the information requested as detailed in our Minimum Standards Reporting Checklist?	
**Availability of data and materials**<br><br>All datasets and code on which the conclusions of the paper rely must be either included in your submission or deposited in publicly available repositories (where available and ethically appropriate), referencing such data using a unique identifier in the references and in the "Availability of Data and Materials" section of your manuscript.<br><br>Have you have met the above requirement as detailed in our Minimum Standards Reporting Checklist?	Yes

Manuscript

Click here to access/download;Manuscript;Pooyan et al-revised 3.docx

The Dynamic Proteome of Oligodendrocyte Lineage Differentiation Features Planar Cell Polarity and Macroautophagy Pathways

Paria Pooyan[1,2,3†], Razieh Karamzadeh[1,2,3†], Mehdi Mirzaei[4,5], Anna Meyfour[6], Ardeshir Amirkhan[5], Yunqi Wu[5], Vivek Gupta[7], Hossein Baharvand[2,3,8*], Mohammad Javan[3,9*], Ghasem Hosseini Salekdeh[1,4*]

[1] Department of Molecular Systems Biology, Cell Science Research Center, Royan Institute for Stem Cell Biology and Technology, ACECR, Tehran, Iran

[2] Department of Stem Cells and Developmental Biology, Cell Science Research Center, Royan Institute for Stem Cell Biology and Technology, ACECR, Tehran, Iran

[3] Department of Brain and Cognitive Science, Cell Science Research Center, Royan Institute for Stem Cell Biology and Technology, ACECR, Tehran, Iran

[4] Department of Molecular Sciences, Macquarie University, Sydney, New South Wales 2109, Australia.

[5] Australian Proteome Analysis Facility, Macquarie University, North Ryde, NSW, Australia

[6] Basic and Molecular Epidemiology of Gastrointestinal Disorders Research Center, Research Institute for Gastroenterology and Liver Diseases, Shahid Beheshti University of Medical Sciences, Tehran, Iran

[7] Department of Clinical Medicine, Macquarie University, Sydney, NSW, Australia

[8]Department of Developmental Biology, University of Science and Culture, Tehran, Iran

[9]Department of Physiology, Faculty of Medical Sciences, Tarbiat Modares University, Tehran, Iran

*Corresponding authors

Ghasem Hosseini Salekdeh, Ph.D., Mohammad Javan, Ph.D., Hossein Baharvand, Ph.D.

Royan Institute, Banihashem Sq., Banihashem St., Resalat highway, Tehran, Iran. Postal Code: 1665659911, P.O. Box: 16635-148, Tel: +98 21 22306485, Fax: +98 21 23562507. Emails: hsalekdeh@gmail.com, mjavan@royaninstitute.org, baharvand@royaninstitute.org

†These authors equally contributed and should be regarded as co-first authors.

Abstract

Background: Generation of oligodendrocytes is a sophisticated multistep process, mechanistic underpinnings of which are not fully understood and demand further investigation. To systematically profile proteome dynamics during human embryonic stem cell differentiation into oligodendrocytes, we applied in-depth quantitative proteomics at different developmental stages and monitored changes in protein abundance using a multiplexed tandem mass tag-based proteomics approach. **Findings:** Our proteome data provided a comprehensive protein expression profile that highlighted specific expression clusters based on the protein abundances over the course of human oligodendrocyte lineage differentiation. We identified the eminence of the planar cell polarity signaling and autophagy (particularly macroautophagy) in the progression of oligodendrocyte lineage differentiation; the cooperation of which is assisted by 106 and 77 proteins, respectively, that showed significant expression changes in this differentiation process. Further, differentially expressed protein analysis of the proteome profile of oligodendrocyte lineage cells revealed 378 proteins that were specifically up-regulated only in one differentiation stage. In addition, comparative pairwise analysis of differentiation stages demonstrated that abundances of 352 proteins differentially changed between consecutive differentiation time points. **Conclusions:** Our study provides a comprehensive systematic proteomics profile of oligodendrocyte lineage cells that can serve as a rich resource for identifying novel biomarkers from these cells, and for indicating numerous proteins that may contribute to the regulation of the development of myelinating oligodendrocytes and other cells of oligodendrocyte lineage. We showed the importance of planar cell polarity signaling in oligodendrocyte lineage differentiation, and revealed the autophagy-related proteins that participate in oligodendrocyte lineage Differentiation.

Keywords: human embryonic stem cell; neural stem cell; progenitor cell; oligodendrocyte; Wnt signaling; autophagy; quantitative proteomics; multiplexed tandem mass tag

Data Description

Background

Oligodendrocytes (OLs; for abbreviations, please refer to Supplementary Table S1) are the myelinating cells of the central nervous system (CNS) that insulate axons with their multispiral membrane-forming myelin. Therefore, OLs allow swift saltatory conduction of action potentials in the CNS [1]. The functional significance of OLs is manifested through myelin loss, in addition to its damage or dysfunction-related neurological disorders such as multiple sclerosis (MS), optic neuritis (ON), spinal cord injury (SCI), and Pelizaeus–Merzbacher disease (PMD) [2]. Irrespective of its background, myelin loss and nervous system failure in remyelination lead to conduction hindrance along the axonal fibers, followed by the destruction of nerve impulses, degenerative axonal loss, and the accumulation of functional disabilities [3]. Oligodendrocyte progenitor cells (OPCs) are the main source of new OLs that can carry on the remyelination process, while neural stem cells (NSCs) and neural progenitor cells (NPCs) may also contribute in new OL generation. In a nutshell, remyelination demands the activation, recruitment, and OL-differentiation of OPCs, and possibly their predecessors [2].

A deeper understanding of the biology of myelinating OL generation alongside their progenitors can equip us with invaluable tools to achieve proper remyelination and preventing further clinical complications of the diseases related to myelin destruction. To accomplish this goal, it is necessary to understand the ways to 1) improve NSCs, NPCs, and OPCs migration into the required site; 2) enhance the mentioned cells survival especially during this process; and 3) boost their differentiation into myelinating OLs in demyelination niche. To fulfill these prospects, we conducted an in-depth quantitative proteomic analysis that spanned the entire course of OL lineage cells generation in an attempt to survey the order, timing, and magnitude of proteome changes during human embryonic stem cell (hESC) differentiation into OL lineage cells. This versatile differentiation model system provides tremendous insight into human OL development, in addition to the information needed for targeted/specific cell-based medical therapies and overall disease modeling [4,5].

Therefore, we studied the global proteome signature of developing OL by conducting a stepwise differentiation process to differentiate the hESC RH6 line into an OL lineage. This process provided us with cell samples from each of the distinct stages of OL differentiation: hESCs, NSCs, NPCs, pre-OPCs, OPCs, and OLs [6,7]. We attempted to use the advantage of the sensitive and precise TMT-based quantitative proteomics in order to spot every stages' specific proteins in OL lineage differentiation, and to identify the key proteins of each step achievement. Our study provides an inclusive profile of the proteins involved in Wnt signaling throughout OL lineage differentiation. Our findings put planar cell polarity (PCP) noncanonical Wnt signaling in the spotlight for further analysis of this controversial signaling of OL differentiation. In addition, the proteome of OL lineage differentiation presents an all-embracing autophagy associated protein profile and accentuates macroautophagy as a valuable contributing factor in OL lineage differentiation.

Global characterization of protein expression during oligodendrocyte (OL) generation

In order to provide a systematic proteomic profiling map of the representative cells in human OL development, the hESC line Royan H6 (RH6) [8] was differentiated into OL lineage through a well-defined stepwise protocol [6,7,9] (Supplementary Fig. S1A). Briefly, hESC (d0, Supplementary Fig. S1B) differentiation was initiated by neural induction through dual SMAD inhibition; within eight days, we observed the presence of SOX1^{+} NSCs (d8, Supplementary Fig. S1C). Further treatment of NSCs by caudalizing and ventralizing morphogens, gave rise to OLIG2^{+} NPCs on day 12 (d12, Supplementary Fig. S1D), which then committed to an OL lineage by day 20 (d20, NKX2.2^{+} pre-OPCs, Supplementary Fig. S1E). Next, maturation of pre-oligodendrocyte progenitor cells (pre-OPCs) was promoted via a chemically defined, growth factor-rich medium, and PDGFRA^{+} OPCs were generated on day 80 (d80, Supplementary Fig. S1F). Finally, PDGFRA^{+} OPCs were terminally differentiated into MBP^{+} OLs (d120, Supplementary Fig. S1G).

Following recapitulation of OL lineage development, the cells were harvested at six distinct time points (in three biological replicates per time point), which corresponded to

the hESC (d0), NSC (d8), NPC (d12), pre-OPC (d20), OPC (d80), and OL (d120) differentiation stages (Fig. 1) [6,7]. Then to accommodate all the biological replicates of each time point, three tandem mass tag (TMT) mass spectrometry experiments were carried out (Fig. 1).

For in-depth quantitative proteomic analysis, the harvested cells were homogenized and protein extracts of each sample were treated with lysine-C/trypsin sequential digestion. Subsequently, the peptides were quantified and subjected to TMT labelling (Fig. 1), then fractionated and analysed by a high-resolution nanoflow liquid chromatography positive ion electrospray ionization tandem mass spectrometry (nanoflow LC/ESI-MS/MS) on a Q Exactive Orbitrap mass spectrometer (Thermo Scientific). Therefore, upon fragmentation in MS/MS mode, sequence assignment of the MS/MS spectra achieved using indexed human UniProt database [10], and next quantification of relative protein expression changes accomplishment from the fragmentation of the tags, which gave rise to mass reporter ions. The mass spectrometry proteomics data can be retrieved via the ProteomeXchange Consortium [11] through the PRIDE partner repository (accession code: PXD017649). In total, at false discovery rate (FDR) of 1%, a total of 66,083 peptides and 59,404 unique peptides from 5,753 unique proteins were identified; among them, 3,527 unique proteins were identified within all three biological replicates (Supplementary Fig. S2A), and 3,519 of unique proteins were quantified across all time points (Supplementary Table S2). Furthermore, 1,056 identified proteins were found to be in common only between two replicas (Supplementary Fig. S2A) of which 1045 proteins were quantified across all time points. Applying a supervised approach [12] on these 1045 proteins and on proteins which were identified in all time points of the three TMT experiments but were not quantified in all of them, resulted in the imputation of quantitative measurements of 336 proteins. Importantly, according to the analysis of variance (ANOVA) about 81% (3132 proteins) of the proteins showed significant changes through the OL lineage differentiation of hESCs (adjusted p-value ≤ 0.05; Supplementary Table S2). Pearson correlation coefficient coupled with hierarchical clustering (using the relative expression for all of the 3,855 quantified proteins) implied a high degree of consistency among sample replicates (Fig. 2A, Supplementary Table S3). The heat map

presentation of the protein distribution profiles demonstrates five distinct groups associated with the differentiation steps. It also represents d8 (NSC stage), d12 (NPC stage), and d20 (pre-OPC stage) in one supergroup, and d20, d80 (OPC stage), and d120 (OL stage) in another supergroup. Therefore, in agreement with the sequential stages of the differentiation process, d20 demonstrated a transition state between the initial and final steps (Fig. 2A). The standard Principal component analysis (PCA) was performed to project the proteome profile of each differentiation time point into a two-dimensional space. PCA clustered all three replicates of each time point together (Fig. 2B and Supplementary Table S3). In order to evaluate the functional diversity of the detected proteins, we classified the total proteins into 26 classes using the PANTHER (PANTHER13.1) classification system — out of 29 indexed parent protein class terms (Supplementary Fig. S2B) [13]. Our data covered a significant number of enriched proteins that included 1,180 enzymes and enzyme modulators, 698 nucleic acid binding and transcription factors (TFs), 425 intra/extracellular trafficking and signaling proteins, 203 cytoskeletal and extracellular matrix (ECM) proteins, and 57 structural and adhesive proteins, indicating the essential role of catalytic activity, gene expression, biosynthesis/trafficking processes and cellular structure in addition to their surroundings, in OL differentiation (Supplementary Fig. S2B).

Oligodendrocyte (OL) lineage differentiation of the human embryonic stem cells (hESCs) is led by the co-operation of three protein clusters

To get a deep understanding of major functional players during the OL lineage differentiation, we explored the dynamic view of the proteome expression during OL differentiation using unsupervised fuzzy c-means clustering on all quantified proteins. As a result, a total of 3,855 proteins (Supplementary Table S3) were segregated into three clusters by their expression trends during differentiation. The clusters' functional enrichment analysis was performed against the Gene Ontology (GO) Biological Process (BP) gene set collection (2018) to ascertain functional groups associated with this differentiation progress (Fig. 3 and Supplementary Table S4).

Cluster 1, consisted of the majority of proteins (2,279), demonstrated a slight decreasing

expression profile (d0 to d120). Based on the functional enrichment analysis, this cluster mostly contained proteins that contribute to gene expression and translation (Fig. 3A and Supplementary Table S4). The two other clusters reflected increasing trends in accordance with the progression and specification of the differentiation process (Fig. 3B and C, and Supplementary Table S4). Both clusters shared some common developmental terms with regard to cellular structure, migration, division, and secretion, that are also in agreement with the OL development; however, cluster 2 with a slightly increasing pattern, seemed to be more involved in early developmental processes by enrichment of the GO terms predominantly related to the regulation of neural stem and progenitor cells (NSPCs), pre-OPCs and OPCs maintenance and differentiation. This was reflected by GO terms, including “vesicle transport” [14], “ephrin (Eph) receptor signaling pathway” [15], “receptor protein tyrosine kinase signaling pathway” [16], “sterol biosynthesis” [17], “nicotinamide adenine dinucleotide phosphate (NADP) metabolic process” [18] and “canonical glycolysis” [18] (Fig. 3B). On the other hand, cluster 3, which indicated an upward trend, mostly on d20 (pre-OPC stage) to d120 (OL stage), revealed enrichment of proteins mainly involved in OPCs differentiation into OLs, OL maturation and myelin formation, such as “gliogenesis”, “positive regulation of calcium ion transport” [19–22], “regulation of filopodium assembly” [23], “regulation of nitric-oxide synthase activity” [24], “response to peptide hormone” [25] and “positive regulation of lipid metabolism” [26] (Fig. 3C). These results indicated that the functional enrichment of the derived proteome architecture was associated with the corresponding differentiation states.

To confirm the authenticity of our clusters, we checked the expression patterns of several marker proteins related to the OL differentiation stages. The depicted heat map showed that the time-dependent changes of these markers was consistent with their fitted clusters and aligned with the progression of the differentiation process (Fig. 3D). Notably, the hESC (d0) markers and regulators of stem cell proliferation, including POU5F (OCT4) and LIN28A, along with NSC (d8) markers SOX2, SOX3, and MSI were grouped in cluster 1, which had a gently decreasing pattern [27–29]. Nevertheless, CDH2, an NSC (d8) marker that is also known to be highly expressed in myelinating OLs [30,31], like OPC

(d80) and OL (d120) markers (PTPRZ, CD9, CNP, and GALC), was classified in cluster 2 (with a slightly increasing trend). Likewise, OPC (d80) and OL (d120) specific proteins, i.e. CNTN1, SIRT2, NDRG1, ACTR1, and GSN were located in cluster 3 (with a sharp upward trend) [29,32–35]. In general, these results revealed the expression distribution of enriched proteins with stage-specific biological functions within our three clusters, which may assist us to identify key proteome signatures associated with OL lineage differentiation. Therefore, this dynamic proteome outlook could support additional discovery of potentially competent proteins for *in vivo* OL differentiation of various neural precursors in patients with myelination defects. It also may provide us with stage-specific profiles that correlate with predominant biological functions associated with this differentiation process.

Canonical and non-canonical Wnt signaling protein profiling during the generation of the oligodendrocyte (OL) lineage cells

Looking through enriched BPs of the clusters, we found the Wnt signaling pathway to be prominently affected (Supplementary Table S4). This pathway has been shown to be involved not only in OL development but also in other developmental processes [36]. We observed enrichment of the GOs related to the regulation of the Wnt signaling pathway, and non-canonical Wnt signaling pathways, particularly planar cell polarity (PCP) pathway in both clusters 1 and 2 (Fig. 3A-, B-left charts and Supplementary Table S4), which seems to have a complementary function in this context. Wnt signaling pathways, including Wnt/β-catenin (canonical) pathway, Wnt/$Ca2^{+}$ (non-canonical) pathway, and PCP (non-canonical) pathway are fundamental mechanisms associated with various levels of vertebrate developmental procedures [37,38].

Systematic analysis of our data by applying DAVID (DAVID 6.8) and UniProt (besides Enrichr, used in clusters functional enrichment analysis) revealed that 147 Wnt signaling related proteins enriched in the OL lineage differentiation process [39–42]. We found that 83 proteins from cluster 1, 56 from cluster 2, and 8 from cluster 3 orchestrated Wnt signaling pathways during OL lineage differentiation (Fig. 4A). According to the heat map illustration of the relative abundances of Wnt signaling related proteins, we noticed that the Wnt signaling

pathways seemed to be highly active in this differentiation process distinctly at the hESCs (d0) and late (OPC and OL) stages (Fig. 4A). In support of our observations in cluster enrichment analysis, we noted that these proteins are mainly involved in GO related to the regulation of both canonical and non-canonical Wnt signaling pathways, especially the PCP pathway (Fig. 4B). To scrutinize the contribution of Wnt signaling components in detail, we also applied Gene Set Enrichment Analysis (GSEA) using whole protein expression profiles. Remarkably, Wnt signaling pathway was enriched especially at three last time points of differentiation (i.e. d20, d80 and d120) compared to the other days (FDR q-value 0.029) indicting the importance of this pathway in OL differentiation (Supplementary Fig S3). In general, this feature supported the results of previous studies that mentioned the crucial implication of canonical Wnt signaling in regulating stemness and development of ESCs; specification, proliferation, and differentiation of OPCs; and maturation and myelination of OLs [43–45]. Even though some observations made canonical Wnt signaling impact on these three cell types baffling, based on the ultimate outcome of all former studies, this cascade’s effect is amenable to the developmental stage, microenvironment, and intensity of the signaling [43,46–50].

The enriched PCP pathway that regulates cell polarity is implicated in cellular morphogenesis, migration, intercalation, and function [51,52]. This non-canonical Wnt signaling pathway has been shown to maintain the NSCs of the subventricular zone (SVZ, located in the periventricular region), in the quiescence state. Chavali *et al.* (2018) demonstrated that a shift in PCP to canonical Wnt activity and keeping their balance induces NSC activation and lineage progression toward the generation of the progenitors that eventually go on to participate in the repair process [53]. It was also well demonstrated by Jarjour *et al.* (2015 and 2020) that the PCP pathway is involved in the myelination initiation, and structural organization of axonal myelin sheath [54,55]. Along with these reports, our finding shows that the high abundance of PCP pathway-related proteins in the middle stages of OL lineage differentiation (Fig. 4A and Supplementary Table S5) may convey the necessity of polarization in OPC specification, migration, and differentiation; which would need additional functional investigation as there cannot be found any study that explored the PCP pathway in pre-OPC and OPC generation and

their differentiation into OL. In addition, significant enrichment of PCP pathway-related proteins in hESCs possibly implies their role in modulating stem cell self-renewal [56].

Briefly, these findings represent non-canonical Wnt signaling pathways, especially the balance between PCP and Wnt/β-catenin pathway, as an enticing field of study in the OL development. The results can potentially improve our knowledge about the implications of all of the Wnt signaling pathways in hESCs maintenance and its further differentiation into the particularly OL lineage; specification, migration and differentiation of OPCs; and OL maturation and myelination, with the ultimate goal of recruiting pre-OPCs and OPCs to the demyelinated regions and achieving their OL differentiation, which may result in restoring myelin regeneration in diseases associated with myelin deficiencies.

Macroautophagy-associated protein profile of the generation of oligodendrocyte (OL) lineage cells

Another set of remarkable BPs in our cluster enrichment analysis was related to autophagy (Supplementary Fig. S4A and Supplementary Table S6), an important lysosomal degradation and recycling pathway in mammalian cell development and differentiation [57]. Autophagy is a substantial issue in developmental processes. Because of the lack of a comprehensive study on the role of autophagy in OL lineage development or differentiation, we sought to peruse the proteome signature of the autophagy pathway through OL lineage differentiation of hESCs.

Autophagy is a highly conserved lysosomal-mediated cellular pathway responsible for catabolism plus recycling of damaged or outlived intracellular cargoes (macromolecules and organelles) to maintain cellular homeostasis and assist cellular structural remodeling during normal development and differentiation. The most common form of autophagy in eukaryotic cells is macroautophagy, which is mainly referred to as autophagy. In this major cellular degradation pathway, double-membrane vesicles (autophagosomes) engulf the cytoplasmic cargos and digest them through the autophagosome-lysosome system [58,59] (Supplementary Fig. S4B).

Further functional analysis of our proteome data using DAVID and UniProt [39,40,60] showed that the proteins involved in autophagy, particularly macroautophagy, were enriched through the OL lineage differentiation process. Our analysis highlighted the BPs engaged in autophagy, macroautophagy, autophagosome formation, and their regulation (Fig. 5). It featured the expressions of key upstream triggers of this pathway, AMP-activated protein kinases PRKAA1, PRKAA2, and PRKAG1 [61] in addition to the proteins involved in the early stages of autophagosome formation, MAP1LC3B2 (a member of LC3s) and PI3KC3 [61]. Our data also showed enrichment of the autophagy-related (ATG) proteins (ATG16L1, ATG5, ATG7, ATG3, ATG2B, and ATG9A) that, in cooperation with LC3s (GABARAPL2 and PI3KC3), control major steps of autophagy, including autophagosome expansion, maturation and lysosomal fusion, as well as cargo recruitment, degradation, and the recycling system [62] (Fig. 5 and Supplementary Fig. S4). Relative expression heat map of the 103 proteins involved in autophagy (found in our data) showed that 77 proteins were members of cluster 2 and 10 proteins were members of cluster 3. This may show the major influence of autophagy and macroautophagy in both specification and function of NPCs, pre-OPC, and OPCs, in addition to OL maturation and myelination (Fig. 5 and Supplementary Table S6). Our results corroborated previous findings that showed the crucial role of macroautophagy in OPC/OL differentiation, survival, maturation, and proper myelin development. In addition, these findings also brought up a possible vital role of this pathway in the early OL developmental stages [63,64]. Furthermore, our data provided a novel proteome profile of autophagy-associated proteins through the OL lineage differentiation process. It revealed proteins related to each cell type (hESCs, NSCs, NPCs, pre-OPCs, OPCs and OLs) and their expression trends in their generation process. Therefore, this may provide researchers with a tremendous repository for a better understanding of the molecular mechanism of autophagy in OL lineage, and designing more effective therapeutic strategies toward demyelination disorders.

Due to its cytoprotective role, autophagy is increasingly believed to promote neuronal and OL survival. However, in a disease like MS (the most well-known example of demyelinating diseases), therapeutic intervention of autophagy gets greatly complicated

since MS is an inflammatory-mediated demyelinating disease, wherein cells of the immune system destroy the myelin sheaths of the nerve axons in the CNS and this is followed by neurodegeneration of both myelinating cells (OPCs and OLs) and neurons [65]. While studies show that pharmacological inducers of autophagy, like rapamycin, can improve myelination of OLs and Schwann cells (SCs; the myelinating glia of the peripheral nervous system), the elevated levels of autophagy in immune cells of MS patients makes this type of medications hazardous [63,66–69]. Nevertheless, our proteome data can be considered as a valuable source for finding an appropriate way of the targeted drug delivery system for these types of medications [70]; particularly with the aim of directly inducing autophagy in OPCs and OLs. Therefore, the induced autophagy would promote these cells function in clearing cellular and myelin debris, protein aggresomes, and their development toward remyelination achievement [64,70,71]. Currently, the pharmaceutical compounds which are used for autophagy induction may target signaling pathways other than autophagy [72]. One potential strategy to minimize the undesirable side effects is to identifying more specific autophagy regulators and mechanism in every cell type would help to achieve targeted autophagy modulations [72,73].

Identification of potential biomarkers in each step of human embryonic stem cell (hESC) differentiation into oligodendrocytes (OLs)

In this study, we used TMT-based quantitative proteomics to discover important proteins involved in OL lineage differentiation. Furthermore, we exploited this approach to identify novel potential biomarkers that improve the selection, tracking and monitoring of each specific cell type of OL lineage. Currently, the majority of the markers in OL lineage are not specific to one cell type or one differentiation stage. Hence, the expression pattern of a panel of markers during differentiation process is usually being used, especially in *in vitro* studies [29]. For instance, LIN28A a known marker of ESCs (Figure 3D) is also a marker of stemness which is highly expressed in NSCs [74–76]. This is also true for MSI1, a marker of NSPCs, which is also a well-known marker for adult stem cells and progenitors in hair follicles, mammary glands, and intestine [77–80]. MSI1 is also considered as a prognostic biomarker in various human malignancies [81]. Thus, we

investigated the identification and abundance of proteins that differentially expressed only in one of the differentiation stages. We observed significant (>2-fold) expression changes in the numbers of differentially expressed proteins (DEPs) in the hESC (d0, n=4), NSC (d8, n=57), NPC (d12, n=9), pre-OPC (d20, n=22), OPC (d80, n=24), and OL (d120, n=251) differentiation stages (Fig. 6, Supplementary Fig. S5 and Supplementary Table S7).

According to Our DEP analysis, four proteins (HMOX1, MT1E, MT2A and ASNS) showed their specificity at hESC (d0) state (Fig. 6A and Supplementary Fig. S5), all of which are cytoprotective factors [82–84]. Among them, the specific role of HMOX1 (Heme oxygenase-1) in the maintenance of self-renewal and pluripotency of ESCs and induced pluripotent stem cells (iPSCs) is well-studied [85–87]. Even though the roles of MT1E and MT2A in ESCs are still not specifically studied, emerging evidence shows the remarkable expression of these metallothioneins in ESCs and iPSCs [88–90]. ASNS (asparagine synthetase), another deferentially up-regulated protein in hESCs, is an ATP-dependent enzyme that synthesizes asparagine (Asn) and glutamate (Glu) at the expense of aspartate (Asp) and glutamine (Gln) [91]. Glu can be used in Gln synthesis by glutamine synthetase (GLUL), an enzyme which has been shown to be involved in cell proliferation, and demonstrated a considerable expression in all steps of OL lineage differentiation (data not shown) [92]. On the basis of previous studies, Gln and Asn act as principal survival and self-renewal factors in ESCs and cancer cells, respectively [91,93,94]. Ryu *et al.* have reported that Gln is an important factor in regulating maintenance of mouse ESCs through transcription regulation via the Akt, PKCe, and mTOR signaling pathways [95]; while Krall *et al.* have revealed that Asn is a powerful regulator of cell amino acid homeostasis, therefore it controls mTOR complex 1 (mTORC1) activation, cellular anabolic metabolism and proliferation [91]. Consequently, this data suggests that the four DEPs (HMOX1, MT1E, MT2A and ASNS) can be efficient biomarkers for ESCs.

As mentioned above, we found 57 proteins that were specifically related to NSCs (Fig. 6B and Supplementary Fig. S5). Most of these proteins, like SOX2, CBX2, CBX5, HMGB2, RHF6, RBMS1 and SALL1 (all of which has been already reported as NSC specific gene by Xiao *et. al.*) [96] are involved in BPs related to chromatin organization

and gene expression, reflecting the needed trigger for the differentiation onset. Epigenetic modification of chromatin, in response to differentiation cues, controls gene expression in different cellular transitions, such as the differentiation of hESC into NSC [97]. The identified NSC state-specific proteins, like LUZP1 [98,99], SOX2, PPT1 [100], SMOC1 [101], MAZ [102,103], CRABP1 [104], GKAP1 [105] and CSRP2 [106], are also involved in BPs associated with cell division, proliferation and differentiation of specially nervous system. These findings demonstrate that NSC state-specific proteins (Fig. 6B and Supplementary Fig. S5) can be used as biomarkers of active NSCs.

As illustrated in Fig. 6C, there are only 9 proteins with specifically high abundances at the NPC stage (Fig. 6C and Supplementary Fig. S5). These proteins are mainly associated with cellular junction, adhesion, mitosis, proliferation as well as cytoskeleton proteins [107], however, apart from FREM2 [108,109], the roles of the other proteins in NPC maintenance and function are not well documented [110]. According to previous studies, that highlighted the importance of cell-cell connections in NSPCs biological behavior [111–113], our data may bring the three differentially expressed desmosomal proteins, DSP, JUP and PKP2, into the spotlight for further investigation of the impact of the desmosome junction on providing more desirable niches for NPCs maintenance, proliferation and differentiation. These findings may lead to the introduction of new potential biomarker proteins for NPC state.

The two most highly DEPs (mhDEPs with Log2 (fold-Change) ≥ 5) on d20, MDK and RBP1, truly reflect the role of this leading step, the pre-OPC stage, in OL generation. Both mhDEPs instigate pre-OPC specification toward OL differentiation [114–117]. Among the 22 DEPs at the pre-OPC stage (Fig. 6D and Supplementary Fig. S5), there are 10 members of histone family (H1F0, H1FX, H2AFX, H2AFZ, HIST1H1B, HIST1H1C, HIST1H1D, HIST1H2BM, HIST1H3A and HIST2H2AB) involved in chromatin organization and show the dynamics of chromatin interaction through this differentiation state. This finding may indicate the significance of histone mark repatterning and remodeling of the chromatin architecture at the NPC stage (Supplementary Fig. S5). The correlation of this differential re-patterning with the high abundance of known pre-OPC proteins such as MDK, RBP1 [114–117], SOX3 [118], and TNFRSF10B [119] points to

the potential of these d20 DEPS to be used as biomarkers for the pre-OPC state.

Our findings also represent 24 DEPs at OPC state (Fig. 6E and Supplementary Fig. S5), four (FN1, TGFBI, TNC and COL3A1) of which have been previously reported as DEPs of OPC by Chaerkady *et al.* [120]. FN1 (Fibronectin) is a glycoprotein of the extracellular matrix (ECM) that stimulates OPC proliferation [121], also has the capacity to impair oligodendrocyte differentiation and myelin sheath formation [122–124]. In OPC, FN1 with PDGFA accompaniment leads to the actin-pERK1 and 2 co-localization and formation of filopodia, thus enhances the migration of these cells [125]. The impact of the ECM protein, TGFBI is not completely clear; however, it is known that TGFBI can regulate cellular adhesion and migration [126]. TNC, another ECM-glycoprotein, is produced by OPCs and preserves their proliferation. Down-regulation of TNC is followed by OL maturation [127–129]. There is no information about the role of COL3A1 in maintenance or function of OPCs; however, Gao *et al.* have reported that COL3A1 is a valid biomarker for diagnostic or therapeutic strategies for glioblastoma [130]. In fact, this protein may put its impact on the maintenance of OPC's stemness. Among the other worth noting proteins at OPC state, there are three mhDEPs - GAP43, ATP2B2 and PCSK1N. GAP43 is a membrane protein in OPCs, and its expression decreases during OL differentiation [131]. ATP2B2, a magnesium-dependent enzyme that catalyzes the hydrolysis of ATP coupled with calcium transport, is a known constituent of the CNS myelin proteome [35] but its function in OPCs remains to be investigated. PCSK1N, a neuropeptide [132], is a proliferation-related molecule [133]. There is no evidence that directly focuses on the role of PCSK1N in OPCs function or maintenance; however, it has been reported that, in mouse insulinoma 6 (MIN6) cell line, Pax6 can directly down-regulate *Pcsk1n* expression [134]. In addition, it is shown that, in the embryonic chick neural tube and spinal cord, Pax6 promotes OPC migration [135], the process which is usually followed by Oligodendrocyte generation. Therefore, it can be hypothesized that PCSK1N may support the proliferation of OPCs. All in all, the company of the DEPs that are associated with cell proliferation and migration can indicate their utility as potential biomarkers at the OPC stage (d80).

Finally, we identified 251 DEPs on d120 (Fig. 6F and Supplementary Fig. S5), among which the transcripts of 40 proteins in the OL state have been previously reported: 4

transcripts by Hu *et al.* [136], 18 transcripts by Najm *et al.* [129], and 25 transcripts by Lager *et al.* [137]. Enrichment analysis of the 251 specifically up-regulated proteins in the OL state by Enrichr [41,42], showed that these proteins participate in OL developmental-related BPs such as ECM and cytoskeleton organization (*e.g.*, BCAN, CD44, CDK5, FMNL2, HTRA1, ITGB4, PFN2 and TGFB2), nervous system development and OL differentiation (*e.g.*, BCAN, CDK5, CNP, DPYSL2, FYN, MAPK1 and LSAMP), post-translational regulation of gene expression (*e.g.,* CRYAB, INS, LUM, PACS2, RRAGA, SEPT3 and TGFB2), L-glutamate transport (*e.g.,* ARL6IP5, PRAF2 and SLC1A3) and fatty acid metabolic process (*e.g.,* ABCD3, ACOX1, ACADS, BDH2, CRAT, CRYL1 and DECR1). According to neXtProt (v2.24.0), amongst these 251 DEPs, there are proteins associated with myelination such as TSPAN3, NDRG1, CA2, EPB41L3, CLU, GALC, ANXA2, GPC1 and AKT1 [107]. In addition, amongst the 40 DEPs that our study shares with the above mentioned works [129,136,137], there are six proteins with well-known functions in promoting OPC development into mature OL: CNTN1 [33], SIRT2 [32,138], TPPP [139], TGFB2 [140], CNP [141] and CDK5 [142]. These findings show the tremendous potential use of the identified DEPs as OL differentiation inducer, and potential biomarkers for diagnostic and prognostic purposes.

Therefore, our study, the first extensive step by step proteomic profiling of hESC differentiation into OL lineage, reveals several novel proteins that are potentially a part of this differentiation process and OL generation. These results propose a total of 378 potential biomarkers for every cell type of this lineage.

Coordinated proteome dynamics of the stage transition specific proteins (STSPs) throughout oligodendrocyte (OL) lineage differentiation

Due to the time-series nature of our study, which enables a deeper insight into the development of every cell type in the OL lineage, subsequently, we sequentially compared pairwise stages towards their achievements by identifying stage transition specific proteins (STSPs) (Fig. 7 and Supplementary Table S11). Comparative pairwise analysis of differentiation stages showed distinct sets of STSPs (PS 1-14) that

meticulously escort sequential steps of this differentiation process (Fig. 7F). At first glance, our results distinctly highlight the weighty role of transition between hESC (d0) – NSC (d8), and pre-OPC (d20) – OPC (d80) by up-regulation of 129 and 152 proteins respectively (Fig. 7A and D). It also displays a considerable transition from NSC (d8) to NPC (d12), NPC (d12) to pre-OPC (d20), and OPC (d80) to OL (d120) by down-regulation of 41 and up-regulation of 38 and 40 proteins respectively (Fig. 7B, C and E).

Rolling down across the Waddington landscape toward NSCs (d8) generation, hESCs (d0) go through the significant down-regulation of HMOX1 and ASNS, two potential hESC (d0) biomarkers (discussed in **Identification of Potential Biomarkers in every step of hESC differentiation into OL**), as well as up-regulation of 129 proteins which may be divided into 2 protein sets (PSs; PS 2 and 3) (Fig. 7F). PS 2 (includes 93 up-regulated proteins on d8) and PS 3, respectively comprise 26 and 29 potential NSC biomarkers. As previously mentioned, on d8, we found 57 NSC stage-specific proteins, 55 of which also show their stage transition specification. The up-regulated STSPs of the NSC (d8) stage also share 54 differentially expressed proteins with those DEPs found in fibroblast-derived NSC reported by Xiao *et. al.* [96]. A number of them are known to be associated with NSC development and function such as SMOC1 [101], SOX2, CRABP1 [104], CSRP2 [106], CRABP2 [143], CTNND2 [144], SOX3, MSI1 [145], HMGB2 [146] and LAMB1 [147]. Moreover, among the 129 up-regulated STSPs at NSC (d8) stage, 36 STSPs (PS 3) show a transient trend as their abundances decrease while approaching the NPC (d12) state. In parallel to the 36 transient STSPs, there are 5 down-regulated STSPs (PS 4) composed of PBDC1, LSM14B, CIC, LSM12, and PHPT1 in addition to 5 up-regulated STSPs (PS 5 and 6) toward reaching the NPC (d12) state (Fig. 7F). The high abundance of STSPs at the NPC (d12) stage include two well-known NPC proteins, MEST (NPC marker) [148,149] and FERM2 (morphogenetic rearrangement protein of the ECM that has a crucial role in neural tube closure) [108], in addition to three intermediate filament family members (KRT19, KRT7, and KRT8). The expression levels of these STSPs, which have been also represented here as the NPC stage-specific DEPs (Supplementary Fig. S5), show a differential reduction shortly after NPC achievement, with the exception of MEST, which first displayed a gentle decrease reaching the pre-OPC (d20) stage, followed by a

dramatic reduction during the pre-OPC (d20) to OPC (d80) conversion. These findings, in addition to those reported by Najm *et. al.*, who monitored the overexpression of MEST in pluripotent mouse epiblast stem cell (EpiSC)-derived OPCs, indicated the significance of MEST in cell fate switching from pluripotent NSCs to pre-OPCs, as more specific OL progenitors.

Taking the principal step towards more committed cells of the OL lineage, 12 highly expressed proteins (STSPs at PS 6 and 7) in the NPC (d12) stage underwent differential down-regulation while the expression levels of 38 STSPs (PS 8 and 9) increased, which promoted entrance of these cells into the pre-OPC (d20) stage (Fig. 7F). At this point, down-regulation of NPC (d12) related STSPs such as FREM2 [108], DSP [150], TPP1 [100], KRT7 [151], KRT8 [152,153], and KRT18 [154] were accompanied by up-regulation of epigenetic factors and transcription regulators (*e.g.*, MDK, HIST1H1C, HIST1H1E, H1FX, H2AFX, HMGA2, HIST1H3A, H1F0, SOX3, H2AFZ, HIST1H1B, H2AFY, HIST2H2AB, TAGLN3 and HIST1H2BM [107]) and some well-known pre-OPC STSPs (*e.g.*, MDK, RBP1 [114–117], DCHS1 [155], SOX3 [118], MAP1B [156], DPYSL4, DPYSL5 [157,158], TF [159], and TNFRSF10B [119]) (Fig. 7F). After succeeding the requisite regulation of the chromatin remodeling and gene expression, the abundance of the mediator STSPs, *e.g.*, MDK, HMGA2, HIST1H1C, SOX3, H2AFX, H1FX, HIST1H3A, H2AFZ, HIST1H1B, HIST2H2AB, H1F0, and HIST1H2BM (the transition STSPs, settled in PS 9), along with MEIS1, HIST1H1D, CRABP1, HMGA1 and HIST1H4A (members of PS 10) [107]) decreased distinctively (Fig. 7F), while pre-OPCs (d20) proceeded towards the specified OPCs (d80). Moreover, the fate specification of the cells into OPC (d80) stage was assisted with the up-regulation of 152 STSPs (PS 11 and 12) (Fig.6F), which are involved in BPs such as cellular proliferation and its regulation (*e.g.*, IGFBP7, FABP7, TNC, GNG2, CRIP2, CD47, and PRKCA), cellular differentiation and development (*e.g.*, GAP43, MAPT, VIM, GPC1, GSN, SLC1A3, and SIRT2) and cellular migration and regulation (*e.g.*, TSPAN3, L1CAM, FN1, NTN1, LMNA, RUFY3 and SULF1) [107]. Amid these up-regulated proteins (PS 11) there was a subset of STSPs (TPPP3, FHL1, GNAO1, GPM6B, DCLK2, TMOD1, CRYL1, CAVIN4, FTL, SLC27A1, CAMK2G, TSPO, PLEC, DES, TMEM65 and SIRT2), that underwent an additional significant up-regulation

during OPC (d80) conversion into OL (d120) stage (Fig. 7F). These STSPs which were shared between PS 11 and PS 14, participate in cytoskeleton organization (*e.g.*, TPPP3, GPM6B, TMOD1, DCLK2, DES and PLEC [107]) a pivotal BP in OPC generation, differentiation, and migration in conjunction with OL generation and myelination. These STSPs are also involved in other OPC and OL BPs that are necessary, such as regulation of cell proliferation and cell cycle (FHL1, SIRT2, and TSPO), cell differentiation (FHL1, CAMK2G, and CAVIN4), nervous system development (CAMK2G, GPM6B, DCLK2, SIRT2, and GNAO1), and in BPs that are implicated in the production of proteins (TPPP3, GPM6B, DCLK2, SLC27A1, SIRT2, TSPO, and GNAO1) and lipids (CRYL1, GPM6B, DCLK2, SLC27A1, SIRT2, and TSPO), which are two main components of myelin [107]. We found that OPC (d80) differentiation was accompanied by selective down-regulation of the 13 STSPs, 9 of which showed transient differential expressions at this stage (Fig. 7F). Among the transient STSPs, GAP43 and ATP2B2 were the top two most highly differentially expressed STSPs at OPC (d80) stage, with log2 (fold-Change) equal to 12.73 and 11.57, respectively. GAP43 is an OPC protein, and its expression has been shown to decrease and reach a plateau, during OPCs differentiation into OLs [160,161]. Although GAP43 is a calmodulin (CaM)-binding protein, like ATP2B2, GAP43 phosphorylation reduces its affinity for CaM. Hence, the interaction between GAP43 and CaM controls the availability of CaM [162], which then regulates the calcium pump activity of ATP2B2, and thus GAP43 takes part in the regulation of OPC maintenance and differentiation [163–165]. PS 14 is another subset of STSPs that promotes this prolonged differentiation process into its final OL target. PS 14 has 40 members, 16 of which are shared with PS 11 (as mentioned above) (Fig. 7F). All 40 STSP members of PS 14 are part of the previously mentioned OL stage-specific proteins. For instance, the merits of INS [166,167], RRAGA [168], GALC, GPM6B [169], PLEC [170], and SIRT2 [32,138] for the OL (d120) stage have been already discussed.

Consequently, these results demonstrate the outstanding potential of STSPs to improve the status of OL lineage differentiation at each of the differential stages and shed light on future mechanism based developments of demyelinating disorder associated strategies.

Methods

Human embryonic stem cell (hESC) differentiation into oligodendrocyte (OL) lineage cells. Adherent confluent RH6 cells (passages 45, 48 and 50, to accommodate the three biological replicates) were induced into $SOX1^+$ NSCs by dual inhibition of SMAD signaling [171,172]. Under the treatment of 10 µM SB431542 (SB, inhibitor of TGF-β/activin/nodal signaling), 250 nM LDN193189 (LDN, inhibitor of bone morphogenetic protein signaling) and 100 nM all trans-retinoic acid (RA, caudalizing patterning agent) for 8 days, nearly all the differentiated cells were $SOX1^+$ NSCs (Supplementary Fig. S1A and C). Next, in order to mimic the embryonic ventral spinal cord environment (pMN domain) and achieve pre-OPC, 100 nM RA and 1 µM smoothened agonist of sonic hedgehog (SAG, ventralizing patterning agent) were applied for 22 days. By day 12 of differentiation, NSCs gave rise to $OLIG2^+$ NPCs (Supplementary Fig. S1D), which were then detached for sphere aggregation; this enriched the $OLIG2^+$ population. By day 20 of differentiation, the $OLIG2^+$ progenitors committed to the OL lineage by co-expressing NKX2.2 (pre-OPCs; Supplementary Fig. S1E). On day 20, supportive reagents for pre-OPCs expansion and maturation toward OPCs, and further OPCs expansion and maturation toward OL producing OPCs, *i.e.* 10 ng/ml platelet-derived growth factor AA (PDGF-AA), 5 ng/ml hepatocyte growth factor (HGF), 10 ng/ml insulin-like growth factor 1 (IGF1), 10 ng/ml neurotrophin 3 (NTF3), 60 ng/ml 3,3,5-Triiodo-l-thyronine (T3, thyroid hormone) , 25 µg/ml insulin, 100ng/ml biotin, and 1µM cyclic adenosine monophosphate (cAMP) were added to the culture medium for 60 days (Supplementary Fig. S1A). On day 30, spherical aggregates were plated onto poly-L-ornithine/laminin-coated (pO/L) dishes and on day 80 $PDGF^+$ OPCs were generated (Supplementary Fig. S1F). In order to eliminate the neurons and astrocytes that migrated out of the spherical aggregates and to achieve a homogenous population of OPCs, we re-plated the cells twice onto poly-L-ornithine/laminin-coated dishes, on day 65 and on day 75 of differentiation. On day 80, the growth-factors were withdrawn from the culture medium and OPCs were differentiated into MBP^+ OLs in the presence of 10 mM HEPES buffer and 20 µg/ml ascorbic acid (AA, supportive for OL differentiation), in addition to T3, insulin, biotin, and cAMP (Supplementary Fig. S1A and G, Supplementary Fig. S6 and Supplementary Table S9) [6,7,173].

Immunostaining. Cells were washed 3X in PBS$^-$ (Life Technologies, cat. no. 10010023) for 3 min and then fixed with 4% (w/v) paraformaldehyde (PFA) for 15 min at room temperature (RT). Following fixation, the cells were washed 3X in 0.1% PBS$^-$ Tween (PBS$^-$ that contained 0.1% Tween 20) for 3 min and stored at 4°C. At the time of staining, cellular membranes were permeabilized by 0.5% PBS$^-$ Triton (PBS$^-$ that contained 0.5% Triton X-100) for one hour at RT. Then, the cells were incubated in blocking solution that consisted of 0.1% PBS$^-$ Triton, 0.2% donkey serum, and 0.2% bovine serum albumin (BSA) for one hour at 37°C. After washing 3X with washing solution (0.1% PBS$^-$ Tween), the primary antibodies were applied overnight at 4°C (for antibodies information, please refer to Supplementary Table S10). The next day, these cells were washed 3X in a washing solution for 10 min and stained with a secondary antibody for 45 min at 37°C (for antibodies information, please refer to Supplementary Table S10). Thereafter, the cells were washed 3X with washing solution for 10 min, counterstained with DAPI at RT, and washed in washing solution. Images were captured using an Olympus IX71 inverted fluorescent microscope equipped with an Olympus DP72 Digital Color Microscope Camera.

Protein isolation. Total protein extraction was performed using TRIzol™ reagent. Cells were washed with PBS, detached mechanically, pelleted by centrifugation, and snap frozen in liquid nitrogen before they were stored at -80°C. For each differentiation stage, we pulled three plates of cultured cells. At the time of isolation, the cell pellets were lysed and homogenized with TRIzol™ reagent according to the manufacturer’s instructions. Then, chloroform was used for phase separation. The samples were centrifuged for 15 min at 12000 ×g at 4°C and the clear upper aqueous phase was used for RNA isolation, the white interphase was discarded, and protein extraction proceeded by the red organic phase. Cold (-20°C) 100% ethanol was applied to dissociate DNA from proteins trapped within the lower red phenol-chloroform phase**.** Thereafter, the dissolved proteins were precipitated by an overnight incubation in acetone at -20°C. The following day, the total proteins of each sample were precipitated via centrifugation and the resultant pellets were washed with washing solution one that contained 1 ml 0.3 M guanidine hydrochloride in 95% ethanol and 2.5% glycerol, and washing solution two comprised of 100% cold

ethanol that contained 2.5% glycerol. The resultant pure protein pellets were air-dried and solubilized in lysis buffer that contained 7 M urea, 2 M thiourea, 4% CHAPS detergent, 50 mM dithiothreitol, and protease and phosphatase inhibitor cocktails. In the end, the protein samples were stored at -70℃ (for provider information, please refer to Supplementary Table S11).

Protein preparation. The isolated protein samples were thawed at 4℃. The concentrations of the total proteins were determined by a spectrophotometer using a modified Bradford dye-binding method [174] and BSA as the standard. A total of 300 µg of each protein sample was reduced with 5 mM dithiothreitol (Sigma-Aldrich, cat. no. D0632) for 30 min at 56℃. Thereafter, an alkylation agent, iodoacetamide (Sigma-Aldrich, cat. no. I6125), was mixed with the reduced proteins at a concentration of 14 mM and samples were left at RT for 30 min in the dark. The process was followed by another reduction procedure. This time, after mixing 5 mM dithiothreitol with alkylated protein samples, the mixtures were kept at RT for 30 min. Subsequently, for protein shipment, protein samples were stored at -70℃ for 24 h. After 24 h, the protein samples were lyophilized for 48 h at about -50℃. Next, the lyophilized proteins were shipped in a pack filled with silica gel beads to prevent any probable dampness.

In order to remove the interfering detergents and contaminants, the proteins were precipitated using the methanol-chloroform protocol [175]. After sequential addition of ice-cold methanol, chloroform, and water with vortexing intervals, the samples were centrifuged for 2 min at 1000 ×g at 4°C. The protein aggregate at the interface layer was washed with ice-cold methanol and acetone. Next, the protein pellet was air-dried and then resuspended in 200 µl of 8 M urea in 50 mM Tris (pH 8.8). Eventually, the protein concentration was determined by a Bicinchoninic Acid (BCA) Assay Kit (Pierce, Rockford, LI, USA) using BSA as the standard.

In the end, dual digestion was conducted on 150 µg of each protein sample, first with Lys-C (Wako, Japan) at a 1:100 enzyme:protein ratio overnight at RT, followed by trypsin (Promega, Madison, WI, USA) digestion at a 1:100 enzyme:protein ratio for at least 4 h at 37°C. The reactions were stopped using trifluoroacetic acid (TFA) to a final

concentration of 1% (pH 2 to 3). Peptide yields were desalted by SDB-RPS (3M-Empore) Stage Tips and the eluted peptides were dried by vacuum centrifuge, then the dried peptides were reconstituted in 200 µl of 200 mM HEPES (pH 8). Next, the peptide concentration was measured using the Micro BCA™ Protein Assay Kit (Thermo Scientific, Rockford, IL, USA) and 70 µg from each sample was aliquoted for labeling in a 10-plex TMT reaction [176].

Tandem mass tag (TMT) labeling. To accommodate four biological replicates from seven different sampling points (0, 8, 12, 20, 50, 80, and 120 days) we performed three interdependent TMT experiments. Each TMT experiment contained the same technical replicate (d0) as a common reference and at least one biological replicate per sampling point as illustrated in the Figure 1. The remaining two empty channels in each TMT experiment were assigned to the 4th biological replicate per sampling points. We have made the experimental design clear by providing a table containing the exact details of the labels and sample identifications (Fig. 1). However, in our preliminary PCA analysis, we noticed inconsistency with our 4th replicate (unknown reason); therefore, we decided to present the most correlated triplicates for the further analysis. Furthermore, day 50 (early-OPC stage) was not further considered in the analysis due to its remarkable similarity to day 80 (OPC stage) in our preliminary Pearson correlation coefficient analysis (explained in "**TMT data analysis**" section).

The TMT labeling was carried out as previously described [176–178]; we added 41 µl of anhydrous acetonitrile to each 0.8 mg label vial, followed by occasional vortexing for 5 min and brief centrifugation. Ten TMT labels (Thermo, San Jose, CA, USA) were added to the 10 individual protein samples in each experiment. Labeling was performed for 1 h at RT with occasional vortexing. To quench any remaining TMT reagent and reverse the tyrosine labeling, 8 µl of 5% hydroxylamine (Sigma-Aldrich) was added to each tube, followed by vortexing and incubation at RT for 15 min. For each of the respective 10-plex experiments, all 10 labeled samples were combined in a clean 2 ml Eppendorf tube and then dried via speed vacuum centrifugation. The dried peptide mixture was reconstituted in 1% formic acid (FA, pH approximately 2–3) and desalted on a 130 mg C18 Sep-Pak

(Waters, Milford, MA, USA) as previously described [179], and eventually dried down again using speed vacuum centrifuge.

Fractionation. To reduce the complexity of the mixture, offline strong cation-exchange (SCX) fractionation was carried out for each of the TMT experiments. The labeled peptides were resuspended in strong cation exchange buffer A (5 mM KH_2PO_4 and 25% v/v acetonitrile [ACN], pH 2.72), and were injected onto a PolySULFOETHYL A™ column (200 mm × 2.1 mm, 5 µm, 200 Å; PolyLC, Columbia, MD, USA), which was also equilibrated with buffer A. The adsorbed peptides were eluted with a linear gradient of 10–45% buffer B (5 mM KH_2PO_4, pH 2.72, 350 mM KCl, 25% ACN) for 70 min, which was then rapidly increased to 100% buffer B for 10 min at a flow rate of 300 µl/min. The collected samples were then desalted using SDB-RPS Stage Tips, dried by vacuum centrifuge, and reconstituted in 40 µl of 0.1% FA in preparation for nanoflow Liquid Chromatography Electrospray Ionization Tandem Mass Spectrometry (nanoflow LC-ESI-MS/MS) as reported by Mirzaei *et al.* [176].

Nanoflow LC-ESI-MS/MS of tandem mass tag (TMT)-labelled peptides. Fractionated peptide samples were analysed on a Q Exactive Orbitrap mass spectrometer (Thermo Scientific, San Jose, CA, USA) coupled to an EASY-nLC1000 nanoflow HPLC system (Thermo Scientific, San Jose, CA, USA) as described previously [179]. The peptides were separated on an in-house packed reverse-phase column (75 µm inner diameter × 100 mm, C18 HALO column, 2.7 µm bead size, 160 Å pore size, Advanced Materials Technology). For sample elution, fractionated labeled peptides were run for over 170 min on a linear gradient of 1–30% solvent B (99.9% ACN/0.1% FA). The Q Exactive mass spectrometer was operated in the data-dependent acquisition (DDA) mode to automatically switch between full Orbitrap MS and ion trap MS/MS acquisition. Survey full-scan MS spectra (from m/z 350–1850) were received at a precursor isolation width of 0.7m/z, resolution of 70000 at m/z 400 and an Automatic Gain Control (AGC) target value of 1×10^6 ions. For identification of the TMT labeled peptides, the 10 most abundant ions were selected for higher energy collisional dissociation (HCD) fragmentation. HCD normalized collision energy (NCE) was set to 35% and fragmentation ions were detected in the Orbitrap at a resolution of 70000. Dynamic exclusion of target ions (selected for

MS/MS) was set to 90 s and for accurate mass measurement, the lock mass option was enabled using the polydimethylcyclosiloxane ion (*m/z* 445.12003) as the internal calibrant [180].

Data processing and protein identification. MS raw data were generated by Xcalibur software (Thermo Scientific) and processed with Proteome Discoverer V1.3 (Thermo Scientific, San Jose, CA, USA). Peptide identification was performed using a local MASCOT server V2.3 (Matrix Science, London, UK). The MS/MS spectra were searched against the reviewed UniProt *Homo sapiens* protein database (20,352 sequences-August 2018) [10]. The following parameters and adjustments were used: MS^1 tolerance was set to ±10 ppm precursor. A limit of 0.1 Da was applied for MS/MS fragment ion tolerance and trypsin was set as cleavage specificity that allowed only one missed cleavage. Carbamidomethylation of cysteine was set as a fixed modification and TMT modification of peptide N-termini plus lysine residues, oxidation of methionine and deamidation of Asn and Gln residues were set as variable modifications. For deconvolution of the high-resolution MS^2 spectra, only peptides with a score >15 and below the Mascot significance threshold filter of $P = 0.05$ were included in the search results. Single peptide identifications required a score equal to or above the Mascot identity threshold to be incorporated in the search results. Protein grouping was enabled such that when a set of peptides in one protein were equal to, or completely contained within the set of peptides of another protein, the two proteins were confined in one protein group. Proteins with at least two unique peptides were regarded as confident identifications. Hence, search results were further filtered to retain proteins that had q values (FDR) of <1% and only master proteins assigned via the protein grouping algorithm were retained.

The mass spectrometry proteomics raw data files, database search results, and TMT ratios can be retrieved via the ProteomeXchange Consortium [11] through the PRIDE partner repository with the dataset identifier PXD017649. In each TMT experiment, relative quantitation of proteins was achieved by pairwise comparison of TMT reporter ion signal to noise (S/N) ratios as the ratio S/N of the labels for each of the differentiation time points versus the labels of the internal control (labelled with TMT 126 reagent) of the corresponding run (Supplementary Table S2).

TMT data analysis. Relative quantitation of all protein abundances, in every differentiation time points, with respect to the reference (labelled with TMT 126 reagent), were extracted from the three TMT experiments and were aggregated into a single report (Supplementary Table S2). Overall data quality was first checked by the analysis of variance (ANOVA) adjusted p-value ≤ 0.05 (Supplementary Table S2). Further data analyses and visualization were generally performed with home-made programs developed in the R statistical computing environment [181]. Proportional Venn diagram comparing the depth of protein coverage (identified proteins) in three TMT mass spectrometry experiments (Supplementary Fig. S2A) was created by 'venn.diagram' function from the 'VennDiagram' package [182]. For each time point, the pairwise correlation of the replicates was computed by the Pearson correlation coefficients (PCCs) method through the 'cor' function; then in order to incorporate the highest correlated replicates into our study, we decided to present the most correlated triplicates for the further analysis (Supplementary Table S2). The proteins that were only quantified in some time points of one TMT experiment, the proteins that were quantified in only one TMT experiment, and the proteins that were quantified in two TMT experiments, but not in all time points were excluded from the study. Then, 'missForest' function from R package 'missForest' [12,183] was applied for the imputation of those identified proteins that were quantified in all time points of two TMT mass spectrometry experiments and their relative expressions were not quantified in some or all time points of the other TMT experiment. As a result of this supervised approach, the missing expression of 336 proteins was imputed and the "Quantified Proteins" table in Supplementary Table S3 was achieved. To illustrate the diversity of the quantified proteins (Supplementary Table S3), protein classification in Supplementary Figure S2B was performed using the PANTHER classification system (http://pantherdb.org/; Supplementary Table S12) [13]. In order to visualize the correlation of the analysed samples, in R, 'cor' function with the 'pearson' method argument was applied (on "Quantified Proteins" table in Supplementary Table S3) for Pearson correlation coefficients (PCCs) analysis, and 'pheatmap' function from the 'pheatmap' package [184] was used for the illustration of the results in Fig. 2A. The clustering_distance_rows argument in 'pheatmap' function was set as 'correlation' (at this point we noticed the remarkable similarity between samples of day 50 and day 80, thus

as day 80 contained a more mature OPCs, we decided to put the data of day 50 out of the current study). With the aim of finding the best summary of our dataset (Supplementary Table S3), we performed principal component analysis (PCA), by first scaling the expression values in R, using ‘scale’ function and then applying ‘prcomp’ function. In this regard, we used ‘rotation’ matrix as a PCA loading matrix, and visualized the linear combination of PC1 and PC2 by ‘ggplot’ function from R package ‘ggplot2’ (Fig. 2B and Supplementary Table S3). Biological DataBase network (bioDBnet) was used for conversion of accession IDs into HGNC Symbols (protein names) (https://biodbnet-abcc.ncifcrf.gov/db/db2db.php) [185]. The averages of the relative protein expression of all quantified proteins, in three replicates, were calculated by the ‘mean’ function and the following analyses were performed on the achieved dataset (Supplementary Table S3) [181]. In order to cluster the data and investigate co-regulated proteins associated with similar BPs, c-means unsupervised fuzzy clustering algorithm was performed using the ‘cmeans’ function of the package ‘e1071’. Maximum 100 iterations were considered while the degree of fuzzification was set to 2. c-membership values denote the similarity of the data points to each of the cluster centers [186] (Fig. 3A–C and Supplementary Table S5). Then, the protein members of each cluster were subjected for Gene Ontology (GO) analysis using Enrichr (https://amp.pharm.mssm.edu/Enrichr/) and an adjusted p-value cut-off of below 0.05 was used to filter the results [41,42] (Fig. 3A–C and Supplementary Table S6). For the visualization, some of the overrepresented BPs of each cluster were selected and presented with bar plots showing the −log10 of the adjusted p-values of significantly enriched BPs (Fig. 3A–C). Heat map illustrations of the relative protein expression changes of the marker proteins in Fig. 3D was conducted by ‘pheatmap’ function from the ‘pheatmap’ package, while the ‘scale’ and ‘clustering_distance_rows’ arguments were set at ‘row’ and ‘correlation’, respectively [184]. Noticing the participation of some OL lineage proteins in Wnt signaling and autophagy pathways (Supplementary Table S6), next we investigated single protein indexed GOs by UniProt (https://www.uniprot.org/, based on reviewed indexed data) database [40], and confirmed the result by neXtProt (https://www.nextprot.org/) [107], and David gene annotation tool v6.8 (https://david.ncifcrf.gov/) [39] databases. Then, we filtered the proteins involved in these two pathways and put them in two data tables Supplementary Table S5 (contains

Wnt signaling associated proteins) and Fig 5 (autophagy associated proteins). The expression profile of these two protein sets (Supplementary Table S5 and S6) was illustrated by heat maps, using 'pheatmap' function. For visualization of the Wnt signaling associated proteins' expression, we set the 'scale' arguments at 'row', then we cut the heat map into three pieces each of which reveals the contribution of one cluster (Fig. 4A). And for the autophagy associated proteins in Fig. 5, in 'pheatmap' function, we set the 'scale' and 'clustering_distance_rows' arguments at 'row' and 'correlation', respectively. GO enrichment analysis of the Wnt signaling associated proteins were performed by Enrichr and an adjusted p-value cut-off of below 0.05 was used to filter the results (Fig. 4B) [41,42]. We have also performed Gene Set Enrichment Analysis (GSEA) using GSEA 4.0.3 [187] with weighted enrichment statistics. The number of permutations was set to 1000. We used gene_set as the permutation type and Signal2Noise metric for ranking genes. The minimum and maximum sizes of the sets were adjusted to 15 and 500, respectively. Wnt signaling components for GSEA analysis were obtained from Molecular Signatures Database (MSigDB) [188] (Supplementary Fig. S3). In order to find specific proteins of each differentiation stage, that were differentially expressed in only one time point in comparison to all other time points (Supplementary Table S3), we conducted a differentially expression analysis by applying R/Bioconductor package 'limma' version 3.34.9 [189]. Moreover, due to the time-series nature of our study, we analysed the differentially expression of each protein (Supplementary Table S3) between two sequential time points by applying R/Bioconductor package 'limma' [189]; using 'lmFit', 'makeContrasts', 'contrasts.fit', 'ebayes' and 'toptable' functions from the package 'limma' in both differentially expression analysis procedures [189]. Proteins having absolute log2 fold-change higher than 1 and Benjamini-Hochberg adjusted p-value less than 0.05 were considered as significant differentially expressed proteins (DEPs) (Supplementary Table S7 and S8). The DEP analyses were illustrated by volcano plots (Fig. 6A–F and 7A–E). The stage-specific proteins where also demonstrated in a heat map (Supplementary Fig. S5) using 'pheatmap' function with the 'scale' argument set at 'row', and 'cluster_cols, and 'cluster_rows' arguments set at 'FALSE'. Finally, for the illustration of the STSPs in Fig. 7F, we used a built-in doughnut chart type in Excel, however, the circular visualization of the protein members of each slice was performed by setting 'circos.par',

‘circos.initialize’, ‘circos.track’ and ‘circos.trackText’ functions of the package ‘circlize’ version 0.4.8 [190].

Availability of Supporting Data and Materials

The mass spectrometry proteomics raw data have been deposited in the ProteomeXchange Consortium through the PRIDE partner repository [11] with the dataset identifier PXD017649. Other data further supporting this work are openly available in the GigaScience respository, GigaDB [192].

Additional Files

Supplemental Information includes six figures and 12 tables, and accompanies this article online in the GigaScience website.

Fig. S1: OL lineage cell generation. (A) Schematic representative of OL differentiation protocol (for materials information please refer to Methods and Supplementary Table S9). First NANOG$^+$ hESCs (B) were induced to NSCs by dual SMAD inhibition. Then, neural progenitor patterning and OPC commitment were achieved by the application of two morphogens, RA and SAG. Subsequently, PDGF medium was used to promote OPC formation. From day 80 onward, Glial medium was utilized for OL derivation. SOX1$^+$ NSCs were detected on day 8 (C) and OLIG2$^+$ NPCs appeared on day 12 (D) and participated in aggregate formation after being detached. Consequently, NKX2.2$^+$ pre-OPCs (E), day 20, differentiated into PDGFRA$^+$ OPCs on day 80 (F); their further differentiation resulted in MBP$^+$ OLs (G) on day 120. The attained OLs demonstrate a typical OL morphology that consisted of a round, central soma with multiple branching processes that expanded symmetrically outward and gave the OL a spider-in-a-web-like appearance [191]. hESC: Human embryonic stem cell; RH6: Royan H6 cell line; NSC: Neural stem cell; NPC: Neural progenitor cell; OPC: Oligodendrocyte progenitor cell; OL: Oligodendrocyte; Scale bars: 50 μm

Fig. S2: Overall validation of the collected proteome data. (A) Proportional Venn diagram compares the depth of protein coverage in three replicates. A total of 3,527 proteins were

identified in the three TMT mixtures that were analysed; while 1,056 proteins were only identified in two replicates. (B) The doughnut chart represents the diversity of the quantified proteins based on PANTHER protein class annotation.

Fig. S3: Enrichment plot of Wnt signaling components (from GSEA data set) between the last three time points (d20, d80 and d120) compared to the others using MSigDB set for Wnt signaling components. (FDR q-value 0.029).

Fig. S4: The contribution of macroautophagy in the OL lineage generation. (A) Cluster enrichment analysis (see Supplementary Table S6) featured the prominent participation of macroautophagy and autophagy pathways in OL lineage differentiation of hESCs. "in C2 and C3" reveal the clusters that these GO terms were enriched in. (B) Schematic illustration of the process and main regulatory machinery of macroautophagy. AMP-activated kinase (AMPK) signaling is depicted as the activator of the macroautophagy process (initiation) that targets the ULK1 (Unc-51-like kinase 1) initiation complex. The initiation complex then triggers membrane nucleation and phagophore formation. Hence, the cup-shaped double membrane phagophore begins to engulf the autophagic cargo and expands into the double-membrane vesicle (autophagosome) that sequesters the cargos completely (phagophore expansion). Subsequently, the autophagosome fuses with acidic lysosomes (fusion with the lysosome) and forms autolysosomes, where the cargo will be degraded (degradation). The colored ovals encompass the proteins quantified in our study of OL lineage differentiation. This figure is adapted from "Autophagy as a promoter of longevity: insights from model organisms," by M. Hansen, D. C. Rubinsztein, and D. W. Walker, 2018, Nat. Rev. Mol. Cell Biol., vol. 19, no. 9, pp. 579–593.

Fig. S5: Illustration of the abundances of differentially expressed proteins at each stage of oligodendrocyte lineage differentiation. Heat map shows the standardized relative protein expression changes of differentially expressed proteins (DEPs) at each step of oligodendrocyte (OL) lineage differentiation. The seven expression profile clusters (left colour-coded bar) describe stage-specific patterns of the dynamics of 378 DEPs. The

clusters are indicated by different colors, each of which demonstrates one specific differentiation step.

Fig. S6: Supporting figure for Supplementary Fig. S2. (A) Cells at the NSCs stage show an almost uniform expression of CDH2. (B-C) Immunofluorescent staining of the control NSC line, RSCB0181, by CDH2 and SOX1 antibodies. (D-L) About 100% of the generated cells at OPC stage (d80) expressed PDGFRA, and 97% of them were also $SOX10^{+}$. (M) Immunofluorescent staining of the $OLIG2^{+}$ NPCs at the d12 stage with an antibody from a different provider shows the same result. (N-O) 90% of the cells at pre-OPC-stage express NKX2.2. we counted five different fields of these two figures as well as Supplementary Fig. S1E. (P-W) 22% of the cells at the OL stage were mature oligodendrocyte. (X-Z) Phase contrast photos of the cells at the OL stage.

Table S1: The left table shows the abbreviations used in this article and their full forms, and the right table contains the neXtProt entry and the full name of the proteins mentioned in the text of this article.

Table S2: Protein identification, TMT reporter ion ratios, protein quantitation, and study design. '171102', '171108' and '171105' tables represent the data acquired by the first, second and third TMT experiments. 'Aggregated Data' table includes the whole identified proteins of the three replicates in one table. The 'Study Design' table shows the TMT experimental design and the 'Replicates Correlation' table illustrates the Pearson correlation coefficients of the replicates. The analysis of variance (ANOVA, in the 'Aggregated Data' table) represents 3132 proteins (about 81% of the whole identified proteins) that showed significant changes (adjusted p-value ≤ 0.05) through the OL lineage differentiation of hESCs. Based on the Pearson correlation coefficient analysis, 'd0_r1' was left out of the study. In addition, we decided to present the most correlated triplicates for the further analysis.

Table S3: The first table (Quantified Proteins) shows the relative expression of the whole identified and quantified proteins in every biological replicate of each time point during OL lineage differentiation. The second table (Variable Loadings Matrix) includes the details

of the PCA of the proteome profile of each differentiation stage in Fig. 2B. The third table (Contribution of PCs) reveals the contribution of computed PCs, among which we chose PC1 and PC2 for the illustration of the PCA analysis in Fig. 2B. The fourth table (Averages of the Replicates) contains the total of 3,855 quantified proteins along with the average of their relative expression with respect to each differentiation stage. The following analyses were conducted on 'Averages of the Replicates' table.

Table S4: Cluster 1, 2 and 3 tables present each cluster's members, their relative expression, and their membership score. Highlighted BPs table involves the highlighted biological processes of each protein cluster.

Table S5: Wnt signaling related GOs of the demonstrated Wnt signaling associated proteins in Fig. 4A. These proteins relative expression changes, and the resultant GOs of their enrichment analysis are presented in Fig. 4A_GO, Fig. 4A_expression and Fig. 4B sheets respectively.

Table S6: This table demonstrates the relative expression of the autophagy associated proteins, which has been illustrated by heatmap in Fig. 5.

Table S7: The first six sheets (named Fig. 6A-F) provide the tabular illustration of the stage-specific proteins of the oligodendrocyte lineage differentiation. The table in ‘Fig. S5’ sheet demonstrates the relative expression changes of the stage-specific proteins. Each table is a supporting data for the figure with the same name. For the identification of the stage-specific proteins, the expression of each protein at a specific time point was compared by its expression in all other time points using the R/Bioconductor package ‘limma’.

Table S8: The first five sheets (named Fig.7A-E) provide the tabular illustration of the stage transition specific proteins (STSPs) in the oligodendrocyte lineage differentiation. Each sheet is supporting data for the figure with the same name. In this analysis, the expression of each protein at a specific time point was compared by its expression at the next time point using the R/Bioconductor package ‘limma’. The demonstrated table in the

sixth sheet (Fig.7F) involves the relative expression changes of all STSPs, the proteins that have been shown in Fig. 7F.

Table S9: Detailed compositions of the culture media used in the experiments.

Table S10: List of antibodies used in the experiments.

Table S11: Details of the reagents used for protein isolation.

Table S12: Oligodendrocyte lineage proteome data covered a significant number of enriched proteins that included: 1,180 enzymes and enzyme modulators, 698 nucleic acid binding and transcription factors (TFs), 425 intra/extracellular trafficking and signaling proteins, 203 cytoskeletal and extracellular matrix (ECM) proteins, and 57 structural and adhesive proteins.

Abbreviations

Please find the list of abbreviations in Supplementary Table S1.

Competing interests

The authors declare that they have no conflict of interests.

Funding

This work was supported by a grant from Royan Institute. Aspects of this research, including the efforts of M. Mirzaei, have been enabled by access to the Australian Proteome Analysis Facility.

Author contributions

G. H. S., P. P., and M. J. conceived the project, designed the study and interpreted results with the efforts of R. K. and M. M. and H. B.; P. P. performed the experiments, except for the TMT experiment which was designed, performed and, in part, analysed by M. M., Y. W., and A. A., and V. G.; A. M. contributed in RH6 preparation; P. P. and R. K. performed computational analyses and prepared figures and tables; P. P. wrote the manuscript with

input from the co-authors; G. H. S. and M. M., and H. B. oversaw all aspects of the study. All authors proofread the paper and approved the final version.

Acknowledgements

We would like to thank the RSCB team for sharing the RH6 (hESC) line. We thank H. Ansari for his assistance in RH6 expansion and express our appreciation to R. Moghimi, F. S. Samani, F. Yekani, S. Nemati, S. Mirshahvaladi, P. Parsamatin, M. Alikhani, A. Samadian, M. Azimi, M. Dorraj, and N. Pirhadi for laboratory management and support. We wish to acknowledge S. M. Hosseini for providing antibodies, E. Shahbazi for his help in immunostaining of the OLIG2$^+$ and MBP$^+$ cells, F. Moeinvaziri for her help in immunostaining of the NKX2.2$^+$ cells, M. Saberian for unsupervised data imputation, and A. Sharifi-Zarchi for technical supports. We also would like to thank S. Simorgh, A. M. Khanghahi, and F. Shekari for their discussions. The authors would like to thank Iran's National Elites Foundation (INEF) for its support.

References

1. Baumann N, Pham-Dinh D. Biology of oligodendrocyte and myelin in the mammalian central nervous system. Physiol Rev [Internet]. 2001;81:871–927. Available from: http://www.ncbi.nlm.nih.gov/pubmed/11274346

2. Franklin RJM, Goldman SA. Glia Disease and Repair-Remyelination. Cold Spring Harb Perspect Biol [Internet]. 2015;7:a020594. Available from: http://www.ncbi.nlm.nih.gov/pubmed/25986556

3. Abu-Rub M, Miller RH. Emerging Cellular and Molecular Strategies for Enhancing Central Nervous System (CNS) Remyelination. Brain Sci [Internet]. 2018;8. Available from: http://www.ncbi.nlm.nih.gov/pubmed/29914096

4. Zhu Z, Huangfu D. Human pluripotent stem cells: an emerging model in developmental biology. Development [Internet]. 2013;140:705–17. Available from: http://dev.biologists.org/cgi/doi/10.1242/dev.086165

5. Chen X, He Y, Lu F. Autophagy in Stem Cell Biology: A Perspective on Stem Cell

Self-Renewal and Differentiation. Stem Cells Int [Internet]. Hindawi; 2018;2018:9131397. Available from: http://www.ncbi.nlm.nih.gov/pubmed/29765428

6. Douvaras P, Wang J, Zimmer M, Hanchuk S, O'Bara MA, Sadiq S, et al. Efficient generation of myelinating oligodendrocytes from primary progressive multiple sclerosis patients by induced pluripotent stem cells. Stem cell reports [Internet]. The Authors; 2014;3:250–9. Available from: http://dx.doi.org/10.1016/j.stemcr.2014.06.012

7. Douvaras P, Fossati V. Generation and isolation of oligodendrocyte progenitor cells from human pluripotent stem cells. Nat Protoc [Internet]. 2015;10:1143–54. Available from: http://dx.doi.org/10.1038/nprot.2015.075

8. Baharvand H, Ashtiani SK, Taee A, Massumi M, Valojerdi MR, Yazdi PE, et al. Generation of new human embryonic stem cell lines with diploid and triploid karyotypes. Dev Growth Differ. 2006;48:117–28.

9. Douvaras P, Rusielewicz T, Kim KH, Haines JD, Casaccia P, Fossati V. Epigenetic modulation of human induced pluripotent stem cell differentiation to oligodendrocytes. Int J Mol Sci. 2016;17.

10. The UniProt Consortium. UniProt: the universal protein knowledgebase. Nucleic Acids Res [Internet]. Oxford University Press; 2017;45:D158–69. Available from: http://www.ncbi.nlm.nih.gov/pubmed/27899622

11. Deutsch EW, Csordas A, Sun Z, Jarnuczak A, Perez-Riverol Y, Ternent T, et al. The ProteomeXchange consortium in 2017: supporting the cultural change in proteomics public data deposition. Nucleic Acids Res [Internet]. 2017;45:D1100–6. Available from: http://www.ncbi.nlm.nih.gov/pubmed/27924013

12. Wei R, Wang J, Su M, Jia E, Chen S, Chen T, et al. Missing Value Imputation Approach for Mass Spectrometry-based Metabolomics Data. Sci Rep [Internet]. Springer US; 2018;8:663. Available from: http://dx.doi.org/10.1038/s41598-017-19120-0

13. Mi H, Muruganujan A, Huang X, Ebert D, Mills C, Guo X, et al. Protocol Update for large-scale genome and gene function analysis with the PANTHER classification system (v.14.0). Nat Protoc [Internet]. Springer US; 2019;14:703–21. Available from: http://dx.doi.org/10.1038/s41596-019-0128-8

14. Vogel A, Upadhya R, Shetty AK. Neural stem cell derived extracellular vesicles: Attributes and prospects for treating neurodegenerative disorders. EBioMedicine [Internet]. Elsevier B.V.; 2018;38:273–82. Available from: https://doi.org/10.1016/j.ebiom.2018.11.026

15. Horgusluoglu E, Nudelman K, Nho K, Saykin AJ. Adult neurogenesis and neurodegenerative diseases: A systems biology perspective. Am J Med Genet B Neuropsychiatr Genet [Internet]. 2017;174:93–112. Available from: http://www.ncbi.nlm.nih.gov/pubmed/26879907

16. Annenkov A. Receptor tyrosine kinase (RTK) signalling in the control of neural stem and progenitor cell (NSPC) development. Mol Neurobiol [Internet]. 2014;49:440–71. Available from: http://www.ncbi.nlm.nih.gov/pubmed/23982746

17. Knobloch M. The Role of Lipid Metabolism for Neural Stem Cell Regulation. Brain Plast (Amsterdam, Netherlands) [Internet]. 2017;3:61–71. Available from: http://www.ncbi.nlm.nih.gov/pubmed/29765860

18. Kim D-Y, Rhee I, Paik J. Metabolic circuits in neural stem cells. Cell Mol Life Sci [Internet]. 2014;71:4221–41. Available from: http://www.ncbi.nlm.nih.gov/pubmed/25037158

19. Soliven B. Calcium signalling in cells of oligodendroglial lineage. Microsc Res Tech [Internet]. 2001;52:672–9. Available from: http://doi.wiley.com/10.1002/jemt.1051

20. Haak LL, Grimaldi M, Russell JT. Mitochondria in myelinating cells: calcium signaling in oligodendrocyte precursor cells. Cell Calcium [Internet]. 2000;28:297–306. Available from: http://www.ncbi.nlm.nih.gov/pubmed/11115369

21. Butt AM. Neurotransmitter-mediated calcium signalling in oligodendrocyte physiology and pathology. Glia [Internet]. 2006;54:666–75. Available from: http://www.ncbi.nlm.nih.gov/pubmed/17006895

22. Li T, Wang L, Ma T, Wang S, Niu J, Li H, et al. Dynamic Calcium Release From Endoplasmic Reticulum Mediated by Ryanodine Receptor 3 Is Crucial for Oligodendroglial Differentiation. Front Mol Neurosci [Internet]. 2018;11:162. Available from: http://www.ncbi.nlm.nih.gov/pubmed/29867353

23. Bacon C, Lakics V, Machesky L, Rumsby M. N-WASP regulates extension of filopodia and processes by oligodendrocyte progenitors, oligodendrocytes, and Schwann cells-implications for axon ensheathment at myelination. Glia [Internet]. 2007;55:844–58. Available from: http://www.ncbi.nlm.nih.gov/pubmed/17405146

24. Boullerne AI, Benjamins JA. Nitric oxide synthase expression and nitric oxide toxicity in oligodendrocytes. Antioxid Redox Signal [Internet]. 2006;8:967–80. Available from: http://www.ncbi.nlm.nih.gov/pubmed/16771686

25. Tager HS, Steiner DF. Peptide hormones. Annu Rev Biochem [Internet]. 1974;43:509–38. Available from: http://www.ncbi.nlm.nih.gov/pubmed/4368999

26. Chrast R, Saher G, Nave K-A, Verheijen MHG. Lipid metabolism in myelinating glial cells: lessons from human inherited disorders and mouse models. J Lipid Res [Internet]. 2011;52:419–34. Available from: http://www.ncbi.nlm.nih.gov/pubmed/21062955

27. Yu J, Vodyanik MA, Smuga-Otto K, Antosiewicz-Bourget J, Frane JL, Tian S, et al. Induced pluripotent stem cell lines derived from human somatic cells. Science [Internet]. 2007;318:1917–20. Available from: http://www.ncbi.nlm.nih.gov/pubmed/18029452

28. Cimadamore F, Amador-Arjona A, Chen C, Huang C-T, Terskikh A V. SOX2-LIN28/let-7 pathway regulates proliferation and neurogenesis in neural precursors. Proc Natl Acad Sci U S A [Internet]. 2013;110:E3017-26. Available from: http://www.ncbi.nlm.nih.gov/pubmed/23884650

29. Goldman SA, Kuypers NJ. How to make an oligodendrocyte. Development [Internet]. 2015;142:3983–95. Available from: http://www.ncbi.nlm.nih.gov/pubmed/26628089

30. Almeida RG. The Rules of Attraction in Central Nervous System Myelination. Front Cell Neurosci [Internet]. 2018;12:367. Available from: http://www.ncbi.nlm.nih.gov/pubmed/30374292

31. Schnädelbach O, Blaschuk OW, Symonds M, Gour BJ, Doherty P, Fawcett JW. N-cadherin influences migration of oligodendrocytes on astrocyte monolayers. Mol Cell Neurosci [Internet]. 2000;15:288–302. Available from: http://www.ncbi.nlm.nih.gov/pubmed/10736205

32. Ji S, Doucette JR, Nazarali AJ. Sirt2 is a novel in vivo downstream target of Nkx2.2 and enhances oligodendroglial cell differentiation. J Mol Cell Biol [Internet]. 2011;3:351–9. Available from: http://www.ncbi.nlm.nih.gov/pubmed/21669943

33. Lamprianou S, Chatzopoulou E, Thomas J-L, Bouyain S, Harroch S. A complex between contactin-1 and the protein tyrosine phosphatase PTPRZ controls the development of oligodendrocyte precursor cells. Proc Natl Acad Sci U S A [Internet]. 2011;108:17498–503. Available from: http://www.ncbi.nlm.nih.gov/pubmed/21969550

34. Shao Z, Lee X, Huang G, Sheng G, Henderson CE, Louvard D, et al. LINGO-1 Regulates Oligodendrocyte Differentiation through the Cytoplasmic Gelsolin Signaling Pathway. J Neurosci. 2017;37:3127–37.

35. Jahn O, Tenzer S, Werner HB. Myelin proteomics: molecular anatomy of an insulating sheath. Mol Neurobiol [Internet]. 2009;40:55–72. Available from: http://www.ncbi.nlm.nih.gov/pubmed/19452287

36. Barresi MJF, Gilbert SF. Developmental Biology, Twelfth Edition [Internet]. Twelfth Ed. New York: Oxford University Press; 2019. Available from: http://www.isbnsearch.org/isbn/9781605358246

37. Veeman MT, Axelrod JD, Moon RT. A second canon. Functions and mechanisms of beta-catenin-independent Wnt signaling. Dev Cell [Internet]. 2003;5:367–77. Available from: https://linkinghub.elsevier.com/retrieve/pii/S1534580703002661

38. Marinou K, Christodoulides C, Antoniades C, Koutsilieris M. Wnt signaling in cardiovascular physiology. Trends Endocrinol Metab [Internet]. Elsevier Ltd; 2012;23:628–36. Available from: http://dx.doi.org/10.1016/j.tem.2012.06.001

39. Huang DW, Sherman BT, Lempicki RA. Systematic and integrative analysis of large gene lists using DAVID bioinformatics resources. Nat Protoc [Internet]. 2009;4:44–57. Available from: http://www.ncbi.nlm.nih.gov/pubmed/19131956

40. UniProt Consortium. UniProt: a worldwide hub of protein knowledge. Nucleic Acids Res [Internet]. Oxford University Press; 2019;47:D506–15. Available from: http://www.ncbi.nlm.nih.gov/pubmed/30395287

41. Kuleshov M V., Jones MR, Rouillard AD, Fernandez NF, Duan Q, Wang Z, et al.

Enrichr: a comprehensive gene set enrichment analysis web server 2016 update. Nucleic Acids Res [Internet]. 2016;44:W90-7. Available from: http://www.ncbi.nlm.nih.gov/pubmed/27141961

42. Chen EY, Tan CM, Kou Y, Duan Q, Wang Z, Meirelles GV, et al. Enrichr: interactive and collaborative HTML5 gene list enrichment analysis tool. BMC Bioinformatics [Internet]. 2013;14:128. Available from: http://www.ncbi.nlm.nih.gov/pubmed/23586463

43. Merrill BJ. Wnt pathway regulation of embryonic stem cell self-renewal. Cold Spring Harb Perspect Biol [Internet]. 2012;4:a007971. Available from: http://cshperspectives.cshlp.org/lookup/doi/10.1101/cshperspect.a007971

44. Fancy SPJ, Baranzini SE, Zhao C, Yuk D-I, Irvine K-A, Kaing S, et al. Dysregulation of the Wnt pathway inhibits timely myelination and remyelination in the mammalian CNS. Genes Dev [Internet]. 2009;23:1571–85. Available from: http://www.ncbi.nlm.nih.gov/pubmed/19515974

45. Dai Z-M, Sun S, Wang C, Huang H, Hu X, Zhang Z, et al. Stage-specific regulation of oligodendrocyte development by Wnt/β-catenin signaling. J Neurosci [Internet]. 2014;34:8467–73. Available from: http://www.ncbi.nlm.nih.gov/pubmed/24948802

46. Sato N, Meijer L, Skaltsounis L, Greengard P, Brivanlou AH. Maintenance of pluripotency in human and mouse embryonic stem cells through activation of Wnt signaling by a pharmacological GSK-3-specific inhibitor. Nat Med [Internet]. 2004;10:55–63. Available from: http://www.ncbi.nlm.nih.gov/pubmed/14702635

47. Singla DK, Schneider DJ, LeWinter MM, Sobel BE. wnt3a but not wnt11 supports self-renewal of embryonic stem cells. Biochem Biophys Res Commun [Internet]. 2006;345:789–95. Available from: http://www.ncbi.nlm.nih.gov/pubmed/16707109

48. Sokol SY. Maintaining embryonic stem cell pluripotency with Wnt signaling. Development [Internet]. 2011;138:4341–50. Available from: http://www.ncbi.nlm.nih.gov/pubmed/21903672

49. Guo F, Lang J, Sohn J, Hammond E, Chang M, Pleasure D. Canonical Wnt signaling in the oligodendroglial lineage--puzzles remain. Glia [Internet]. 2015;63:1671–93. Available from: http://www.ncbi.nlm.nih.gov/pubmed/25782433

50. Soomro SH, Jie J, Fu H. Oligodendrocytes Development and Wnt Signaling Pathway. Mustafa Hegazy AA, editor. Int J Hum Anat [Internet]. 2018;1:17–35. Available from: https://openaccesspub.org/ijha/article/883

51. Rodrigo Albors A, Tazaki A, Rost F, Nowoshilow S, Chara O, Tanaka EM. Planar cell polarity-mediated induction of neural stem cell expansion during axolotl spinal cord regeneration. Elife [Internet]. 2015;4:e10230. Available from: http://www.ncbi.nlm.nih.gov/pubmed/26568310

52. Butler MT, Wallingford JB. Planar cell polarity in development and disease. Nat Rev Mol Cell Biol [Internet]. Nature Publishing Group; 2017;18:375–88. Available from: http://dx.doi.org/10.1038/nrm.2017.11

53. Chavali M, Klingener M, Kokkosis AG, Garkun Y, Felong S, Maffei A, et al. Non-canonical Wnt signaling regulates neural stem cell quiescence during homeostasis and after demyelination. Nat Commun [Internet]. Springer US; 2018;9:36. Available from: http://dx.doi.org/10.1038/s41467-017-02440-0

54. Jarjour AA, Boyd A, Dow LE, Holloway RK, Goebbels S, Humbert PO, et al. The polarity protein Scribble regulates myelination and remyelination in the central nervous system. PLoS Biol [Internet]. 2015;13:e1002107. Available from: http://www.ncbi.nlm.nih.gov/pubmed/25807062

55. Jarjour AA, Velichkova AN, Boyd A, Lord KM, Torsney C, Henderson DJ, et al. The formation of paranodal spirals at the ends of CNS myelin sheaths requires the planar polarity protein Vangl2. Glia [Internet]. 2020;glia.23809. Available from: https://onlinelibrary.wiley.com/doi/abs/10.1002/glia.23809

56. Hayes MN, McCarthy K, Jin A, Oliveira ML, Iyer S, Garcia SP, et al. Vangl2/RhoA Signaling Pathway Regulates Stem Cell Self-Renewal Programs and Growth in Rhabdomyosarcoma. Cell Stem Cell [Internet]. Elsevier Inc.; 2018;22:414-427.e6. Available from: https://doi.org/10.1016/j.stem.2018.02.002

57. Mizushima N, Levine B. Autophagy in mammalian development and differentiation. Nat Cell Biol [Internet]. Nature Publishing Group; 2010;12:823–30. Available from: http://dx.doi.org/10.1038/ncb0910-823

58. Yue Z, Jin S, Yang C, Levine AJ, Heintz N. Beclin 1, an autophagy gene essential for early embryonic development, is a haploinsufficient tumor suppressor. Proc Natl Acad Sci U S A [Internet]. 2003;100:15077–82. Available from: http://www.ncbi.nlm.nih.gov/pubmed/14657337

59. Levine B, Kroemer G. Autophagy in the pathogenesis of disease. Cell [Internet]. 2008;132:27–42. Available from: https://linkinghub.elsevier.com/retrieve/pii/S0092867407016856

60. Huang DW, Sherman BT, Lempicki RA. Bioinformatics enrichment tools: paths toward the comprehensive functional analysis of large gene lists. Nucleic Acids Res [Internet]. 2009;37:1–13. Available from: http://www.ncbi.nlm.nih.gov/pubmed/19033363

61. Hansen M, Rubinsztein DC, Walker DW. Autophagy as a promoter of longevity: insights from model organisms. Nat Rev Mol Cell Biol [Internet]. Springer US; 2018;19:579–93. Available from: http://dx.doi.org/10.1038/s41580-018-0033-y

62. Behrends C, Sowa ME, Gygi SP, Harper JW. Network organization of the human autophagy system. Nature [Internet]. Nature Publishing Group; 2010;466:68–76. Available from: http://dx.doi.org/10.1038/nature09204

63. Smith CM, Mayer JA, Duncan ID. Autophagy promotes oligodendrocyte survival and function following dysmyelination in a long-lived myelin mutant. J Neurosci [Internet]. 2013;33:8088–100. Available from: http://www.ncbi.nlm.nih.gov/pubmed/23637198

64. Bankston AN, Forston MD, Howard RM, Andres KR, Smith AE, Ohri SS, et al. Autophagy is essential for oligodendrocyte differentiation, survival, and proper myelination. Glia [Internet]. 2019;67:1745–59. Available from: http://www.ncbi.nlm.nih.gov/pubmed/31162728

65. Vaughn CB, Jakimovski D, Kavak KS, Ramanathan M, Benedict RHB, Zivadinov R, et al. Epidemiology and treatment of multiple sclerosis in elderly populations. Nat Rev Neurol [Internet]. Springer US; 2019;15:329–42. Available from: http://dx.doi.org/10.1038/s41582-019-0183-3

66. Alirezaei M, Fox HS, Flynn CT, Moore CS, Hebb ALO, Frausto RF, et al. Elevated ATG5 expression in autoimmune demyelination and multiple sclerosis. Autophagy

[Internet]. 2009;5:152–8. Available from: http://www.ncbi.nlm.nih.gov/pubmed/19066443

67. Igci M, Baysan M, Yigiter R, Ulasli M, Geyik S, Bayraktar R, et al. Gene expression profiles of autophagy-related genes in multiple sclerosis. Gene [Internet]. 2016;588:38–46. Available from: http://www.ncbi.nlm.nih.gov/pubmed/27125224

68. Rangaraju S, Verrier JD, Madorsky I, Nicks J, Dunn WA, Notterpek L. Rapamycin activates autophagy and improves myelination in explant cultures from neuropathic mice. J Neurosci [Internet]. 2010;30:11388–97. Available from: http://www.ncbi.nlm.nih.gov/pubmed/20739560

69. Meikle L, Pollizzi K, Egnor A, Kramvis I, Lane H, Sahin M, et al. Response of a neuronal model of tuberous sclerosis to mammalian target of rapamycin (mTOR) inhibitors: effects on mTORC1 and Akt signaling lead to improved survival and function. J Neurosci [Internet]. 2008;28:5422–32. Available from: http://www.ncbi.nlm.nih.gov/pubmed/18495876

70. Liang P, Le W. Role of autophagy in the pathogenesis of multiple sclerosis. Neurosci Bull [Internet]. 2015;31:435–44. Available from: http://www.ncbi.nlm.nih.gov/pubmed/26254059

71. Belgrad J, De Pace R, Fields RD. Autophagy in Myelinating Glia. J Neurosci [Internet]. 2020;40:256–66. Available from: http://www.jneurosci.org/lookup/doi/10.1523/JNEUROSCI.1066-19.2019

72. Djajadikerta A, Keshri S, Pavel M, Prestil R, Ryan L, Rubinsztein DC. Autophagy Induction as a Therapeutic Strategy for Neurodegenerative Diseases. J Mol Biol [Internet]. Elsevier Ltd; 2020;432:2799–821. Available from: https://doi.org/10.1016/j.jmb.2019.12.035

73. Di Rienzo M, Antonioli M, Fusco C, Liu Y, Mari M, Orhon I, et al. Autophagy induction in atrophic muscle cells requires ULK1 activation by TRIM32 through unanchored K63-linked polyubiquitin chains. Sci Adv [Internet]. 2019;5:eaau8857. Available from: http://www.ncbi.nlm.nih.gov/pubmed/31123703

74. Richards M, Tan S-P, Tan J-H, Chan W-K, Bongso A. The transcriptome profile of human embryonic stem cells as defined by SAGE. Stem Cells [Internet]. 2004;22:51–

64. Available from: http://www.ncbi.nlm.nih.gov/pubmed/14688391

75. Yang M, Yang S-L, Herrlinger S, Liang C, Dzieciatkowska M, Hansen KC, et al. Lin28 promotes the proliferative capacity of neural progenitor cells in brain development. Development [Internet]. 2015;142:1616–27. Available from: http://www.ncbi.nlm.nih.gov/pubmed/25922525

76. Tsialikas J, Romer-Seibert J. LIN28: roles and regulation in development and beyond. Development [Internet]. 2015;142:2397–404. Available from: http://www.ncbi.nlm.nih.gov/pubmed/26199409

77. Kaneko Y, Sakakibara S, Imai T, Suzuki A, Nakamura Y, Sawamoto K, et al. Musashi1: an evolutionally conserved marker for CNS progenitor cells including neural stem cells. Dev Neurosci [Internet]. 2000;22:139–53. Available from: http://www.ncbi.nlm.nih.gov/pubmed/10657706

78. Sugiyama-Nakagiri Y, Akiyama M, Shibata S, Okano H, Shimizu H. Expression of RNA-binding protein Musashi in hair follicle development and hair cycle progression. Am J Pathol [Internet]. American Society for Investigative Pathology; 2006;168:80–92. Available from: http://dx.doi.org/10.2353/ajpath.2006.050469

79. Clarke RB, Spence K, Anderson E, Howell A, Okano H, Potten CS. A putative human breast stem cell population is enriched for steroid receptor-positive cells. Dev Biol [Internet]. 2005;277:443–56. Available from: http://www.ncbi.nlm.nih.gov/pubmed/15617686

80. Rezza A, Skah S, Roche C, Nadjar J, Samarut J, Plateroti M. The overexpression of the putative gut stem cell marker Musashi-1 induces tumorigenesis through Wnt and Notch activation. J Cell Sci [Internet]. 2010;123:3256–65. Available from: http://www.ncbi.nlm.nih.gov/pubmed/20826465

81. Kudinov AE, Karanicolas J, Golemis EA, Boumber Y. Musashi RNA-Binding Proteins as Cancer Drivers and Novel Therapeutic Targets. Clin Cancer Res [Internet]. 2017;23:2143–53. Available from: http://www.ncbi.nlm.nih.gov/pubmed/28143872

82. Slamecka J, McClellan S, Wilk A, Laurini J, Manci E, Hoerstrup SP, et al. Induced pluripotent stem cells derived from human amnion in chemically defined conditions. Cell

Cycle [Internet]. 2018;17:330–47. Available from: http://www.ncbi.nlm.nih.gov/pubmed/29143560

83. Loboda A, Damulewicz M, Pyza E, Jozkowicz A, Dulak J. Role of Nrf2/HO-1 system in development, oxidative stress response and diseases: an evolutionarily conserved mechanism. Cell Mol Life Sci [Internet]. Springer International Publishing; 2016;73:3221–47. Available from: http://www.ncbi.nlm.nih.gov/pubmed/27100828

84. Lomelino CL, Andring JT, McKenna R, Kilberg MS. Asparagine synthetase: Function, structure, and role in disease. J Biol Chem [Internet]. 2017;292:19952–8. Available from: http://www.ncbi.nlm.nih.gov/pubmed/29084849

85. Lin C-Y, Peng C-Y, Huang T-T, Wu M-L, Lai Y-L, Peng DH, et al. Exacerbation of oxidative stress-induced cell death and differentiation in induced pluripotent stem cells lacking heme oxygenase-1. Stem Cells Dev [Internet]. 2012;21:1675–87. Available from: http://www.ncbi.nlm.nih.gov/pubmed/22034921

86. Stepniewski J, Kachamakova-Trojanowska N, Ogrocki D, Szopa M, Matlok M, Beilharz M, et al. Induced pluripotent stem cells as a model for diabetes investigation. Sci Rep [Internet]. 2015;5:8597. Available from: http://www.ncbi.nlm.nih.gov/pubmed/25716801

87. Stepniewski J, Pacholczak T, Skrzypczyk A, Ciesla M, Szade A, Szade K, et al. Heme oxygenase-1 affects generation and spontaneous cardiac differentiation of induced pluripotent stem cells. IUBMB Life [Internet]. 2018;70:129–42. Available from: http://www.ncbi.nlm.nih.gov/pubmed/29316264

88. Phanstiel DH, Brumbaugh J, Wenger CD, Tian S, Probasco MD, Bailey DJ, et al. Proteomic and phosphoproteomic comparison of human ES and iPS cells. Nat Methods [Internet]. 2011;8:821–7. Available from: http://www.ncbi.nlm.nih.gov/pubmed/21983960

89. Kempf H, Olmer R, Kropp C, Rückert M, Jara-Avaca M, Robles-Diaz D, et al. Controlling expansion and cardiomyogenic differentiation of human pluripotent stem cells in scalable suspension culture. Stem cell reports [Internet]. 2014;3:1132–46. Available from: http://www.ncbi.nlm.nih.gov/pubmed/25454631

90. Choi J, Lee S, Mallard W, Clement K, Tagliazucchi GM, Lim H, et al. A comparison

of genetically matched cell lines reveals the equivalence of human iPSCs and ESCs. Nat Biotechnol [Internet]. Nature Publishing Group; 2015;33:1173–81. Available from: http://dx.doi.org/10.1038/nbt.3388

91. Krall AS, Xu S, Graeber TG, Braas D, Christofk HR. Asparagine promotes cancer cell proliferation through use as an amino acid exchange factor. Nat Commun [Internet]. Nature Publishing Group; 2016;7:11457. Available from: http://dx.doi.org/10.1038/ncomms11457

92. Vermeulen T, Görg B, Vogl T, Wolf M, Varga G, Toutain A, et al. Glutamine synthetase is essential for proliferation of fetal skin fibroblasts. Arch Biochem Biophys [Internet]. 2008;478:96–102. Available from: http://www.ncbi.nlm.nih.gov/pubmed/18662667

93. Carey BW, Finley LWS, Cross JR, Allis CD, Thompson CB. Intracellular α-ketoglutarate maintains the pluripotency of embryonic stem cells. Nature [Internet]. Nature Publishing Group; 2015;518:413–6. Available from: http://dx.doi.org/10.1038/nature13981

94. Shparberg RA, Glover HJ, Morris MB. Modeling Mammalian Commitment to the Neural Lineage Using Embryos and Embryonic Stem Cells. Front Physiol [Internet]. 2019;10:705. Available from: http://www.ncbi.nlm.nih.gov/pubmed/31354503

95. Ryu JM, Lee SH, Seong JK, Han HJ. Glutamine contributes to maintenance of mouse embryonic stem cell self-renewal through PKC-dependent downregulation of HDAC1 and DNMT1/3a. Cell Cycle [Internet]. 2015;14:3292–305. Available from: http://www.ncbi.nlm.nih.gov/pubmed/26375799

96. Xiao D, Liu X, Zhang M, Zou M, Deng Q, Sun D, et al. Direct reprogramming of fibroblasts into neural stem cells by single non-neural progenitor transcription factor Ptf1a. Nat Commun [Internet]. Springer US; 2018;9:2865. Available from: http://dx.doi.org/10.1038/s41467-018-05209-1

97. Atlasi Y, Stunnenberg HG. The interplay of epigenetic marks during stem cell differentiation and development. Nat Rev Genet [Internet]. Nature Publishing Group; 2017;18:643–58. Available from: http://dx.doi.org/10.1038/nrg.2017.57

98. Chan SF, Huang X, McKercher SR, Zaidi R, Okamoto S-I, Nakanishi N, et al. Transcriptional profiling of MEF2-regulated genes in human neural progenitor cells derived from embryonic stem cells. Genomics data [Internet]. The Authors; 2015;3:24–7. Available from: http://dx.doi.org/10.1016/j.gdata.2014.10.022

99. Lee MW, Chang AC, Sun DS, Hsu CY, Chang NC. Restricted expression of LUZP in neural lineage cells: a study in embryonic stem cells. J Biomed Sci [Internet]. 2001;8:504–11. Available from: http://www.ncbi.nlm.nih.gov/pubmed/11702014

100. Sima N, Li R, Huang W, Xu M, Beers J, Zou J, et al. Neural stem cells for disease modeling and evaluation of therapeutics for infantile (CLN1/PPT1) and late infantile (CLN2/TPP1) neuronal ceroid lipofuscinoses. Orphanet J Rare Dis [Internet]. Orphanet Journal of Rare Diseases; 2018;13:54. Available from: https://ojrd.biomedcentral.com/articles/10.1186/s13023-018-0798-2

101. Liu B, Li Y, Stackpole EE, Novak A, Gao Y, Zhao Y, et al. Regulatory discrimination of mRNAs by FMRP controls mouse adult neural stem cell differentiation. Proc Natl Acad Sci U S A [Internet]. 2018;115:E11397–405. Available from: http://www.ncbi.nlm.nih.gov/pubmed/30373821

102. Wang J, Cheng H, Li X, Lu W, Wang K, Wen T. Regulation of neural stem cell differentiation by transcription factors HNF4-1 and MAZ-1. Mol Neurobiol [Internet]. 2013;47:228–40. Available from: http://www.ncbi.nlm.nih.gov/pubmed/22944911

103. Liu B, Ma A, Zhang F, Wang Y, Li Z, Li Q, et al. MAZ mediates the cross-talk between CT-1 and NOTCH1 signaling during gliogenesis. Sci Rep [Internet]. Nature Publishing Group; 2016;6:21534. Available from: http://dx.doi.org/10.1038/srep21534

104. Najm FJ, Lager AM, Zaremba A, Wyatt K, Caprariello A V., Factor DC, et al. Transcription factor-mediated reprogramming of fibroblasts to expandable, myelinogenic oligodendrocyte progenitor cells. Nat Biotechnol [Internet]. Nature Publishing Group; 2013;31:426–33. Available from: http://dx.doi.org/10.1038/nbt.2561

105. Thakurela S, Tiwari N, Schick S, Garding A, Ivanek R, Berninger B, et al. Mapping gene regulatory circuitry of Pax6 during neurogenesis. Cell Discov [Internet]. Nature Publishing Group; 2016;2:15045. Available from:

http://dx.doi.org/10.1038/celldisc.2015.45

106. Andersen J. Study of Ascl1 function in the neurogenic lineage of the adult mouse hippocampus [Internet]. Dr. thesis, UCL (University Coll. London). University College London; 2015. Available from: http://discovery.ucl.ac.uk/1469969/

107. Gaudet P, Michel P-A, Zahn-Zabal M, Britan A, Cusin I, Domagalski M, et al. The neXtProt knowledgebase on human proteins: 2017 update. Nucleic Acids Res [Internet]. 2017;45:D177–82. Available from: http://www.ncbi.nlm.nih.gov/pubmed/27899619

108. Timmer JR, Mak TW, Manova K, Anderson K V., Niswander L. Tissue morphogenesis and vascular stability require the Frem2 protein, product of the mouse myelencephalic blebs gene. Proc Natl Acad Sci U S A [Internet]. 2005;102:11746–50. Available from: http://www.ncbi.nlm.nih.gov/pubmed/16087869

109. Beaumont M, Akloul L, Carré W, Quélin C, Journel H, Pasquier L, et al. Targeted panel sequencing establishes the implication of planar cell polarity pathway and involves new candidate genes in neural tube defect disorders. Hum Genet [Internet]. Springer Berlin Heidelberg; 2019;138:363–74. Available from: http://dx.doi.org/10.1007/s00439-019-01993-y

110. CORTECON: A Temporal Transcriptome Analysis of In Vitro Human Cerebral Cortex Development From Human Embryonic Stem Cells. van de Leemput et. al, Neuron (2014) [Internet]. [cited 2019 Aug 30]. Available from: cortecon.neuralsci.org/index.php?cort_mode=genedisplay&geneid=3728

111. Angst BD, Marcozzi C, Magee AI. The cadherin superfamily: diversity in form and function. J Cell Sci [Internet]. 2001;114:629–41. Available from: http://www.ncbi.nlm.nih.gov/pubmed/11171368

112. Yang L, Jiang Z, Zhou L, Zhao K, Ma X, Cheng G. Hydrophilic cell-derived extracellular matrix as a niche to promote adhesion and differentiation of neural progenitor cells. RSC Adv [Internet]. Royal Society of Chemistry; 2017;7:45587–94. Available from: http://xlink.rsc.org/?DOI=C7RA08273H

113. Jiao Q, Li X, An J, Zhang Z, Chen X, Tan J, et al. Cell-Cell Connection Enhances Proliferation and Neuronal Differentiation of Rat Embryonic Neural Stem/Progenitor

Cells. Front Cell Neurosci [Internet]. 2017;11:200. Available from: http://www.ncbi.nlm.nih.gov/pubmed/28785204

114. Kim SY, Kelland EE, Kim J hong, Lund BT, Chang X, Wang K, et al. The influence of retinoic acid on the human oligodendrocyte precursor cells by RNA-sequencing. Biochem Biophys reports [Internet]. Elsevier B.V.; 2017;9:166–72. Available from: http://dx.doi.org/10.1016/j.bbrep.2016.12.004

115. Zhang YW, Denham J, Thies RS. Oligodendrocyte progenitor cells derived from human embryonic stem cells express neurotrophic factors. Stem Cells Dev. 2006;15:943–52.

116. Chou AP, Chowdhury R, Li S, Chen W, Kim AJ, Piccioni DE, et al. Identification of retinol binding protein 1 promoter hypermethylation in isocitrate dehydrogenase 1 and 2 mutant gliomas. J Natl Cancer Inst [Internet]. 2012;104:1458–69. Available from: http://www.ncbi.nlm.nih.gov/pubmed/22945948

117. Singec I, Crain AM, Hou J, Tobe BTD, Talantova M, Winquist AA, et al. Quantitative Analysis of Human Pluripotency and Neural Specification by In-Depth (Phospho)Proteomic Profiling. Stem Cell Reports [Internet]. The Authors; 2016;7:527–42. Available from: http://dx.doi.org/10.1016/j.stemcr.2016.07.019

118. Hoffmann SA, Hos D, Küspert M, Lang RA, Lovell-Badge R, Wegner M, et al. Stem cell factor Sox2 and its close relative Sox3 have differentiation functions in oligodendrocytes. Development [Internet]. 2014;141:39–50. Available from: http://www.ncbi.nlm.nih.gov/pubmed/24257626

119. Xiao M-L, Liu J-Q, Chen C. [Effect of tumor necrosis factor-related apoptosis-inducing ligand on developing human oligodendrocytes in culture]. Mol Biol (Mosk) [Internet]. 2014;48:963–9. Available from: http://www.ncbi.nlm.nih.gov/pubmed/25845236

120. Chaerkady R, Letzen B, Renuse S, Sahasrabuddhe NA, Kumar P, All AH, et al. Quantitative temporal proteomic analysis of human embryonic stem cell differentiation into oligodendrocyte progenitor cells. Proteomics [Internet]. 2011;11:4007–20. Available from: http://www.ncbi.nlm.nih.gov/pubmed/21770034

121. Stoffels JMJ, Hoekstra D, Franklin RJM, Baron W, Zhao C. The EIIIA domain from astrocyte-derived fibronectin mediates proliferation of oligodendrocyte progenitor cells following CNS demyelination. Glia [Internet]. 2015;63:242–56. Available from: http://www.ncbi.nlm.nih.gov/pubmed/25156142

122. Domingues HS, Portugal CC, Socodato R, Relvas JB. Oligodendrocyte, Astrocyte, and Microglia Crosstalk in Myelin Development, Damage, and Repair. Front cell Dev Biol [Internet]. 2016;4:71. Available from: http://www.ncbi.nlm.nih.gov/pubmed/27551677

123. Sisková Z, Yong VW, Nomden A, van Strien M, Hoekstra D, Baron W. Fibronectin attenuates process outgrowth in oligodendrocytes by mislocalizing MMP-9 activity. Mol Cell Neurosci [Internet]. 2009;42:234–42. Available from: http://www.ncbi.nlm.nih.gov/pubmed/19607919

124. Stoffels JMJ, de Jonge JC, Stancic M, Nomden A, van Strien ME, Ma D, et al. Fibronectin aggregation in multiple sclerosis lesions impairs remyelination. Brain [Internet]. 2013;136:116–31. Available from: http://www.ncbi.nlm.nih.gov/pubmed/23365094

125. Tripathi A, Parikh ZS, Vora P, Frost EE, Pillai PP. pERK1/2 Peripheral Recruitment and Filopodia Protrusion Augment Oligodendrocyte Progenitor Cell Migration: Combined Effects of PDGF-A and Fibronectin. Cell Mol Neurobiol [Internet]. Springer US; 2017;37:183–94. Available from: http://www.ncbi.nlm.nih.gov/pubmed/26993510

126. Logan TT, Villapol S, Symes AJ. TGF-β superfamily gene expression and induction of the Runx1 transcription factor in adult neurogenic regions after brain injury. PLoS One [Internet]. 2013;8:e59250. Available from: http://www.ncbi.nlm.nih.gov/pubmed/23555640

127. Czopka T, von Holst A, Ffrench-Constant C, Faissner A. Regulatory mechanisms that mediate tenascin C-dependent inhibition of oligodendrocyte precursor differentiation. J Neurosci [Internet]. 2010;30:12310–22. Available from: http://www.ncbi.nlm.nih.gov/pubmed/20844127

128. Faissner A, Reinhard J. The extracellular matrix compartment of neural stem and

glial progenitor cells. Glia [Internet]. 2015;63:1330–49. Available from: http://www.ncbi.nlm.nih.gov/pubmed/25913849

129. Najm FJ, Zaremba A, Caprariello A V., Nayak S, Freundt EC, Scacheri PC, et al. Rapid and robust generation of functional oligodendrocyte progenitor cells from epiblast stem cells. Nat Methods [Internet]. 2011;8:957–62. Available from: http://www.ncbi.nlm.nih.gov/pubmed/21946668

130. Gao Y-F, Mao X-Y, Zhu T, Mao C-X, Liu Z-X, Wang Z-B, et al. COL3A1 and SNAP91: novel glioblastoma markers with diagnostic and prognostic value. Oncotarget [Internet]. 2016;7:70494–503. Available from: http://www.ncbi.nlm.nih.gov/pubmed/27655637

131. Curtis R, Hardy R, Reynolds R, Spruce BA, Wilkin GP. Down-regulation of GAP-43 During Oligodendrocyte Development and Lack of Expression by Astrocytes In Vivo: Implications for Macroglial Differentiation. Eur J Neurosci [Internet]. 1991;3:876–86. Available from: http://www.ncbi.nlm.nih.gov/pubmed/12106454

132. Hu M-L, Zhu H-M, Zhang Q-L, Liu J-J, Ding Y, Zhong J-M, et al. Exploring the Mechanisms of Electroacupuncture-Induced Analgesia through RNA Sequencing of the Periaqueductal Gray. Int J Mol Sci [Internet]. 2017;19:1–44. Available from: http://www.ncbi.nlm.nih.gov/pubmed/29295561

133. Tsukushi S, Nishida Y, Sugiura H, Nakashima H, Ishiguro N. Soft tissue sarcomas of the chest wall. J Thorac Oncol [Internet]. International Association for the Study of Lung Cancer; 2009;4:834–7. Available from: http://dx.doi.org/10.1097/JTO.0b013e3181a97da3

134. Liu T, Zhao Y, Tang N, Feng R, Yang X, Lu N, et al. Pax6 directly down-regulates Pcsk1n expression thereby regulating PC1/3 dependent proinsulin processing. PLoS One [Internet]. 2012;7:e46934. Available from: http://www.ncbi.nlm.nih.gov/pubmed/23056534

135. Marques S, Zeisel A, Codeluppi S, van Bruggen D, Mendanha Falcão A, Xiao L, et al. Oligodendrocyte heterogeneity in the mouse juvenile and adult central nervous system. Science [Internet]. 2016;352:1326–9. Available from:

https://www.sciencemag.org/lookup/doi/10.1126/science.aaf6463

136. Hu J-G, Wang Y-X, Zhou J-S, Chen C-J, Wang F-C, Li X-W, et al. Differential gene expression in oligodendrocyte progenitor cells, oligodendrocytes and type II astrocytes. Tohoku J Exp Med [Internet]. 2011;223:161–76. Available from: http://www.ncbi.nlm.nih.gov/pubmed/21372517

137. Lager AM, Corradin OG, Cregg JM, Elitt MS, Shick HE, Clayton BLL, et al. Rapid functional genetics of the oligodendrocyte lineage using pluripotent stem cells. Nat Commun [Internet]. Springer US; 2018;9:3708. Available from: http://dx.doi.org/10.1038/s41467-018-06102-7

138. Thangaraj MP, Furber KL, Gan JK, Ji S, Sobchishin L, Doucette JR, et al. RNA-binding Protein Quaking Stabilizes Sirt2 mRNA during Oligodendroglial Differentiation. J Biol Chem [Internet]. 2017;292:5166–82. Available from: http://www.ncbi.nlm.nih.gov/pubmed/28188285

139. Lehotzky A, Lau P, Tokési N, Muja N, Hudson LD, Ovádi J. Tubulin polymerization-promoting protein (TPPP/p25) is critical for oligodendrocyte differentiation. Glia [Internet]. 2010;58:157–68. Available from: http://www.ncbi.nlm.nih.gov/pubmed/19606501

140. McKinnon RD, Piras G, Ida JA, Dubois-Dalcq M. A role for TGF-beta in oligodendrocyte differentiation. J Cell Biol [Internet]. 1993;121:1397–407. Available from: http://www.ncbi.nlm.nih.gov/pubmed/8509457

141. Gravel M, Peterson J, Yong VW, Kottis V, Trapp B, Braun PE. Overexpression of 2',3'-cyclic nucleotide 3'-phosphodiesterase in transgenic mice alters oligodendrocyte development and produces aberrant myelination. Mol Cell Neurosci [Internet]. 1996;7:453–66. Available from: http://www.ncbi.nlm.nih.gov/pubmed/8875429

142. Miyamoto Y, Yamauchi J, Chan JR, Okada A, Tomooka Y, Hisanaga S, et al. Cdk5 regulates differentiation of oligodendrocyte precursor cells through the direct phosphorylation of paxillin. J Cell Sci [Internet]. 2007;120:4355–66. Available from: http://www.ncbi.nlm.nih.gov/pubmed/18042622

143. Yu S, Levi L, Siegel R, Noy N. Retinoic acid induces neurogenesis by activating

both retinoic acid receptors (RARs) and peroxisome proliferator-activated receptor β/δ (PPARβ/δ). J Biol Chem [Internet]. 2012;287:42195–205. Available from: http://www.ncbi.nlm.nih.gov/pubmed/23105114

144. Maussion G, Diallo AB, Gigek CO, Chen ES, Crapper L, Théroux J-F, et al. Investigation of genes important in neurodevelopment disorders in adult human brain. Hum Genet [Internet]. Springer Berlin Heidelberg; 2015;134:1037–53. Available from: http://www.ncbi.nlm.nih.gov/pubmed/26194112

145. Lin J-C, Tsai J-T, Chao T-Y, Ma H-I, Liu W-H. Musashi-1 Enhances Glioblastoma Migration by Promoting ICAM1 Translation. Neoplasia [Internet]. The Authors; 2019;21:459–68. Available from: https://doi.org/10.1016/j.neo.2019.02.006

146. Kimura A, Matsuda T, Sakai A, Murao N, Nakashima K. HMGB2 expression is associated with transition from a quiescent to an activated state of adult neural stem cells. Dev Dyn [Internet]. 2018;247:229–38. Available from: http://www.ncbi.nlm.nih.gov/pubmed/28771884

147. Sun Y-M, Cooper M, Finch S, Lin H-H, Chen Z-F, Williams BP, et al. Rest-mediated regulation of extracellular matrix is crucial for neural development. PLoS One [Internet]. 2008;3:e3656. Available from: http://www.ncbi.nlm.nih.gov/pubmed/18987749

148. Rajan TS, Scionti D, Diomede F, Piattelli A, Bramanti P, Mazzon E, et al. Prolonged Expansion Induces Spontaneous Neural Progenitor Differentiation from Human Gingiva-Derived Mesenchymal Stem Cells. Cell Reprogram [Internet]. 2017;19:389–401. Available from: http://www.ncbi.nlm.nih.gov/pubmed/29058474

149. Sansom SN, Hébert JM, Thammongkol U, Smith J, Nisbet G, Surani MA, et al. Genomic characterisation of a Fgf-regulated gradient-based neocortical protomap. Development [Internet]. 2005;132:3947–61. Available from: http://www.ncbi.nlm.nih.gov/pubmed/16079153

150. Pham JT, Gallicano GI. Specification of neural cell fate and regulation of neural stem cell proliferation by microRNAs. Am J Stem Cells [Internet]. 2012;1:182–95. Available from: http://www.ncbi.nlm.nih.gov/pubmed/23671807

151. Herberth B, Minkó K, Csillag A, Jaffredo T, Madarász E. SCL, GATA-2 and Lmo2

expression in neurogenesis. Int J Dev Neurosci [Internet]. 2005;23:449–63. Available from: http://www.ncbi.nlm.nih.gov/pubmed/16011889

152. de Souza Martins SC, Agbulut O, Diguet N, Larcher J-C, Paulsen BS, Rehen SK, et al. Dynamic expression of synemin isoforms in mouse embryonic stem cells and neural derivatives. BMC Cell Biol [Internet]. 2011;12:51. Available from: http://www.ncbi.nlm.nih.gov/pubmed/22107957

153. Iwatsuki H, Suda M. Transient expression of keratin during neuronal development in the adult rabbit spinal ganglion. Anat Sci Int [Internet]. 2010;85:46–55. Available from: http://www.ncbi.nlm.nih.gov/pubmed/19629632

154. Kania G, Corbeil D, Fuchs J, Tarasov K V., Blyszczuk P, Huttner WB, et al. Somatic stem cell marker prominin-1/CD133 is expressed in embryonic stem cell-derived progenitors. Stem Cells [Internet]. 2005;23:791–804. Available from: http://www.ncbi.nlm.nih.gov/pubmed/15917475

155. Lin J, Luo J, Redies C. Cadherin-19 expression is restricted to myelin-forming cells in the chicken embryo. Neuroscience [Internet]. Elsevier Inc.; 2010;165:168–78. Available from: http://dx.doi.org/10.1016/j.neuroscience.2009.10.032

156. Zhao L, Ku L, Chen Y, Xia M, LoPresti P, Feng Y. QKI Binds MAP1B mRNA and Enhances MAP1B Expression during Oligodendrocyte Development. Wickens MP, editor. Mol Biol Cell [Internet]. 2006;17:4179–86. Available from: http://www.ncbi.nlm.nih.gov/pubmed/1635361

157. Ricard D, Stankoff B, Bagnard D, Aguera M, Rogemond V, Antoine JC, et al. Differential expression of collapsin response mediator proteins (CRMP/ULIP) in subsets of oligodendrocytes in the postnatal rodent brain. Mol Cell Neurosci [Internet]. 2000;16:324–37. Available from: http://www.ncbi.nlm.nih.gov/pubmed/11085871

158. Ricard D, Rogemond V, Charrier E, Aguera M, Bagnard D, Belin MF, et al. Isolation and expression pattern of human Unc-33-like phosphoprotein 6/collapsin response mediator protein 5 (Ulip6/CRMP5): coexistence with Ulip2/CRMP2 in Sema3a- sensitive oligodendrocytes. J Neurosci [Internet]. 2001;21:7203–14. Available from: http://www.ncbi.nlm.nih.gov/pubmed/11549731

159. Silvestroff L, Franco PG, Pasquini JM. Neural and oligodendrocyte progenitor cells: transferrin effects on cell proliferation. ASN Neuro [Internet]. 2013;5:e00107. Available from: http://journals.sagepub.com/doi/10.1042/AN20120075

160. Deloulme JC, Laeng P, Janet T, Sensenbrenner M, Baudier J. Expression of neuromodulin (GAP-43) and its regulation by basic fibroblast growth factor during the differentiation of O-2A progenitor cells. J Neurosci Res [Internet]. 1993;36:147–62. Available from: http://www.ncbi.nlm.nih.gov/pubmed/8263968

161. Deloulme JC, Janet T, Au D, Storm DR, Sensenbrenner M, Baudier J. Neuromodulin (GAP43): a neuronal protein kinase C substrate is also present in 0-2A glial cell lineage. Characterization of neuromodulin in secondary cultures of oligodendrocytes and comparison with the neuronal antigen. J Cell Biol [Internet]. 1990;111:1559–69. Available from: http://www.ncbi.nlm.nih.gov/pubmed/2170423

162. Gnegy ME. Calmodulin in neurotransmitter and hormone action. Annu Rev Pharmacol Toxicol [Internet]. 1993;33:45–70. Available from: http://www.ncbi.nlm.nih.gov/pubmed/8098596

163. Boczek T, Kozaczuk A, Ferenc B, Kosiorek M, Pikula S, Zylinska L. Gene expression pattern in PC12 cells with reduced PMCA2 or PMCA3 isoform: selective up-regulation of calmodulin and neuromodulin. Mol Cell Biochem [Internet]. 2012;360:89–102. Available from: http://www.ncbi.nlm.nih.gov/pubmed/21912933

164. Boczek T, Ferenc B, Lisek M, Zylinska L. Regulation of GAP43/calmodulin complex formation via calcineurin-dependent mechanism in differentiated PC12 cells with altered PMCA isoforms composition. Mol Cell Biochem [Internet]. Springer US; 2015;407:251–62. Available from: http://www.ncbi.nlm.nih.gov/pubmed/26045175

165. Nicot A, Ratnakar P V., Ron Y, Chen C-C, Elkabes S. Regulation of gene expression in experimental autoimmune encephalomyelitis indicates early neuronal dysfunction. Brain [Internet]. 2003;126:398–412. Available from: http://www.ncbi.nlm.nih.gov/pubmed/12538406

166. Bassil F, Canron M-H, Vital A, Bezard E, Li Y, Greig NH, et al. Insulin resistance and exendin-4 treatment for multiple system atrophy. Brain [Internet]. 2017;140:1420–

36. Available from: http://www.ncbi.nlm.nih.gov/pubmed/28334990

167. Chirivella L, Kirstein M, Ferrón SR, Domingo-Muelas A, Durupt FC, Acosta-Umanzor C, et al. Cyclin-Dependent Kinase 4 Regulates Adult Neural Stem Cell Proliferation and Differentiation in Response to Insulin. Stem Cells [Internet]. 2017;35:2403–16. Available from: http://doi.wiley.com/10.1002/stem.2694

168. Meireles AM, Shen K, Zoupi L, Iyer H, Bouchard EL, Williams A, et al. The Lysosomal Transcription Factor TFEB Represses Myelination Downstream of the Rag-Ragulator Complex. Dev Cell [Internet]. Elsevier Inc.; 2018;47:319-330.e5. Available from: https://doi.org/10.1016/j.devcel.2018.10.003

169. Werner HB, Krämer-Albers E-M, Strenzke N, Saher G, Tenzer S, Ohno-Iwashita Y, et al. A critical role for the cholesterol-associated proteolipids PLP and M6B in myelination of the central nervous system. Glia [Internet]. 2013;61:567–86. Available from: http://www.ncbi.nlm.nih.gov/pubmed/23322581

170. Walko G, Wögenstein KL, Winter L, Fischer I, Feltri ML, Wiche G. Stabilization of the dystroglycan complex in Cajal bands of myelinating Schwann cells through plectin-mediated anchorage to vimentin filaments. Glia [Internet]. 2013;61:1274–87. Available from: http://www.ncbi.nlm.nih.gov/pubmed/23836526

171. Piao J, Major T, Auyeung G, Policarpio E, Menon J, Droms L, et al. Human embryonic stem cell-derived oligodendrocyte progenitors remyelinate the brain and rescue behavioral deficits following radiation. Cell Stem Cell [Internet]. Elsevier Inc.; 2015;16:198–210. Available from: http://dx.doi.org/10.1016/j.stem.2015.01.004

172. Chambers SM, Fasano CA, Papapetrou EP, Tomishima M, Sadelain M, Studer L. Highly efficient neural conversion of human ES and iPS cells by dual inhibition of SMAD signaling. Nat Biotechnol [Internet]. 2009;27:275–80. Available from: http://www.ncbi.nlm.nih.gov/pubmed/19252484

173. Pooyan P, Baharvand H, Javan M, Salekdeh GH. Human embryonic stem cells differentiation into oligodendrocyte lineage cells. protocols.io [Internet]. 2020; Available from: http://dx.doi.org/10.17504/protocols.io.bddni25e.

174. Kruger NJ. The Bradford method for protein quantitation. Methods Mol Biol

[Internet]. 1994;32:9–15. Available from: http://www.ncbi.nlm.nih.gov/pubmed/7951753

175. Wessel D, Flügge UI. A method for the quantitative recovery of protein in dilute solution in the presence of detergents and lipids. Anal Biochem [Internet]. 1984;138:141–3. Available from: http://www.ncbi.nlm.nih.gov/pubmed/6731838

176. Mirzaei M, Pascovici D, Wu JX, Chick J, Wu Y, Cooke B, et al. TMT One-Stop Shop: From Reliable Sample Preparation to Computational Analysis Platform. In: Keerthikumar S, Mathivanan S, editors. Methods Mol Biol [Internet]. New York: Humana Press; 2017. p. 45–66. Available from: http://link.springer.com/10.1007/978-1-4939-6740-7_5

177. Deng L, Pushpitha K, Joseph C, Gupta V, Rajput R, Chitranshi N, et al. Amyloid β Induces Early Changes in the Ribosomal Machinery, Cytoskeletal Organization and Oxidative Phosphorylation in Retinal Photoreceptor Cells. Front Mol Neurosci [Internet]. 2019;12:24. Available from: http://www.ncbi.nlm.nih.gov/pubmed/30853886

178. Mirzaei M, Pushpitha K, Deng L, Chitranshi N, Gupta V, Rajput R, et al. Upregulation of Proteolytic Pathways and Altered Protein Biosynthesis Underlie Retinal Pathology in a Mouse Model of Alzheimer's Disease. Mol Neurobiol [Internet]. Molecular Neurobiology; 2019;56:6017–34. Available from: http://www.ncbi.nlm.nih.gov/pubmed/30707393

179. Emery SJ, Mirzaei M, Vuong D, Pascovici D, Chick JM, Lacey E, et al. Induction of virulence factors in Giardia duodenalis independent of host attachment. Sci Rep [Internet]. Nature Publishing Group; 2016;6:20765. Available from: http://www.ncbi.nlm.nih.gov/pubmed/26867958

180. McAlister GC, Nusinow DP, Jedrychowski MP, Wühr M, Huttlin EL, Erickson BK, et al. MultiNotch MS3 enables accurate, sensitive, and multiplexed detection of differential expression across cancer cell line proteomes. Anal Chem [Internet]. 2014;86:7150–8. Available from: http://www.ncbi.nlm.nih.gov/pubmed/24927332

181. R Core Team. R: A language and environment for statistical computing. Vienna, Austria: R Foundation for Statistical Computing; 2018.

182. Chen H, Boutros PC. VennDiagram: a package for the generation of highly-

customizable Venn and Euler diagrams in R. BMC Bioinformatics [Internet]. 2011;12:35. Available from: https://bmcbioinformatics.biomedcentral.com/articles/10.1186/1471-2105-12-35

183. Stekhoven DJ, Bühlmann P. Missforest-Non-parametric missing value imputation for mixed-type data. Bioinformatics. 2012;28:112–8.

184. Kolde R. Pheatmap: Pretty Heatmaps. R package version 1.0.12. [Internet]. 2019. p. 1–8. Available from: https://cran.r-project.org/package=pheatmap

185. Mudunuri U, Che A, Yi M, Stephens RM. bioDBnet: the biological database network. Bioinformatics [Internet]. 2009;25:555–6. Available from: http://www.ncbi.nlm.nih.gov/pubmed/19129209

186. Windham MP. Cluster Validity for the Fuzzy c-Means Clustering Algorithrm. IEEE Trans Pattern Anal Mach Intell [Internet]. 1982;PAMI-4:357–63. Available from: http://ieeexplore.ieee.org/document/4767266/

187. Subramanian A, Tamayo P, Mootha VK, Mukherjee S, Ebert BL, Gillette MA, et al. Gene set enrichment analysis: a knowledge-based approach for interpreting genome-wide expression profiles. Proc Natl Acad Sci U S A [Internet]. 2005;102:15545–50. Available from: http://www.ncbi.nlm.nih.gov/pubmed/16199517

188. Liberzon A, Subramanian A, Pinchback R, Thorvaldsdóttir H, Tamayo P, Mesirov JP. Molecular signatures database (MSigDB) 3.0. Bioinformatics [Internet]. 2011;27:1739–40. Available from: http://www.ncbi.nlm.nih.gov/pubmed/21546393

189. Phipson B, Lee S, Majewski IJ, Alexander WS, Smyth GK. Robust hyperparameter estimation protects against hypervariable genes and improves power to detect differential expression. Ann Appl Stat [Internet]. 2016;10:946–63. Available from: http://www.ncbi.nlm.nih.gov/pubmed/28367255

190. Gu Z, Gu L, Eils R, Schlesner M, Brors B. circlize Implements and enhances circular visualization in R. Bioinformatics [Internet]. 2014;30:2811–2. Available from: https://academic.oup.com/bioinformatics/article-lookup/doi/10.1093/bioinformatics/btu393

191. Nevin ZS, Factor DC, Karl RT, Douvaras P, Laukka J, Windrem MS, et al.

Modeling the Mutational and Phenotypic Landscapes of Pelizaeus-Merzbacher Disease with Human iPSC-Derived Oligodendrocytes. Am J Hum Genet [Internet]. ElsevierCompany.; 2017;100:617–34. Available from: http://dx.doi.org/10.1016/j.ajhg.2017.03.005

192. Pooyan P; Karamzadeh R; Mirzaei M; Meyfour A; Amirkhan A; Wu Y; Gupta V; Baharvand H; Javan M; Salekdeh GH: Supporting data for "The Dynamic Proteome of Oligodendrocyte Lineage Differentiation Features Planar Cell Polarity and Macroautophagy Pathways" GigaScience Database. 2020. Available from: http://dx.doi.org/10.5524/100798

Figure 1: TMT labeling workflow for comprehensive proteome study of hESCs differentiation into the OL lineage. For in-depth quantitative proteomic profiling of the human OL lineage, the hESC line (Royan H6) first went through a stepwise differentiation process that resulted in the generation of NSCs, NPCs, pre-OPCs, early-OPCs, OPCs, and OLs (in three replicates). Cellular samples were collected at six consecutive time points (d0, d8, d12, d20, d50, d80, and d120). Their protein contents were extracted and denatured, reduced and alkylated, and then subjected to digestion with lysine-C besides trypsin. Peptides were next quantified and reacted with isobaric TMT reagents across individual batches per sample. After labeling, the samples were combined equally and ionized onto a mass spectrometer. Three TMT experiments were conducted to accommodate all the replicates. In the MS^1 spectrum, the peptides were detected as a single and identical precursor ion peak. Following fragmentation, in the MS^2 spectrum, the tags from each differentiation time point produced a unique signature reporter ion. The intensities of these reporter ions were used for the relative quantification of peptides. The identification of peptides was achieved through matching the resulting ion peaks to those indexed fragments in UniProt. The table shows the study design of each TMT experiment. Note that, after Pearson correlation coefficients analysis, the samples analysed in this study have been refined (see **TMT data analysis**). hESC: Human embryonic stem cell; NSC: Neural stem cell; NPC: Neural progenitor cell; OPC: Oligodendrocyte progenitor cell; OL: Oligodendrocyte; ictrl: Inner control; TMT: Tandem mass tag, LC: liquid chromatography; MS: Mass spectrum.

Figure 2: Temporal Profiling of Protein Expression through hESC differentiation into OL lineage. (A) Pearson correlation analysis along with the hierarchical clustering of the 3,855 quantified proteins reveals the biological replicates' cohesion and dynamics of the proteome during OL lineage differentiation. Red colour denotes stronger correlations. (B) Principal component analysis (PCA) reveals a temporal trend in protein expression patterns. The same colour represents different replicates of the same differentiation time point. PC1 and PC2 axes demonstrate 37.54% and 17.45% of variations.

Figure 3: Proteome dynamic landscape of hESC differentiation into OL lineage and expression pattern of marker proteins. Unsupervised clustering of the quantified proteins

using the fuzzy c-means algorithm distinguishes three protein expression profiles (A-, B- and C-left charts) mainly based on protein abundance trends at the initial and final time points. The identified clusters are visualized separately with line charts plotting protein expression level against differentiation time points. Color-coded membership represents how well a single protein expression pattern fits with the general profile of the cluster. GO enrichment analysis of each cluster was performed with respect to the BPs by Enrichr. Some of the overrepresented BPs of each cluster are shown with bar charts (A-, B- and C-right charts). (D) Heat map illustrates the standardized relative protein expression changes of several stage-specific proteins along with differentiation. Markers of early differentiation stages (d0, d8, and d12) are members of cluster 1, which shows a slight decreasing trend. Cluster 2, with a slightly increasing pattern, accommodates CDH2, a marker of NSCs (d8), which is also involved in the regulation of OPCs (d80) proliferation and OL (d120) myelination. The heat map also reveals that (pre-)OPC and OL markers are generally assigned to the two increasing clusters (clusters 2 and 3). n: Represents the protein counts in a cluster; Eph: Ephrin; NADP: Nicotinamide adenine dinucleotide phosphate; hESC: Human embryonic stem cell; NSPC: Neural Stem and Progenitor Cell; OPC: Oligodendrocyte progenitor cell; OL: Oligodendrocyte; GO: Gene Ontology; BP: Biological processes.

Figure 4: The dynamics of Wnt signaling associated proteins during OL lineage differentiation. (A) Heat map shows the standardized relative expression changes of Wnt signaling associated proteins along the OL lineage differentiation process. The colour of the protein names' bar demonstrates the cluster statue of proteins. Light gray, medium gray, and gray indicate if the protein is fitted for clusters 1, 2 or 3. Most expression changes occurred during the early and late stages. (B) The table shows the active Wnt signaling related biological processes (BP) in OL lineage differentiation. The enrichment analysis of the proteins of each cluster indicates their roles with the Wnt signaling related BPs. Their involvement scores are based on -Log10 of the adjusted p-value. The blank cells show there either were no participation or the -Log10 of the adjusted p-value was more than 0.05.

Figure 5: Expression profile of autophagy associated proteins through oligodendrocyte (OL) lineage differentiation. Heat map shows the standardized relative expression changes of autophagy associated proteins along the OL lineage differentiation process. The colour of the protein names' bar demonstrates the cluster statue of proteins. Light gray, medium gray, and gray indicate if the protein is a fit for cluster 1, 2 or 3. The autophagy related biological processes (BPs) that each protein is implicated in is shown in front of it.

Figure 6: Stage-specific proteins of the oligodendrocyte (OL) lineage differentiation. (A - F) Volcano plots represent the comparison of protein expression at each time point with all other differentiation time points (or stages). The colour code was used for separating differentially expressed proteins (DEPs) and similarly expressed proteins. The red dots demonstrate proteins that were highly up-regulated at that specific stage (mentioned at the top right corner, above the plot) compared to all other stages while the green dots display the proteins with significantly low abundances at that stage, and the light yellow dots indicate the proteins with consistent expression level, Log2 (fold-Change) >1 and -Log10 (adjusted p-value) <0.05.

Figure 7: Dynamic proteome remodeling across the stepwise differentiation of hESC into the oligodendrocyte (OL) lineage cells. (A - E) Volcano plot representations of the log-ratio of protein expression values in two different consecutive differentiation stages. The colour code was used for separating differentially expressed proteins (stage transition specific proteins [STSPs]) and similarly expressed proteins. The red dots demonstrate proteins that highly up-regulated at the achieved stage while the green dots display the proteins with high abundances at its predecessor stage. The selection criteria of Log2 (fold-Change) >1 and -Log10 (adjusted p-value) <0.05 were used to indicate the differentially expressed stage transition proteins. (F) Generally, the STSPs can be classified in 14 protein sets (PS), illustrated as a colour-coded circular track, based on their differential expressions. The circular line between protein names and colour-coded track reveals the stages of the differentiation. As shown by the colour-coded bars, attached to the circular track, 42 of the STSPs are specific to more than one stage transition events. *i.e.*, CRABP1 (the black arrow) abundance increases during d0 (human

embryonic stem cell [hESC] stage) conversion into d8 (neural stem cell [NSC] stage), and its expression differentially down-regulates through d20 (pre-oligodendrocyte progenitor cell [pre-OPC] stage) to d80 (OPC stage) transition. The colour key is placed inside the circle.

Figure 1
Click here to access/download;Figure;Fig 1.pdf

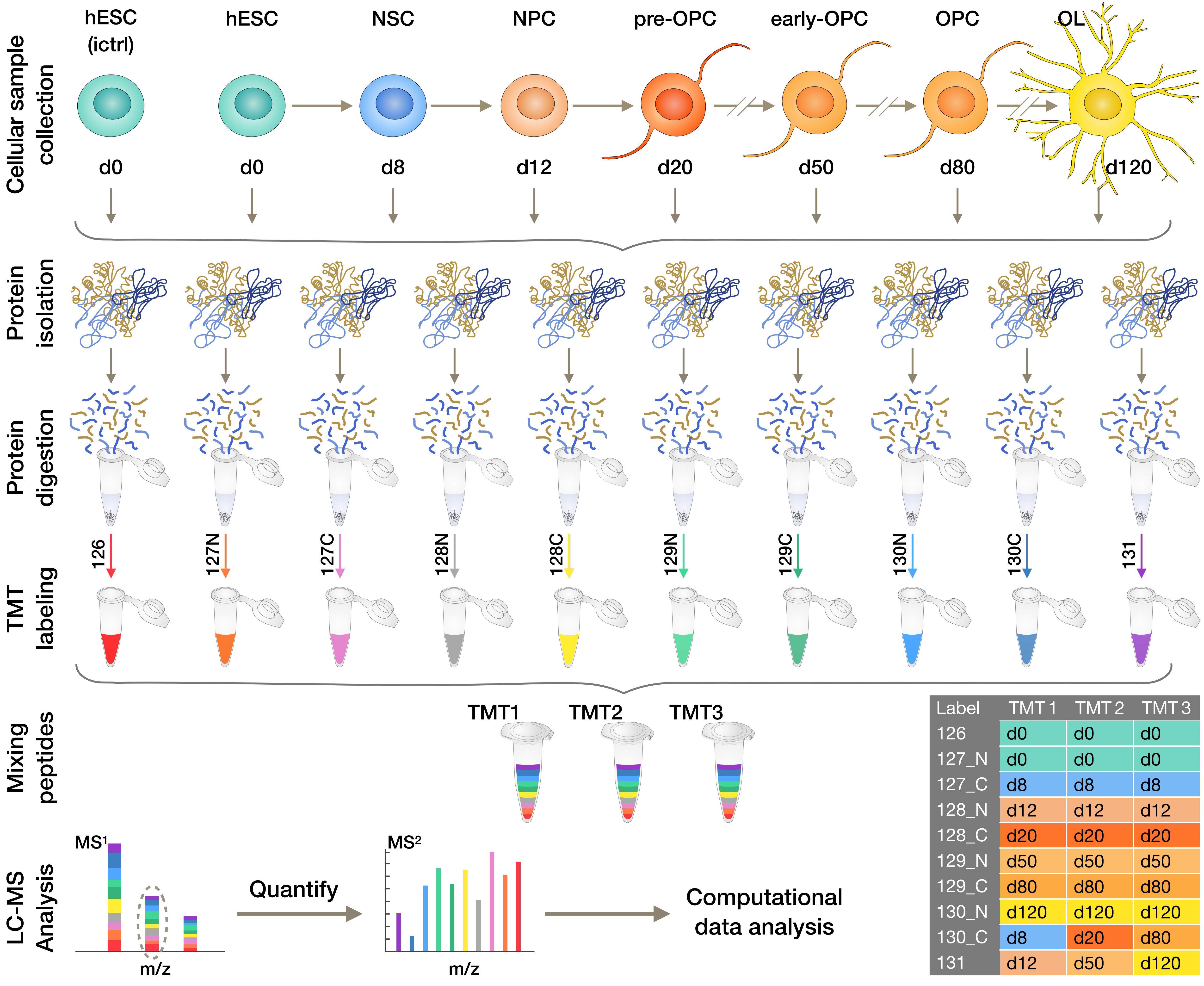

Label	TMT 1	TMT 2	TMT 3
126	d0	d0	d0
127_N	d0	d0	d0
127_C	d8	d8	d8
128_N	d12	d12	d12
128_C	d20	d20	d20
129_N	d50	d50	d50
129_C	d80	d80	d80
130_N	d120	d120	d120
130_C	d8	d20	d80
131	d12	d50	d120

Figure 2

Click here to access/download;Figure;Fig 2.pdf

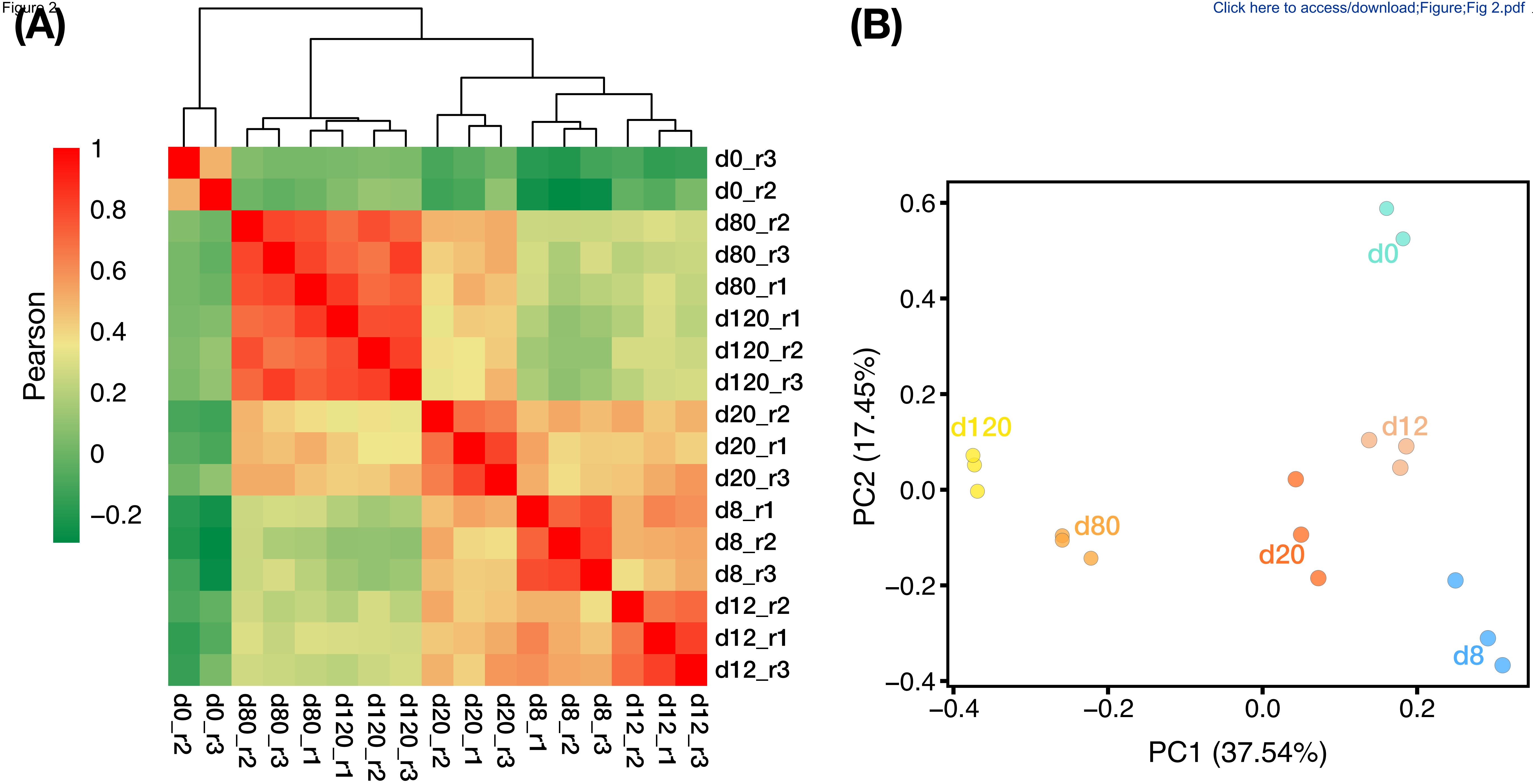

Figure 3

Click here to access/download;Figure;Fig 3.pdf

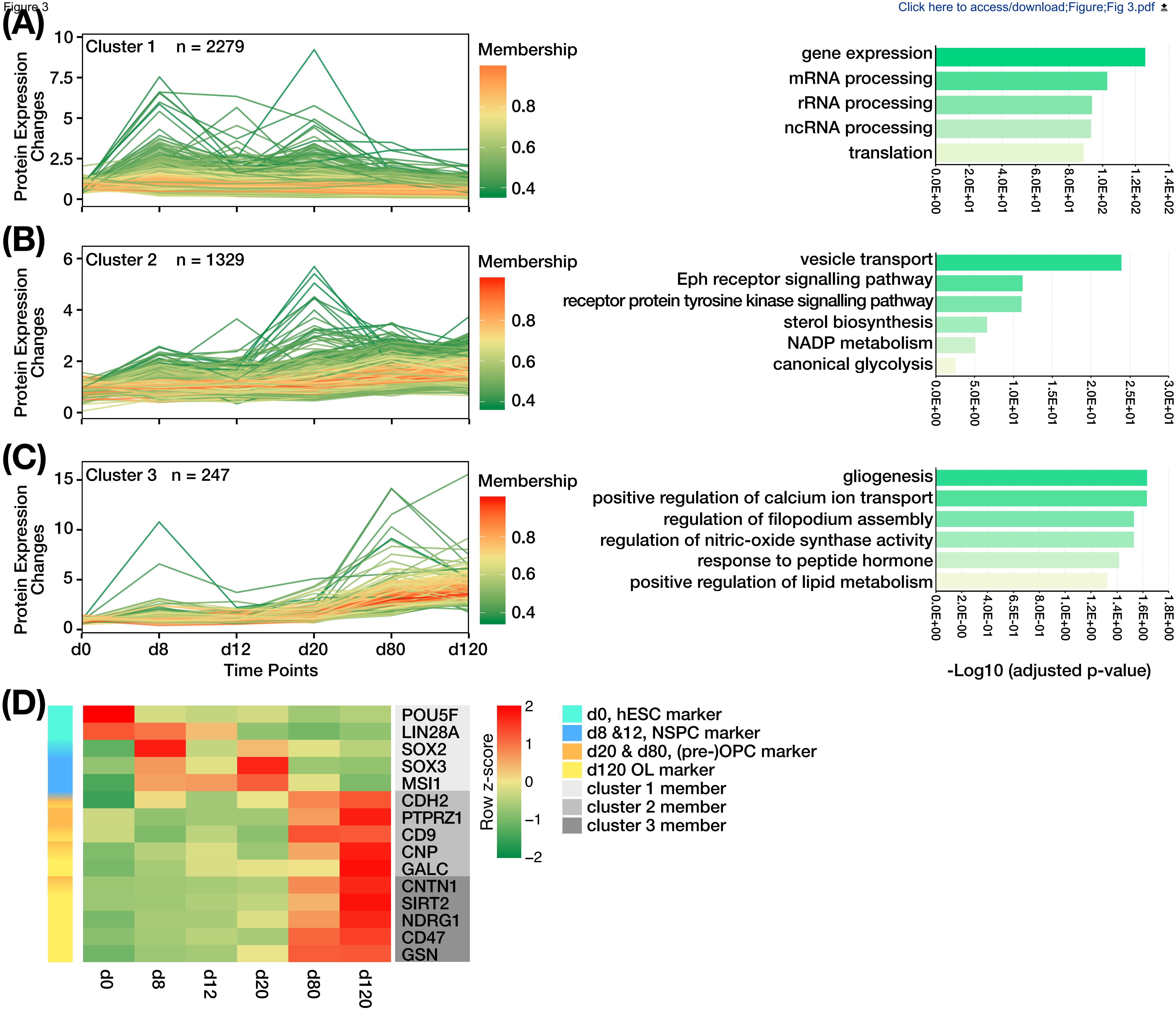

Click here to access/download;Figure;Fig 4.pdf

(A)

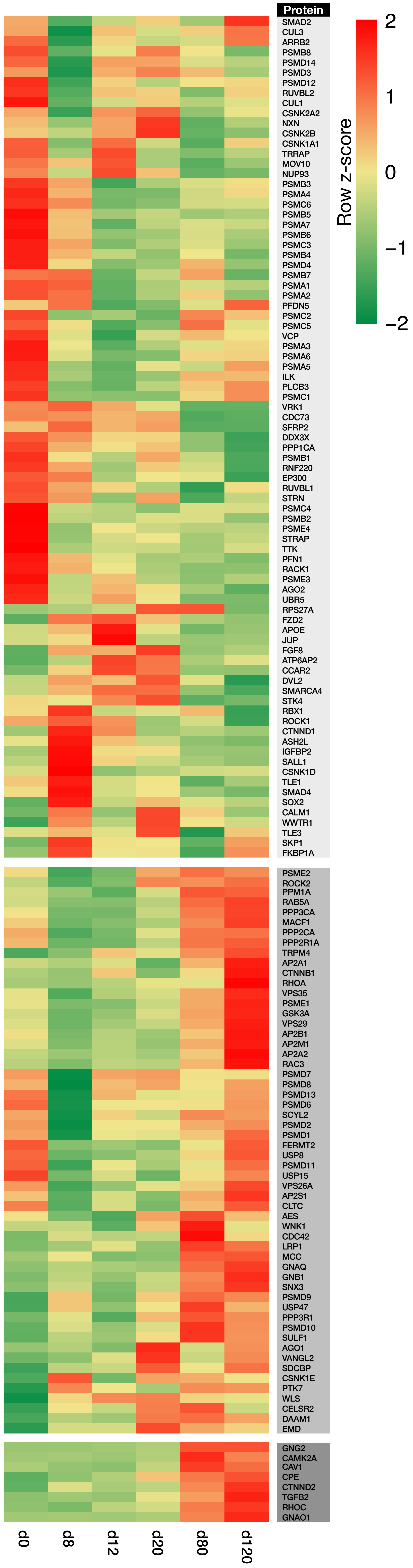

(B)

	positive regulation of canonical Wnt signaling pathway	Wnt signaling pathway, planar cell polarity pathway	positive regulation of Wnt signaling pathway	negative regulation of canonical Wnt signaling pathway	non-canonical Wnt signaling pathway	regulation of canonical Wnt signaling pathway	negative regulation of Wnt signaling pathway
Clus. 1	1.2E-08	3.1E-08	6.8E-08	1.1E-06	1.2E-06	3.9E-06	5.1E-06
Clus. 2		1.3E-05	5.00E-03	1.40E-02	1.3E-05		
Clus. 3							

member of cluster 1 member of cluster 2 member of cluster 3

Click here to access/download;Figure;Fig 5.pdf

Figure 5

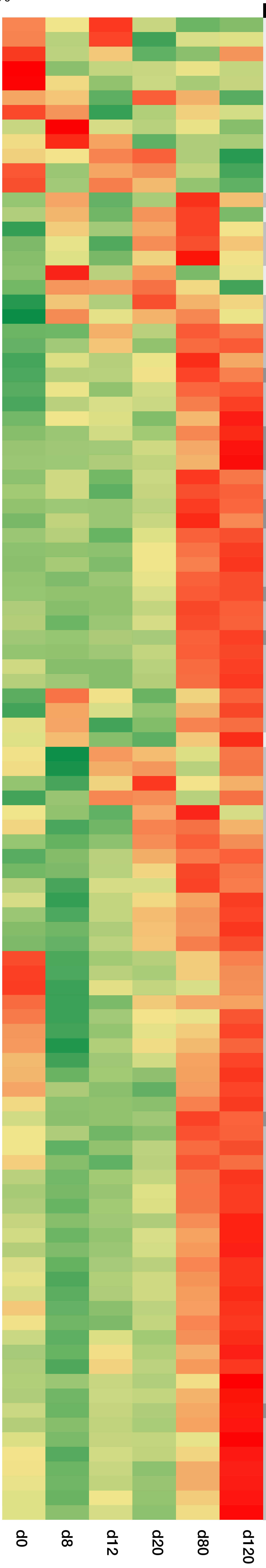

Protein	Term
MFN1	macroautophagy
PIP4K2C	positive regulation of autophagosome assembly; regulation of autophagy
SNX5	regulation of macroautophagy
EIF4G1	negative regulation of autophagy
HSPA8	Chaperone-mediated autophagy; Late endosomal microautophagy
ATG16L1	autophagosome assembly; macroautophagy
CHMP4A	negative regulation of autophagosome assembly; macroautophagy
ATP6V1G1	regulation of macroautophagy
TOMM70	macroautophagy
VPS4B	macroautophagy
PRKAA2	macroautophagy; regulation of macroautophagy; positive regulation of autophagy; positive regulation of macroautophagy
VPS25	macroautophagy
ABL1	autophagy; regulation of autophagy
RAB1B	regulation of autophagosome assembly; autophagy
SNAP29	autophagosome maturation; autophagosome membrane docking
CHMP4B	negative regulation of autophagosome assembly; macroautophagy; autophagy
MTDH	positive regulation of autophagy
TOMM40	macroautophagy
VPS36	macroautophagy
BNIP3	positive regulation of macroautophagy; positive regulation of autophagy; autophagy of mitochondrion; positive regulation of autophagy of mitochondrion
EI24	macroautophagy
IFI16	autophagy; regulation of autophagy
RAB39B	autophagy; regulation of autophagy
ATP6V1E1	regulation of macroautophagy
OPTN	autophagy; positive regulation of autophagy
LAMTOR1	regulation of macroautophagy
SRC	macroautophagy
VPS18	autophagy
GBA	autophagosome organization; regulation of macroautophagy; autophagy; positive regulation of autophagy of mitochondrion in response to mitochondrial depolarization
INS	regulation of autophagy
SIRT2	autophagy; negative regulation of autophagy
HGS	macroautophagy
MLST8	regulation of macroautophagy
MAPT	regulation of autophagy
PIP4K2B	positive regulation of autophagosome assembly; regulation of autophagy
SNX6	regulation of macroautophagy
ATP6V1B2	regulation of macroautophagy
GABARAPL2	autophagosome assembly; autophagosome maturation; macroautophagy; autophagy; autophagy of mitochondrion
RAB1A	autophagosome assembly; autophagy
MAP1LC3B2	Autophagosome assembly; Autophagy of mitochondrion
ATP6V1A	regulation of macroautophagy
PRKAA1	macroautophagy; regulation of macroautophagy; positive regulation of autophagy
DCN	positive regulation of macroautophagy; positive regulation of autophagy
VPS26B	regulation of macroautophagy
RAB5A	regulation of autophagosome assembly
SH3GLB1	positive regulation of autophagy; regulation of macroautophagy; autophagic cell death; autophagy; positive regulation of autophagosome assembly
LAMTOR3	regulation of macroautophagy
STAM	macroautophagy
CHMP2B	macroautophagy; autophagy
VPS4A	macroautophagy
SNF8	macroautophagy
TOMM22	macroautophagy
BCL2	negative regulation of autophagy
CISD2	regulation of autophagy; autophagy of mitochondrion
ATG2B	autophagosome assembly; autophagy of mitochondrion
PIP4K2A	positive regulation of autophagosome assembly; regulation of autophagy
TSG101	autophagosome maturation; macroautophagy
CHMP6	macroautophagy
HDAC6	regulation of autophagy of mitochondrion; regulation of macroautophagy; Parkin-mediated stimulation of mitophagy in response to mitochondrial depolarization
VPS28	macroautophagy
ATP6V1D	regulation of macroautophagy
VPS11	autophagy
AKT1	negative regulation of macroautophagy; negative regulation of autophagy
RAB24	autophagy
ATG5	autophagosome assembly; macroautophagy; autophagy; autophagy of mitochondrion
RAB3GAP2	positive regulation of autophagosome assembly
EIF4G2	negative regulation of autophagy
RAB3GAP1	positive regulation of autophagosome assembly
PIK3C3	autophagosome assembly; macroautophagy; autophagy
VPS26A	regulation of macroautophagy
DNM1L	regulation of autophagy of mitochondrion
VTA1	macroautophagy
CAPN1	regulation of macroautophagy
RAB33B	autophagosome assembly
RAB12	autophagy; positive regulation of macroautophagy
HSPB1	regulation of autophagy
UCHL1	regulation of macroautophagy
PIKFYVE	regulation of autophagosome assembly
VPS16	autophagosome maturation
CHMP2A	macroautophagy
CDK5	regulation of macroautophagy
EXOC8	regulation of macroautophagy
ATG9A	autophagosome assembly; autophagy of mitochondrion
GSK3A	positive regulation of autophagy; regulation of autophagy of mitochondrion
MTOR	regulation of macroautophagy; negative regulation of autophagy
CAPNS1	regulation of macroautophagy
EXOC4	regulation of macroautophagy
VPS35	regulation of macroautophagy
ATG3	autophagosome assembly; macroautophagy; autophagy of mitochondrion
PAFAH1B2	positive regulation of macroautophagy [GO:0016239]
ATP6V1H	regulation of macroautophagy
ATP6V0D1	regulation of macroautophagy
PRKAG1	macroautophagy; regulation of macroautophagy
RPTOR	regulation of macroautophagy
VPS33A	autophagosome maturation
RRAGA	regulation of autophagy (GO:0010506); Negative regulation of autophagy (GO:0010507)
EXOC1	regulation of macroautophagy
ATG7	autophagosome assembly; macroautophagy; autophagy; autophagy of mitochondrion; positive regulation of autophagy
ATP6V0A1	regulation of macroautophagy
ATP6V1C1	regulation of macroautophagy
EXOC7	regulation of macroautophagy
MFN2	macroautophagy
RAB7A	autophagosome assembly

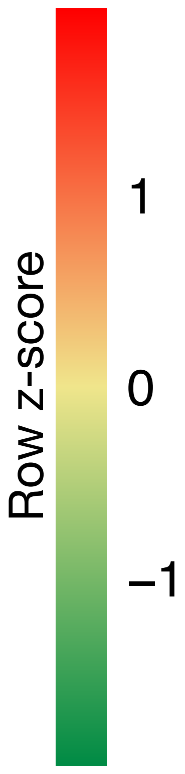

Figure 6

Click here to access/download;Figure;Fig 6.pdf

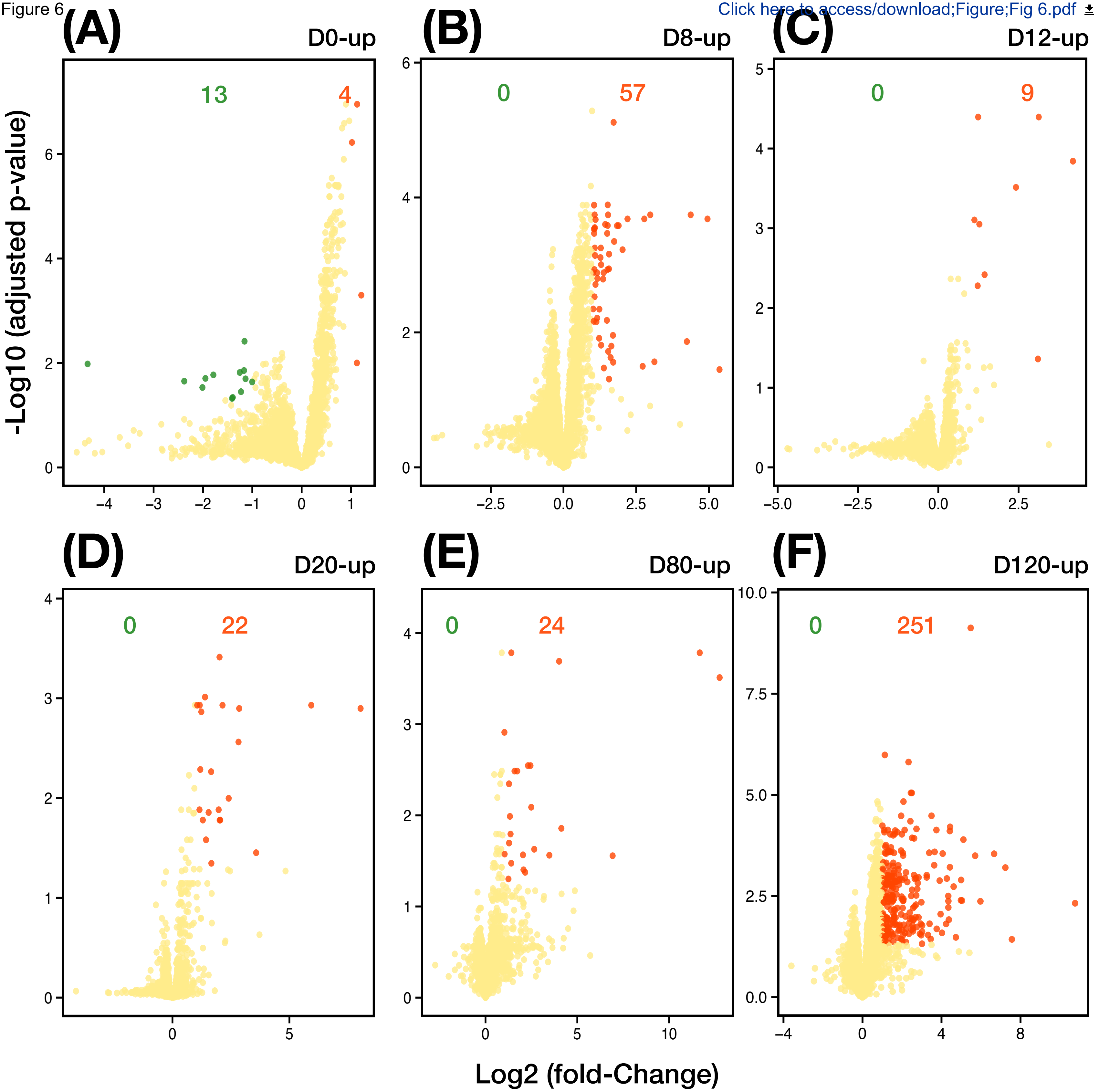

Figure 7

Click here to access/download;Figure;Fig 7.pdf

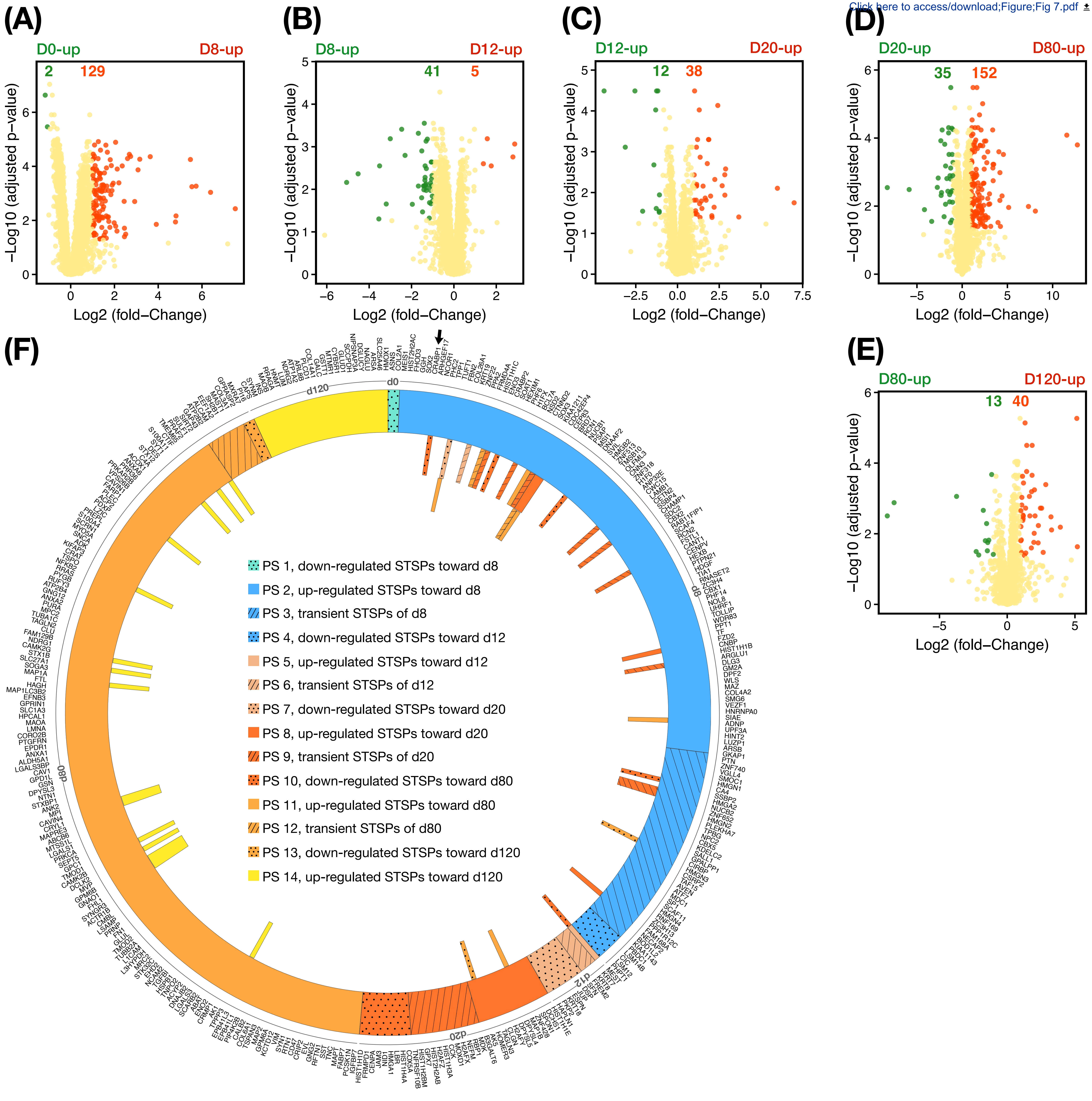

Figure S1

Click here to access/download
Supplementary Material
Supplementary_Figure_S1.pdf

Figure S2

Click here to access/download
Supplementary Material
Supplementary_Figure_S2.pdf

Figure S3

Click here to access/download

Supplementary Material

Supplementary_Figure_S3.pdf

Figure S4

Click here to access/download
Supplementary Material
Supplementary_Figure_S4.pdf

Figure S5

Click here to access/download
Supplementary Material
Fig S5.pdf

Supplementary Figure S6

Click here to access/download
Supplementary Material
Fig S6.jpg

Table S1

Click here to access/download
Supplementary Material
Table S1.xlsx

Table S2

Click here to access/download
Supplementary Material
Table S2.xlsx

Table S3

Click here to access/download
Supplementary Material
Table S3.xlsx

Table S4

Click here to access/download
Supplementary Material
Table S4.xlsx

Table S5

Click here to access/download
Supplementary Material
Table S5.xlsx

Table S6

Click here to access/download
Supplementary Material
Table S6.xlsx

Table S7

Click here to access/download
Supplementary Material
Table S7.xlsx

Table S8

Click here to access/download
Supplementary Material
Table S8.xlsx

Table S9

Click here to access/download
Supplementary Material
Table S9.xlsx

Table S10

Click here to access/download
Supplementary Material
Table S10.xlsx

Table S11

Click here to access/download
Supplementary Material
Table S11.xlsx

Table S12

Click here to access/download
Supplementary Material
Table S12.xlsx
