# Supplementary figures and images for: The Dynamic Proteome of Oligodendrocyte Lineage Differentiation Features Planar Cell Polarity and Macroautophagy Pathways
Source: Gigascience. 2020 Oct 31;9(11):giaa116. doi: 10.1093/gigascience/giaa116 (PMC7601170; doi:10.1093/gigascience/giaa116)

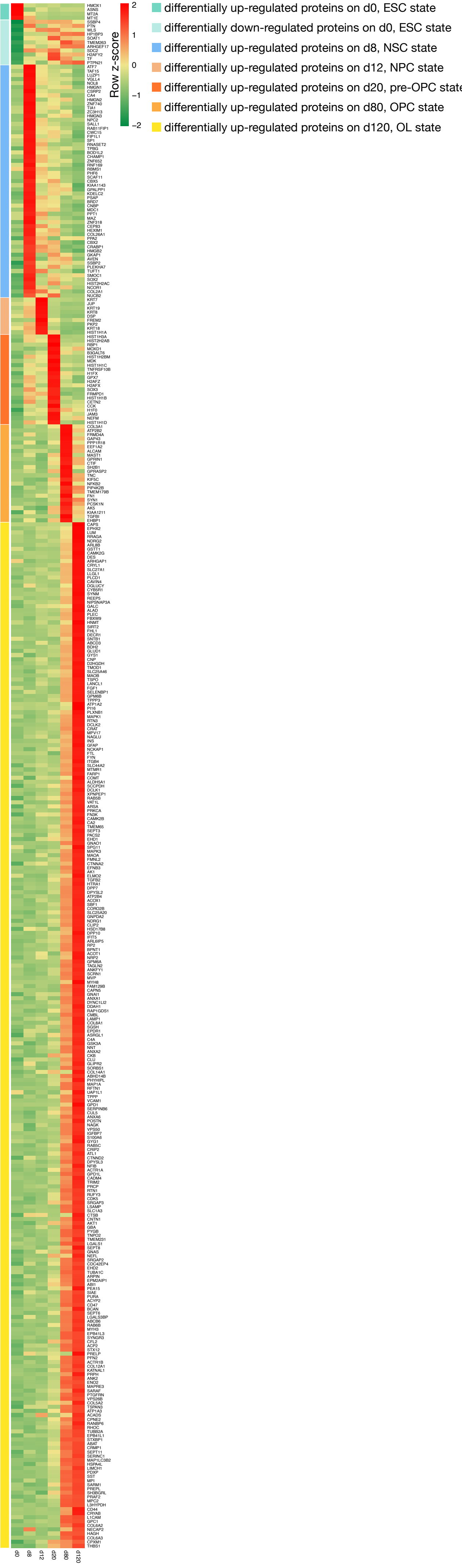

Supplement: giaa116_Supplemental_Figures_and_Tables [file giaa116_supplemental_figures_and_tables.zip › Fig S5.pdf]

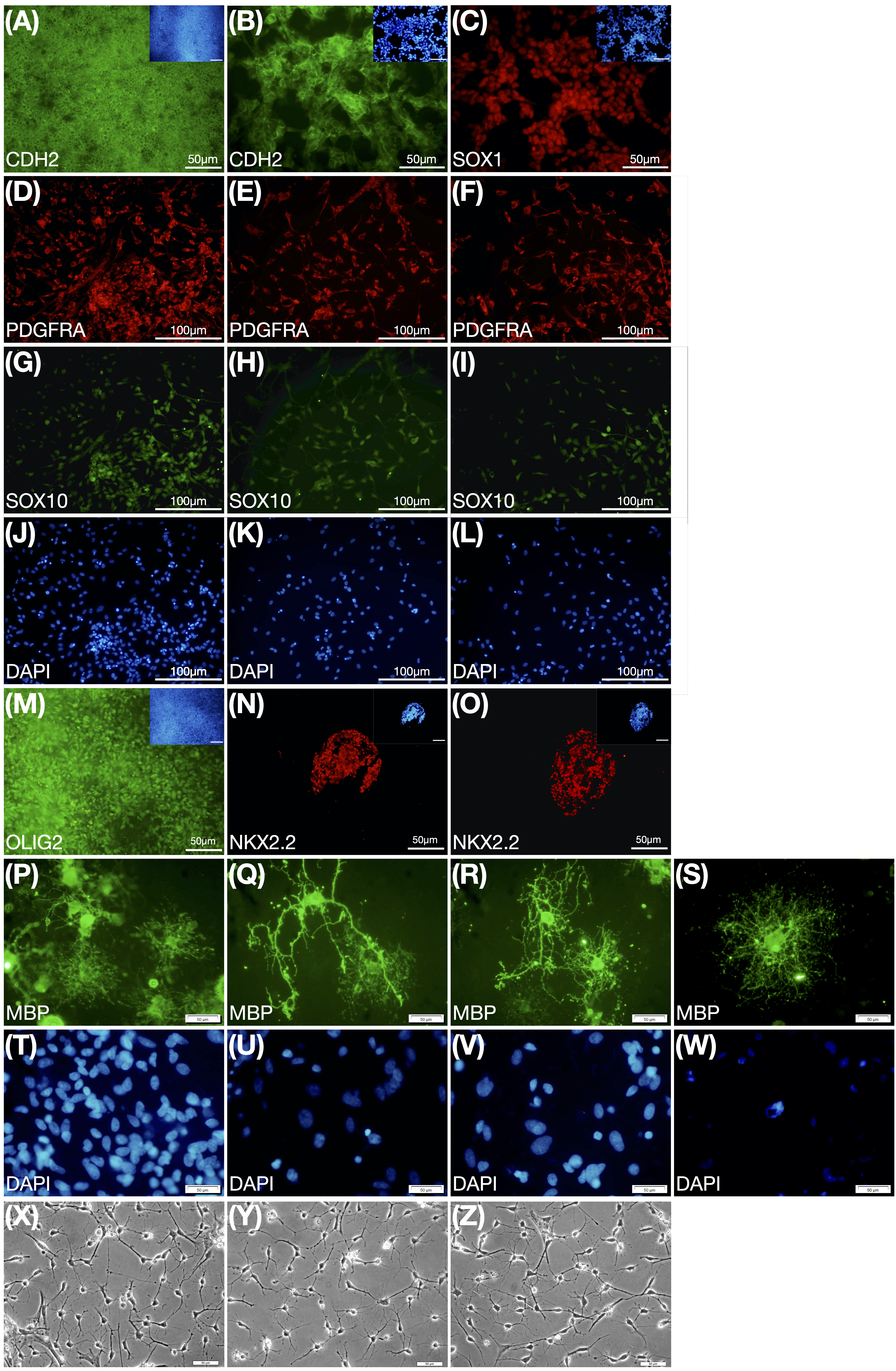

Supplement: giaa116_Supplemental_Figures_and_Tables [file giaa116_supplemental_figures_and_tables.zip › Fig S6.jpg]

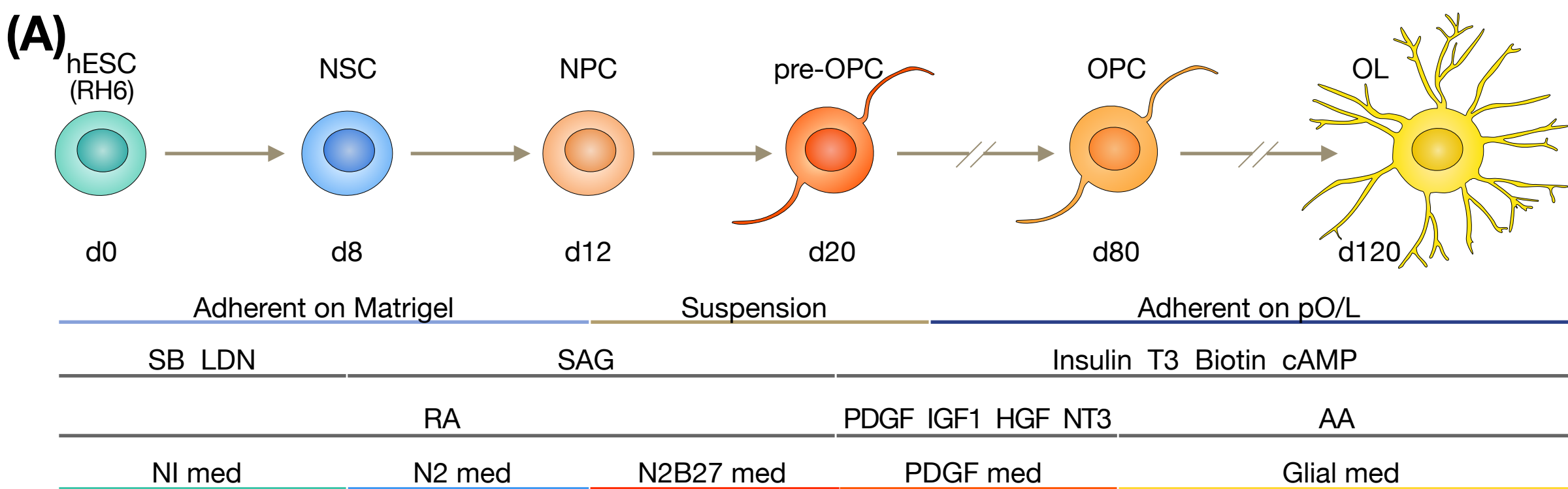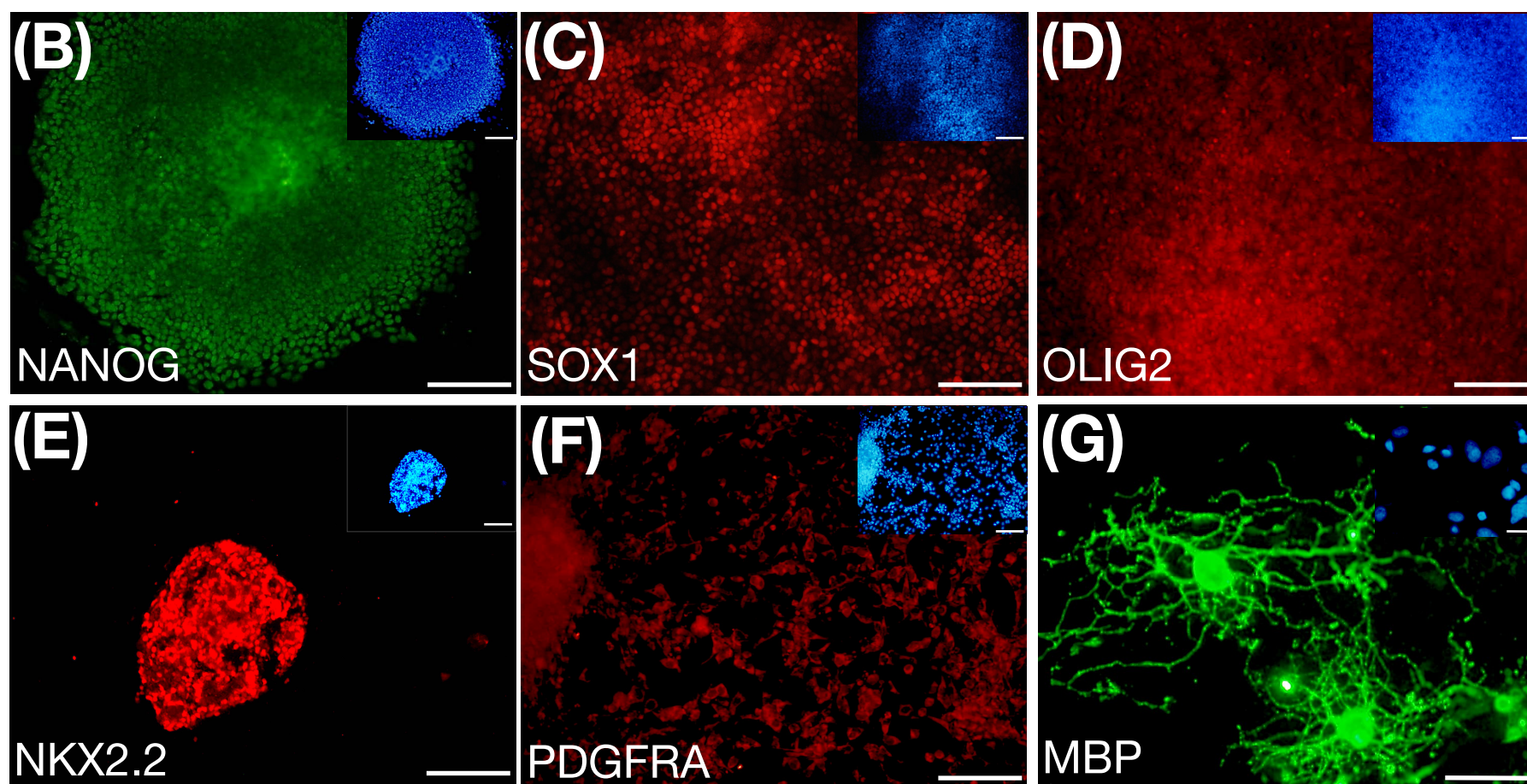

Supplement: giaa116_Supplemental_Figures_and_Tables [file giaa116_supplemental_figures_and_tables.zip › Supplementary_Figure_S1.pdf]

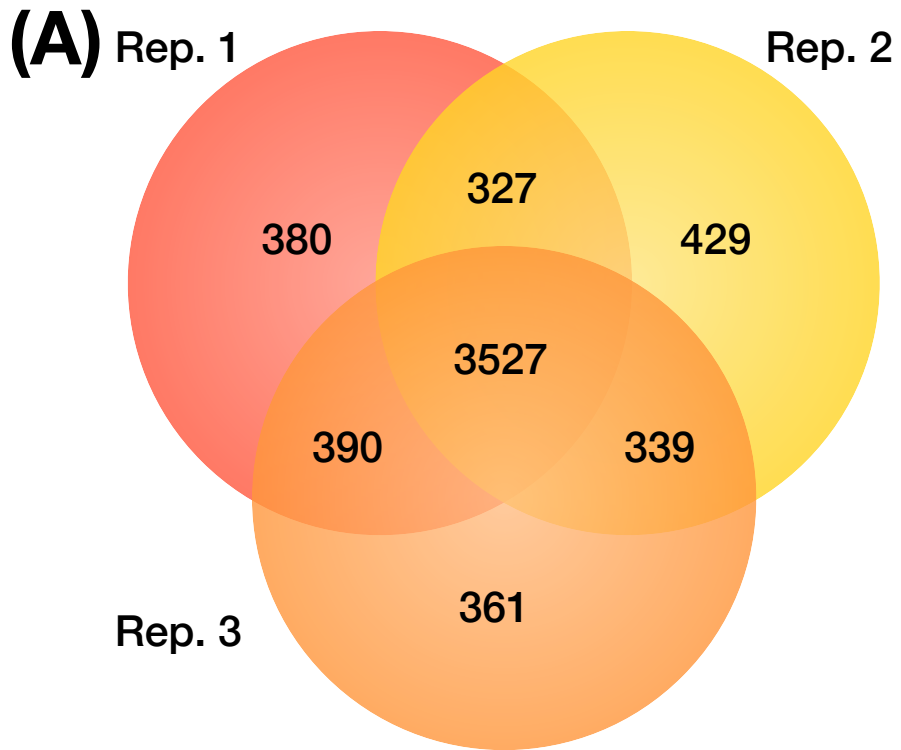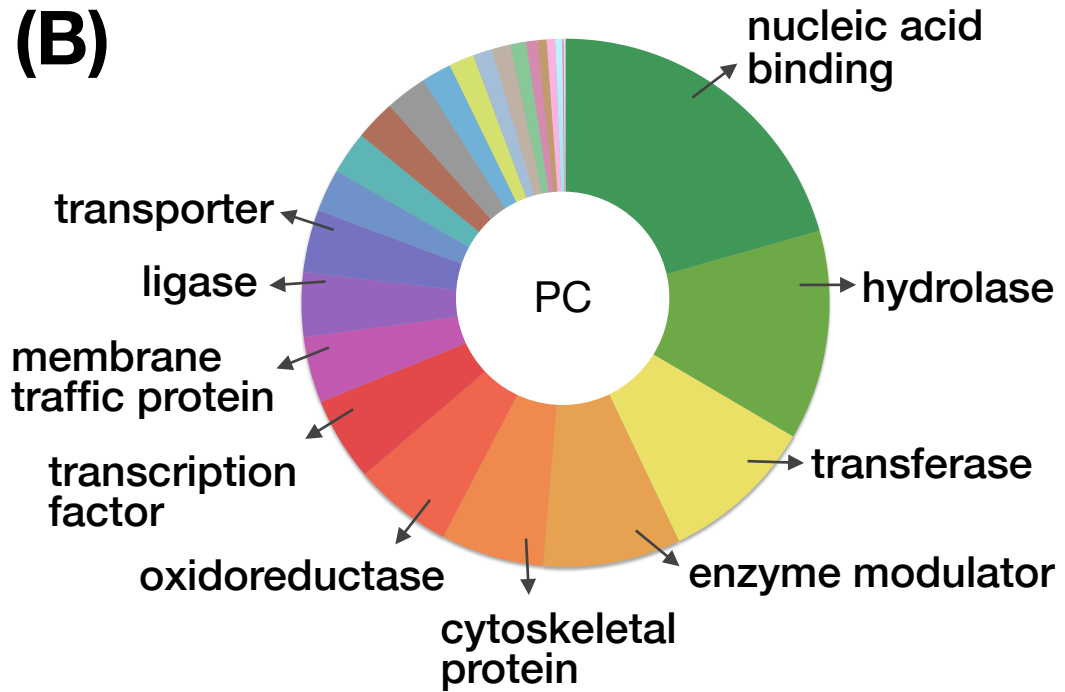

Supplement: giaa116_Supplemental_Figures_and_Tables [file giaa116_supplemental_figures_and_tables.zip › Supplementary_Figure_S2.pdf]

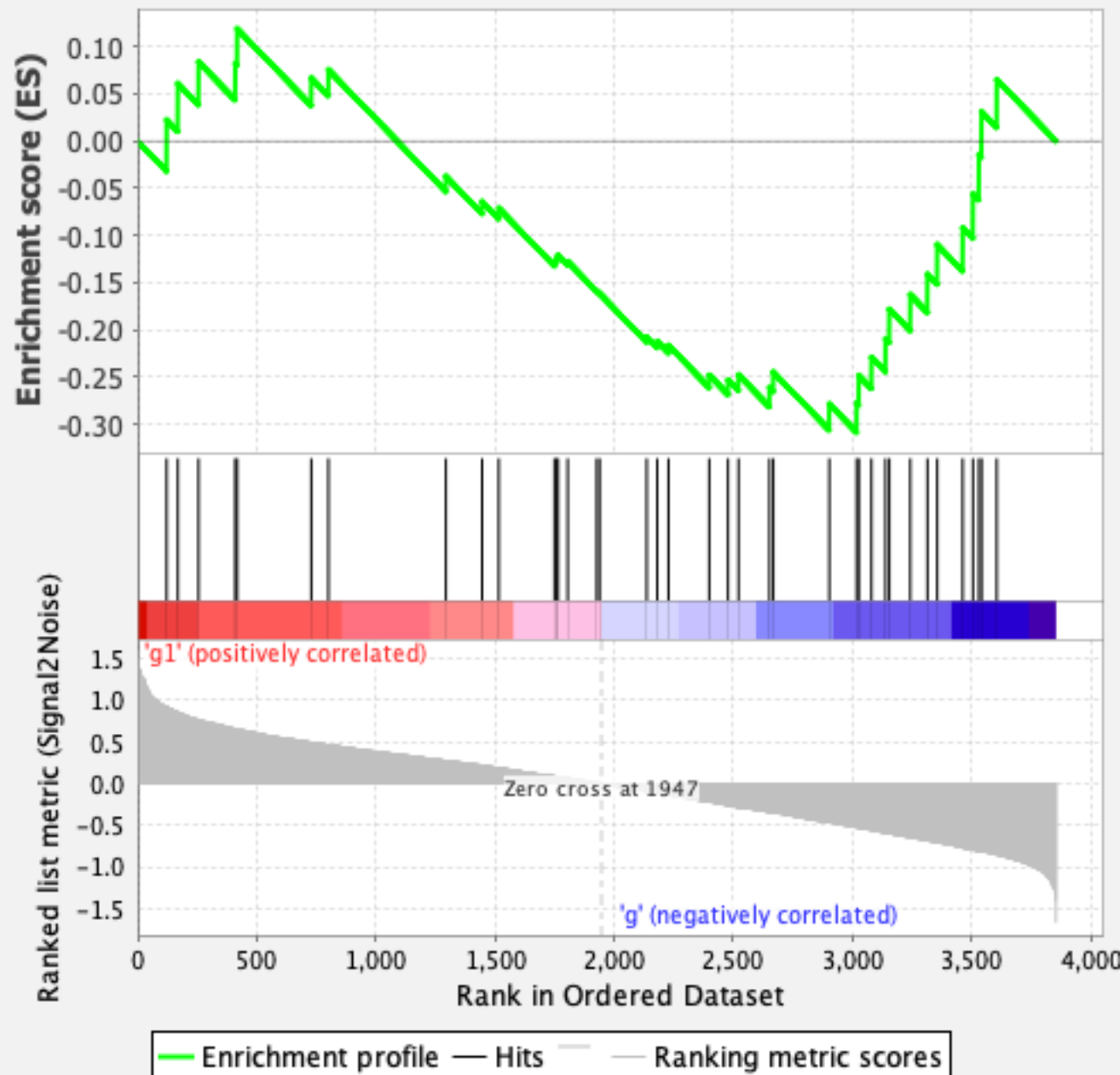

Supplement: giaa116_Supplemental_Figures_and_Tables [file giaa116_supplemental_figures_and_tables.zip › Supplementary_Figure_S3.pdf]

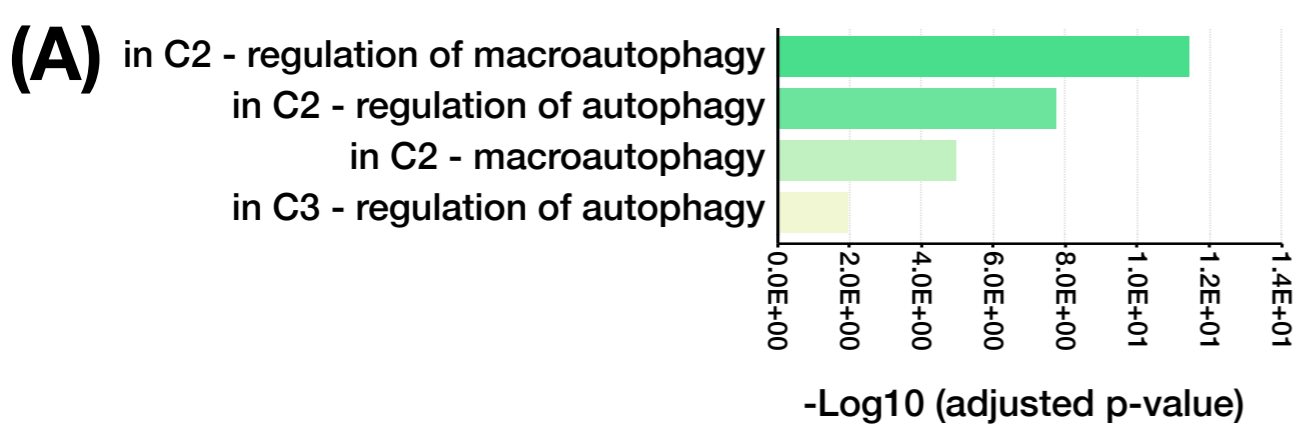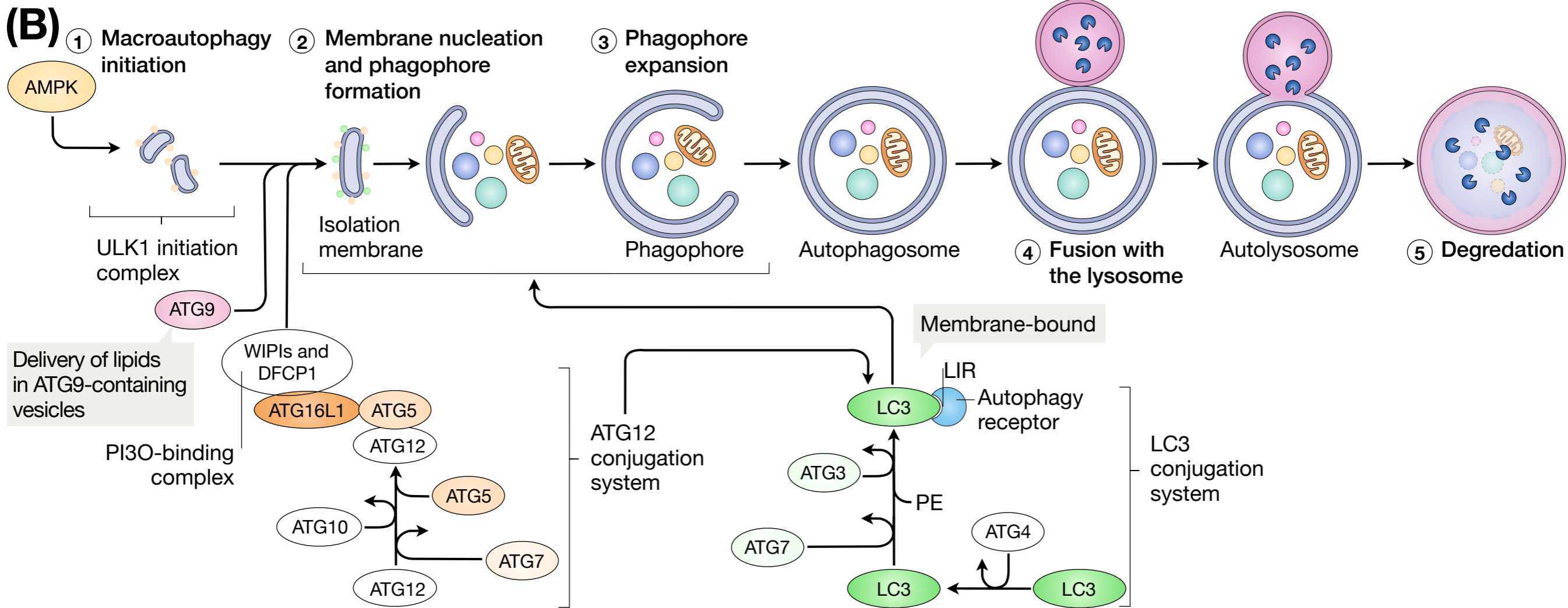

Supplement: giaa116_Supplemental_Figures_and_Tables [file giaa116_supplemental_figures_and_tables.zip › Supplementary_Figure_S4.pdf]
