# Supplementary material for: The Dynamic Proteome of Oligodendrocyte Lineage Differentiation Features Planar Cell Polarity and Macroautophagy Pathways
Source: Gigascience. 2020 Oct 31;9(11):giaa116. doi: 10.1093/gigascience/giaa116 (PMC7601170; doi:10.1093/gigascience/giaa116)
Supplement: giaa116_Response_to_Reviewer_Comments_Original_Submission [file giaa116_response_to_reviewer_comments_original_submission.pdf]

Author's Response To Reviewer Comments

Close

July 21, 2020
Dear Prof. Laurie Goodman,

We wish to thank you for the thorough review of our manuscript, " The Dynamic Proteome of Oligodendrocyte Lineage Differentiation Features Planar Cell Polarity and Macroautophagy Pathways". We are pleased that the reviewer has found our review article informative, comprehensive, and timely. As seen in the below point-by-point response to reviewer, we have revised the enclosed manuscript in accord with the comments. We also attach a "revised manuscript with track changes" as supplementary material to highlight the revised sections. Furthermore, we added 7 new supplementary tables with multiple sheets to address reviewers' comments.
We trust that the manuscript may now be accepted for publication.

Sincerely yours,
Hosseini Salekdeh

Prof. Dr. Ghasem Hosseini Salekdeh
Professor in Systems Biology
Royan Institute for Stem Cell Biology and Technology
Honorary Professor
Macquarie University, Sydney, Australia
Email: hsalekdeh@gmail.com
Hosseini.salekdeh@mq.edu.au

Response to comments:

Reviewer #1:
The manuscript by Pooyan et al describes TMT-based quantitative proteome of oligodendrocyte lineage (OL) differentiation by analyzing human embryonic stem cells (hESCs) through several developmental OL stages. Using clustering and ontological analyses, the authors found hundreds of proteins that were differentially expressed between these stages. These proteins pointed towards the importance of Planar Cell Polarity (PCP) and macro-autophagy in OL differentiation.
Strengths -
Generally, the manuscript is well written and the objective is clear. This seems to be a comprehensive proteomics approach to study OL differentiation. The comprehensive multiplexed proteomics study is the big strength of the manuscript. The data is deposited in public repository.
Limitations-
The rationale for biomarkers was not clear. Some analysis methods/steps are not explained in requisite detail for a general user. This data serves as a rich resource as claimed by authors, but it is not available for users in an easily digestible/usable format. Also, the biological description of proteins was felt to be slightly verbose. Major analysis goals/outcomes are not clear and also not validated. Is this a resource, or an analysis paper?
RE: we appreciate reviewer's comments and addressed them point-by-point in the letter of response.
Major comments -
1. The design is a bit confusing with 10 plex TMT used for labelling only 7 samples. Though the authors have written about three samples not being used, why label those? Was the study used a TMT 10-plex of which only 7 labels were mixed for MS analysis? Or all 10 were mixed, but analysis was represented for only 7? Why were these 3 samples left out of analysis?
RE: We apologize for not having clearly described the TMT experiments' study design. In this study, to accommodate four biological replicates from seven different sampling points (0, 8, 12, 20, 50, 80, and 120 days) we performed three interdependent TMT experiments. Each TMT experiment contained the

same technical replicate (d0; labelled with TMT 126 reagent) as a common reference and at least one biological replicate per sampling point as illustrated in the Figure 1. The remaining two empty channels in each TMT experiment were assigned to the 4th biological replicate per sampling points. We have made the experimental design clear by providing a table containing the exact details of the labels and sample identifications (Fig. 1). In our preliminary PCA analysis, we noticed inconsistency with our 4th replicate (unknown reason); therefore, for each time point, the pairwise correlation of the replicates was computed by the Pearson correlation coefficients (PCCs) method and based on the result (presented in Supplementary Table S2) we decided to present the most correlated triplicates for the further analysis. Furthermore, day 50 (early-OPC stage) was not further considered in the analysis due to its remarkable similarity to day 80 (OPC stage) in our preliminary Pearson correlation coefficient analysis (explained in the first paragraph at “Tandem mass tag (TMT) labeling” section and in “TMT data analysis” section).
To address the reviewer comments, we have revised the Supplementary Table S2 and refined Figure 1, along with the first 13 lines in“Tandem mass tag (TMT) labeling,” and “TMT data analysis” sections, to better elucidate how the TMT data was analysed in the current study.
2. Describing known OL differentiation stages in depth by the proteome is a big knowledge addition. However, the rationale of using important proteins as biomarkers is not clear. If the stages are well defined, what is the need for further biomarkers? Is it clinically relevant?
RE: Thanks for raising an important point – To address this we have added the following statements in the manuscript, in the “Identification of potential biomarkers in each step of human embryonic stem cell (hESC) differentiation into oligodendrocytes (OLs)” section, line 344-354: “In this study, we used TMT-based quantitative proteomics to discover important proteins involved in OL lineage differentiation. Furthermore, we exploited this approach to identify novel potential biomarkers that improve the selection, tracking and monitoring of each specific cell type of OL lineage. Currently, the majority of the markers in OL lineage are not specific to one cell type or one differentiation stage. Hence, the expression pattern of a panel of markers during differentiation process is usually being used, especially in in vitro studies [1]. For instance, LIN28A a known marker of ESCs (Figure 3D) is also a marker of stemness which is highly expressed in NSCs [2–4]. This is also true for MSI1, a marker of NSPCs, which is also a well-known marker for adult stem cells and progenitors in hair follicles, mammary glands, and intestine [5–8]. MSI1 is also considered as a prognostic biomarker in various human malignancies [9].”
3. The selection/discovery of Wnt signaling pathways and PCP pathways was not clear from figures 3A and B or analysis as claimed on page 8 para 2.
RE: We agree with the comment and apologies for the confusion. We prepared a new Supplementary Table S4 to address the reviewer’s comment by presenting the major functional groups of each cluster in Figure 3.
4. There is plenty of literature for PCP pathway in OL developmental stages, however the authors claimed the opposite on page 9, para 2. Please clarify any nuanced point missed here.
RE: We corrected this discrepancy and added the three main relevant studies in the revised manuscript as follows, line 256-269: “This non-canonical Wnt signaling pathway has been shown to maintain the NSCs of the subventricular zone (SVZ, located in the periventricular region), in the quiescence state. Chavali et al. (2018) demonstrated that a shift in PCP to canonical Wnt activity and keeping their balance induces NSC activation and lineage progression toward the generation of the progenitors that eventually go on to participate in the repair process [10]. It was also well demonstrated by Jarjour et al. (2015 and 2020) that the PCP pathway is involved in the myelination initiation, and structural organization of axonal myelin sheath [11,12]. Along with these reports, our finding shows that the high abundance of PCP pathway-related proteins in the middle stages of OL lineage differentiation (Fig. 4A and Supplementary Table S5) may convey the necessity of polarization in OPC specification, migration, and differentiation; which would need additional functional investigation as there cannot be found any study that explored the PCP pathway in pre-OPC and OPC generation and their differentiation into OL.”
Along with this revision, we also revised the last sentence of the last paragraph in “Canonical and non-canonical Wnt signaling protein profiling during the generation of the oligodendrocyte (OL) lineage cells” section, line 273-278: “These results can potentially improve our knowledge about the implications of all of the Wnt signaling pathways in hESCs maintenance and its further differentiation into the particularly OL lineage; specification, migration, and differentiation of OPCs; and OL maturation and myelination, with the ultimate goal of recruiting pre-OPCs and OPCs to the demyelinated regions and achieving their OL differentiation, which may result in restoring myelin regeneration in diseases associated with myelin deficiencies.”
5. How is this data useful for targeted drug delivery as a resource? Some use case may be explained (page 11 end of para 2).
RE: To address this comment and for further clarification, we revised the “Macroautophagy-associated protein profile of the generation of oligodendrocyte (OL) lineage cells” section. In the last sentence of the third paragraph, we mentioned, line 319-322: “Therefore, this may provide researchers with a tremendous repository for a better understanding of the molecular mechanism of autophagy in OL

lineage, and designing more effective therapeutic strategies toward demyelination disorders." Then we clarified the use of our data for targeted drug delivery as a resource by adding the following sentences, line 334-341: "particularly with the aim of directly inducing autophagy in OPCs and OLs. Therefore, the induced autophagy would promote these cells function in clearing cellular and myelin debris, protein aggresomes, and their development toward remyelination achievement [13–15]. Currently, the pharmaceutical compounds which are used for autophagy induction may target signaling pathways other than autophagy [16]. One potential strategy to minimize the undesirable side effects is to identifying more specific autophagy regulators and mechanism in every cell type would help to achieve targeted autophagy modulations [16,17]."
For this section, we also provided "Supplementary Table S6" which contains the tabular information of the relative expression of the autophagy associated proteins:
6. The data should be available in an accessible format (webpage or server) where users can explore easily without having to reanalyse everything in detail. Although, this is only a suggestion, it would immensely increase the utility of the work for researchers in the field.
RE: To address the reviewer's comments, we provided the manuscript with several new supplementary tables illustrating our step by step TMT data analysis procedure. These supplementary tables involve:
Table S2: Protein identification, TMT reporter ion ratios, protein quantitation, and study design. '171102', '171108' and '171105' sheets represent the data acquired by the first, second and third TMT experiments. 'Aggregated Data' table includes the whole identified proteins of the three replicates in one table. The 'Study Design' table shows the TMT experimental design and the 'Replicates Correlation' table illustrates the Pearson correlation coefficients of the replicates. The analysis of variance (ANOVA, in the 'Aggregated Data' table) represents 3132 proteins (about 81% of the whole identified proteins) that showed significant changes (adjusted p-value ≤ 0.05) through the OL lineage differentiation of hESCs. Based on the Pearson correlation coefficient analysis, 'd0_r1' was left out of the study. In addition, we decided to present the most correlated triplicates for the further analysis.
Table S3: The first table (Quantified Proteins) shows the relative expression of the whole identified and quantified proteins in every biological replicate of each time point during OL lineage differentiation. The second table (Variable Loadings Matrix) includes the details of the PCA of the proteome profile of each differentiation stage in Fig. 2B. The third table (Contribution of PCs) reveals the contribution of computed PCs, among which we chose PC1 and PC2 for the illustration of the PCA analysis in Fig. 2B. The fourth table (Averages of the Replicates) contains the total of 3,855 quantified proteins along with the average of their relative expression with respect to each differentiation stage. The following analyses were conducted on 'Averages of the Replicates' table.
7. Lack of any validation for novel proteins/ pathways in the discovered list.
RE: This manuscript has been submitted as a data note not the full research paper. Therefore, validation of subset of proteins/pathways would not be required based on the journal format/requirement.
8. Methods for Fold change and TMT quant are unclear. Whether ratio or intensity were used to merge biological replicates? If outliers were removed before protein quant summarization?
RE: We apologize for the lack of clarity. To make our method of data processing and TMT data analysis clear we revised the "Data processing and protein identification" section and rewrote the "TMT data analysis" section. Below you can see the changes in regard to this comment.
In "Data processing and protein identification" section, line 733-737, we wrote: "In each TMT experiment, relative quantitation of protein abundances was achieved by pairwise comparison of TMT reporter ion signal to noise (S/N) ratios as the ratio S/N of the label for each of the differentiation time points versus the label of the internal control (labelled with TMT 126 reagent) of the corresponding run (Supplementary Table S2)."
Then the "TMT data analysis" section starts with: "Relative quantitation of all protein abundances, in every differentiation time points, with respect to the reference (labelled with TMT 126 reagent), were extracted from the three TMT experiments and were aggregated into a single report (Supplementary Table S2)."
In "TMT data analysis" section, line 751-759, we wrote: "The proteins that were only quantified in some time points of one TMT experiment, the proteins that were quantified in only one TMT experiment, and the proteins that were quantified in two TMT experiments, but not in all time points were excluded from the study. Then, 'missForest' function from R package 'missForest' [18,19] was applied for the imputation of those identified proteins that were quantified in all time points of two TMT mass spectrometry experiments and their relative expressions were not quantified in some or all time points of the other TMT experiment. As a result of this supervised approach, the missing expression of 336 proteins was imputed and the "Quantified Proteins" table in Supplementary Table S3 was achieved."
In "TMT data analysis" section, line 777-780, we wrote: "The averages of the relative protein expression of all quantified proteins, in three replicates, were calculated by the 'mean' function and the following analyses were performed on the achieved dataset (Supplementary Table S3) [20]."
Minor comments -

1. The biomarker list/important DEPs- are these same?
RE: The potential biomarkers are the stage-specific proteins that are included in the list of DEPs. These proteins were differentially expressed only in one of the differentiation stages.
To make this analysis approach clear, in "TMT data analysis" section, line 814-832, we wrote: "In order to find specific proteins of each differentiation stage, that were differentially expressed in only one time point in comparison to all other time points (Supplementary Table S3), we conducted a differentially expression analysis by applying R/Bioconductor package 'limma' version 3.34.9 [21]. Moreover, due to the time-series nature of our study, we analysed the differentially expression of each protein (Supplementary Table S3) between two sequential time points by applying R/Bioconductor package 'limma' [21]; using 'lmFit', 'makeContrasts', 'contrasts.fit', 'ebayes' and 'toptable' functions from the package 'limma' in both differentially expression analysis procedures [21]. Proteins having absolute log2 fold-change higher than 1 and Benjamini-Hochberg adjusted p-value less than 0.05 were considered as significant differentially expressed proteins (DEPs) (Supplementary Table S7 and S8). The DEP analyses were illustrated by volcano plots (Fig. 6A–F and 7A–E). The stage-specific proteins where also demonstrated in a heat map (Supplementary Fig. S5) using 'pheatmap' function with the scale argument set at 'row', and 'cluster_cols, and 'cluster_rows' arguments set at 'FALSE'. Finally, for the illustration of the STSPs in Fig. 7F, we used a built-in doughnut chart type in Excel, however, the circular visualization of the protein members of each slice was performed by setting 'circos.par', 'circos.initialize', 'circos.track' and 'circos.trackText' functions of the package 'circlize' version 0.4.8 [22]."
2. If these important proteins can be tabulated, it can be useful.
RE: According to this comment, we combined "Supporting_data_for_Figure_6" and "Supporting_data_for_Supplementary_Figure_S5" and presented the data in Supplementary Table S7.
Table S7: The first six sheets (named Fig. 6A-F) provide the tabular illustration of the stage-specific proteins of the oligodendrocyte lineage differentiation. The table in 'Fig. S5' sheet demonstrates the relative expression changes of the stage-specific proteins. Each table is a supporting data for the figure with the same name. For the identification of the stage-specific proteins, the expression of each protein at a specific time point was compared by its expression in all other time points using the R/Bioconductor package 'limma'.
We also presented the "Supporting_data_for_Figure_7" as Supplementary Table S8, which involves the tabulated data of Figure 7.
Table S8: The first five sheets (named Fig.7A-E) provide the tabular illustration of the stage transition specific proteins (STSPs) in the oligodendrocyte lineage differentiation. Each sheet is supporting data for the figure with the same name. In this analysis, the expression of each protein at a specific time point was compared by its expression at the next time point using the R/Bioconductor package 'limma'. The demonstrated table in the sixth sheet (Fig.7F) involves the relative expression changes of all STSPs, the proteins that have been shown in Fig. 7F.
3. Rationale of Protein z-score formula is unclear.
RE: We apologize for the mistake. We have not calculated the protein z-score and removed the relevant sentence (the second sentence in "TMT data analysis" section) in the revised manuscript.
4. Whether the cut off of 1.5 (TMT analysis beginning, page 24) or FC 2 was used (Log FC ±1 is stated later) to define DEPs?
RE: We thank the reviewer for bringing this good point. FC 2 was used (Log FC ±1 is stated later) to define DEPs. The first sentence of this section has been removed.
5. Whether the Heatmaps use the z-score or FC or log2FC is not clear? Color legend in all figures bears no caption to inform about this.
RE: We apologize for the lack of clarity. Raw z scores were used to draw heat maps (except for Fig. 2A which illustrates the Pearson correlation coefficients). Figures, figure legends and text (in "TMT data analysis" section) have been revised accordingly.
Therefore, in Fig 2A we added "Pearson" as the caption, next to the colour legend. And in all other heat maps (Fig. 3D, 4A, 5 and Supplementary Figure S5) we added "Row z-score" as the caption, next to the colour legends.
For the latter group, we also revised the figure legends and mentioned there that heat maps show the standardized relative protein expression changes. Furthermore, in the "TMT data analysis" section we also explained how the heat maps were drawn. There we noted that we used 'pheatmap' function from the 'pheatmap' package with the 'scale' arguments set at 'row'.
6. Figure 4 either has no column/condition labels, or it is cropped.
RE: We inserted the label.
7. Figure 6, showing only stage specific proteins on a volcano plot gives a confusing/biased picture. It can be better given as a table. It may be better to change representation or data, based on the message that the authors wish to convey.
RE: We agree with the reviewer and have added these details in the Supplementary Table S7. This supplementary table contains six tables, each of which is a supportive data for one of the volcano plots

in Figure 6. It also involves a supporting data for Figure S5 which illustrates the relative expression changes of these stage-specific proteins.

Reviewer #2:
Paria Pooyan et. al. present the study titled: The Dynamic Proteome of Oligodendrocyte Lineage Differentiation Features Planar Cell Polarity and Macroautophagy Pathways. The authors utilize powerful TMT-quantitative proteomics approaches to understand the proteome changes during the differentiation of human pluripotent stem cells into oligodendrocytes and generate samples of the intermediate differentiation stages at defined time-points. This results in a comprehensive protein expression profile showing differential protein expression over the course of human oligodendrocyte lineage differentiation. This is a resource that will be interesting/useful for other researchers in the field and would be appropriately placed for a publication in GigaScience. The data analysis is understandable. Enthusiasm for the study is limited by a lack of characterization of the differentiating cells and lack of validation of the findings.
RE: We appreciate reviewer's comments and addressed them point-by-point in the letter of response.

The authors cite the differentiation protocols established in the Fossati lab as the basis for their own differentiation approach. The success of the differentiation is hard to judge from the provided data (Fig. S1) and none of the immunocytochemistry stains have been quantified. It is hard to know what percentage of the cells differentiated into oligodendrocytes.
- The authors use SOX1 as a marker for neuronal stem cells and show almost uniform expression. In one of the three cited publications SOX1 was used as well to define the NSCs (Epigenetic Modulation of Human Induced Pluripotent Stem Cell Differentiation to Oligodendrocytes) but the staining pattern looks different at the same time-point (only present in some of the differentiating cells). It would be good to see the SOX1 stain in controls using pluripotent stem cells and other time-points.
RE: In regard to applying the differentiation protocol established in Dr. Fossati's lab, in our laboratory and on the RH6 cell line, we had to make some adjustments. Douvaras et al. (2014 and 2015) started the differentiation induction (d0), 24 hours after plating PSCs with the density of 1x104 cells/cm2, by applying mTeSR Custom medium, containing the small molecules SB431542 10µM and LDN193189 250nM as well as 100nM all-trans-retinoic acid [23,24]. However, based on Piao et al. (2015) [25], we replaced the mTeSR custom medium with NI medium (Supplementary Table S9); nevertheless, we applied the same small molecules and morphogen at the exact concentrations as Douvaras et al. (2014 and 2015). Next, we found that RH6 cells had to be completely confluent at the time we start the differentiation, otherwise, cells would start to be degraded from the point that they had no cell to cell attachment. We revised the manuscript, and in the first sentence of the "Human embryonic stem cell (hESC) differentiation into oligodendrocyte (OL) lineage cells" section, mentioned the confluent culture condition of RH6: "Adherent confluent RH6 cells (passages 45, 48 and 50, to accommodate the three biological replicates) were induced into SOX1+ NSCs by dual inhibition of SMAD signaling [9,171]."
As a result of this differentiation procedure, the massive stem cells' proliferation through the hESCs transition to SOX1+ NSCs caused the culture to become over confluent and resulted in the formation of the 3D structure by day 8; which made it impossible to count the immunostained cells at this differentiation stage.
In their Nature Protocols article, Douvaras and Fossati (2015) stained their achieved cells on day 8 of the differentiation by PAX6, which is another marker for NSCs. They mentioned that at this time point of the differentiation, the population of the PAX6+ cells should be more than 90% [24]. Instead of PAX6, we used another early neuroectodermal marker Sox1 [26], which did show to be expressed in more than 90% of the generated cells on NSC (d8) stage.
We do not have negative control for SOX1 immunostaining, but as it is presented in Supplementary Fig. S6A, we immunostained the generated cells on NSC (d8) stage by CDH2 (N-cadherin), which also shows almost uniform expression. We also had stained a positive control NSC line, RSCB0181, by these two antibodies (Supplementary Fig. S6B-C).
- Staining of oligodendrocyte markers SOX10 would be important across all time-points as well.
RE: According to our references [23,24], the generated NSCs (d8) will give rise to OLIG2+ NPCs by day 12. Next, the OLIG2+ progenitors be differentiated into NKX2.2+ pre-OPCs. This transition will be followed by the generation of the SOX10 expressing cells around day 40. Therefore, we did not stain all the time points with SOX10. However, we co-stained the generated cells on OPC (d80) stage with SOX10 and PDGFRA antibodies. Supplementary Fig. S6D-L represents the co-stained OPCs with SOX10 and PDGFRA antibodies, in the three differentiation replicates. Counting these stained cells, we found that about 100% of the cells were PDGF+ and about 97% of them were SOX10+.
Here we should mention another change we made in our differentiation protocol to achieve the most homogeneous population of OPCs. As it has been mentioned in "Human embryonic stem cell (hESC) differentiation into oligodendrocyte (OL) lineage cells" section: "In order to eliminate the neurons and

astrocytes that migrated out of the spherical aggregates and to achieve a homogenous population of OPCs, we re-plated the cells twice onto poly-L-ornithine/laminin-coated dishes, on day 65 and on day 75 of differentiation."
- Similarly, the OLIG2 and NKX2.2 staining are labeling 100% of the cells while the original publication shows a mixture of negative, single and double positive cells. Ideally these should be dual stains with appropriate controls and quantification of the co-expression. The results should be discussed, and differences or improvements made to the original differentiation protocol should be explained.
RE: As it has been mentioned in response to your comment on SOX1+ cells, we had made some changes in our reference protocol to adjust it in our lab and on our cell line of choice. Please see our step by step differentiation protocol published in protocols.io [27].
We did not perform double staining for the NPC and pre-OPC states. However, for the NPC stage (d12) immunostaining, we also used another OLIG2 antibody (ab81093), the result of which is presented in Supplementary Fig. S6M. Moreover, at this differentiation stage (d12), due to the 3D structure of the over confluent cells, we could not count the OLIG2+ cells.
Furthermore, for pre-OPC stage (d20), we presented two other immunostained sections of the day 20 aggregates in Supplementary Fig. S6N-O. We counted NKX2.2+ cells in five different fields of the three presented NKX2.2 immunostained photos and found that, in total, about 90% of the cells were expressing this protein.
- The MBP staining shows typical oligodendrocyte morphology and the correct staining pattern but is only presented in high resolution. It would be good to see a low-resolution image taken from all replicates and a quantification of the differentiation percentage should be conducted.
RE: Supplementary Fig. S6P-W presents MBP stained OLs in all replicates, besides an image (Supplementary Fig. S6S and W) with the lower resolution. Counting MBP+ cells in the four presented photos, we found that more than 22% of the cells at day 120 were expressing this mature oligodendrocyte marker.
- It is unclear, what percentage of cells are oligodendrocytes and thus it is unclear what is measured in the proteomics experiments. If the percentage of the differentiation is high the mixed culture is appropriate for these experiments. If the percentage is low, it might be important to sort the positive cells which should be possible using available antibodies.
RE: As mentioned above, at OL stage, more than 22% of the cells were expressing this mature OL marker. Unfortunately, we could not stain our cells with earlier OL markers, however, based on the quantification of the PDGFRA+ OPCs (mentioned above), and in regard to high expression of the oligodendrocyte markers illustrated in Figure 3D, which was achieved by the sensitive and precise TMT-based quantitative proteomics, and in accordance with the phase contrast photos of the cells of the OL stage (represented in three replicates in Supplementary Fig. S6X-Z), we believe a considerable number of oligodendrocytes in OL stage were early generated oligodendrocytes that were not mature enough to express MBP protein.
To our knowledge, our reference protocol is one of the most efficient published protocols of human pluripotent stem cell (PSC) differentiation into OLs. In their both publications, Douvaras et al., at most, achieved >35% MBP+ cells at the OL stage [23,24]. They also showed that their cultures consisted of 15% ± 2% GFAP+ astrocytes and 20% ± 2% MAP2+ neurons of total cells [23]. Considering that suitable conditions to expand purified human OLs in the absence of supporting astrocytes and neurons have not yet been established; and the fact that the available human OL differentiation protocols of the human pluripotent stem cells are on the basis of knowledge gained from studies of rodent embryonic development, we believe describing OL differentiation stages in depth by the proteome data can provide the scientists with the great knowledge for deeper understanding of major functional players during the OL lineage differentiation and major proteins of each differentiation stages. Due to these reasons, in our manuscript, we tried to always mention the differentiation stages not the particular cell type; as these stages are needed to achieve mature human OL in vitro.
- The authors write that the experiments were conducted using three biological replicates, but it is unclear how this was defined as only one cell line was used. Did they use three clonal cell lines or differentiation replicates, or did they conduct one differentiation at the same time but in different plates?
RE: We apologize for the lack of clarity. We have now clarified how the biological replicates were defined by revising the first sentence in "Human embryonic stem cell (hESC) differentiation into oligodendrocyte (OL) lineage cells" section: "Adherent confluent RH6 cells (passages 45, 48 and 50, to accommodate the three biological replicates) were induced into SOX1+ NSCs by dual inhibition of SMAD signaling [9,171]."
- Could the experiments be repeated using other (genetically distinct) control cell lines? It might be too much to ask for another time-course, but it would be great to compare add one early (pluripotent or d8 as well as the late-timepoint) using TMT-proteomics. Overlap across cell lines would increase the statistical significance of the presented findings and make this resource much more valuable to the community.
RE: We agree that including further cell lines may increase the value of any manuscript but

unfortunately this is not feasible at this stage as our laboratory is not functioning due to Covid-19 pandemic. So, we appreciate if the reviewer accepts our extensively revised manuscript at its current form.
Validation of a few of the most important and identified drivers of differentiation and newly identified DEPs that are hypothesized to play a role in oligodendrocytes should be tested using immunocytochemistry in cultures co-stained with MBP.
RE: This manuscript has been submitted as Data Note not full research paper. So, further functional analysis is not essential based on the journal format/requirement.

Close
