# Supplementary material for: The Dynamic Proteome of Oligodendrocyte Lineage Differentiation Features Planar Cell Polarity and Macroautophagy Pathways
Source: Gigascience. 2020 Oct 31;9(11):giaa116. doi: 10.1093/gigascience/giaa116 (PMC7601170; doi:10.1093/gigascience/giaa116)
Supplement: giaa116_Response_to_Reviewer_Comments_Revision_1 [file giaa116_response_to_reviewer_comments_revision_1.pdf]

Author's Response To Reviewer Comments

Close

Reply to Reviewer:

Reviewer #1:
Authors have done a good job in addressing the concerns raised.
I have few minor points-
1. The data is still not publicly accessible which defeats the purpose of submitting it to a public data repository.

Re: To make the data publicly available, PRIDE requires DOI or PubmedID. As soon as we received one of these, we will make the data public.

2. Targeted drug delivery means focusing on specific organ/tissue for drug delivery. Probably the authors meant pathway targeting instead. I would suggest correcting this ambiguous terminology.

Re: we agree with reviewer's comment and replaced 'targeted drug delivery' with 'pathway targeting'.

3. Reporter ion quantitation using S/N ratio as the quantitation is something I have not heard/read. Either the authors meant something else or I missed the point.

Re: In our analysis pipeline, to filter reporter ion quantification input data that is detected from a number of ions that is too small we use the signal to noise ratio (S/N) values of the reporter ion peaks instead of their intensities, especially when data is acquired using Orbitraps (Hogreben et al., 20018. Nature Communications volume 9, 1045)(Mirzaei et al., 2019, Molecular Neurobiology volume 56, 6017–6034). Proteome discoverer adopts this strategy.

Close
