# Supplementary material for: The Dynamic Proteome of Oligodendrocyte Lineage Differentiation Features Planar Cell Polarity and Macroautophagy Pathways
Source: Gigascience. 2020 Oct 31;9(11):giaa116. doi: 10.1093/gigascience/giaa116 (PMC7601170; doi:10.1093/gigascience/giaa116)
Supplement: giaa116_Response_to_Reviewer_Comments_Revision_2 [file giaa116_response_to_reviewer_comments_revision_2.pdf]

Author's Response To Reviewer Comments

Close

Response to the editorial comments
1. AU: Please add the following sentence here:
"Other data further supporting this work are openly available in the GigaScience respository, GigaDB [192]."
RE: done!
2. AU: Please add new Reference #192 and cite the DOI exactly as follows:
Pooyan P; Karamzadeh R; Mirzaei M; Meyfour A; Amirkhan A; Wu Y; Gupta V; Baharvand H; Javan M; Salekdeh GH: Supporting data for "The Dynamic 192. Proteome of Oligodendrocyte Lineage Differentiation Features Planar Cell Polarity and Macroautophagy Pathways" GigaScience Database. 2020. http://dx.doi.org/10.5524/100798
RE: The new reference and its DOI was added as instructed
3. Added edits
We also edited the following parts:
On page 22, line 625: the misplaced parenthesis was removed.
On page 31, line 893: the legend text for Fig. S6C was clarified by adding the letter C.
On page 31, line 895: the PDGF protein name is now written in its standard format “PDGFRA”

Close
