# Supplementary material for: The Dynamic Proteome of Oligodendrocyte Lineage Differentiation Features Planar Cell Polarity and Macroautophagy Pathways
Source: Gigascience. 2020 Oct 31;9(11):giaa116. doi: 10.1093/gigascience/giaa116 (PMC7601170; doi:10.1093/gigascience/giaa116)
Supplement: giaa116_Reviewer_1_Report_Original_Submission — Amit Yadav -- 4/17/2020 Reviewed [file giaa116_reviewer_1_report_original_submission.pdf]

Reviewer Report

Title: The Dynamic Proteome of Oligodendrocyte Lineage Differentiation Features Planar Cell Polarity and Macroautophagy Pathways

Version: Original Submission **Date:** 4/17/2020

Reviewer name: Amit Yadav

Reviewer Comments to Author:

The manuscript by Pooyan et al describes TMT-based quantitative proteome of oligodendrocyte lineage (OL) differentiation by analyzing human embryonic stem cells (hESCs) through several developmental OL stages. Using clustering and ontological analyses, the authors found hundreds of proteins that were differentially expressed between these stages. These proteins pointed towards the importance of Planar Cell Polarity (PCP) and macro-autophagy in OL differentiation.
Strengths -
Generally, the manuscript is well written and the objective is clear. This seems to be a comprehensive proteomics approach to study OL differentiation. The comprehensive multiplexed proteomics study is the big strength of the manuscript. The data is deposited in public repository.
Limitations-
The rationale for biomarkers was not clear. Some analysis methods/steps are not explained in requisite detail for a general user. This data serves as a rich resource as claimed by authors, but it is not available for users in an easily digestible/usable format. Also, the biological description of proteins was felt to be slightly verbose. Major analysis goals/outcomes are not clear and also not validated. Is this a resource, or an analysis paper?
Major comments -

1. The design is a bit confusing with 10 plex TMT used for labelling only 7 samples. Though the authors have written about three samples not being used, why label those? Was the study used a TMT 10-plex of which only 7 labels were mixed for MS analysis? Or all 10 were mixed, but analysis was represented for only 7? Why were these 3 samples left out of analysis?
2. Describing known OL differentiation stages in depth by the proteome is a big knowledge addition. However, the rationale of using important proteins as biomarkers is not clear. If the stages are well defined, what is the need for further biomarkers? Is it clinically relevant?
3. The selection/discovery of Wnt signaling pathways and PCP pathways was not clear from figures 3A and B or analysis as claimed on page 8 para 2.
4. There is plenty of literature for PCP pathway in OL developmental stages, however the authors claimed the opposite on page 9, para 2. Please clarify any nuanced point missed here.
5. How is this data useful for targeted drug delivery as a resource? Some use case may be explained (page 11 end of para 2).
6. The data should be available in an accessible format (webpage or server) where users can explore easily without having to reanalyse everything in detail. Although, this is only a suggestion, it would immensely increase the utility of the work for researchers in the field.

7. Lack of any validation for novel proteins/ pathways in the discovered list.
8. Methods for Fold change and TMT quant are unclear. Whether ratio or intensity were used to merge biological replicates? If outliers were removed before protein quant summarization?

Minor comments -

1. The biomarker list/important DEPs- are these same?
2. If these important proteins can be tabulated, it can be useful.
3. Rationale of Protein z-score formula is unclear.
4. Whether the cut off of 1.5 (TMT analysis beginning, page 24) or FC 2 was used (Log FC Â±1 is stated later) to define DEPs?
5. Whether the Heatmaps use the z-score or FC or log2FC is not clear? Color legend in all figures bears no caption to inform about this.
6. Figure 4 either has no column/condition labels, or it is cropped.
7. Figure 6, showing only stage specific proteins on a volcano plot gives a confusing/biased picture. It can be better given as a table. It may be better to change representation or data, based on the message that the authors wish to convey.

Level of Interest

Please indicate how interesting you found the manuscript: Choose an item.

Quality of Written English

Please indicate the quality of language in the manuscript: Choose an item.

Declaration of Competing Interests

Please complete a declaration of competing interests, considering the following questions:

- Have you in the past five years received reimbursements, fees, funding, or salary from an organisation that may in any way gain or lose financially from the publication of this manuscript, either now or in the future?
- Do you hold any stocks or shares in an organisation that may in any way gain or lose financially from the publication of this manuscript, either now or in the future?
- Do you hold or are you currently applying for any patents relating to the content of the manuscript?
- Have you received reimbursements, fees, funding, or salary from an organization that holds or has applied for patents relating to the content of the manuscript?
- Do you have any other financial competing interests?
- Do you have any non-financial competing interests in relation to this paper?

If you can answer no to all of the above, write 'I declare that I have no competing interests' below. If your reply is yes to any, please give details below.

I declare that I have no competing interests

I agree to the open peer review policy of the journal. I understand that my name will be included on my report to the authors and, if the manuscript is accepted for publication, my named report including any attachments I upload will be posted on the website along with the authors' responses. I agree for my report to be made available under an Open Access Creative Commons CC-BY license (http://creativecommons.org/licenses/by/4.0/). I understand that any comments which I do not wish to be included in my named report can be included as confidential comments to the editors, which will not be published.

Choose an item.

To further support our reviewers, we have joined with Publons, where you can gain additional credit to further highlight your hard work (see: https://publons.com/journal/530/gigascience). On publication of this paper, your review will be automatically added to Publons, you can then choose whether or not to claim your Publons credit. I understand this statement.

Yes Choose an item.
