# Supplementary material for: The Dynamic Proteome of Oligodendrocyte Lineage Differentiation Features Planar Cell Polarity and Macroautophagy Pathways
Source: Gigascience. 2020 Oct 31;9(11):giaa116. doi: 10.1093/gigascience/giaa116 (PMC7601170; doi:10.1093/gigascience/giaa116)
Supplement: giaa116_Reviewer_2_Report_Original_Submission — Tim Ahfeldt -- 4/29/2020 Reviewed [file giaa116_reviewer_2_report_original_submission.pdf]

Reviewer Report

Title: The Dynamic Proteome of Oligodendrocyte Lineage Differentiation Features Planar Cell Polarity and Macroautophagy Pathways

Version: Original Submission **Date:** 4/29/2020

Reviewer name: Tim Ahfeldt

Reviewer Comments to Author:

Paria Pooyan et. al. present the study titled: The Dynamic Proteome of Oligodendrocyte Lineage Differentiation Features Planar Cell Polarity and Macroautophagy Pathways. The authors utilize powerful TMT-quantitative proteomics approaches to understand the proteome changes during the differentiation of human pluripotent stem cells into oligodendrocytes and generate samples of the intermediate differentiation stages at defined time-points. This results in a comprehensive protein expression profile showing differential protein expression over the course of human oligodendrocyte lineage differentiation. This is a resource that will be interesting/useful for other researchers in the field and would be appropriately placed for a publication in GigaScience. The data analysis is understandable. Enthusiasm for the study is limited by a lack of characterization of the differentiating cells and lack of validation of the findings.

The authors cite the differentiation protocols established in the Fossati lab as the basis for their own differentiation approach. The success of the differentiation is hard to judge from the provided data (Fig. S1) and none of the immunocytochemistry stains have been quantified. It is hard to know what percentage of the cells differentiated into oligodendrocytes. The authors use SOX1 as a marker for neuronal stem cells and show almost uniform expression. In one of the three cited publications SOX1 was used as well to define the NSCs (Epigenetic Modulation of Human Induced Pluripotent Stem Cell Differentiation to Oligodendrocytes) but the staining pattern looks different at the same time-point (only present in some of the differentiating cells). It would be good to see the SOX1 stain in controls using pluripotent stem cells and other time-points. Staining of oligodendrocyte markers SOX10 would be important across all time-points as well. Similarly, the OLIG2 and NKX2.2 staining are labeling 100% of the cells while the original publication shows a mixture of negative, single and double positive cells. Ideally these should be dual stains with appropriate controls and quantification of the co-expression. The results should be discussed, and differences or improvements made to the original differentiation protocol should be explained. The MBP staining shows typical oligodendrocyte morphology and the correct staining pattern but is only presented in high resolution. It would be good to see a low-resolution image taken from all replicates and a quantification of the differentiation percentage should be conducted.

It is unclear, what percentage of cells are oligodendrocytes and thus it is unclear what is measured in the proteomics experiments. If the percentage of the differentiation is high the mixed culture is appropriate for these experiments. If the percentage is low, it might be important to sort the positive cells which should be possible using available antibodies.

The authors write that the experiments were conducted using three biological replicates, but it is

unclear how this was defined as only one cell line was used. Did they use three clonal cell lines or differentiation replicates, or did they conduct one differentiation at the same time but in different plates?
Could the experiments be repeated using other (genetically distinct) control cell lines? It might be too much to ask for another time-course, but it would be great to compare add one early (pluripotent or d8 as well as the late-timepoint) using TMT-proteomics. Overlap across cell lines would increase the statistical significance of the presented findings and make this resource much more valuable to the community.
Validation of a few of the most important and identified drivers of differentiation and newly identified DEPs that are hypothesized to play a role in oligodendrocytes should be tested using immunocytochemistry in cultures co-stained with MBP.

Level of Interest

Please indicate how interesting you found the manuscript: Choose an item.

Quality of Written English

Please indicate the quality of language in the manuscript: Choose an item.

Declaration of Competing Interests

Please complete a declaration of competing interests, considering the following questions:

- Have you in the past five years received reimbursements, fees, funding, or salary from an organisation that may in any way gain or lose financially from the publication of this manuscript, either now or in the future?
- Do you hold any stocks or shares in an organisation that may in any way gain or lose financially from the publication of this manuscript, either now or in the future?
- Do you hold or are you currently applying for any patents relating to the content of the manuscript?
- Have you received reimbursements, fees, funding, or salary from an organization that holds or has applied for patents relating to the content of the manuscript?
- Do you have any other financial competing interests?
- Do you have any non-financial competing interests in relation to this paper?

If you can answer no to all of the above, write 'I declare that I have no competing interests' below. If your reply is yes to any, please give details below.

I declare that I have no competing interests

I agree to the open peer review policy of the journal. I understand that my name will be included on my report to the authors and, if the manuscript is accepted for publication, my named report including any attachments I upload will be posted on the website along with the authors' responses. I agree for my report to be made available under an Open Access Creative Commons CC-BY license (http://creativecommons.org/licenses/by/4.0/). I understand that any comments which I do not wish to be included in my named report can be included as confidential comments to the editors, which will not be published.

Choose an item.

To further support our reviewers, we have joined with Publons, where you can gain additional credit to further highlight your hard work (see: https://publons.com/journal/530/gigascience). On publication of this paper, your review will be automatically added to Publons, you can then choose whether or not to claim your Publons credit. I understand this statement.

Yes Choose an item.
